# Electrochemical Oxidation of Aryl Boronic Acids via Fluoride Activation and Alternating Polarity Electrolysis for Aryl C–P Bond Formation

**DOI:** 10.1002/celc.202500363

**Published:** 2025-11-07

**Authors:** Enqi Feng, Ian Vanswearingen, Maxime Boudjelel, Lise Fabre, Rossul Aldhufari, Christian A. Malapit

**Affiliations:** Department of Chemistry, Northwestern University, 2145 N Sheridan Road, Evanston, IL 60208, USA

**Keywords:** aryl boronic acids, aryl radicals, alternating polarity electrolysis, fluoroboronates, electrosynthesis

## Abstract

Aryl organoboron reagents play an important role in modern organic synthesis, and interest in radical-based coupling reactions from these precursors has grown rapidly. However, direct electrochemical generation of aryl radicals from aryl boronic acids, ArB(OH)_2_, remains understudied due to their high oxidation potentials (*E*_ox_ > 2 V vs. Fc/Fc^+^) and challenges associated with electrochemical processes such as electrode passivation. Aryl potassium trifluoroborate salts (ArBF_3_K) can be oxidized efficiently to aryl radicals is previously reported using alternating polarity electrolysis. Building on this, it is combined alternating polarity electrosynthesis with in situ fluoride activation to generate redox-active aryl fluoroborate intermediates, ArBF(OH)_2_ and/or ArBF_2_(OH), which have significantly lower oxidation potentials than their parent boronic acids or trifluoroborates. Moreover, it is found that for highly electron deficient aryl boronic acids with oxidation potentials higher than 1.7 V versus Fc/Fc^+^, a different mechanism is proposed where aryl boronic acids underwent *ipso*-substitution with oxidatively generated P(OEt)_3_ radical cation. Overall, this dual mechanistic pathway allows an efficient radical-based functionalization of a broad range of aryl boronic acids to form aryl C─P bonds.

## 1. Introduction

Aryl boronic acids are indispensable building blocks in synthetic chemistry and widely used in cross-coupling reactions such as Suzuki–Miyaura and Chan–Lam couplings to construct diverse medicinally relevant scaffolds.^[1–5]^ Despite their versatility, methods to access aryl radicals directly from aryl boronic acids remain underdeveloped. The high oxidation potential of these species makes single-electron oxidation difficult, and reported approaches typically require stoichiometric chemical oxidants (e.g., Mn(III), Ag(I), Fe(II/III) complexes)^[6–8]^ or photoredox catalysis with external mediators.^[9–12]^ Recently, Lei developed an electrochemical approach to generate alkyl radicals from alkyl boronic acids using a Mn electromediator, however, their attempt to translate this to aryl boronic acids proved to be challenging.^[13]^

**Figure 1.**
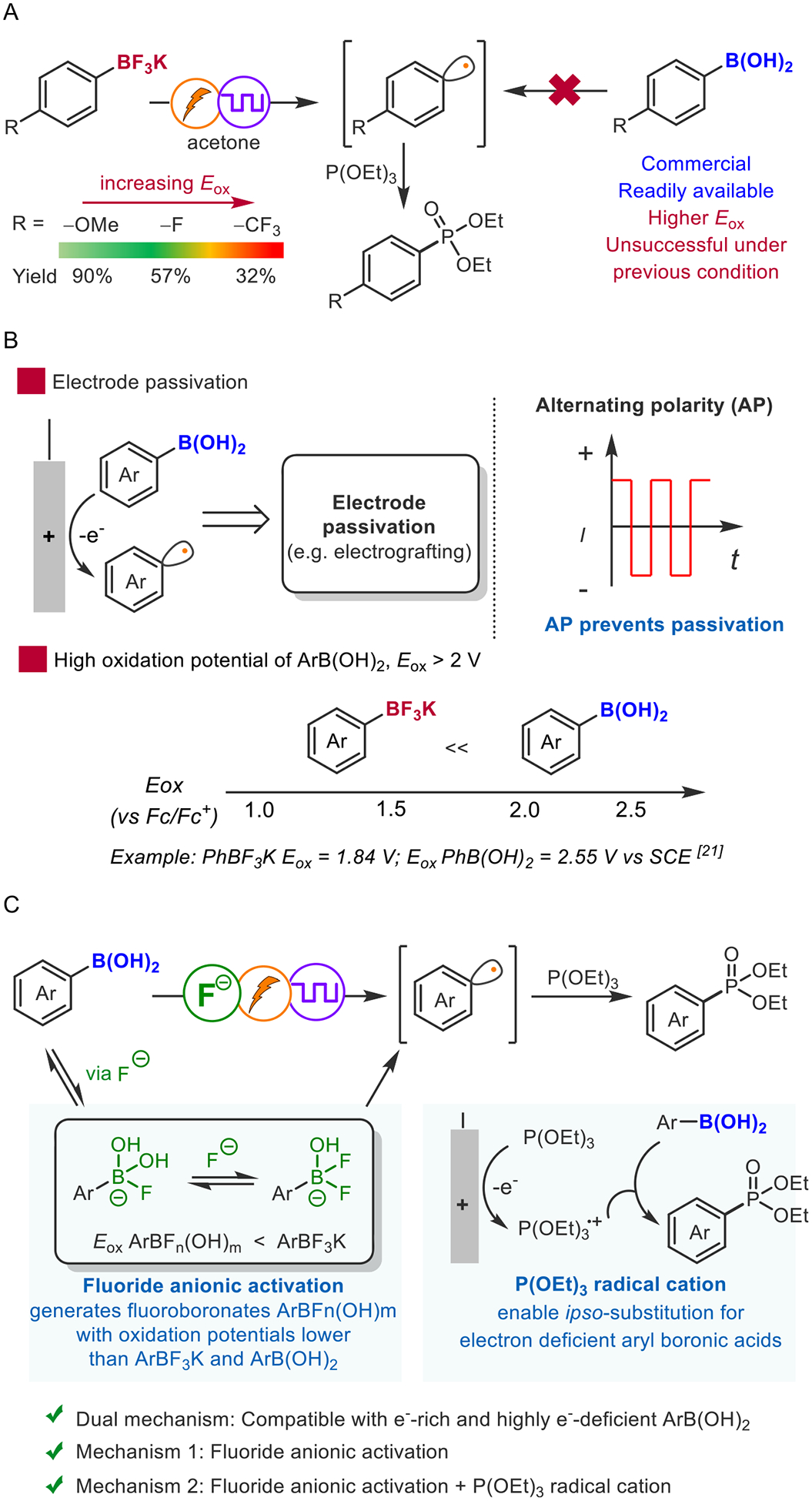
A–C) Electrochemical generation of aryl radicals from aromatic organoboron reagents: fundamental challenges and use of alternating polarity.

Owing to the sustainable advantages of electrosynthesis,^[14–20]^ the development of an electrochemical anodic oxidation of aryl boronic acids to efficiently generate aryl radicals would lead to an oxidant and metal-free approach. However, direct electrochemical oxidation of aryl boronic acids remained challenging, mainly due to their high oxidation potential^[21]^ and possible electrode passivation.^[22–24]^ Our group recently addressed these challenges with alternating polarity (AP) electrolysis, which efficiently generates aryl radicals from aryl trifluoroborates (Figure 1A).^[25]^ During the anodic phase, aryl trifluoroborates undergo single-electron oxidation to form aryl radicals, while the cathodic phase reduces acetone, and the continuous polarity reversal prevented electrode fouling. Although this platform is broadly effective for electron-rich and neutral aryl trifluoroborates, electron deficient aryl trifluoroborates and, more importantly aryl boronic acids remain ineffective, motivating further optimization to establish a metal- and oxidant-free platform for aryl radical generation and downstream bond-forming reactions.

Two fundamental challenges to enable aryl radical generation form aryl boronic acids include: 1) electrode passivation (most likely due to radical grafting),^[23]^ and 2) high oxidation potential of aryl boronic acids (*E*_ox_ > 2 V vs. Fc/Fc^+^). Recent studies in AP electrosynthesis offered new opportunities to better conduct redox reactions than direct current electrolysis.^[26–41]^ By optimizing current and frequency, we found that a suitable working potential could be applied and help in generating aryl radicals efficiently from aryl trifluoroboate salts.^[40,41]^ In previous studies, the quaternary center of boron was key to lowering the oxidation potential of organoboron reagents.^[21,25,42,43]^ Compared with aryl trifluoroborates, aryl boronic acids generally have higher potential and slower electrode kinetics according to cyclic voltammetry studies (Figure S2–S5, see Supporting Information for detail). Hence, in order to oxidize aryl boronic acids directly, we hypothesize that an exogenous nucleophilic anionic activator is needed to generate the quaternary boron intermediate in situ.^[43–46]^ Importantly, we hypothesize that the intermediates generated from the anionic activation have lower oxidation potentials than aryl boronic acids or potassium trifluoroborate salts.^[43]^ In this work, we utilized fluoride as an activator to boronic acids and no other chemical oxidants were added. P(OEt)_3_ was used to quench aryl radicals and form aryl C(sp^2^) P bonds. Two possible mechanisms were proposed for substrates with different oxidation potentials.

## 2. Results and Discussions

**Figure 2.**
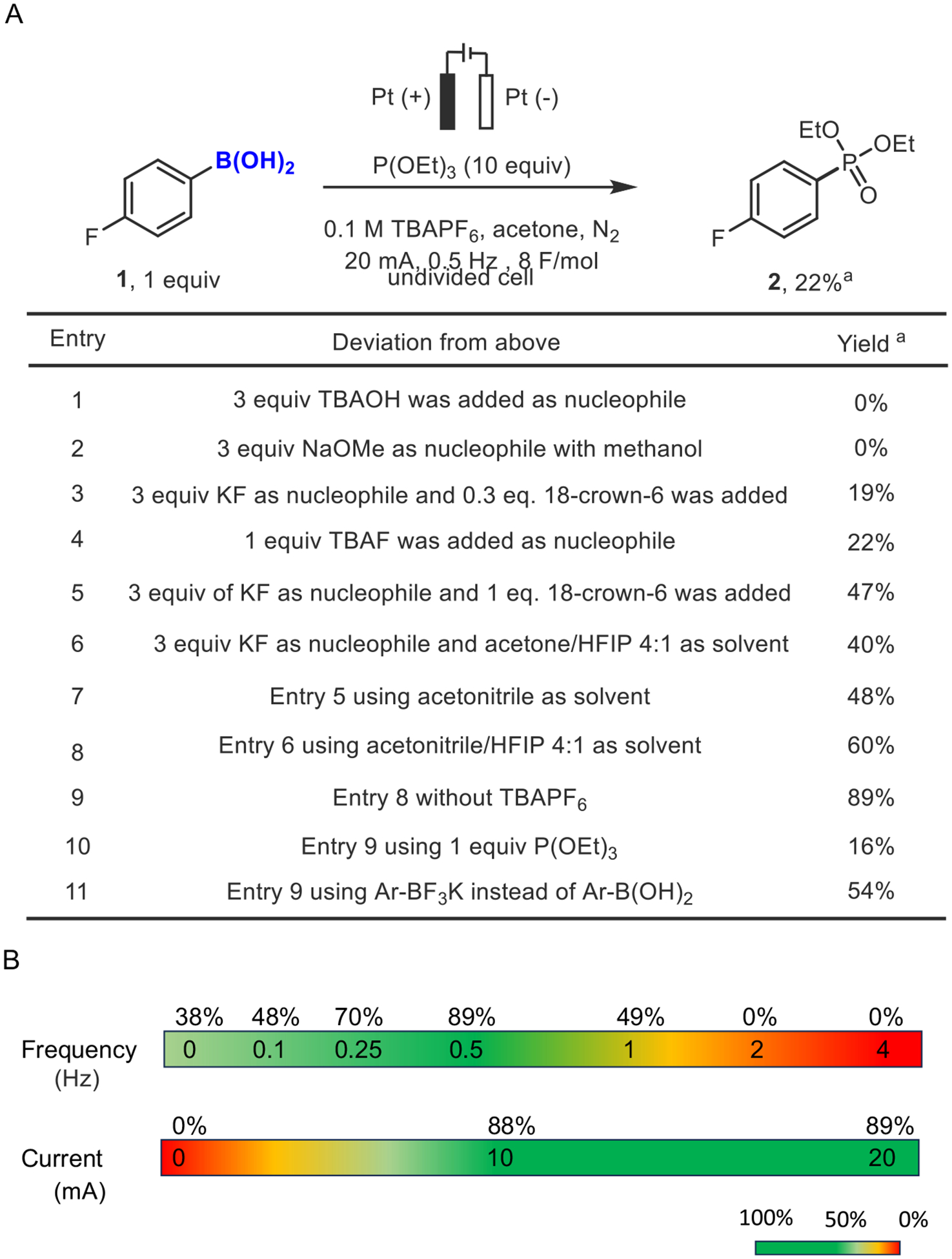
A,B) Optimization of reaction conditions on electrochemical oxidation of (4-fluorophenyl)boronic acid for aryl radical generation. ^a^Yields were determined by ^31^P NMR spectroscopy using triphenyl phosphine oxide as an internal standard.

To begin optimization, we selected (4-fluorophenyl)boronic acid **1** as model substrate, as its corresponding aryl BF_3_K salt had shown moderate yield (57%)^[25]^ under our previously reported aryl BF_3_K electrolysis conditions (Figure 2). When tested under these prior conditions, the yield is only 22% (crude NMR) highlighting the greater difficulty in oxidizing the boronic acid without activation. We first explored nucleophiles capable of quaternization of the boron center,^[43–46]^ focusing on −OR and −F groups. If hydroxide/methoxide were used as nucleophiles/activators, no desired product was found after the reaction conditions (Figure 2A, entry 1 and 2). A small amount of product started to form after fluoride reagents were added combined with phase transfer agents, which helped the solubility of inorganic salts in organic solvent. The use of 3 equivalents of potassium fluoride with 0.3 equivalents of 18-crown-6 (entry 3) or 1 equivalent of tetrabutylammonium fluoride (entry 4) both afforded about 20% yields. Moving forward, KF was chosen as the standard activator for its ease in handling and storage.

Next, phase transfer agents were screened between 18-crown-6^[47]^ and hexafluoroisopropanol (HFIP).^[48]^ If 1 equivalent of 18-crown-6 was added in acetone, the yield was increased to 47% (entry 5). When HFIP was used as phase transfer agent and cosolvent, 4:1 v/v acetone/HFIP (entry 6), 40% yield was obtained. The preliminary success found in using HFIP is exciting as HFIP can have multiple strategic roles: as phase transfer agent, as sacrificial reagent for reduction (via H^+^ reduction, onset *E*_red_ = −0.8 V vs. Fc/Fc^+^ in MeCN), and as cosolvent. After investigation of other solvents, we found significantly improved yields with acetonitrile (entries 7 and 8). Moreover, the use of KF (3 equivalents) as activator and electrolyte (in place of TBAPF_6_) resulted in stable working potential and an even higher yield (89%, entry 9), which indicated KF could function as both an activator and supporting electrolyte. Under the optimal conditions, if we lower down the amount of P(OEt)_3_ to 1 equivalent, the reaction yield dropped to only 16%, indicating large excess amount of radical trapping reagent is necessary in this reaction (entry 10, see also Supporting InformationTable S5).

Finally, we revisited other electrochemical conditions (Figure 2B). Under 20 mA, different frequencies were tested to show 0.5 Hz was the best for the reaction. It was worth mentioning that small change in frequency value (0.25 Hz to 0.5 Hz) can cause a 20% yield fluctuation, highlighting the relevance of alternating polarity. While the frequency value change may seem small, this is a large change with respect to polarity switching time (in seconds) (e.g. 0.2 Hz = 5 s, and 0.5 Hz = 2 s). This alternation in frequency effect will be applied in the optimization among strong electron poor substrates in this work. Under 0.5 Hz, a smaller current 10 mA was applied, and the reaction yield stayed at 88%. After rigorous optimization, the standard condition is described as: platinum plates as anode and cathode, 10 equivalents of triethyl phosphite as a radical quencher, MeCN/HFIP (4:1) as solvent, current at 20 mA and 0.5 Hz while passing 8 F mol^−1^ of charge. Interestingly, if we apply this standard condition on oxidation of potassium (4-fluorophenyl)trifluoroborate, the yield was only at 54% (Figure 2A, entry 11), indicating that other redox active reaction intermediates (instead of ArBF_3_K intermediates) are involved (see mechanistic studies below).

**Figure 3.**
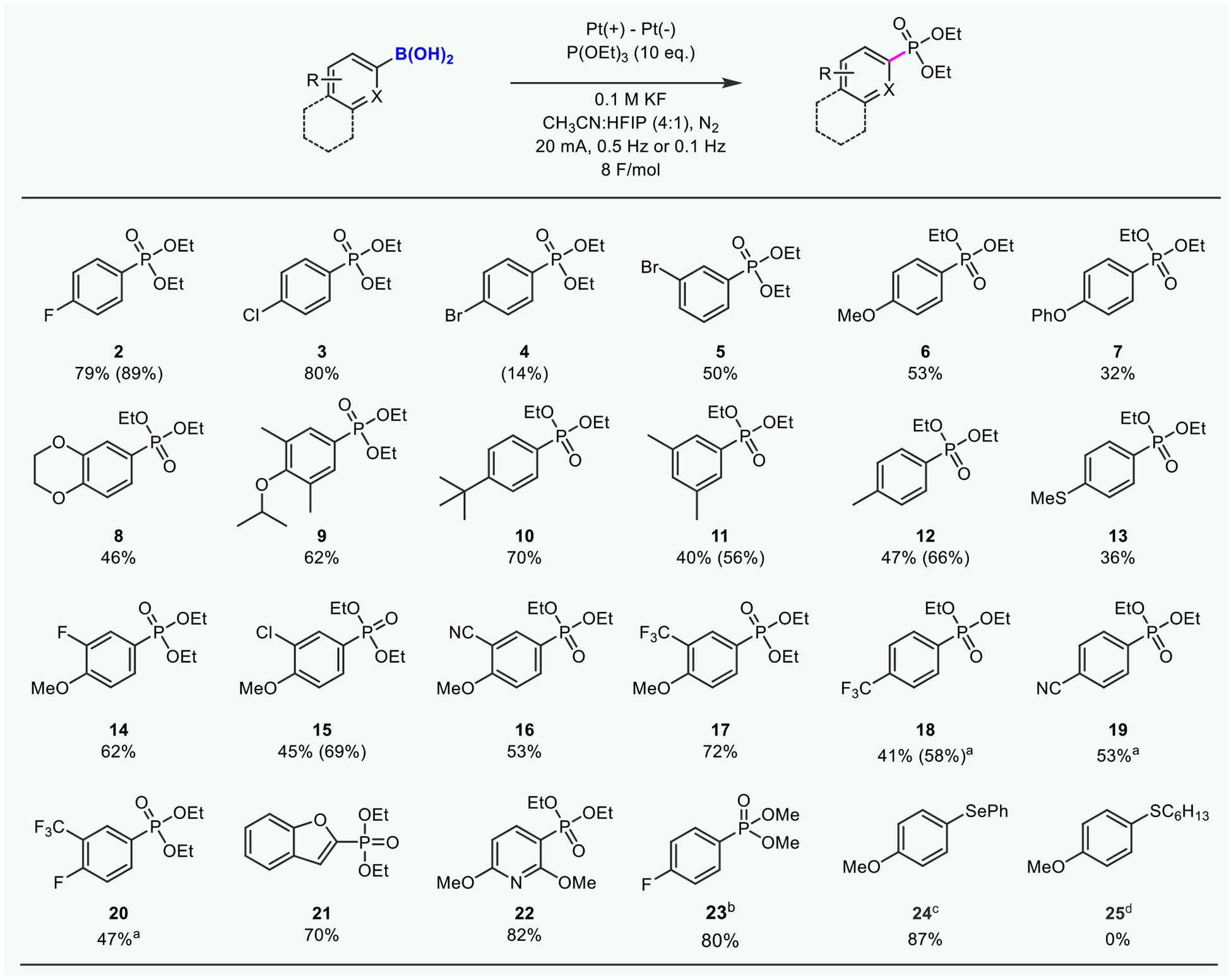
Scope of the electrochemical oxidative functionalization of aryl-B(OH)_2_ to form C─P bonds. Standard condition: Pt plates as anode and cathode, 0.14 mmol of aryl-B(OH)_2_ in MeCN/HFIP (4:1) solvent (4 mL in total), 0.1 M KF (2.9 equiv. to substrate), 10 equiv. of P(OEt)_3_, electrolysis at alternating current (20 mA, 0.5 Hz), 8 F mol^−1^, N_2_ environment. Yields are isolated percent yields. Values in parenthesis are yields determined by ^1^H NMR of the crude using 1,3,5-trimethoxybenzene as internal standard. ^a^Frequency of 0.5 Hz was used instead of 0.1 Hz. ^b^10 equiv. of P(OMe)_3_ as a radical trapping reagent. ^c^5 equiv. of (PhSe)_2_ as a radical trapping reagent. ^d^10 equiv. of (C_6_H_13_S)_2_ as a radical trapping reagent.

Next, the electrochemical method utilizing fluoride anionic activation and AP electrolysis was applied to a series of aryl boronic acids (Figure 3). The standard condition showed excellent tolerance on substrates bearing fluorine and chlorine (**2**, **3**) while 4-bromophenyl boronic acid remained problematic maybe due to poor radical stability when Br is on *para* position (**4**). When the bromine was at *meta* position, the reaction gave 50% yield (**5**). The electron-rich and neutral substrates afforded moderate yields (**6**–**12**). The reaction condition was also compatible with oxidatively sensitive functional group like thioether (**13**). Di-substituted electron-neutral phenyl boronic acids with halogen atoms also showed good compatibility (**14**–**17**). Strong electron-poor substrates (**18**–**20**) can be accessed by tuning the frequency from 0.5 Hz to 0.1 Hz, thus, increasing the working potential during AP electrolysis.^[40]^ Heteroaryl rings such as benzofuran and pyridine showed great reactivity and gave good yields (**21**, **22**). Other type of phosphite reagent, such as trimethyl phosphite was applied (**23**) and the yield was at 80%. Using triphenyl phosphite as a radical trap showed messy result and gave no desired product (**34**, Supporting information, Figure S12). Finally, other radical trapping reagents were tested. Using diphenyl diselenide as a trapping reagent (**24**) afforded high yield as 87%. Using sulfide as trapping reagent did not form desired product (**25**, see supporting information Figure S12 for other failed substrates).

Among the substrates examined, several electron-deficient aryl boronic acids (**2**, **18**–**20**) were of particular interest. Under the new condition, significant improvements in yield was obtained in substrate **2** compared with previously reported aryl trifluoroborates oxidation.^[25]^ In contrast, other electron-deficient substrates (**18**–**19**) showed similar reactivities with their trifluoroborates counterparts. These observations suggest the possibility of two distinct mechanistic pathways for different classes of aryl boronic acids. Hence, we further investigated the mechanism of KF activation and why the electron-poor aryl boronic acids could be accessed successfully.

**Figure 4.**
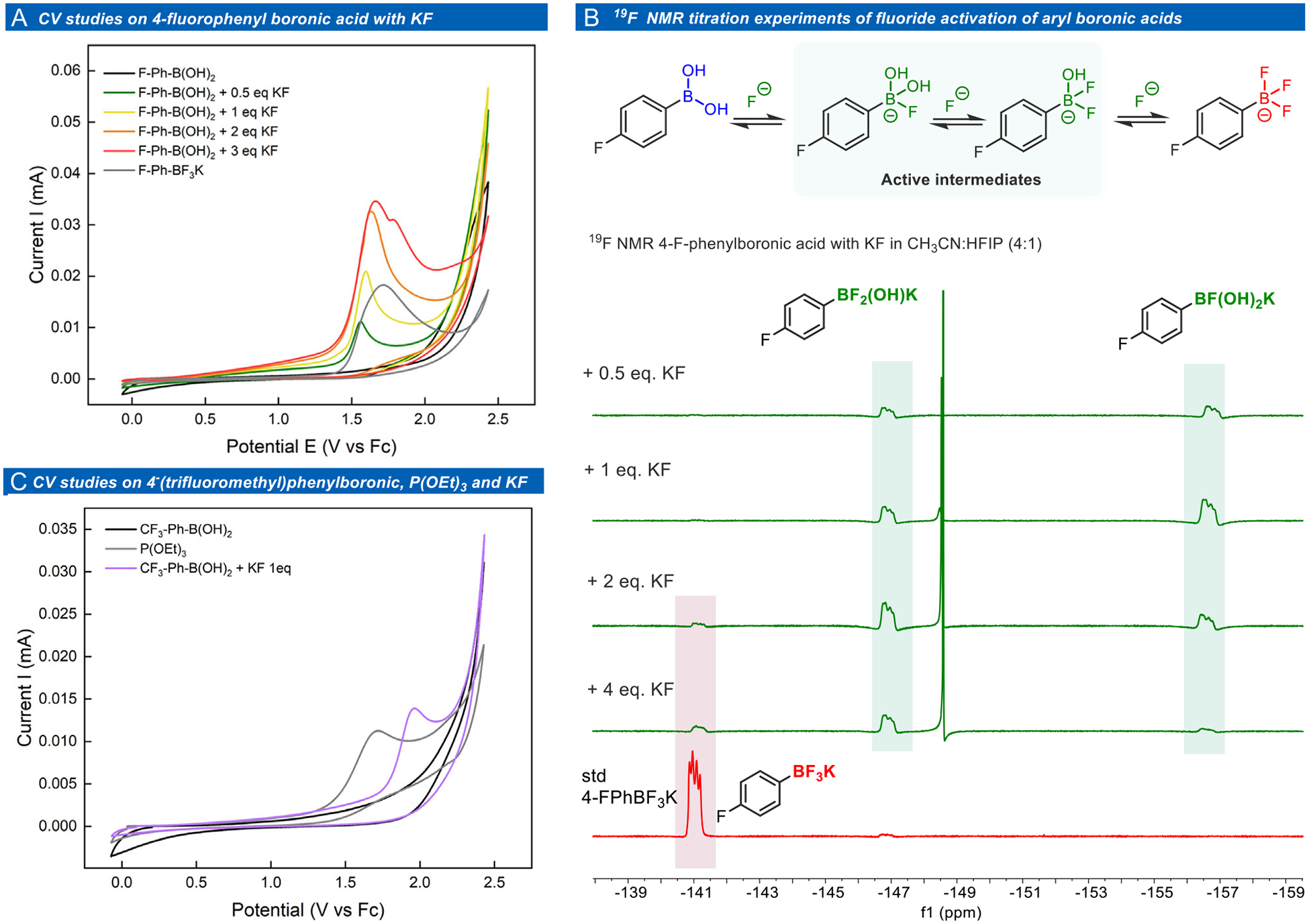
Mechanistic studies and proposed redox-active intermediates. A) CV studies and B) ^19^F NMR analysis showing fluoride activation of aryl boronic acids to form redox-active ArB(F)_*n*_(OH)_m_ intermediates. C) CV studies of fluoride activated 4-trifluorophenylboronic acid versus P(OEt)_3_.

To probe fluoride anionic activation, 4-fluorophenylboronic acid was treated with varying amounts of KF in the reaction solvent mixture (MeCN/HFIP = 4/1) and analyzed by cyclic voltammetry and ^19^F NMR spectroscopy (Figure 4A and B). In the absence of KF, the 4-fluorophenylboronic acid exhibited an oxidation potential of 2.20 V versus Fc/Fc^+^ and slow electron transfer. Addition of 0.5 equivalent of KF to this solution produced a new oxidation wave at 1.55 V versus Fc/Fc^+^, a 650 and 150 mV decrease in oxidation potential than boronic acid and 4-fluorophenyltrifluoroborate (*E*_ox_ = 1.70 V vs. Fc/Fc^+^), respectively, indicating a different and more easily oxidized organoboron intermediates. With more KF added, the intensity of oxidation wave grew larger, with the potential gradually shifting anodically until approaching that of potassium 4-fluorophenyltrifluoroborate (*E*_ox_ = 1.70 V vs. Fc/Fc^+^). The active boron intermediate in the equilibrium had 150 mV lower oxidation potential than 4-fluorophenyltrifluoroborate and triethyl phosphite (*E*_ox_ = 1.70 V vs. Fc/Fc^+^). Titration experiments using ^19^F NMR analysis provided another evidence that the active quaternary intermediates differ from corresponding perfluorinated Ar–BF_3_K salt (Figure 4B, Ar = 4-fluorophenyl). Upon addition of 0.5 equivalents of fluoride, two distinct fluorine signals appeared in the B─F bond chemical shift region. These intermediates were assigned as Ar–BF(OH)_2_ and Ar–BF_2_(OH) based on reported values and trends in the literature.^[44–46]^ At 4 equivalents of KF, Ar–BF_3_K started to form, however, the mono and bifluoroboronates remained as major species in solution. Same trends were also observed in the case of 4-(trifluoromethyl)phenylboronic acid under analogous studies (Figure S8, see Supporting Information for detail). During the bulk electrolysis, the active species in the solution were an equilibrium form of Ar–BF(OH)_2_ and Ar–BF_2_(OH). This decrease in oxidation potential enabled by fluoride activation demonstrated why the reaction conditions in this work could generate aryl radicals more effectively over our previous work even with electron-poor aryl boronic acids as substrates.

Next, we looked at the underlying mechanism behind aryl boronic acids with highly positive oxidation potentials. Using 4-(trifluoromethyl)phenylboronic acid as model substrate, we observed similar trends and patterns of KF titration in cyclic voltammetry (Figure S2, see Supporting Information for detail). In this case, the observed redox-active fluoroboronate intermediates (*E*_ox_ = 1.95 V vs. Fc/Fc^+^) have 100 and 350 mV lower oxidation potential than their Ar–BF_3_K and Ar–B(OH)_2_ (Ar = 4-trifluorophenyl) counterpart. Importantly, these intermediates have higher oxidation potential than triethyl phosphite (*E*_ox_ = 1.70 V vs. Fc/Fc^+^) (Figure 4C). These observations indicate that under direct oxidation conditions, triethyl phosphite undergoes oxidation to form P(OEt)_3_ radical cation, which is then trapped by the aryl boronates. If P(OEt)_3_ is the first species to be oxidized, alternating polarity would not be necessary to form products. A controlled experiment with direct current (0 Hz) was performed and 51% NMR yield was obtained (see Supporting Information Table S6), which correlates with our proposed mechanism. The slightly increased yield from alternating polarity is likely due to an optimal working potential to oxidize P(OEt)_3_ by slowly switching current.^[40,41]^

**Figure 5.**
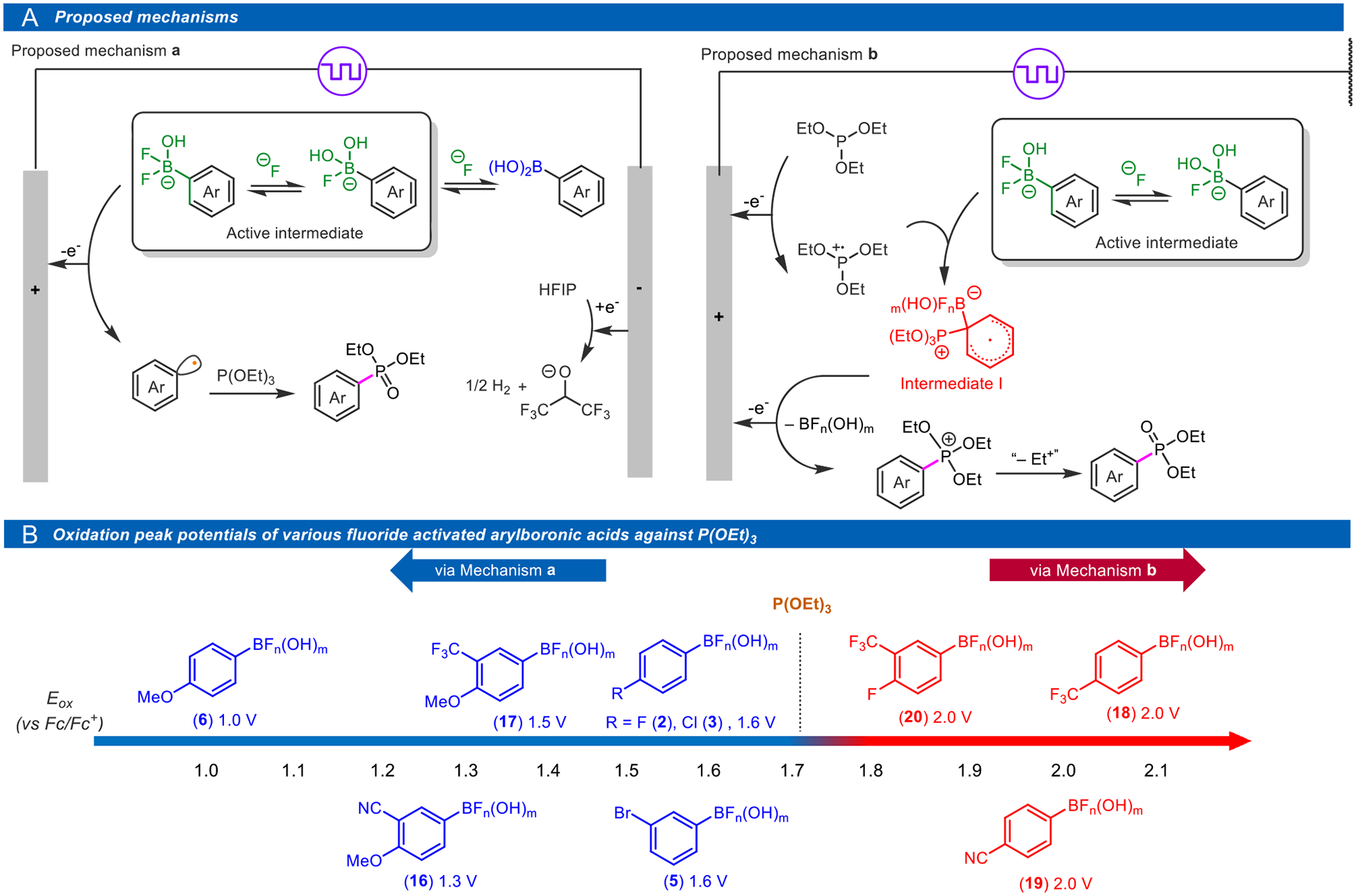
A) Proposed dual mechanisms a and b. B) Redox potentials for the oxidation of fluoride-activated arylboronic acids (selected examples) versus P(OEt)_3_ and indication of their most probable mechanism.

Based on the mechanistic studies, two general reaction pathways were proposed (Figure 5). For substrates of which fluoride-activated intermediates oxidize at lower potential than P(OEt)_3_, the reactions proceed via pathway **a**. Upon fluoride coordination to the boron center, the organoboron species becomes easier to oxidize. At the anode, these fluoride-activated organoboron intermediates undergo direct oxidation to generate aryl radicals, which are then trapped by P(OEt)_3_ to form the C─P bond. Meanwhile on the cathode, proton reduction from HFIP (onset *E*_red_ = −0.8 V vs. Fc/Fc^+^) occurs. The mild reductive potential offered by HFIP (vs. acetone reduction)^[25]^ provided a potential-gated electrolysis^[49]^ and protects aryl halides from undesired electroreduction. As for those activated aryl boronic acids which oxidize after P(OEt)_3_, the reaction mechanism is shown in pathway **b** as an *ipso*-substitution on aryl boronates.^[50–54]^ On the anode, P(OEt)_3_ was oxidized first to form phosphine radical cation.^[55,56]^ Subsequently, this radical cation added to aromatic ring, leading to intermediate **I**. Then, a second electron oxidation happens, followed by elimination to regenerate the aromatic ring. The proposed *ipso*-substitution process is highly likely to be the mechanism for electron-poor aryl BF_3_K substrates (E_ox_ > 1.7 V vs. Fc/Fc^+^) as well in our previous work.^[25]^ As such, we evaluated a set of substrates and obtained the oxidation potentials of their active fluoroborate intermediates. Most fluorinated intermediates of our substrates have lower oxidation potential than 1.7 V vs. Fc/Fc^+^, which undergo aryl radical mechanism (mechanism **a**). Electron-poor compounds **18**–**20** have higher oxidation potential than P(OEt)_3_, leading to the *ipso*-substitution mechanism **b** (Figure 5B).

## 3. Conclusion

In conclusion, we have developed a new electrochemical strategy to generate aryl radicals from aryl boronic acids to form aryl C─P bonds with broad scope encompassing electron rich and deficient aryl boronic acids. This strategy was enabled by the integration of alternating polarity electrolysis and in situ fluoride activation to generate fluoroborates with lower oxidation potentials than their corresponding trifluoroborate salts and boronic acids. Moreover, we uncover a previously unrecognized deborylative reaction between phosphite radical cation with electron-deficient aryl boronic acids, expanding the scope and mechanistic understanding of electrochemical aryl boronic acid cross-coupling reactions.

## Supplementary Material

Supporting Information

The authors have cited additional references within the Supporting Information.^[57–66]^

Supporting information for this article is available on the WWW under https://doi.org/10.1002/celc.202500363

## Data Availability

The data that support the findings of this study are available in the supplementary material of this article.
