# Supplementary material for: Electrochemical Oxidation of Aryl Boronic Acids via Fluoride Activation and Alternating Polarity Electrolysis for Aryl C–P Bond Formation
Source: ChemElectroChem. Author manuscript; Available in PMC 2026 Jul 11. (PMC13354047; doi:10.1002/celc.202500363)
Supplement: Supporting Information [file NIHMS2183584-supplement-Supporting_Information.pdf]

Supporting Information for

Electrochemical Oxidation of Aryl Boronic Acids via Fluoride Activation and Alternating Polarity Electrolysis for Aryl C–P Bond Formation

Enqi Feng, Ian Vanswearingen, Maxime Boudjelel, Lise Fabre, Rossul Aldhufarri, and Christian A. Malapit*

Department of Chemistry, Northwestern University, Evanston, Illinois 60208, United States

Email: christian.malapit@northwestern.edu

Contents

1. **General Information** S3
2. **Experimental procedure** S3
3. **Additional Information for Condition Optimization** S4
4. **Cyclic Voltammetry Study** S7
5. **NMR titration** S13
6. **Compound Characterization** S14
7. **NMR spectra** S34
8. **Reference** S111

1. General Information

All experiments were conducted using standard Schlenk lines techniques under a nitrogen atmosphere. Acetonitrile, HFIP were used as ACS grade without further drying and deoxygenated by nitrogen purge. Aryl boronic acids were purchased from Ambeed or Sigma-Aldrich and used as received. Triethylphosphite and potassium fluoride were purchased from Sigma-Aldrich. Deuterated solvents were purchased from Cambridge Isotope Laboratories Inc. Cyclic voltammetry were recorded using Biologic SP–50e. Electrosynthesis were performed using IKA ElectraSyn 2.0 using platinum plate electrodes purchased from Surepure Chemetals. Column chromatography was performed on Biotage flash chromatography. NMR spectra were recorded on Bruker Avance III HD 500 MHz spectrometer. ^{1}H and ^{13}C chemical shifts are reported in ppm relative to tetramethylsilane using residual solvent as an internal standard. ^{19}F and ^{31}P chemical shifts are reported in ppm without internal standard.

2. Experimental procedure

Procedure for general aryl boronic acid with KF

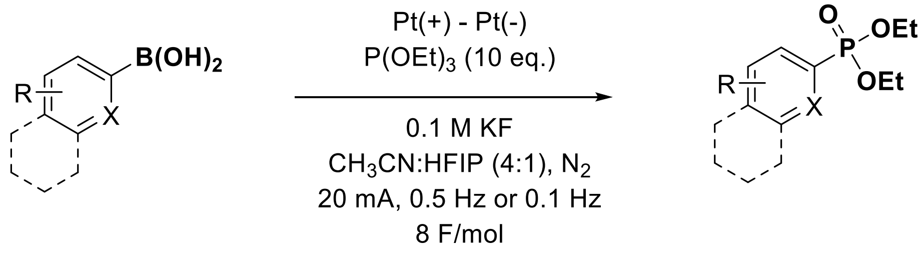

A 4 mL Electrasyn vial was charged with boronic acid (0.14 mmol, 1 eq.), potassium fluoride (23 mg, 0.4 mmol, 2.9 eq.) and triethyl phosphite (0.24 mL, 1.4 mmol, 10 eq.). A stir bar was added and the threads were wrapped with parafilm to ensure an airtight seal when cap added. 4 mL of a 0.1 M KF solution in (4:1) MeCN (3.2 mL): HFIP (0.8 mL). An electrasyn cap was prepared with 2 platinum plates (10 mm x 10 mm x 0.1 mm) and a rubber septa was used to cover the top cap (Figure S1). The cap was then twisted onto the vial, ensuring the electrodes aren't touching and are submerged in the solution. The solution was then purged with nitrogen for 5 minutes then was replaced with a N_2 balloon. This vial was equipped to an Electrasyn and the following conditions (20 mA, 0.5 Hz or 0.1 Hz, 8 F/mol). Once the reaction was finished, 8.4 mg of 1,3,5-trimethoxybenzene was added as NMR internal standard. The crude solution was diluted with 25 mL ethyl acetate and washed with 10 mL saturated $NaHCO_3$ and 10 mL brine. The organic layer was collected and dried by $MgSO_4$. The organic phase was then concentrated under vacuum and the yield was determined by ^{1}H NMR. The crude product was purified by passing through a silica gel column (EA/hexane 10% - 60%). The product collected after column was then dried on vacuum line overnight to afford yellow oil.

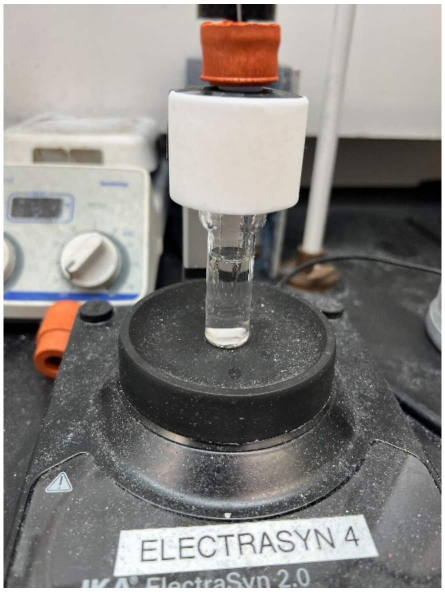

Figure S1. Electrolysis setup

3. Additional Information for Condition Optimization

Table S1. Selection of activators

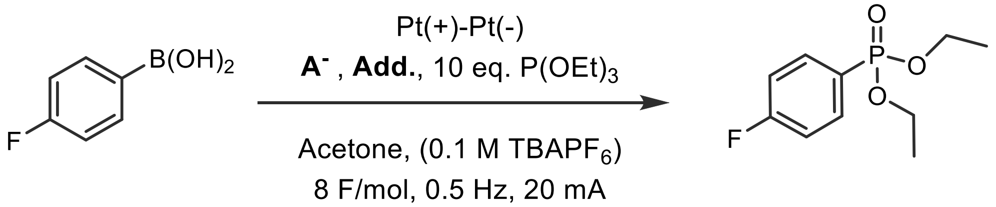

Activator (3 eq)	Additive (3 eq)	Yield
TBAOH	N/A	0%
KOH	H_2O	0%
NaOMe	MeOH	0%
NaOEt	15C5	0%
CsF	18C6	9%
CsF	PEG 300	13%

KF	18C6	19%
TMAF	None	21%

Table S2. Selection of phase transfer reagents and solvents

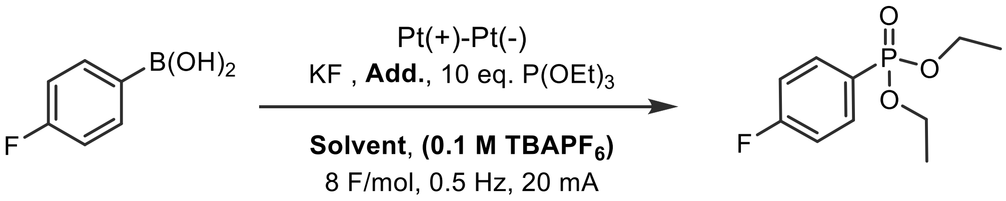

Additive	Solvent	Electrolyte	Yield
3 eq. PEG 300	Acetone	$TBAPF_6$	15%
0.3 eq. 18C6	Acetone	$TBAPF_6$	19%
1 eq. 18C6	Acetone	$TBAPF_6$	47%
HFIP	Acetone	$TBAPF_6$	40%
1 eq. 18C6	Acetonitrile	$TBAPF_6$	48%
HFIP	Acetonitrile	$TBAPF_6$	59%
1 eq. 18C6	Acetonitrile	None	82%
HFIP	Acetonitrile	None	89%

Table S3. Selection of electrolysis charges

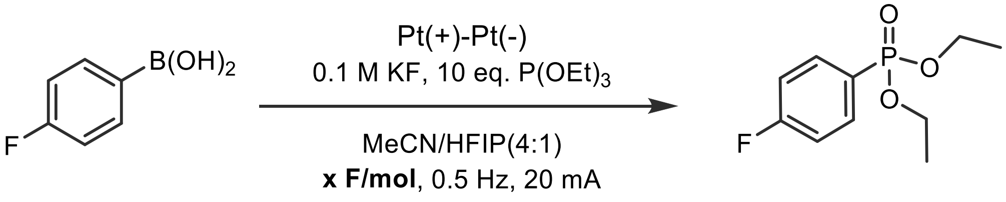

Charges (F/mol)	Yield
0	0%
4	28%

8	89%

Table S4. Selection of electrolysis current

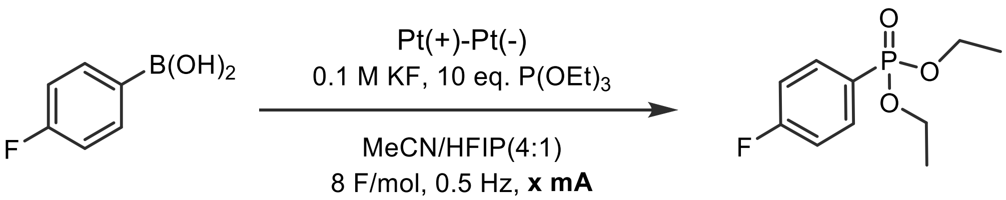

Current (mA)	Yield
0	0%
10	88%
20	89%
30	86%
40	85%
60	86%

Table S5. Selection of the amount of P(OEt)3 under different mechanisms

For mechanism **a**

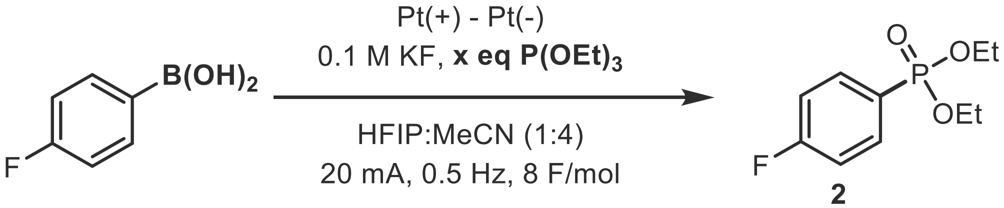

2

$P(OEt)_3$ (eq.)	Yield
1	16%
5	84%
10	89%

For mechanism **b**

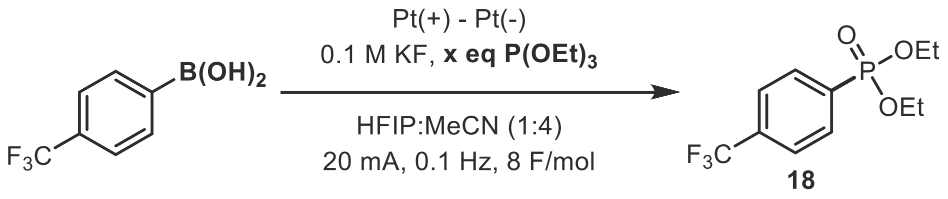

$P(OEt)_3$ (eq.)	Yield
2	38%
5	53%
10	58%

Table S6. Frequency studies on electron-poor substrate 18

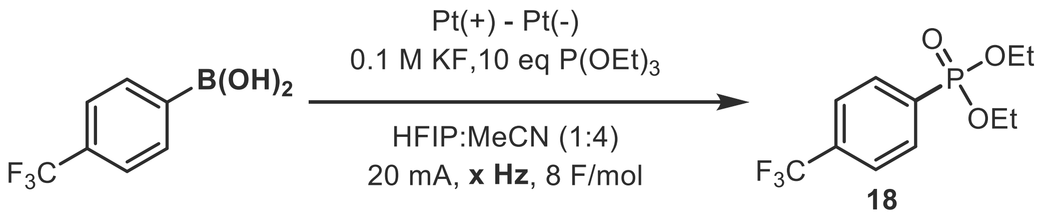

Frequency (Hz)	Yield
0	51%
0.1	58%
0.5	32%

4. Cyclic Voltammetry Study

Cyclic voltammetry experiments were performed on a 5 mM solution of the substrate in a 4:1 mixture of acetonitrile and hexafluoro-2-propanol (total volume: 10 mL), using tetrabutylammonium hexafluorophosphate (0.1 M) as the supporting electrolyte.

A platinum working electrode (2mm diameter, CHI102), a carbon graphite counter electrode, and a silver/silver nitrate reference electrode were employed. All measurements were conducted within a potential window of 0–2.5 V. A scan rate of 100mV/s was used.

Ferrocene was used to reference the electrochemical potentials.

Potassium fluoride was added in varying equivalents: 0.5 eq (+1.5 mg), 1.0 eq (+1.5 mg), 2.0 eq (+3.0 mg), and 3.0 eq (+3.0 mg), relative to the substrate.

Subsequent cyclic voltammetry experiments were carried out under identical conditions using the potassium trifluoroborate analogue of the substrate.

Figure S2: Cyclic voltammogram of 4-(trifluoromethyl)phenylboronic acid with incremental potassium fluoride addition and of Potassium 4-(trifluoromethyl)phenyltrifluoroborate

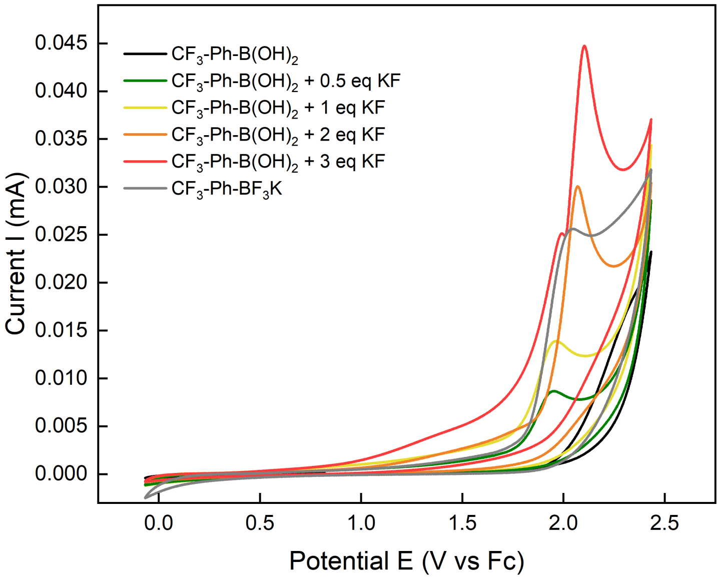

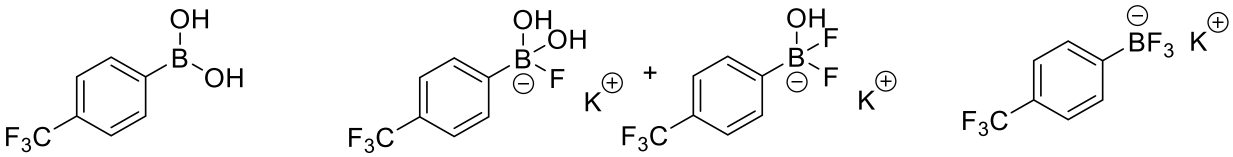

E ox = 2.30 V E ox = 1.95 V E ox = 2.04 V

A noticeable shift in the oxidation potential is observed between the boronic acid (2.30 V) and the boronic acid with 0.5 equivalent of KF (1.95 V), consistent with the formation of fluoride-bound intermediates. This intermediate behavior is compared to the cyclic voltammogram of the corresponding potassium trifluoroborate (2.04 V).

Figure S3: Cyclic voltammogram of 4-(cyanophenyl)boronic acid with incremental potassium fluoride addition and of Potassium 4-(cyano)phenyltrifluoroborate

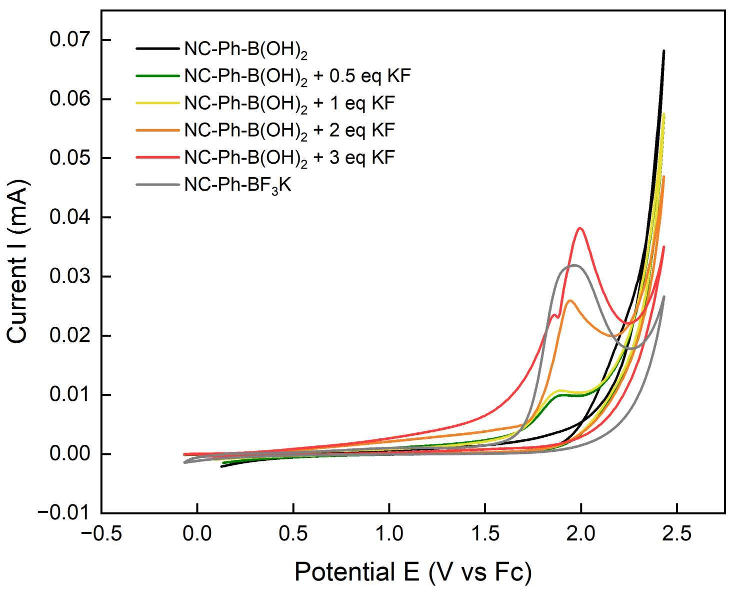

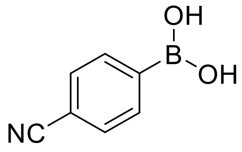

$E_{ox} = 2.15$ V

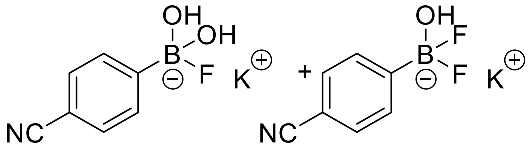

$E_{ox} = 1.88$ V

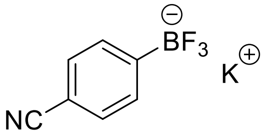

$E_{ox} = 1.96$ V

A noticeable shift in the oxidation potential is observed between the boronic acid (2.15 V) and the boronic acid with 0.5 equivalent of KF (1.88 V), consistent with the formation of fluoride-bound intermediates. This intermediate behavior is compared to the cyclic voltammogram of the corresponding potassium trifluoroborate (1.96 V).

Figure S4: Cyclic voltammogram of 4-(fluorophenyl)boronic acid with incremental potassium fluoride addition and of Potassium 4-(fluoro)phenyltrifluoroborate

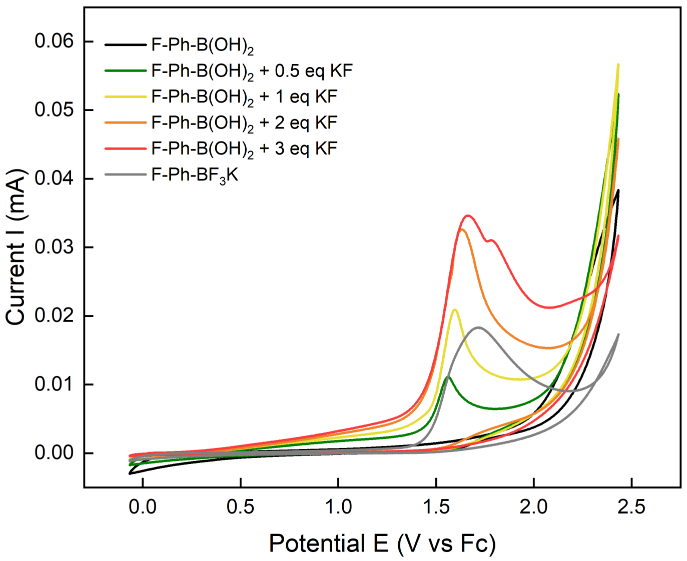

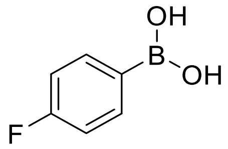

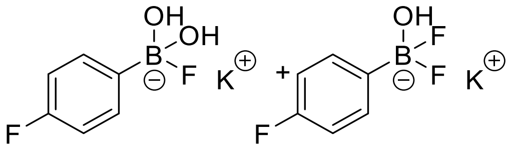

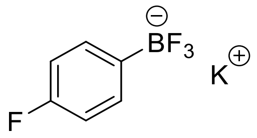

$E_{ox} = 2.20$ V $E_{ox} = 1.55$ V $E_{ox} = 1.70$ V

A noticeable shift in the oxidation potential is observed between the boronic acid (2.20 V) and the boronic acid with 0.5 equivalent of KF (1.55 V), consistent with the formation of fluoride-bound intermediates. This intermediate behavior is compared to the cyclic voltammogram of the corresponding potassium trifluoroborate (1.70 V).

Figure S5: Cyclic voltammogram of 4-(methoxyphenyl)boronic acid with incremental potassium fluoride addition and of Potassium 4-(methoxy)phenyltrifluoroborate

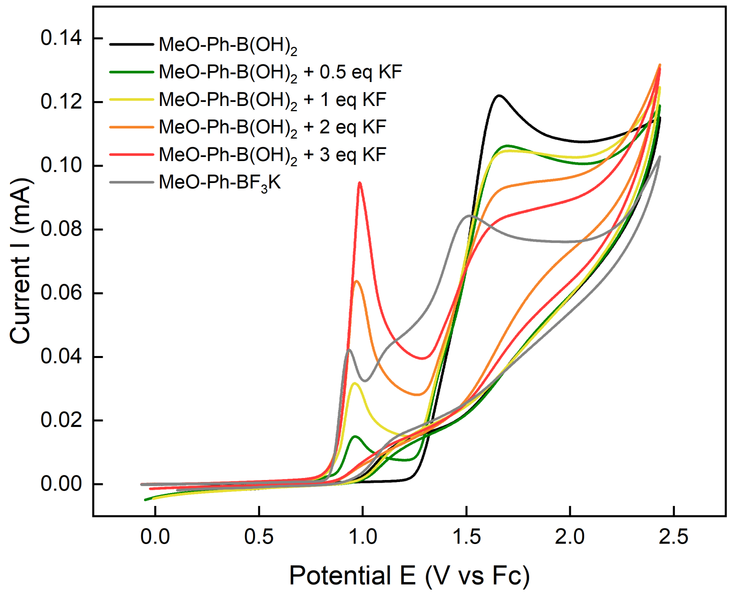

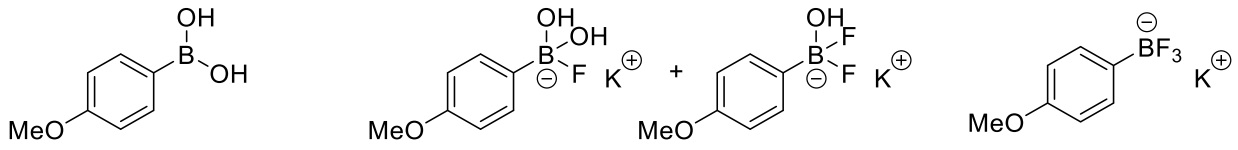

E ox = 1.18 V | E ox = 0.96 V | E ox = 0.94 V

A noticeable shift in the oxidation potential is observed between the boronic acid (1.18 V) and the boronic acid with 0.5 equivalent of KF (0.96 V), consistent with the formation of fluoride-bound intermediates. This intermediate behavior is compared to the cyclic voltammogram of the corresponding potassium trifluoroborate (0.94 V).

Control cyclic voltammetry experiments were carried out to verify the mechanism under the same conditions. Measurements were performed on a 5 mM solution of the substrate in a 4:1 mixture of acetonitrile and hexafluoro-2-propanol (total volume: 10 mL), using tetrabutylammonium hexafluorophosphate (0.1 M) as the supporting electrolyte.

A platinum working electrode (2mm diameter, CHI102), a carbon graphite counter electrode, and a silver/silver nitrate reference electrode were employed. All measurements were conducted within a potential window of 0–2.5 V. A scan rate of 100mV/s was used.

Ferrocene was used to reference the electrochemical potentials.

Figure S6: Cyclic voltammogram of triethyl phosphite with incremental 4-(trifluoromethyl)phenylboronic acid addition

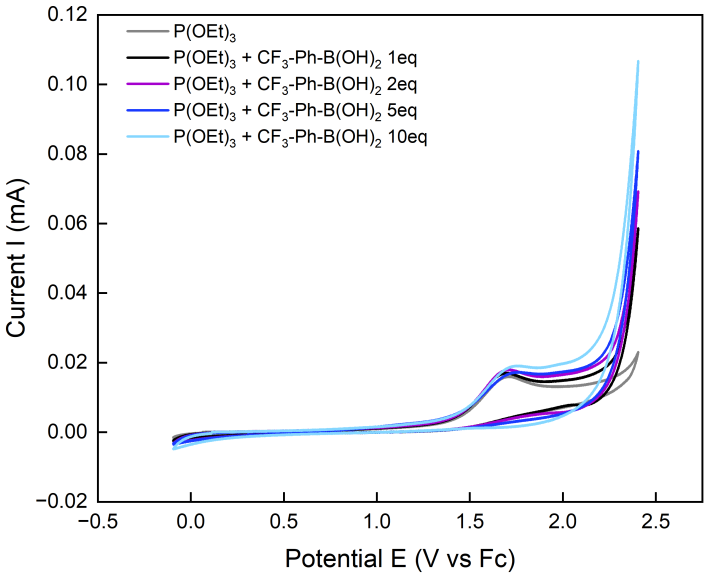

Figure S7: Cyclic voltammogram of activated intermediates against $P(OEt)_3$

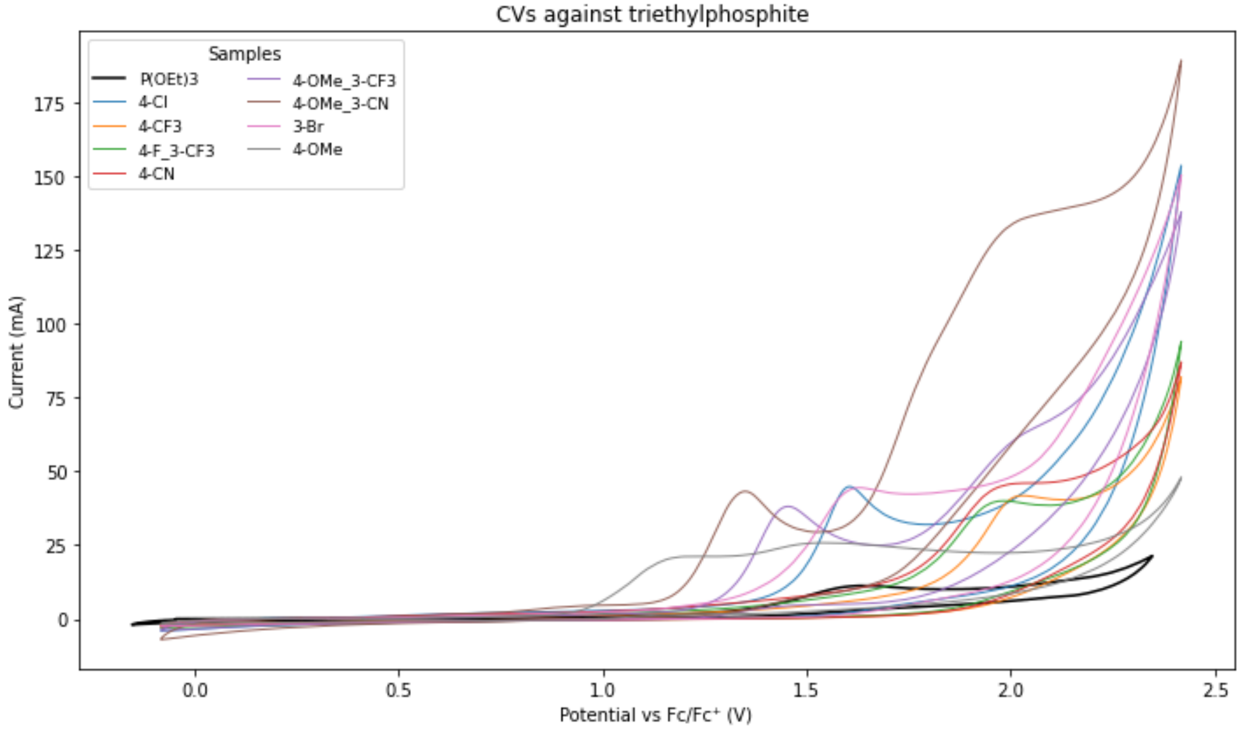

5. NMR titration

Figure S8: NMR titration on 4-(trifluoromethyl)phenylboronic acid with incremental addition of KF

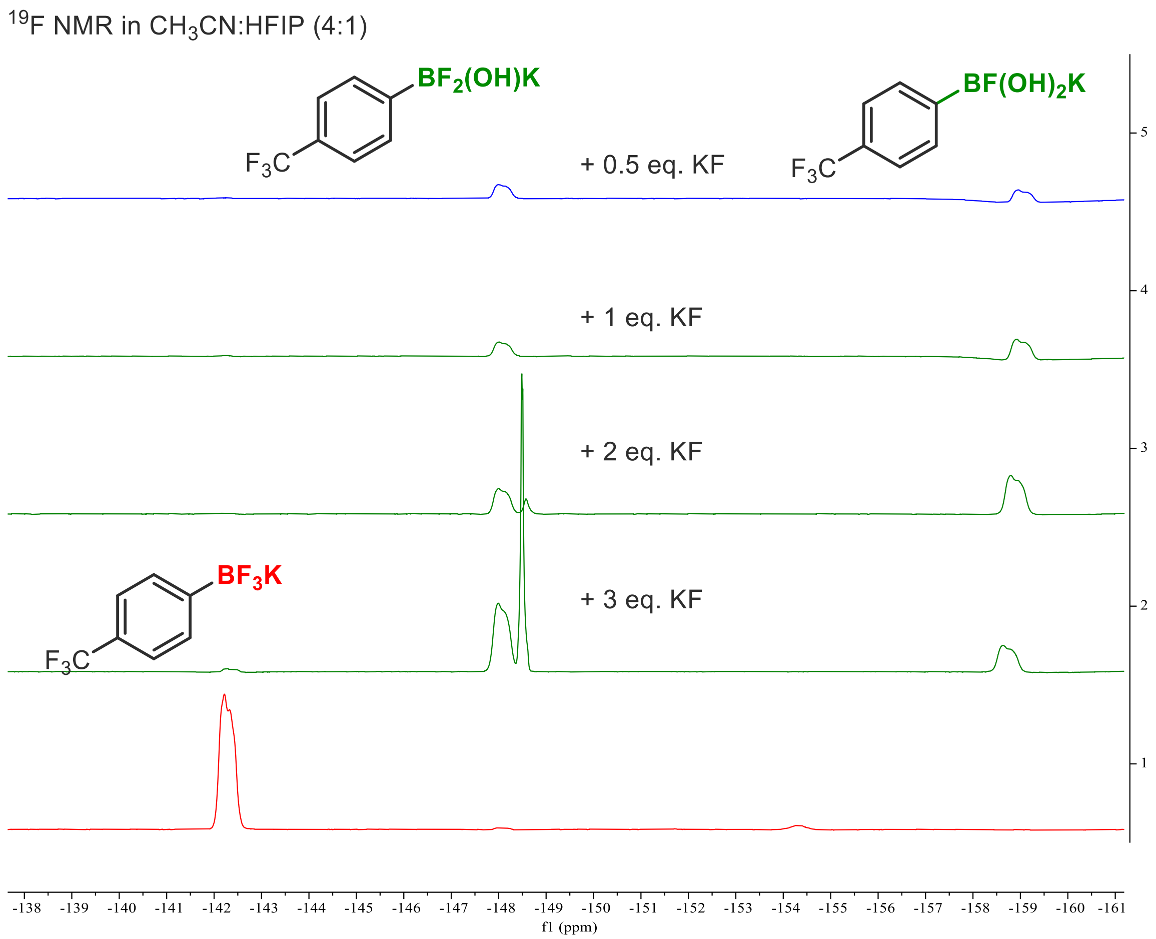

Five vials were prepared for the titration experiment.
To the first four vials, 0.05 mmol (9.5 mg) of trifluoromethyl phenylboronic acid was added. Potassium fluoride (KF) was then added in increasing equivalents as follows:
Vial 1: 0.5 equivalent of KF (+ 1.45 mg)
Vial 2: 1 equivalent of KF (+ 2.9 mg)
Vial 3: 2 equivalents of KF (+ 5.8 mg)
Vial 4: 3 equivalents of KF (+ 8.7 mg)
Each vial was then supplemented with 1 mL of a 4:1 mixture of acetonitrile (MeCN) and hexafluoroisopropanol (HFIP).
Vial 5 contained a 0.05 mmol (12.6 mg) solution of potassium trifluoromethylphenylborate in the same MeCN:HFIP (4:1) solvent mixture and served as a reference sample.

The resulting mixtures were transferred to clean and dry NMR tubes for ^{19}F NMR analysis.

6. Radical trapping experiments

To prove the mechanism **a** where aryl radicals were generated by electrolysis, 4-methoxyphenyl boronic acid and 4-fluorophenyl boronic acid were used to react with 10 equivalents of 1,1-diphenylethylene as a radical trapping reagent.

Figure S9: GC/MS Chromatogram of crude mixture of 4-methoxyphenyl boronic acid.

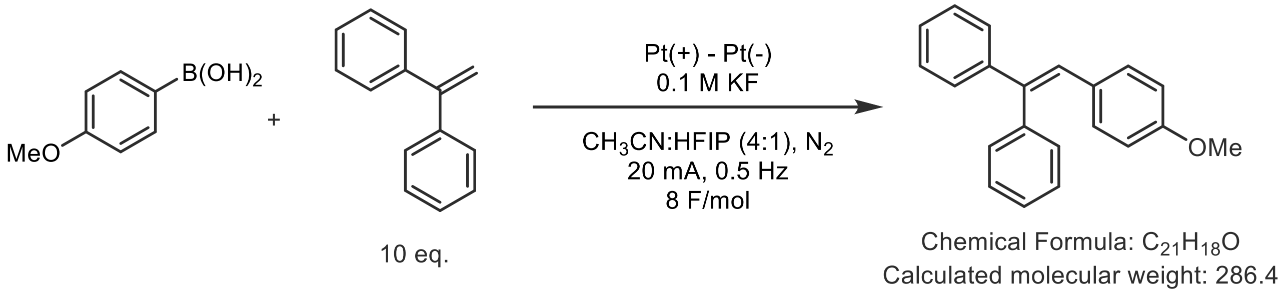

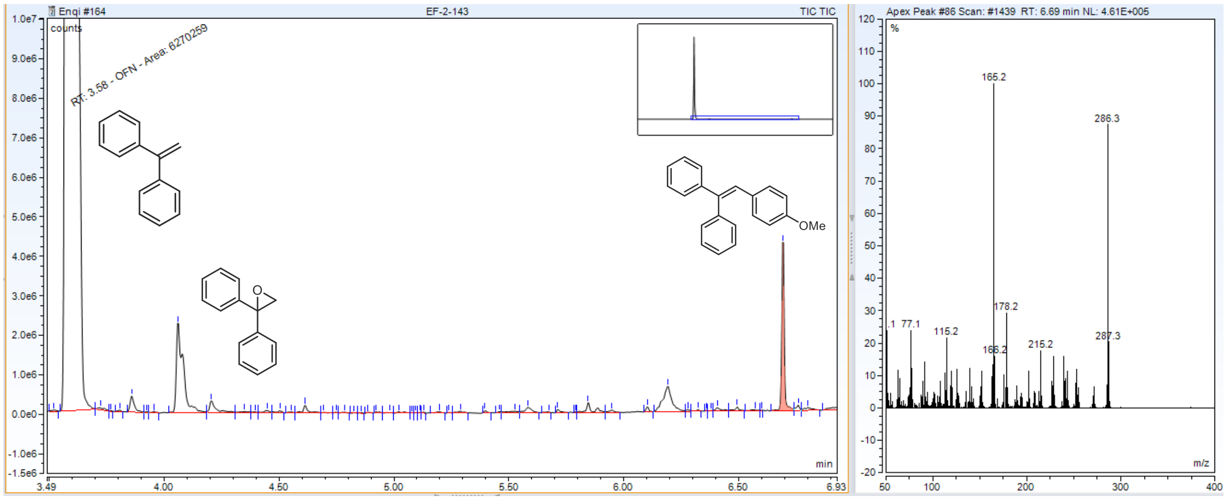

Figure S10: GC/MS Chromatogram of crude mixture of 4-fluorophenyl boronic acid.

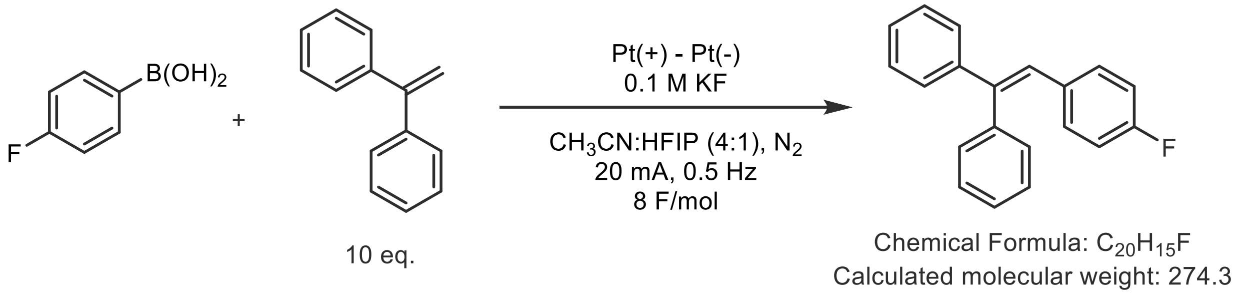

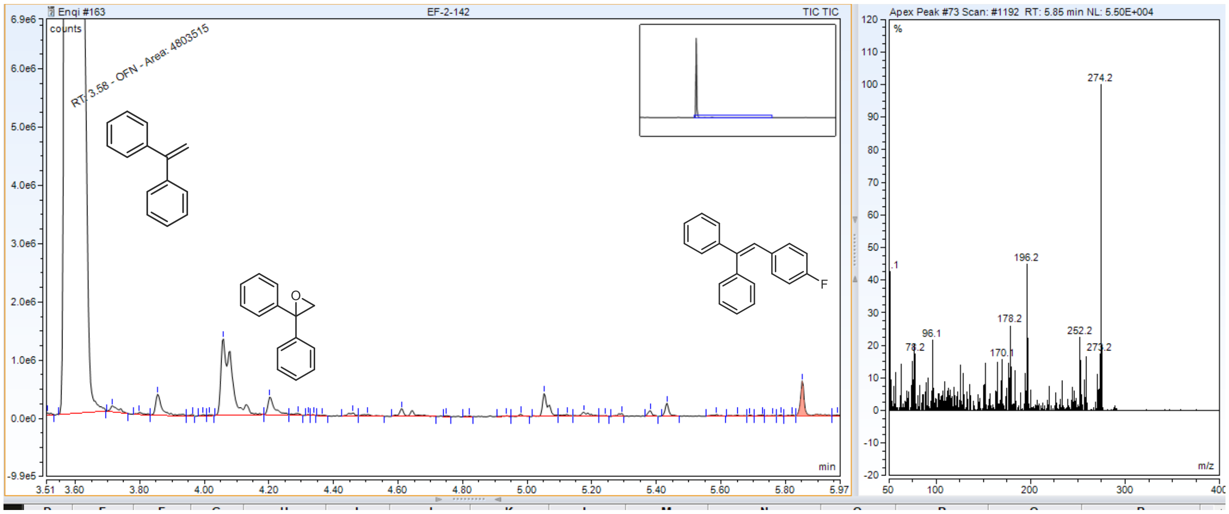

To prove the ipso-substitution happened through phosphine radical cation, we have used 20 equivalents of benzene to trap the intermediate. $TBAPF_6$ was chosen as supporting electrolyte. Reaction solvent was kept the same. The current and frequency were chosen as the standard condition when electrolyzing electron poor substrates **18-20**. The reaction crude NMR yield was 66% using 1,3,5-trimethoxy benzene as internal standard. The crude ^{31}P NMR also proved the existence of desired phosphorylation product at 19.55 ppm. The chemical shift and mechanism matched the literature report. (*Nat. Commun.* **2021**, *12*, 6629.). This trapping experiment demonstrates that the phosphine radical cation has been generated in our reaction system and underdoes *ipso*-substitution on the bare benzene ring.

Figure S11: Crude ^{1}H and ^{31}P NMR for trapping $P(OEt)_3$ with benzene.

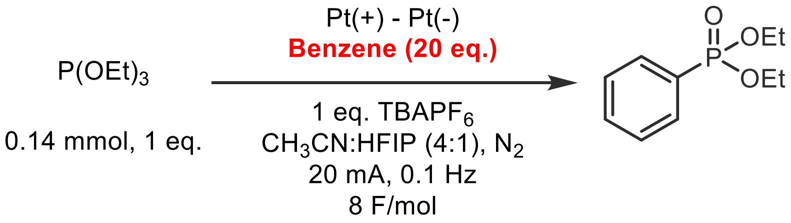

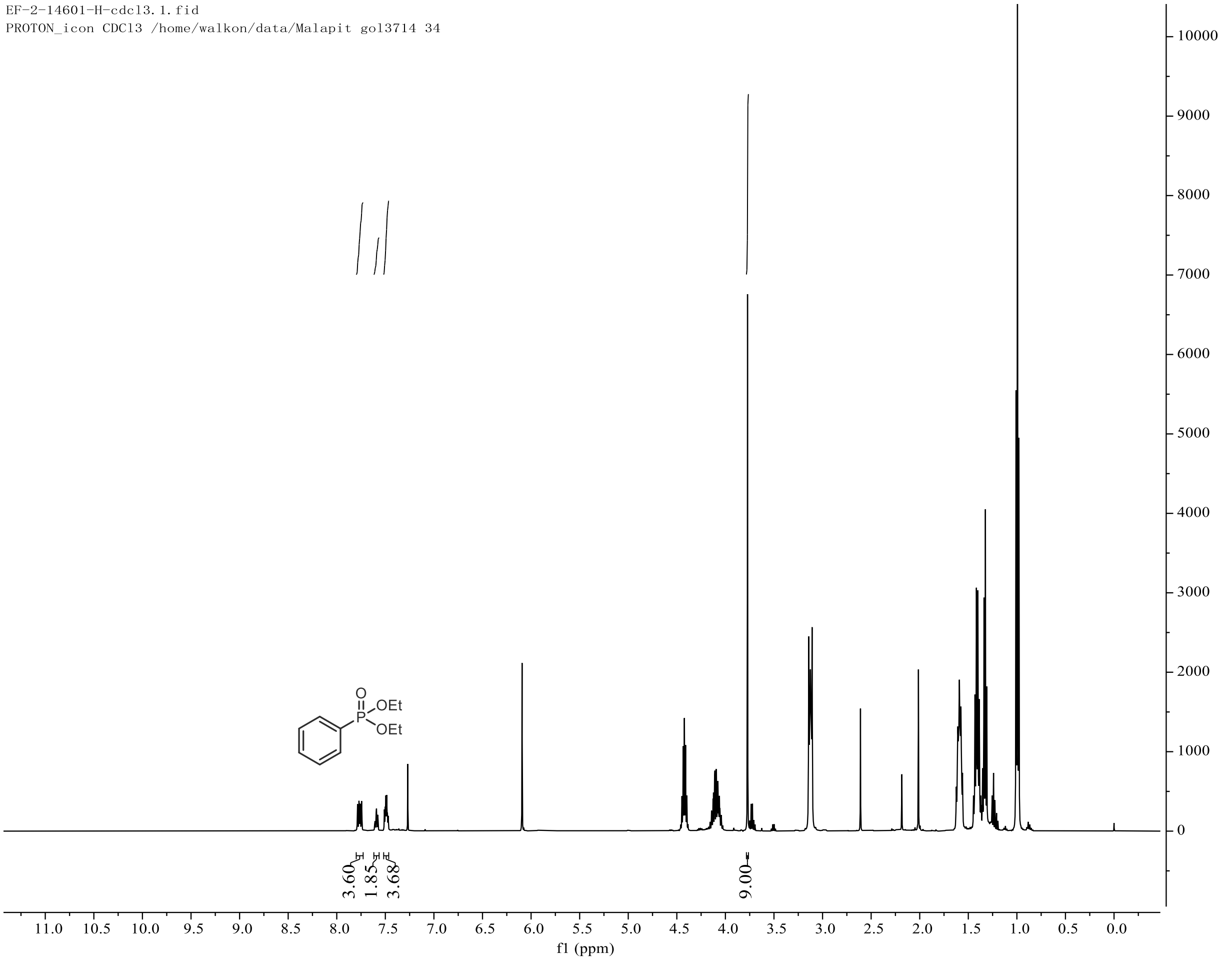
EF-2-14601-H-cdcl3.1.fid
PROTON_icon CDCl3 /home/walkon/data/Malapit gol3714 34
OEt
OEt
3.60
1.85
3.68
9.00
f1 (ppm)

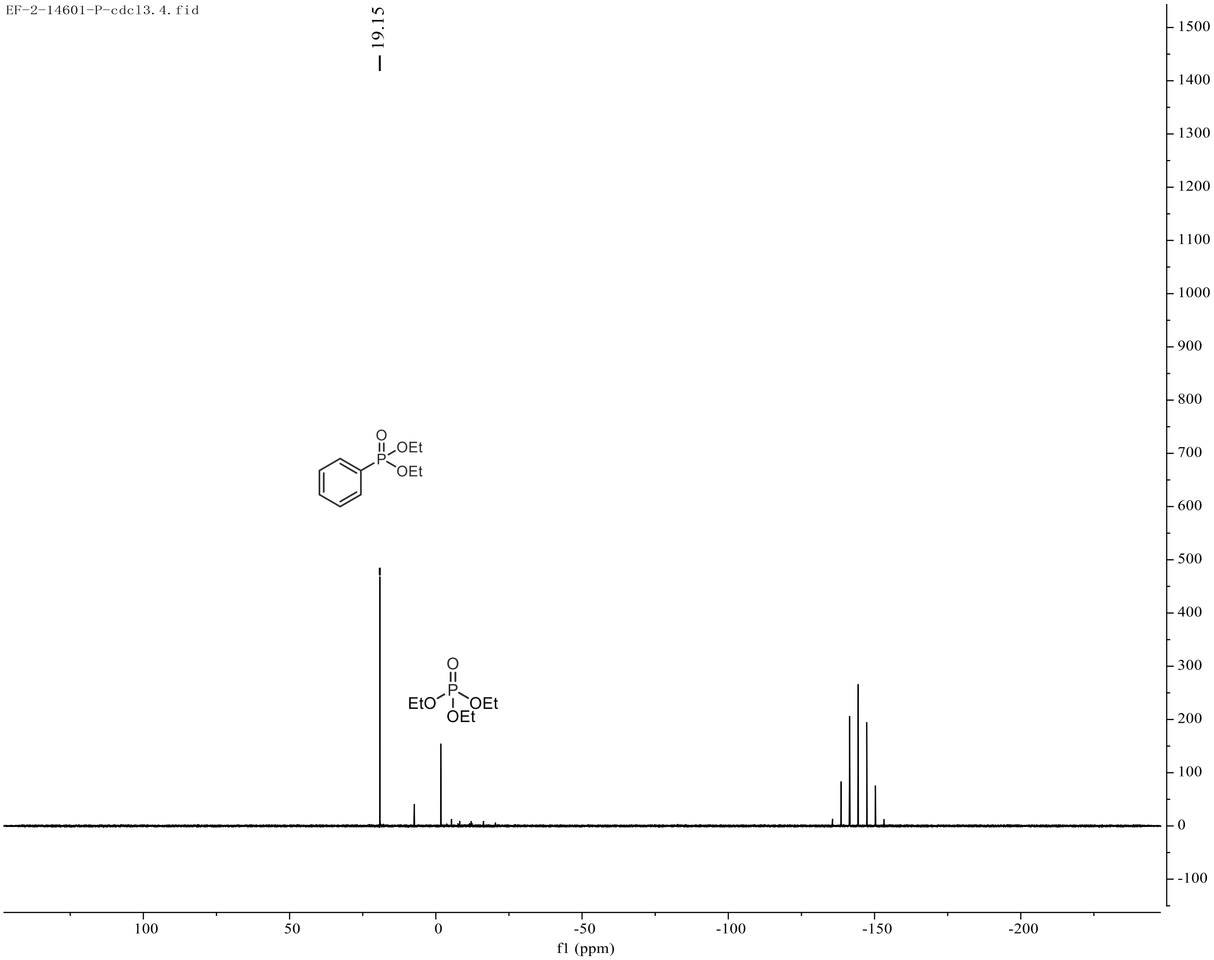

EF-2-14601-P-cdcl3.4.fid
19.15
OEt
OEt
O
P
EtO
OEt
OEt
f1 (ppm)

7. Failed substrate scope

Figure S12: Failed substrate scope

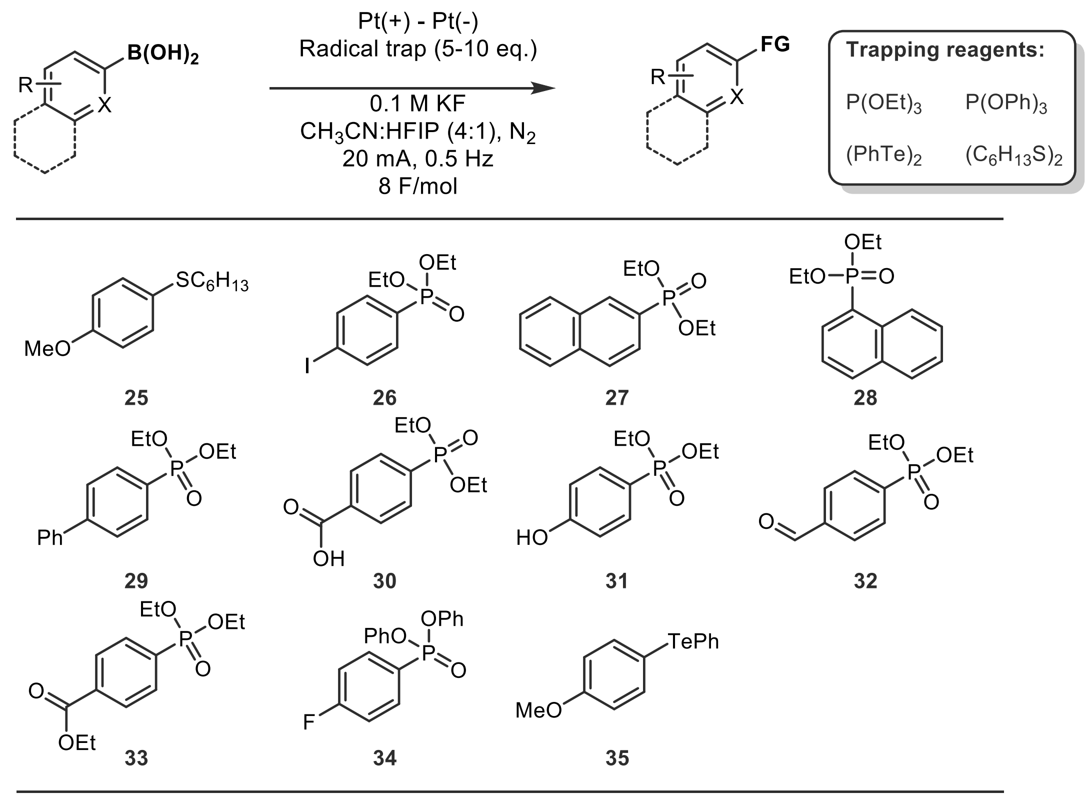

Yields were determined by ^{1}H NMR with 1,3,5-trimethoxybenzene as internal standard.

8. Compound Characterization

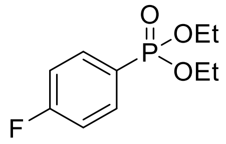

2

A 4 mL Electrasyn vial was charged with (4-fluorophenyl)boronic acid (19.6 mg, 0.14 mmol, 1 eq.), potassium fluoride (23 mg, 0.4 mmol, 2.9 eq.) and triethyl phosphite (0.24 mL, 1.4 mmol, 10 eq.). A stir bar was added and the threads were wrapped with parafilm to ensure an airtight seal when cap added. 4 mL of a 0.1 M KF solution in (4:1) MeCN (3.2 mL): HFIP (0.8 mL). An electrasyn cap was prepared with 2 platinum plates (10 mm x 10 mm x 0.1 mm) and a rubber septa was used to cover the top cap (Figure S1). The cap was then twisted onto the vial, ensuring the electrodes aren't touching and are submerged in the solution. The solution was then purged with nitrogen for 5 minutes then was replaced with a N_2 balloon. This vial was equipped to an Electrasyn and the following conditions (20 mA, 0.5 Hz, 8 F/mol). Once the reaction was finished, 8.4 mg of 1,3,5-trimethoxybenzene was added as NMR internal standard. The crude solution was diluted with 25 mL ethyl acetate and washed with 10 mL saturated $NaHCO_3$ and 10 mL brine. The organic

layer was collected and dried by $MgSO_4$. The organic phase was then concentrated under vacuum and the crude yield was determined by ^{1}H NMR (89% yield). The crude product was purified by passing through a silica gel column (EA/hexane 10% - 60%). The product collected after column was then dried on vacuum line overnight to afford yellow oil (79% yield). The NMR data matches literature.[1]

Diethyl (4-fluorophenyl)phosphonate (2). ^{1}H NMR (500 MHz, $CDCl_3$) δ 7.82 (ddd, *J* = 12.8, 8.6, 5.8 Hz, 2H), 7.15 (td, *J* = 8.7, 3.1 Hz, 2H), 4.19 – 4.03 (m, 4H), 1.33 (t, *J* = 7.1 Hz, 6H). ^{31}P NMR (202 MHz, $CDCl_3$) δ 18.03 – 17.58 (m). ^{19}F NMR (470 MHz, $CDCl_3$) δ -105.98 – -106.09 (m, 1F). ^{13}C NMR (126 MHz, $CDCl_3$) δ 165.5 (dd, *J* = 253.5, 3.9 Hz), 134.5 (dd, *J* = 11.3, 8.8 Hz), 124.7 (dd, *J* = 192.8, 3.4 Hz), 116.0 (dd, *J* = 21.5, 16.3 Hz), 62.4 (d, *J* = 5.4 Hz), 16.5 (d, *J* = 6.5 Hz).

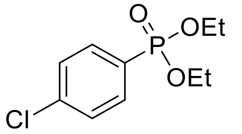

3

A 4 mL Electrasyn vial was charged with (4-chlorophenyl)boronic acid (21.9 mg, 0.14 mmol, 1 eq.), potassium fluoride (23 mg, 0.4 mmol, 2.9 eq.) and triethyl phosphite (0.24 mL, 1.4 mmol, 10 eq.). A stir bar was added and the threads were wrapped with parafilm to ensure an airtight seal when cap added. 4 mL of a 0.1 M KF solution in (4:1) MeCN (3.2 mL): HFIP (0.8 mL). An electrasyn cap was prepared with 2 platinum plates (10 mm x 10 mm x 0.1 mm) and a rubber septa was used to cover the top cap (Figure S1). The cap was then twisted onto the vial, ensuring the electrodes aren't touching and are submerged in the solution. The solution was then purged with nitrogen for 5 minutes then was replaced with a N_2 balloon. This vial was equipped to an Electrasyn and the following conditions (20 mA, 0.5 Hz, 8 F/mol). Once the reaction was finished, 8.4 mg of 1,3,5-trimethoxybenzene was added as NMR internal standard. The crude solution was diluted with 25 mL ethyl acetate and washed with 10 mL saturated $NaHCO_3$ and 10 mL brine. The organic layer was collected and dried by $MgSO_4$. The organic phase was then concentrated under vacuum. The crude product was purified by passing through a silica gel column (EA/hexane 10% - 60%). The product collected after column was then dried on vacuum line overnight to afford yellow oil (80% yield). The NMR data matches literature.[1]

Diethyl (4-chlorophenyl)phosphonate (3). ^{1}H NMR (500 MHz, $CDCl_3$) δ 7.79 – 7.71 (dd, *J* = 13.0, 8.4 Hz, 2H), 7.45 (dd, *J* = 8.4, 3.4 Hz, 2H), 4.20 – 4.03 (m, 4H), 1.32 (t, *J* = 7.0 Hz, 6H). 13C NMR (126 MHz, CDCl3) δ 139.2 (d, J = 4.0 Hz), 133.4 (d, J = 10.7 Hz), 129.0 (d, J = 15.6 Hz), 127.2 (d, J = 191.0 Hz), 62.5 (d, J = 5.5 Hz), 16.5 (d, J = 6.5 Hz). ^{31}P NMR (202 MHz, $CDCl_3$) δ 17.87 – 17.37 (m).

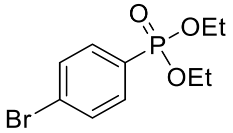

4

A 4 mL Electrasyn vial was charged with (4-bromophenyl)boronic acid (28.1 mg, 0.14 mmol, 1 eq.), potassium fluoride (23 mg, 0.4 mmol, 2.9 eq.) and triethyl phosphite (0.24 mL, 1.4 mmol, 10 eq.). A stir bar was added and the threads were wrapped with parafilm to ensure an airtight seal when cap added. 4 mL of a 0.1 M KF solution in (4:1) MeCN (3.2 mL): HFIP (0.8 mL). An electrasyn cap was prepared with 2 platinum plates (10 mm x 10 mm x 0.1 mm) and a rubber septa was used to cover the top cap (Figure S1). The cap was then twisted onto the vial, ensuring the electrodes aren't touching and are submerged in the solution. The solution was then purged with nitrogen for 5 minutes then was replaced with a N_2 balloon. This vial was equipped to an Electrasyn and the following conditions (20 mA, 0.5 Hz, 8 F/mol). Once the reaction was finished, 8.4 mg of 1,3,5-trimethoxybenzene was added as NMR internal standard. The crude solution was diluted with 25 mL ethyl acetate and washed with 10 mL saturated $NaHCO_3$ and 10 mL brine. The organic layer was collected and dried by $MgSO_4$. The organic phase was then concentrated under vacuum and the yield was determined by ^{1}H NMR (14%). The NMR data matches literature.[1] The crude product was attempted to purify by passing through a silica gel column (EA/hexane 10% - 60%). No desired product was obtained.

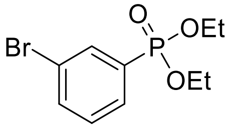

5

A 4 mL Electrasyn vial was charged with (3-bromophenyl)boronic acid (28.1 mg, 0.14 mmol, 1 eq.), potassium fluoride (23 mg, 0.4 mmol, 2.9 eq.) and triethyl phosphite (0.24 mL, 1.4 mmol, 10 eq.). A stir bar was added and the threads were wrapped with parafilm to ensure an airtight seal when cap added. 4 mL of a 0.1 M KF solution in (4:1) MeCN (3.2 mL): HFIP (0.8 mL). An electrasyn cap was prepared with 2 platinum plates (10 mm x 10 mm x 0.1 mm) and a rubber septa was used to cover the top cap (Figure S1). The cap was then twisted onto the vial, ensuring the electrodes aren't touching and are submerged in the solution. The solution was then purged with nitrogen for 5 minutes then was replaced with a N_2 balloon. This vial was equipped to an Electrasyn and the following conditions (20 mA, 0.5 Hz, 8 F/mol). Once the reaction was finished, 8.4 mg of 1,3,5-trimethoxybenzene was added as NMR internal standard. The crude solution was diluted with 25 mL ethyl acetate and washed with 10 mL saturated $NaHCO_3$ and 10 mL brine. The organic layer was collected and dried by $MgSO_4$. The organic phase was then concentrated under vacuum. The crude product was purified by passing through a silica gel column (EA/hexane 10% - 60%). The product collected after column was then dried on vacuum line overnight to afford yellow oil (50% yield with minor impurity). The NMR data matches literature.[2]

Diethyl (3-bromophenyl)phosphonate (5). ^{1}H NMR (500 MHz, $CDCl_3$) δ 7.95 (dt, J = 13.7, 1.7 Hz, 1H), 7.75 (ddt, J = 13.0, 7.6, 1.3 Hz, 1H), 7.69 (ddt, J = 8.1, 2.2, 1.1 Hz, 1H), 7.36 (td, J = 7.8, 4.8 Hz, 1H), 4.23 – 4.06 (m, 5H), 1.35 (t, J = 7.1 Hz, 7H). ^{31}P NMR (202 MHz, $CDCl_3$) δ 16.54 – 16.06 (m). ^{13}C NMR (126 MHz, $CDCl_3$) δ 135.7 (d, J = 3.0 Hz), 134.6 (d, J = 10.7 Hz), 130.4 (d, J = 1.6 Hz), 130.3 (d, J = 5.2 Hz), 123.1 (d, J = 19.9 Hz), 116.8 (d, J = 562.0 Hz), 62.7 (d, J = 5.7 Hz), 16.5 (d, J = 6.4 Hz).

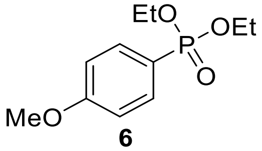

6

A 4 mL Electrasyn vial was charged with (4-methoxyphenyl)boronic acid (21.3 mg, 0.14 mmol, 1 eq.), potassium fluoride (23 mg, 0.4 mmol, 2.9 eq.) and triethyl phosphite (0.24 mL, 1.4 mmol, 10 eq.). A stir bar was added and the threads were wrapped with parafilm to ensure an airtight seal when cap added. 4 mL of a 0.1 M KF solution in (4:1) MeCN (3.2 mL): HFIP (0.8 mL). An electrasyn cap was prepared with 2 platinum plates (10 mm x 10 mm x 0.1 mm) and a rubber septa was used to cover the top cap (Figure S1). The cap was then twisted onto the vial, ensuring the electrodes aren't touching and are submerged in the solution. The solution was then purged with nitrogen for 5 minutes then was replaced with a N_2 balloon. This vial was equipped to an Electrasyn and the following conditions (20 mA, 0.5 Hz, 8 F/mol). Once the reaction was finished, 8.4 mg of 1,3,5-trimethoxybenzene was added as NMR internal standard. The crude solution was diluted with 25 mL ethyl acetate and washed with 10 mL saturated $NaHCO_3$ and 10 mL brine. The organic layer was collected and dried by $MgSO_4$. The organic phase was then concentrated under vacuum. The crude product was purified by passing through a silica gel column (EA/hexane 10% - 60%). The product collected after column was then dried on vacuum line overnight to afford yellow oil (53% yield). The NMR data matches literature.[1]

Diethyl (4-methoxyphenyl)phosphonate (6). ^{1}H NMR (500 MHz, $CDCl_3$) δ 7.74 (dd, J = 12.8, 8.7 Hz, 2H), 6.97 (dd, J = 8.7, 3.3 Hz, 2H), 4.16 – 4.01 (m, 4H), 3.85 (s, 3H), 1.31 (t, J = 7.1 Hz, 6H). ^{31}P NMR (202 MHz, CDCl3) δ 20.01 – 19.49 (m). ^{13}C NMR (126 MHz, $CDCl_3$) δ 163.0 (d, J = 3.4 Hz), 133.9 (d, J = 11.3 Hz), 119.6 (d, J = 195.0 Hz), 114.2 (d, J = 16.1 Hz), 62.1 (d, J = 5.3 Hz), 55.5, 16.5 (d, J = 6.6 Hz).

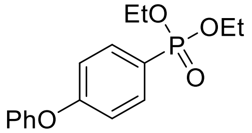

7

A 4 mL Electrasyn vial was charged with (4-phenoxyphenyl)boronic acid (30.0 mg, 0.14 mmol, 1 eq.), potassium fluoride (23 mg, 0.4 mmol, 2.9 eq.) and triethyl phosphite (0.24 mL, 1.4 mmol, 10

eq.). A stir bar was added and the threads were wrapped with parafilm to ensure an airtight seal when cap added. 4 mL of a 0.1 M KF solution in (4:1) MeCN (3.2 mL): HFIP (0.8 mL). An electrasyn cap was prepared with 2 platinum plates (10 mm x 10 mm x 0.1 mm) and a rubber septa was used to cover the top cap (Figure S1). The cap was then twisted onto the vial, ensuring the electrodes aren't touching and are submerged in the solution. The solution was then purged with nitrogen for 5 minutes then was replaced with a N_2 balloon. This vial was equipped to an Electrasyn and the following conditions (20 mA, 0.5 Hz, 8 F/mol). Once the reaction was finished, 8.4 mg of 1,3,5-trimethoxybenzene was added as NMR internal standard. The crude solution was diluted with 25 mL ethyl acetate and washed with 10 mL saturated $NaHCO_3$ and 10 mL brine. The organic layer was collected and dried by $MgSO_4$. The organic phase was then concentrated under vacuum. The crude product was purified by passing through a silica gel column (EA/hexane 10% - 60%). The product collected after column was then dried on vacuum line overnight to afford yellow oil (32% yield). The NMR data matches literature.[3]

Diethyl (4-phenoxyphenyl)phosphonate (7). ^{1}H NMR (500 MHz, $CDCl_3$) δ 7.80 – 7.73 (m, 2H), 7.43 – 7.37 (m, 2H), 7.23 – 7.17 (m, 1H), 7.09 – 7.06 (m, 2H), 7.06 – 7.01 (m, 2H), 4.21 – 4.04 (m, 4H), 1.34 (t, J = 7.1 Hz, 6H). ^{31}P NMR (202 MHz, $CDCl_3$) δ 19.19 – 18.72 (m). ^{13}C NMR (126 MHz, $CDCl_3$) δ 161.6 (d, J = 3.6 Hz), 155.6, 134.0 (d, J = 11.4 Hz), 130.2, 124.7, 122.0 (d, J = 193.5 Hz), 120.3, 117.7 (d, J = 15.9 Hz), 62.2 (d, J = 5.4 Hz), 16.5 (d, J = 6.4 Hz).

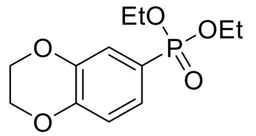

8

A 4 mL Electrasyn vial was charged with boronic acid (25.2 mg, 0.14 mmol, 1 eq.), potassium fluoride (23 mg, 0.4 mmol, 2.9 eq.) and triethyl phosphite (0.24 mL, 1.4 mmol, 10 eq.). A stir bar was added and the threads were wrapped with parafilm to ensure an airtight seal when cap added. 4 mL of a 0.1 M KF solution in (4:1) MeCN (3.2 mL): HFIP (0.8 mL). An electrasyn cap was prepared with 2 platinum plates (10 mm x 10 mm x 0.1 mm) and a rubber septa was used to cover the top cap (Figure S1). The cap was then twisted onto the vial, ensuring the electrodes aren't touching and are submerged in the solution. The solution was then purged with nitrogen for 5 minutes then was replaced with a N_2 balloon. This vial was equipped to an Electrasyn and the following conditions (20 mA, 0.5 Hz, 8 F/mol). Once the reaction was finished, 8.4 mg of 1,3,5-trimethoxybenzene was added as NMR internal standard. The crude solution was diluted with 25 mL ethyl acetate and washed with 10 mL saturated $NaHCO_3$ and 10 mL brine. The organic layer was collected and dried by $MgSO_4$. The organic phase was then concentrated under vacuum. The crude product was purified by passing through a silica gel column (EA/hexane 10% - 60%). The product collected after column was then dried on vacuum line overnight to afford yellow oil (46% yield). The NMR data matches literature.[4]

Diethyl (2,3-dihydrobenzo[b][1,4]dioxin-6-yl)phosphonate (8). ^{1}H NMR (500 MHz, $CDCl_3$) δ 7.34 – 7.27 (m, 2H), 6.94 (dd, *J* = 8.1, 4.6 Hz, 1H), 4.33 – 4.26 (m, 4H), 4.17 – 4.01 (m, 4H), 1.32 (t, *J* = 7.0 Hz, 6H). ^{31}P NMR (202 MHz, $CDCl_3$) δ 19.23 – 18.75 (m). ^{13}C NMR (126 MHz, $CDCl_3$) δ 147.4 (d, *J* = 3.5 Hz), 143.7 (d, *J* = 20.9 Hz), 125.6 (d, *J* = 10.2 Hz), 121.2 (d, *J* = 12.1 Hz), 120.7 (d, *J* = 193.5 Hz), 117.8 (d, *J* = 17.7 Hz), 64.7, 64.3, 62.2 (d, *J* = 5.3 Hz), 16.5 (d, *J* = 6.6 Hz).

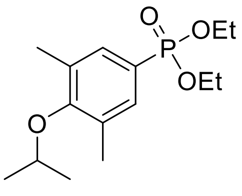

9

A 4 mL Electrasyn vial was charged with boronic acid (29.1 mg, 0.14 mmol, 1 eq.), potassium fluoride (23 mg, 0.4 mmol, 2.9 eq.) and triethyl phosphite (0.24 mL, 1.4 mmol, 10 eq.). A stir bar was added and the threads were wrapped with parafilm to ensure an airtight seal when cap added. 4 mL of a 0.1 M KF solution in (4:1) MeCN (3.2 mL): HFIP (0.8 mL). An electrasyn cap was prepared with 2 platinum plates (10 mm x 10 mm x 0.1 mm) and a rubber septa was used to cover the top cap (Figure S1). The cap was then twisted onto the vial, ensuring the electrodes aren't touching and are submerged in the solution. The solution was then purged with nitrogen for 5 minutes then was replaced with a N_2 balloon. This vial was equipped to an Electrasyn and the following conditions (20 mA, 0.5 Hz, 8 F/mol). Once the reaction was finished, 8.4 mg of 1,3,5-trimethoxybenzene was added as NMR internal standard. The crude solution was diluted with 25 mL ethyl acetate and washed with 10 mL saturated $NaHCO_3$ and 10 mL brine. The organic layer was collected and dried by $MgSO_4$. The organic phase was then concentrated under vacuum. The crude product was purified by passing through a silica gel column (EA/hexane 10% - 60%). The product collected after column was then dried on vacuum line overnight to afford yellow oil (62% yield). The NMR data matches literature.[5]

Diethyl (4-isopropoxy-3,5-dimethylphenyl)phosphonate (9). ^{1}H NMR (500 MHz, $CDCl_3$) δ 7.46 (s, 1H), 7.44 (s, 1H), 4.24 (hept, *J* = 6.2 Hz, 1H), 4.18 – 4.01 (m, 4H), 2.29 (s, 6H), 1.33 (t, *J* = 7.1 Hz, 6H), 1.29 (d, *J* = 6.1 Hz, 6H). ^{31}P NMR (202 MHz, $CDCl_3$) δ 20.18 – 19.77 (m). ^{13}C NMR (126 MHz, $CDCl_3$) δ 158.7 (d, *J* = 3.9 Hz), 132.7 (d, *J* = 10.6 Hz), 132.0 (d, *J* = 16.3 Hz), 122.3 (d, *J* = 190.5 Hz), 75.0, 62.1 (d, *J* = 5.4 Hz), 22.7, 17.3, 16.5 (d, *J* = 6.4 Hz).

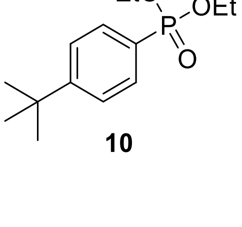

10

A 4 mL Electrasyn vial was charged with (4-(tert-butyl)phenyl)boronic acid (24.9 mg, 0.14 mmol, 1 eq.), potassium fluoride (23 mg, 0.4 mmol, 2.9 eq.) and triethyl phosphite (0.24 mL, 1.4 mmol, 10 eq.). A stir bar was added and the threads were wrapped with parafilm to ensure an airtight seal when cap added. 4 mL of a 0.1 M KF solution in (4:1) MeCN (3.2 mL): HFIP (0.8 mL). An electrasyn cap was prepared with 2 platinum plates (10 mm x 10 mm x 0.1 mm) and a rubber septa was used to cover the top cap (Figure S1). The cap was then twisted onto the vial, ensuring the electrodes aren't touching and are submerged in the solution. The solution was then purged with nitrogen for 5 minutes then was replaced with a N_2 balloon. This vial was equipped to an Electrasyn and the following conditions (20 mA, 0.5 Hz, 8 F/mol). Once the reaction was finished, 8.4 mg of 1,3,5-trimethoxybenzene was added as NMR internal standard. The crude solution was diluted with 25 mL ethyl acetate and washed with 10 mL saturated $NaHCO_3$ and 10 mL brine. The organic layer was collected and dried by $MgSO_4$. The organic phase was then concentrated under vacuum. The crude product was purified by passing through a silica gel column (EA/hexane 10% - 60%). The product collected after column was then dried on vacuum line overnight to afford yellow oil (70% yield). The NMR data matches literature.[1]

Diethyl (4-(tert-butyl)phenyl)phosphonate (10). ^{1}H NMR (500 MHz, $CDCl_3$) δ 7.74 (dd, J = 13.0, 8.3 Hz, 2H), 7.49 (dd, J = 8.4, 3.9 Hz, 2H), 4.20 – 4.03 (m, 4H), 1.34 (d, J = 4.2 Hz, 15H). ^{31}P NMR (202 MHz, $CDCl_3$) δ 19.72 – 19.28 (m). ^{13}C NMR (126 MHz, $CDCl_3$) δ 156.1 (d, J = 3.2 Hz), 131.8 (d, J = 10.2 Hz), 125.6 (d, J = 15.2 Hz), 125.0 (d, J = 190.1 Hz), 62.2 (d, J = 5.4 Hz), 35.2, 31.2, 16.5 (d, J = 6.6 Hz).

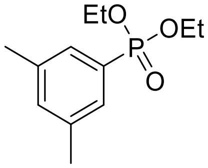

11

A 4 mL Electrasyn vial was charged with boronic acid (21.0 mg, 0.14 mmol, 1 eq.), potassium fluoride (23 mg, 0.4 mmol, 2.9 eq.) and triethyl phosphite (0.24 mL, 1.4 mmol, 10 eq.). A stir bar was added and the threads were wrapped with parafilm to ensure an airtight seal when cap added. 4 mL of a 0.1 M KF solution in (4:1) MeCN (3.2 mL): HFIP (0.8 mL). An electrasyn cap was prepared with 2 platinum plates (10 mm x 10 mm x 0.1 mm) and a rubber septa was used to cover the top cap (Figure S1). The cap was then twisted onto the vial, ensuring the electrodes aren't touching and are submerged in the solution. The solution was then purged with nitrogen for 5 minutes then was replaced with a N_2 balloon. This vial was equipped to an Electrasyn and the following conditions (20 mA, 0.5 Hz, 8 F/mol). Once the reaction was finished, 8.4 mg of 1,3,5-trimethoxybenzene was added as NMR internal standard. The crude solution was diluted with 25 mL ethyl acetate and washed with 10 mL saturated $NaHCO_3$ and 10 mL brine. The organic layer was collected and dried by $MgSO_4$. The organic phase was then concentrated under vacuum and

the yield was determined by ^{1}H NMR (56% yield). The crude product was purified by passing through a silica gel column (EA/hexane 10% - 60%). The product collected after column was then dried on vacuum line overnight to afford yellow oil (40% yield). The isolation yield was not accurate due to the volatility of product. The NMR data matches literature.[6]

Diethyl (3,5-dimethylphenyl)phosphonate (11). ^{1}H NMR (500 MHz, $CDCl_3$) δ 7.45 (s, 1H), 7.42 (s, 1H), 7.19 (s, 1H), 4.20 – 4.03 (m, 4H), 2.36 (s, 6H), 1.34 (t, *J* = 7.1 Hz, 6H). ^{31}P NMR (202 MHz, $CDCl_3$) δ 20.00 – 19.58 (m). ^{13}C NMR (126 MHz, $CDCl_3$) δ 138.4 (d, *J* = 15.8 Hz), 134.3 (d, *J* = 3.4 Hz), 129.5 (d, *J* = 9.9 Hz), 128.0 (d, *J* = 186.1 Hz), 62.2 (d, *J* = 5.4 Hz), 21.4 (d, *J* = 1.3 Hz), 16.5 (d, *J* = 6.5 Hz).

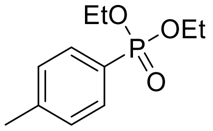

12

A 4 mL Electrasyn vial was charged with (4-methylphenyl)boronic acid (19.0 mg, 0.14 mmol, 1 eq.), potassium fluoride (23 mg, 0.4 mmol, 2.9 eq.) and triethyl phosphite (0.24 mL, 1.4 mmol, 10 eq.). A stir bar was added and the threads were wrapped with parafilm to ensure an airtight seal when cap added. 4 mL of a 0.1 M KF solution in (4:1) MeCN (3.2 mL): HFIP (0.8 mL). An electrasyn cap was prepared with 2 platinum plates (10 mm x 10 mm x 0.1 mm) and a rubber septa was used to cover the top cap (Figure S1). The cap was then twisted onto the vial, ensuring the electrodes aren't touching and are submerged in the solution. The solution was then purged with nitrogen for 5 minutes then was replaced with a N_2 balloon. This vial was equipped to an Electrasyn and the following conditions (20 mA, 0.5 Hz, 8 F/mol). Once the reaction was finished, 8.4 mg of 1,3,5-trimethoxybenzene was added as NMR internal standard. The crude solution was diluted with 25 mL ethyl acetate and washed with 10 mL saturated $NaHCO_3$ and 10 mL brine. The organic layer was collected and dried by $MgSO_4$. The organic phase was then concentrated under vacuum and the yield was determined by ^{1}H NMR (66% yield). The crude product was purified by passing through a silica gel column (EA/hexane 10% - 60%). The product collected after column was then dried on vacuum line overnight to afford yellow oil. The isolation yield was not accurate due to the volatility of product. The NMR data matches literature.[4]

Diethyl p-tolylphosphonate (12). ^{1}H NMR (500 MHz, $CDCl_3$) δ 7.71 (dd, *J* = 13.1, 8.0 Hz, 2H), 7.28 (dd, *J* = 8.0, 4.0 Hz 2H), 4.19 – 4.02 (m, 4H), 2.41 (s, 3H), 1.32 (t, *J* = 7.0 Hz, 6H). ^{31}P NMR (202 MHz, $CDCl_3$) δ 19.78 – 19.32 (m). ^{13}C NMR (126 MHz, $CDCl_3$) δ 143.1 (d, *J* = 3.2 Hz), 132.0 (d, *J* = 10.3 Hz), 129.4 (d, *J* = 15.4 Hz), 125.1 (d, *J* = 190.1 Hz), 62.2 (d, *J* = 5.3 Hz), 21.8 (d, *J* = 1.4 Hz), 16.5 (d, *J* = 6.5 Hz).

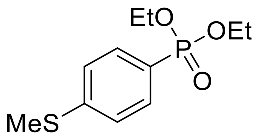

13

A 4 mL Electrasyn vial was charged with (4-(methylthio)phenyl)boronic acid (23.5 mg, 0.14 mmol, 1 eq.), potassium fluoride (23 mg, 0.4 mmol, 2.9 eq.) and triethyl phosphite (0.24 mL, 1.4 mmol, 10 eq.). A stir bar was added and the threads were wrapped with parafilm to ensure an airtight seal when cap added. 4 mL of a 0.1 M KF solution in (4:1) MeCN (3.2 mL): HFIP (0.8 mL). An electrasyn cap was prepared with 2 platinum plates (10 mm x 10 mm x 0.1 mm) and a rubber septa was used to cover the top cap (Figure S1). The cap was then twisted onto the vial, ensuring the electrodes aren't touching and are submerged in the solution. The solution was then purged with nitrogen for 5 minutes then was replaced with a N_2 balloon. This vial was equipped to an Electrasyn and the following conditions (20 mA, 0.5 Hz, 8 F/mol). Once the reaction was finished, 8.4 mg of 1,3,5-trimethoxybenzene was added as NMR internal standard. The crude solution was diluted with 25 mL ethyl acetate and washed with 10 mL saturated $NaHCO_3$ and 10 mL brine. The organic layer was collected and dried by $MgSO_4$. The organic phase was then concentrated under vacuum. The crude product was purified by passing through a silica gel column (EA/hexane 10% - 60%). The product collected after column was then dried on vacuum line overnight to afford yellow oil (36% yield). The NMR data matches literature.[7]

Diethyl (4-(methylthio)phenyl)phosphonate (13). ^{1}H NMR (500 MHz, $CDCl_3$) 7.71 (dd, J = 13.0, 8.4 Hz, 2H), 7.30 (dd, J = 8.3, 3.7 Hz, 2H), 4.19 – 4.02 (m, 4H), 2.52 (s, 3H), 1.33 (t, J = 7.1 Hz, 6H). ^{31}P NMR (202 MHz, $CDCl_3$) δ 19.28 – 18.86 (m). ^{13}C NMR (126 MHz, $CDCl_3$) δ 145.1 (d, J = 3.5 Hz), 132.2 (d, J = 10.6 Hz), 125.4 (d, J = 15.5 Hz), 123.9 (d, J = 192.3 Hz), 62.3 (d, J = 5.4 Hz), 16.5 (d, J = 6.6 Hz), 14.9.

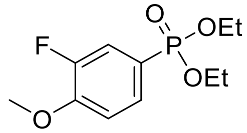

14

A 4 mL Electrasyn vial was charged with boronic acid (23.8 mg, 0.14 mmol, 1 eq.), potassium fluoride (23 mg, 0.4 mmol, 2.9 eq.) and triethyl phosphite (0.24 mL, 1.4 mmol, 10 eq.). A stir bar was added and the threads were wrapped with parafilm to ensure an airtight seal when cap added. 4 mL of a 0.1 M KF solution in (4:1) MeCN (3.2 mL): HFIP (0.8 mL). An electrasyn cap was prepared with 2 platinum plates (10 mm x 10 mm x 0.1 mm) and a rubber septa was used to cover the top cap (Figure S1). The cap was then twisted onto the vial, ensuring the electrodes aren't touching and are submerged in the solution. The solution was then purged with nitrogen for 5 minutes then was replaced with a N_2 balloon. This vial was equipped to an Electrasyn and the

following conditions (20 mA, 0.5 Hz, 8 F/mol). Once the reaction was finished, 8.4 mg of 1,3,5-trimethoxybenzene was added as NMR internal standard. The crude solution was diluted with 25 mL ethyl acetate and washed with 10 mL saturated $NaHCO_3$ and 10 mL brine. The organic layer was collected and dried by $MgSO_4$. The organic phase was then concentrated under vacuum. The crude product was purified by passing through a silica gel column (EA/hexane 10% - 60%). The product collected after column was then dried on vacuum line overnight to afford yellow oil (62% yield). The NMR data matches literature.[8]

Diethyl (3-fluoro-4-methoxyphenyl)phosphonate (14). ^{1}H NMR (500 MHz, $CDCl_3$) δ 7.58 (ddt, J = 13.3, 8.5, 1.5 Hz, 1H), 7.48 (ddd, J = 13.7, 10.9, 1.8 Hz, 1H), 7.03 (td, J = 8.2, 4.4 Hz, 1H), 4.19 – 4.02 (m, 4H), 3.94 (s, 3H), 1.32 (t, J = 7.1 Hz, 6H). ^{31}P NMR (202 MHz, $CDCl_3$) δ 17.90 – 17.35 (m). ^{19}F NMR (470 MHz, $CDCl_3$) δ -133.90 – -134.25 (m). ^{13}C NMR (126 MHz, $CDCl_3$) δ 152.1 (dd, J = 249.2, 21.8 Hz), 151.4 (dd, J = 10.5, 3.3 Hz), 129.2 (dd, J = 10.1, 3.9 Hz), 120.5 (dd, J = 195.3, 4.6 Hz), 119.3 (dd, J = 19.2, 11.4 Hz), 113.2 (dd, J = 18.4, 1.8 Hz), 62.3 (d, J = 5.4 Hz), 56.3, 16.4 (d, J = 6.4 Hz).

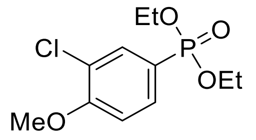

15

A 4 mL Electrasyn vial was charged with boronic acid (26.1 mg, 0.14 mmol, 1 eq.), potassium fluoride (23 mg, 0.4 mmol, 2.9 eq.) and triethyl phosphite (0.24 mL, 1.4 mmol, 10 eq.). A stir bar was added and the threads were wrapped with parafilm to ensure an airtight seal when cap added. 4 mL of a 0.1 M KF solution in (4:1) MeCN (3.2 mL): HFIP (0.8 mL). An electrasyn cap was prepared with 2 platinum plates (10 mm x 10 mm x 0.1 mm) and a rubber septa was used to cover the top cap (Figure S1). The cap was then twisted onto the vial, ensuring the electrodes aren't touching and are submerged in the solution. The solution was then purged with nitrogen for 5 minutes then was replaced with a N_2 balloon. This vial was equipped to an Electrasyn and the following conditions (20 mA, 0.5 Hz, 8 F/mol). Once the reaction was finished, 8.4 mg of 1,3,5-trimethoxybenzene was added as NMR internal standard. The crude solution was diluted with 25 mL ethyl acetate and washed with 10 mL saturated $NaHCO_3$ and 10 mL brine. The organic layer was collected and dried by $MgSO_4$. The organic phase was then concentrated under vacuum and the crude yield was determined by ^{1}H NMR (69% yield). The crude product was purified by passing through a silica gel column (EA/hexane 10% - 60%). The product collected after column was then dried on vacuum line overnight to afford yellow oil (45% yield).

Diethyl (3-chloro-4-methoxyphenyl)phosphonate (15). ^{1}H NMR (500 MHz, $CDCl_3$) δ 7.79 (dd, J = 13.1, 1.9 Hz, 1H), 7.72 (ddd, J = 12.9, 8.3, 1.9 Hz, 1H), 7.00 (dd, J = 8.4, 3.9 Hz, 1H), 4.20 – 4.03 (m, 4H), 3.96 (s, 3H), 1.33 (t, J = 7.1 Hz, 6H). ^{31}P NMR (202 MHz, $CDCl_3$) δ 17.82 – 17.32

(m). ^{13}C NMR (126 MHz, $CDCl_3$) δ 158.4 (d, J = 3.2 Hz), 133.7 (d, J = 11.7 Hz), 132.3 (d, J = 10.5 Hz), 123.2 (d, J = 20.7 Hz), 121.1 (d, J = 195.0 Hz), 111.9 (d, J = 17.2 Hz), 62.4 (d, J = 5.3 Hz), 56.4, 16.5 (d, J = 6.5 Hz).

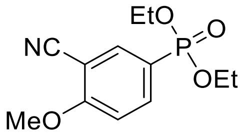

16

A 4 mL Electrasyn vial was charged with boronic acid (24.8 mg, 0.14 mmol, 1 eq.), potassium fluoride (23 mg, 0.4 mmol, 2.9 eq.) and triethyl phosphite (0.24 mL, 1.4 mmol, 10 eq.). A stir bar was added and the threads were wrapped with parafilm to ensure an airtight seal when cap added. 4 mL of a 0.1 M KF solution in (4:1) MeCN (3.2 mL): HFIP (0.8 mL). An electrasyn cap was prepared with 2 platinum plates (10 mm x 10 mm x 0.1 mm) and a rubber septa was used to cover the top cap (Figure S1). The cap was then twisted onto the vial, ensuring the electrodes aren't touching and are submerged in the solution. The solution was then purged with nitrogen for 5 minutes then was replaced with a N_2 balloon. This vial was equipped to an Electrasyn and the following conditions (20 mA, 0.5 Hz, 8 F/mol). Once the reaction was finished, 8.4 mg of 1,3,5-trimethoxybenzene was added as NMR internal standard. The crude solution was diluted with 25 mL ethyl acetate and washed with 10 mL saturated $NaHCO_3$ and 10 mL brine. The organic layer was collected and dried by $MgSO_4$. The organic phase was then concentrated under vacuum. The crude product was purified by passing through a silica gel column (EA/hexane 10% - 60%). The product collected after column was then dried on vacuum line overnight to afford yellow oil (53% yield). The NMR data matches literature.[8]

Diethyl (3-cyano-4-methoxyphenyl)phosphonate (16). ^{1}H NMR (500 MHz, $CDCl_3$) δ 8.04 – 7.95 (m, 2H), 7.07 (dd, J = 9.0, 2.8 Hz, 1H), 4.21 – 4.04 (m, 4H), 4.00 (s, 3H), 1.34 (t, J = 7.1 Hz, 6H). ^{31}P NMR (202 MHz, $CDCl_3$) δ 16.27 – 15.79 (m). ^{13}C NMR (126 MHz, $CDCl_3$) δ 164.0 (d, J = 3.3 Hz), 138.0 (dd, J = 67.3, 11.4 Hz), 121.4 (d, J = 197.6 Hz), 115.5 (d, J = 1.8 Hz), 111.5 (d, J = 15.7 Hz), 102.7 (dd, J = 18.0, 1.2 Hz), 62.6 (d, J = 5.6 Hz), 56.6, 16.4 (d, J = 6.4 Hz).

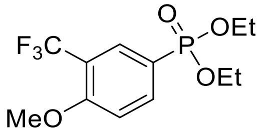

17

A 4 mL Electrasyn vial was charged with boronic acid (30.8 mg, 0.14 mmol, 1 eq.), potassium fluoride (23 mg, 0.4 mmol, 2.9 eq.) and triethyl phosphite (0.24 mL, 1.4 mmol, 10 eq.). A stir bar was added and the threads were wrapped with parafilm to ensure an airtight seal when cap added.

4 mL of a 0.1 M KF solution in (4:1) MeCN (3.2 mL): HFIP (0.8 mL). An electrasyn cap was prepared with 2 platinum plates (10 mm x 10 mm x 0.1 mm) and a rubber septa was used to cover the top cap (Figure S1). The cap was then twisted onto the vial, ensuring the electrodes aren't touching and are submerged in the solution. The solution was then purged with nitrogen for 5 minutes then was replaced with a N_2 balloon. This vial was equipped to an Electrasyn and the following conditions (20 mA, 0.5 Hz, 8 F/mol). Once the reaction was finished, 8.4 mg of 1,3,5-trimethoxybenzene was added as NMR internal standard. The crude solution was diluted with 25 mL ethyl acetate and washed with 10 mL saturated $NaHCO_3$ and 10 mL brine. The organic layer was collected and dried by $MgSO_4$. The organic phase was then concentrated under vacuum. The crude product was purified by passing through a silica gel column (EA/hexane 10% - 60%). The product collected after column was then dried on vacuum line overnight to afford yellow oil (72% yield).

Diethyl (4-methoxy-3-(trifluoromethyl)phenyl)phosphonate (17). ^{1}H NMR (500 MHz, $CDCl_3$) δ 8.02 – 7.93 (m, 2H), 7.09 (dd, *J* = 8.5, 3.2 Hz, 1H), 4.20 – 4.04 (m, 4H), 3.97 (s, 3H), 1.34 (t, *J* = 7.1 Hz, 6H). ^{13}C NMR (126 MHz, $CDCl_3$) δ 160.6 (dq, *J* = 3.2, 1.6 Hz), 137.6 (d, *J* = 11.0 Hz), 131.0 (dq, *J* = 12.5, 5.2 Hz), 123.1 (qd, *J* = 272.7, 2.1 Hz), 119.8 (d, *J* = 197.8 Hz), 119.1 (qd, *J* = 31.5, 16.1 Hz), 111.9 (d, *J* = 15.9 Hz), 62.4 (d, *J* = 5.5 Hz), 56.2, 16.3 (d, *J* = 6.5 Hz).^{31}P NMR (202 MHz, $CDCl_3$) δ 17.77 – 17.24 (m). ^{19}F NMR (470 MHz, $CDCl_3$) δ -62.89. HRMS (ESI/Q-TOF) m/z [M+H]+ Calcd for C12H17F3O4P 313.0812; Found 313.0823.

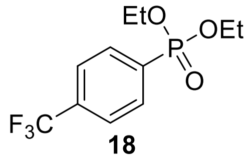

18

A 4 mL Electrasyn vial was charged with (4-(trifluoromethyl)phenyl)boronic acid (26.6 mg, 0.14 mmol, 1 eq.), potassium fluoride (23 mg, 0.4 mmol, 2.9 eq.) and triethyl phosphite (0.24 mL, 1.4 mmol, 10 eq.). A stir bar was added and the threads were wrapped with parafilm to ensure an airtight seal when cap added. 4 mL of a 0.1 M KF solution in (4:1) MeCN (3.2 mL): HFIP (0.8 mL). An electrasyn cap was prepared with 2 platinum plates (10 mm x 10 mm x 0.1 mm) and a rubber septa was used to cover the top cap (Figure S1). The cap was then twisted onto the vial, ensuring the electrodes aren't touching and are submerged in the solution. The solution was then purged with nitrogen for 5 minutes then was replaced with a N_2 balloon. This vial was equipped to an Electrasyn and the following conditions (20 mA, 0.1 Hz, 8 F/mol). Once the reaction was finished, 8.4 mg of 1,3,5-trimethoxybenzene was added as NMR internal standard. The crude solution was diluted with 25 mL ethyl acetate and washed with 10 mL saturated $NaHCO_3$ and 10 mL brine. The organic layer was collected and dried by $MgSO_4$. The organic phase was then concentrated under vacuum and the yield was determined by ^{1}H NMR (58% yield). The crude product was purified by passing through a silica gel column (EA/hexane 10% - 60%). The product

collected after column was then dried on vacuum line overnight to afford yellow oil (41% yield). The NMR data matches literature.[4]

Diethyl (4-(trifluoromethyl)phenyl)phosphonate (18). ^{1}H NMR (500 MHz, $CDCl_3$) δ 7.96 (dd, *J* = 13.0, 7.9 Hz, 2H), 7.74 (dd, *J* = 8.2, 3.5 Hz, 2H), 4.25 – 4.07 (m, 4H), 1.35 (t, *J* = 7.0 Hz, 6H). ^{31}P NMR (202 MHz, $CDCl_3$) δ 16.52 – 16.01 (m). ^{19}F NMR (470 MHz, $CDCl_3$) δ -63.29. ^{13}C NMR (126 MHz, $CDCl_3$) δ 134.3 (qd, *J* = 32.7, 3.3 Hz), 132.9 (d, *J* = 188.0 Hz), 132.4 (d, *J* = 10.1 Hz), 125.5 (dq, *J* = 15.1, 3.7 Hz), 123.7 (q, *J* = 272.7 Hz), 62.7 (d, *J* = 5.6 Hz), 16.5 (d, *J* = 6.4 Hz).

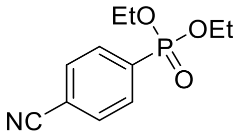

19

A 4 mL Electrasyn vial was charged with (4-cyanophenyl)boronic acid (20.6 mg, 0.14 mmol, 1 eq.), potassium fluoride (23 mg, 0.4 mmol, 2.9 eq.) and triethyl phosphite (0.24 mL, 1.4 mmol, 10 eq.). A stir bar was added and the threads were wrapped with parafilm to ensure an airtight seal when cap added. 4 mL of a 0.1 M KF solution in (4:1) MeCN (3.2 mL): HFIP (0.8 mL). An electrasyn cap was prepared with 2 platinum plates (10 mm x 10 mm x 0.1 mm) and a rubber septa was used to cover the top cap (Figure S1). The cap was then twisted onto the vial, ensuring the electrodes aren't touching and are submerged in the solution. The solution was then purged with nitrogen for 5 minutes then was replaced with a N_2 balloon. This vial was equipped to an Electrasyn and the following conditions (20 mA, 0.1 Hz, 8 F/mol). Once the reaction was finished, 8.4 mg of 1,3,5-trimethoxybenzene was added as NMR internal standard. The crude solution was diluted with 25 mL ethyl acetate and washed with 10 mL saturated $NaHCO_3$ and 10 mL brine. The organic layer was collected and dried by $MgSO_4$. The organic phase was then concentrated under vacuum. The crude product was purified by passing through a silica gel column (EA/hexane 10% - 60%). The product collected after column was then dried on vacuum line overnight to afford yellow oil (53% yield). The NMR data matches literature.[4]

Diethyl (4-cyanophenyl)phosphonate (19). ^{1}H NMR (500 MHz, $CDCl_3$) δ 7.94 (dd, *J* = 13.1, 8.3 Hz, 2H), 7.77 (dd, *J* = 8.3, 3.6 Hz, 2H), 4.25 – 4.08 (m, 4H), 1.35 (t, *J* = 7.1 Hz, 6H). ^{31}P NMR (202 MHz, $CDCl_3$) δ 15.59 – 15.09 (m). ^{13}C NMR (126 MHz, $CDCl_3$) δ 134.1 (d, *J* = 187.8 Hz), 132.4 (d, *J* = 9.9 Hz), 132.2 (d, *J* = 15.0 Hz), 118.0 (d, *J* = 1.6 Hz), 116.2 (d, *J* = 3.6 Hz), 62.9 (d, *J* = 5.6 Hz), 16.5 (d, *J* = 6.3 Hz).

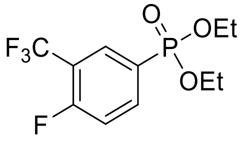

20

A 4 mL Electrasyn vial was charged with boronic acid (29.1 mg, 0.14 mmol, 1 eq.), potassium fluoride (23 mg, 0.4 mmol, 2.9 eq.) and triethyl phosphite (0.24 mL, 1.4 mmol, 10 eq.). A stir bar was added and the threads were wrapped with parafilm to ensure an airtight seal when cap added. 4 mL of a 0.1 M KF solution in (4:1) MeCN (3.2 mL): HFIP (0.8 mL). An electrasyn cap was prepared with 2 platinum plates (10 mm x 10 mm x 0.1 mm) and a rubber septa was used to cover the top cap (Figure S1). The cap was then twisted onto the vial, ensuring the electrodes aren't touching and are submerged in the solution. The solution was then purged with nitrogen for 5 minutes then was replaced with a N_2 balloon. This vial was equipped to an Electrasyn and the following conditions (20 mA, 0.1 Hz, 8 F/mol). Once the reaction was finished, 8.4 mg of 1,3,5-trimethoxybenzene was added as NMR internal standard. The crude solution was diluted with 25 mL ethyl acetate and washed with 10 mL saturated $NaHCO_3$ and 10 mL brine. The organic layer was collected and dried by $MgSO_4$. The organic phase was then concentrated under vacuum. The crude product was purified by passing through a silica gel column (EA/hexane 10% - 60%). The product collected after column was then dried on vacuum line overnight to afford yellow oil (47% yield).

Diethyl (4-fluoro-3-(trifluoromethyl)phenyl)phosphonate (20). ^{1}H NMR (500 MHz, $CDCl_3$) δ 8.12 – 7.99 (m, 2H), 7.33 (ddd, *J* = 11.3, 8.5, 3.1 Hz, 1H), 4.25 – 4.07 (m, 4H), 1.36 (t, *J* = 7.1 Hz, 6H). ^{13}C NMR (126 MHz, $CDCl_3$) δ 162.2 (ddd, *J* = 263.4, 3.9, 2.0 Hz), 137.9 (t, *J* = 10.1 Hz), 131.5 – 131.2 (m), 125.6 (dd, *J* = 195.0, 4.2 Hz), 122.2 (q, *J* = 272.7 Hz), 119.0 (ddd, *J* = 33.8, 16.9, 4.2 Hz), 117.4 (dd, *J* = 20.8, 15.7 Hz), 62.7 (d, *J* = 5.7 Hz), 16.3 (d, *J* = 6.3 Hz).^{31}P NMR (202 MHz, $CDCl_3$) δ 15.76 – 15.09 (m). ^{19}F NMR (470 MHz, $CDCl_3$) δ -61.61 (d, *J* = 12.7 Hz), -107.42 – -107.53 (m). HRMS (ESI/Q-TOF) m/z [M+H]+ Calcd for C11H14F4O3P 301.0611; Found 301.0623.

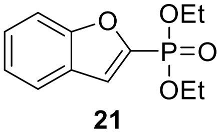

21

A 4 mL Electrasyn vial was charged with boronic acid (22.7 mg, 0.14 mmol, 1 eq.), potassium fluoride (23 mg, 0.4 mmol, 2.9 eq.) and triethyl phosphite (0.24 mL, 1.4 mmol, 10 eq.). A stir bar was added and the threads were wrapped with parafilm to ensure an airtight seal when cap added. 4 mL of a 0.1 M KF solution in (4:1) MeCN (3.2 mL): HFIP (0.8 mL). An electrasyn cap was prepared with 2 platinum plates (10 mm x 10 mm x 0.1 mm) and a rubber septa was used to cover the top cap (Figure S1). The cap was then twisted onto the vial, ensuring the electrodes aren't touching and are submerged in the solution. The solution was then purged with nitrogen for 5

minutes then was replaced with a N_2 balloon. This vial was equipped to an Electrasyn and the following conditions (20 mA, 0.5 Hz, 8 F/mol). Once the reaction was finished, 8.4 mg of 1,3,5-trimethoxybenzene was added as NMR internal standard. The crude solution was diluted with 25 mL ethyl acetate and washed with 10 mL saturated $NaHCO_3$ and 10 mL brine. The organic layer was collected and dried by $MgSO_4$. The organic phase was then concentrated under vacuum. The crude product was purified by passing through a silica gel column (EA/hexane 10% - 60%). The product collected after column was then dried on vacuum line overnight to afford yellow oil (70% yield). The NMR data matches literature.[9]

Diethyl benzofuran-2-ylphosphonate (21). ^{1}H NMR (500 MHz, $CDCl_3$) δ 7.69 (d, J = 7.8 Hz, 1H), 7.58 (d, J = 8.4 Hz, 1H), 7.52 (d, J = 3.1 Hz, 1H), 7.44 (t, J = 7.7 Hz, 1H), 7.31 (t, J = 7.5 Hz, 1H), 4.32 – 4.15 (m, 4H), 1.38 (t, J = 7.1 Hz, 6H). ^{31}P NMR (202 MHz, $CDCl_3$) δ 4.72 – 4.40 (m). ^{13}C NMR (126 MHz, $CDCl_3$) δ 157.5 (d, J = 11.6 Hz), 145.9 (d, J = 237.0 Hz), 127.2, 126.5 (d, J = 12.3 Hz), 123.8 (d, J = 1.3 Hz), 122.6, 119.3 (d, J = 24.2 Hz), 112.3 (d, J = 1.5 Hz), 63.4 (d, J = 5.3 Hz), 16.4 (d, J = 6.4 Hz).

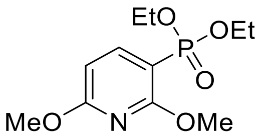

22

A 4 mL Electrasyn vial was charged with boronic acid (25.6 mg, 0.14 mmol, 1 eq.), potassium fluoride (23 mg, 0.4 mmol, 2.9 eq.) and triethyl phosphite (0.24 mL, 1.4 mmol, 10 eq.). A stir bar was added and the threads were wrapped with parafilm to ensure an airtight seal when cap added. 4 mL of a 0.1 M KF solution in (4:1) MeCN (3.2 mL): HFIP (0.8 mL). An electrasyn cap was prepared with 2 platinum plates (10 mm x 10 mm x 0.1 mm) and a rubber septa was used to cover the top cap (Figure S1). The cap was then twisted onto the vial, ensuring the electrodes aren't touching and are submerged in the solution. The solution was then purged with nitrogen for 5 minutes then was replaced with a N_2 balloon. This vial was equipped to an Electrasyn and the following conditions (20 mA, 0.5 Hz, 8 F/mol). Once the reaction was finished, 8.4 mg of 1,3,5-trimethoxybenzene was added as NMR internal standard. The crude solution was diluted with 25 mL ethyl acetate and washed with 10 mL saturated $NaHCO_3$ and 10 mL brine. The organic layer was collected and dried by $MgSO_4$. The organic phase was then concentrated under vacuum. The crude product was purified by passing through a silica gel column (EA/hexane 10% - 60%). The product collected after column was then dried on vacuum line overnight to afford yellow oil (82% yield). The NMR data matches literature.[10]

Diethyl (2,6-dimethoxypyridin-3-yl)phosphonate (22). ^{1}H NMR (500 MHz, $CDCl_3$) δ 7.97 (dd, J = 13.2, 8.2 Hz, 1H), 6.35 (dd, J = 8.2, 1.9 Hz, 1H), 4.19 – 4.05 (m, 4H), 4.01 (s, 3H), 3.95 (s, 3H), 1.32 (t, J = 7.0 Hz, 6H). ^{31}P NMR (202 MHz, $CDCl_3$) δ 17.01 – 16.65 (m). ^{13}C NMR (126

MHz, $CDCl_3$) δ 166.4, 164.3 (d, J = 7.0 Hz), 147.0 (d, J = 8.7 Hz), 101.7 (d, J = 11.8 Hz), 100.6 (d, J = 200.3 Hz), 62.3 (d, J = 5.4 Hz), 53.9 (d, J = 5.4 Hz), 16.42 (d, J = 6.5 Hz).

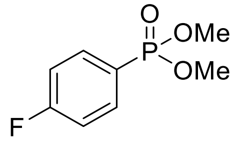

23

A 4 mL Electrasyn vial was charged with boronic acid (19.6 mg, 0.14 mmol, 1 eq.), potassium fluoride (23 mg, 0.4 mmol, 2.9 eq.) and trimethyl phosphite (0.16 mL, 1.4 mmol, 10 eq.). A stir bar was added and the threads were wrapped with parafilm to ensure an airtight seal when cap added. 4 mL of a 0.1 M KF solution in (4:1) MeCN (3.2 mL): HFIP (0.8 mL). An electrasyn cap was prepared with 2 platinum plates (10 mm x 10 mm x 0.1 mm) and a rubber septa was used to cover the top cap (Figure S1). The cap was then twisted onto the vial, ensuring the electrodes aren't touching and are submerged in the solution. The solution was then purged with nitrogen for 5 minutes then was replaced with a N_2 balloon. This vial was equipped to an Electrasyn and the following conditions (20 mA, 0.5 Hz, 8 F/mol). Once the reaction was finished, 8.4 mg of 1,3,5-trimethoxybenzene was added as NMR internal standard. The crude solution was diluted with 25 mL ethyl acetate and washed with 10 mL saturated $NaHCO_3$ and 10 mL brine. The organic layer was collected and dried by $MgSO_4$. The organic phase was then concentrated under vacuum and the yield was determined by ^{1}H NMR (85%). The crude product was purified by passing through a silica gel column (EA/hexane 10% - 60%). The product collected after column was then dried on vacuum line overnight to afford yellow oil (80% yield). The NMR data matches literature.[11]

Dimethyl (4-fluorophenyl)phosphonate (2'). ^{1}H NMR (500 MHz, $CDCl_3$) δ 7.82 (dddd, J = 12.7, 8.8, 5.7, 1.4 Hz, 1H), 7.17 (tdd, J = 8.6, 3.4, 1.2 Hz, 1H), 3.76 (dd, J = 11.2, 1.3 Hz, 3H). ^{13}C NMR (126 MHz, $CDCl_3$) δ 165.7 (dd, J = 254.0, 3.9 Hz), 134.7 (dd, J = 11.4, 8.9 Hz), 123.2 (dd, J = 193.7, 3.5 Hz), 116.1 (dd, J = 21.5, 16.4 Hz), 52.9 (d, J = 5.6 Hz). ^{19}F NMR (565 MHz, $CDCl_3$) δ -105.44 (td, J = 8.8, 4.3 Hz, 1F). ^{31}P NMR (243 MHz, $CDCl_3$) δ 20.65.

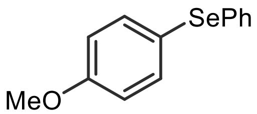

24

A 4 mL Electrasyn vial was charged with boronic acid (21.3 mg, 0.14 mmol, 1 eq.), potassium fluoride (23 mg, 0.4 mmol, 2.9 eq.) and diphenyl diselenide (0.16 mL, 1.4 mmol, 10 eq.). A stir bar was added and the threads were wrapped with parafilm to ensure an airtight seal when cap added. 4 mL of a 0.1 M KF solution in (4:1) MeCN (3.2 mL): HFIP (0.8 mL). An electrasyn cap was prepared with 2 platinum plates (10 mm x 10 mm x 0.1 mm) and a rubber septa was used to cover the top cap (Figure S1). The cap was then twisted onto the vial, ensuring the electrodes aren't

touching and are submerged in the solution. The solution was then purged with nitrogen for 5 minutes then was replaced with a N_2 balloon. This vial was equipped to an Electrasyn and the following conditions (20 mA, 0.5 Hz, 8 F/mol). Once the reaction was finished, 8.4 mg of 1,3,5-trimethoxybenzene was added as NMR internal standard. The crude solution was diluted with 25 mL ethyl acetate and washed with 10 mL saturated $NaHCO_3$ and 10 mL brine. The organic layer was collected and dried by $MgSO_4$. The crude product was purified by passing through a silica gel column (EA/hexane 0-10%). The product collected after column was then dried on vacuum line overnight to afford yellow oil (87% yield). The NMR data matches literature.[5]

(4-Methoxyphenyl)(phenyl)selane (**23**). ^{1}H NMR (500 MHz, $CDCl_3$) δ 7.44 – 7.39 (m, 1H), 7.27 – 7.21 (m, 1H), 7.17 – 7.06 (m, 2H), 6.77 (d, *J* = 8.4 Hz, 1H), 3.71 (s, 2H). ^{13}C NMR (126 MHz, $CDCl_3$) δ 159.9, 136.7, 133.4, 131.1, 129.3, 126.6, 120.1, 115.3, 55.4.

9. NMR spectra

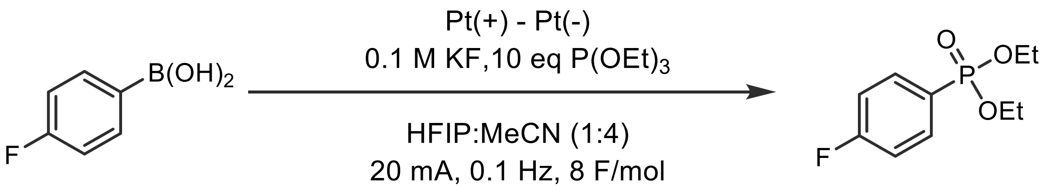
B(OH)$_2$
F
Pt(+) - Pt(-)
0.1 M KF,10 eq P(OEt)$_3$
HFIP:MeCN (1:4)
20 mA, 0.1 Hz, 8 F/mol
O
P
OEt
OEt
F

89% NMR yield

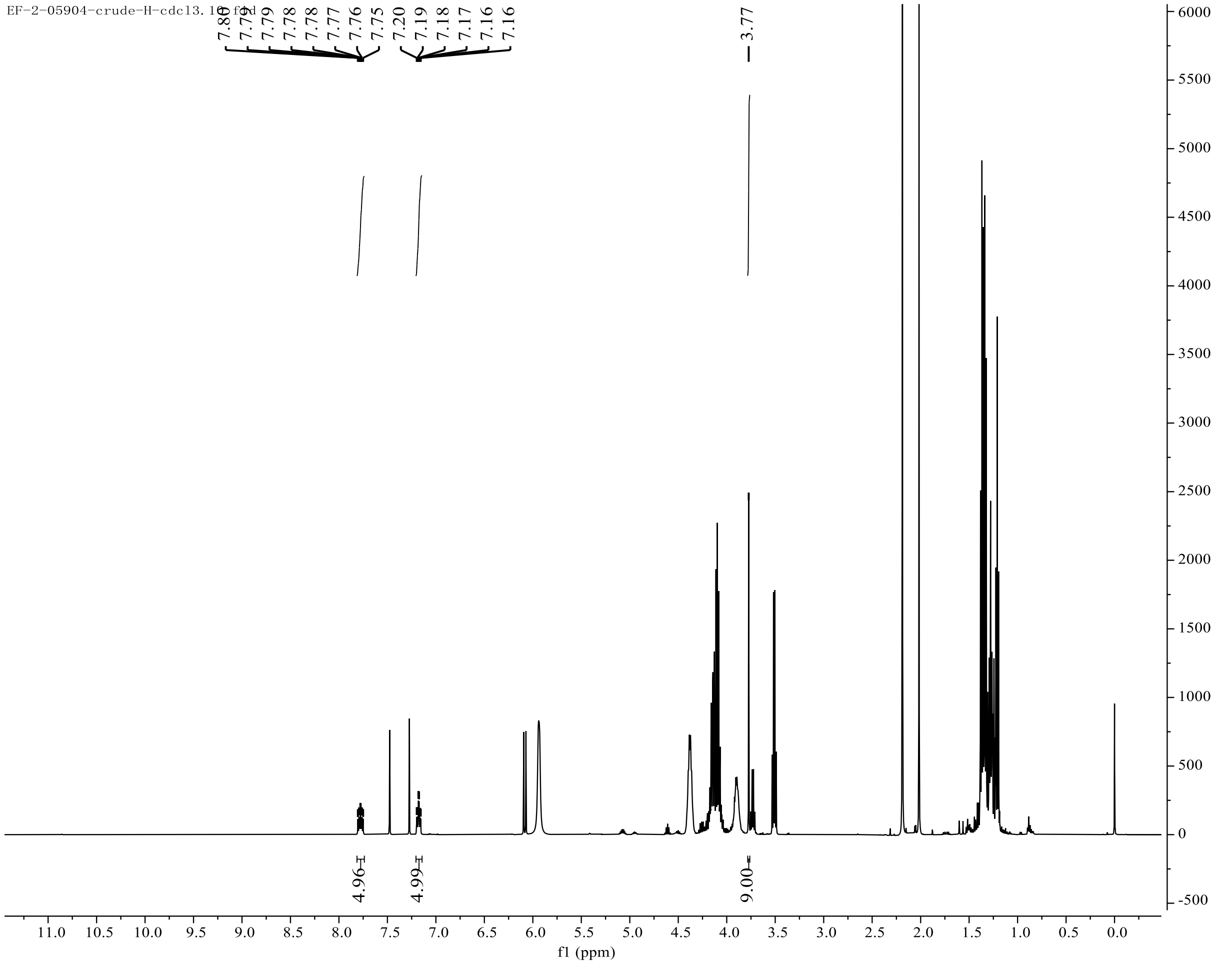
EF-2-05904-crude-H-cdcl3.10.fid
7.80
7.79
7.79
7.78
7.78
7.77
7.76
7.75
7.20
7.19
7.18
7.17
7.16
7.16
3.77
4.96
4.99
9.00
f1 (ppm)

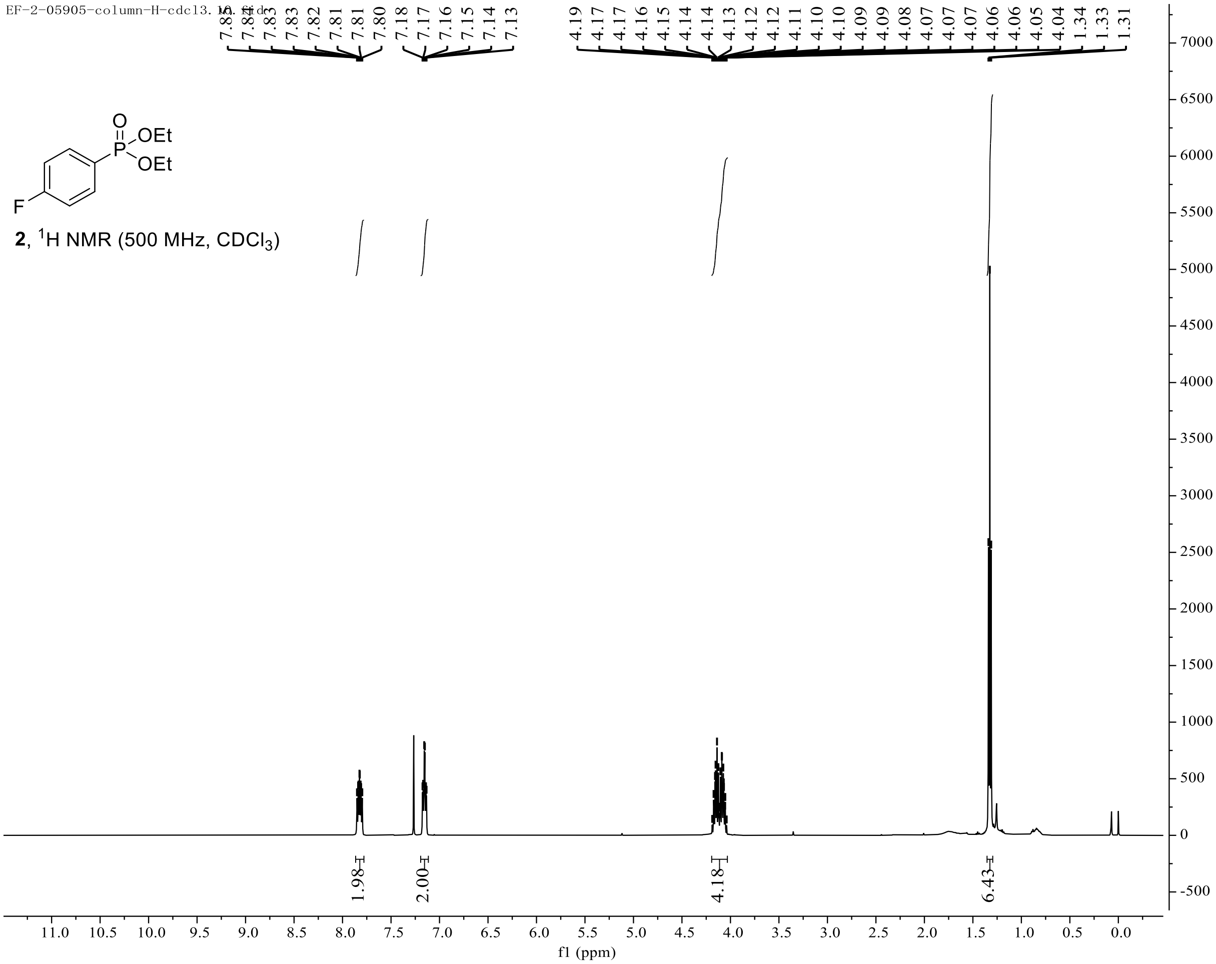
EF-2-05905-column-H-cdcl3.10.fid
2, ¹H NMR (500 MHz, CDCl3)
f1 (ppm)

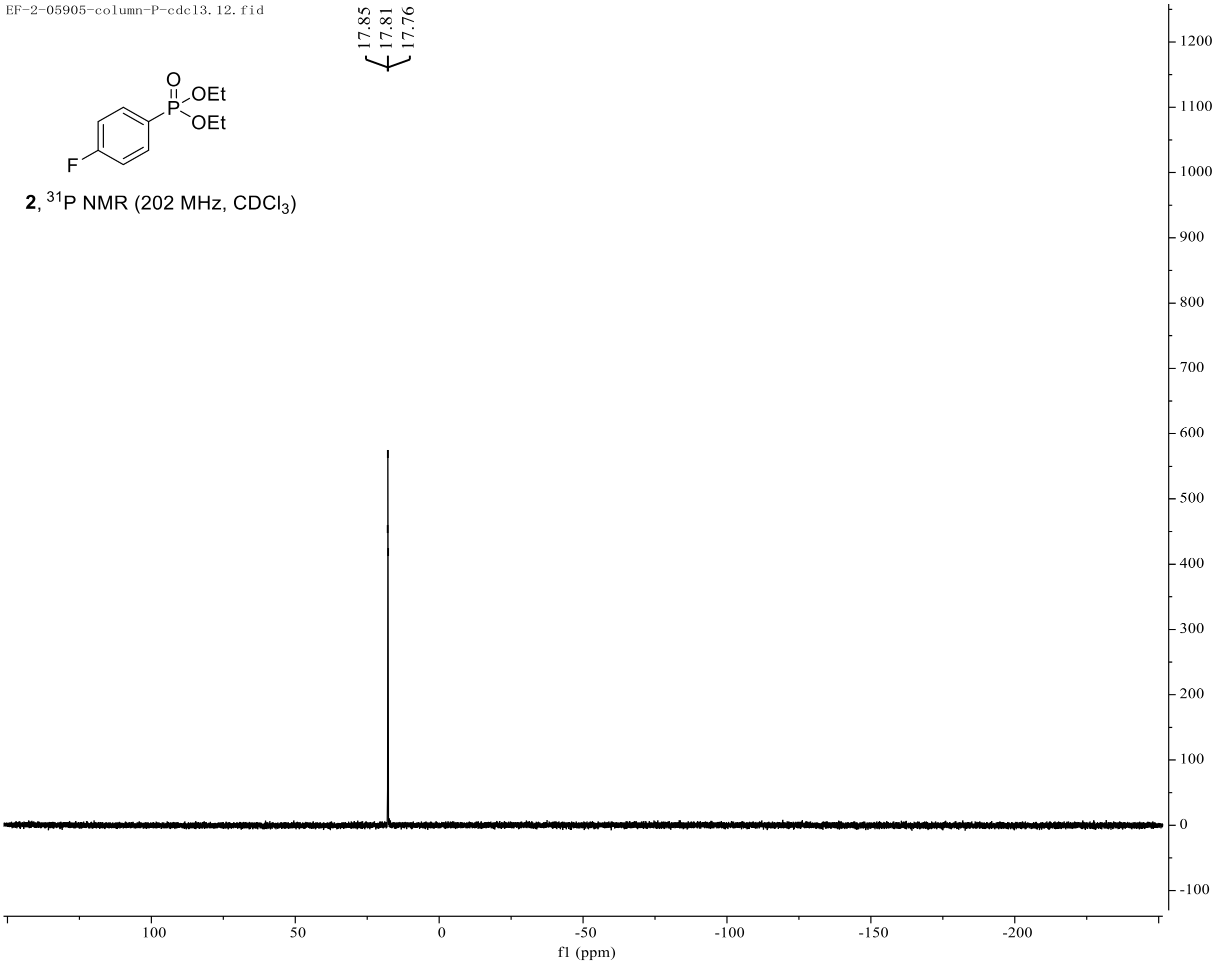

2, ^{31}P NMR (202 MHz, $CDCl_3$)

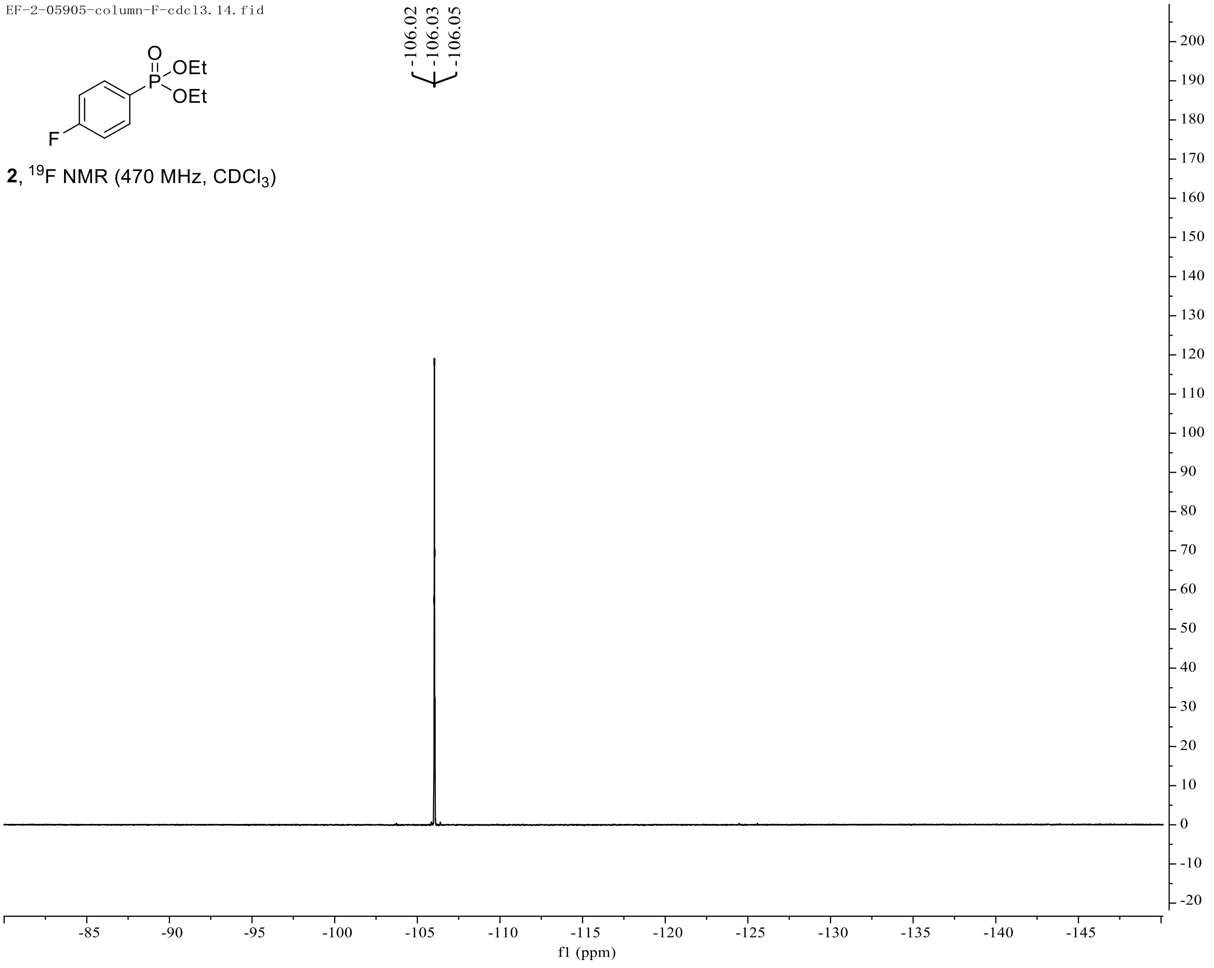
EF-2-05905-column-F-cdcl3.14.fid
O
OEt
P
OEt
F
2, 19F NMR (470 MHz, CDCl3)
-106.02
-106.03
-106.05
200
190
180
170
160
150
140
130
120
110
100
90
80
70
60
50
40
30
20
10
0
-10
-20
-85
-90
-95
-100
-105
-110
-115
-120
-125
-130
-135
-140
-145
f1 (ppm)

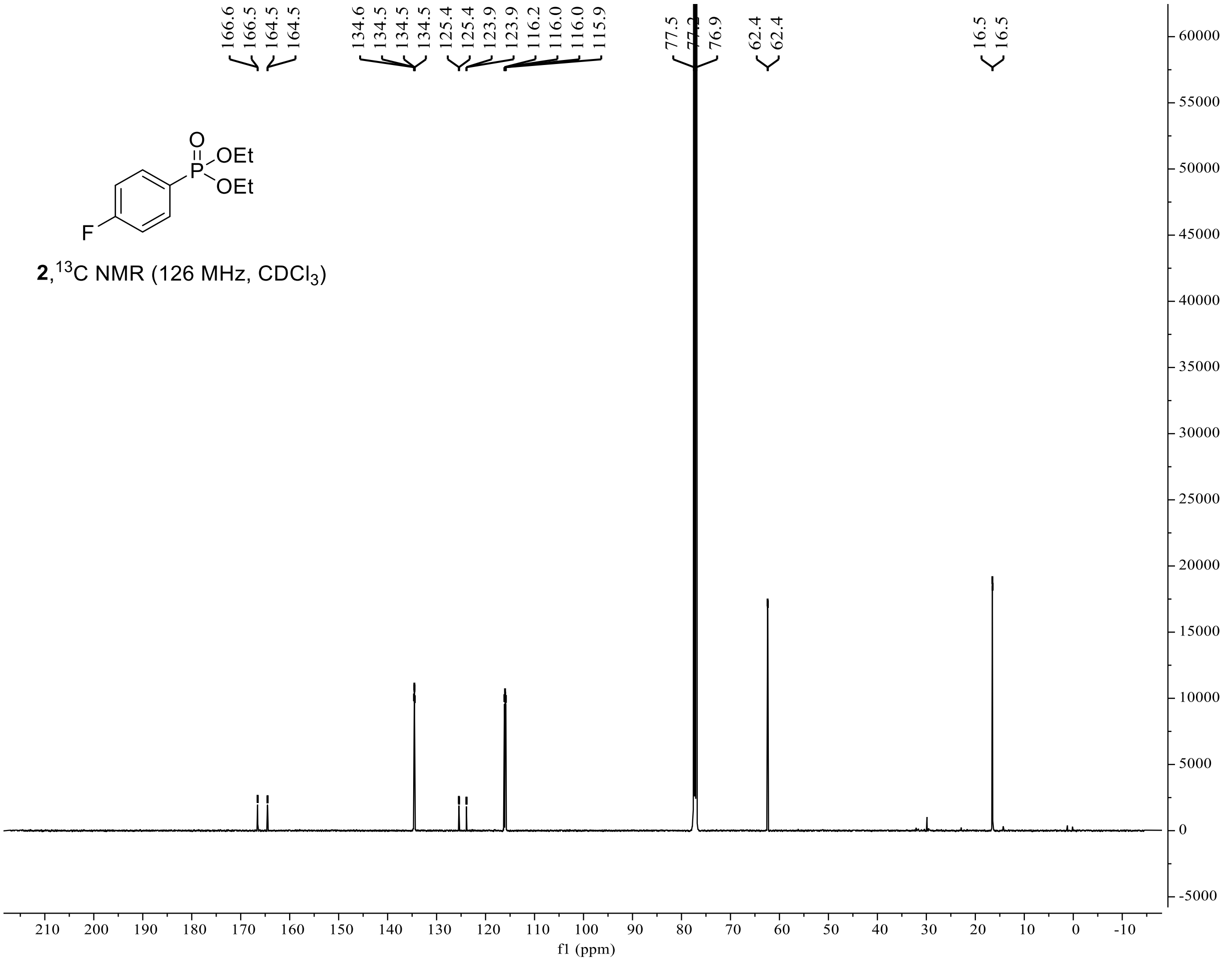

2, ^{13}C NMR (126 MHz, $CDCl_3$)

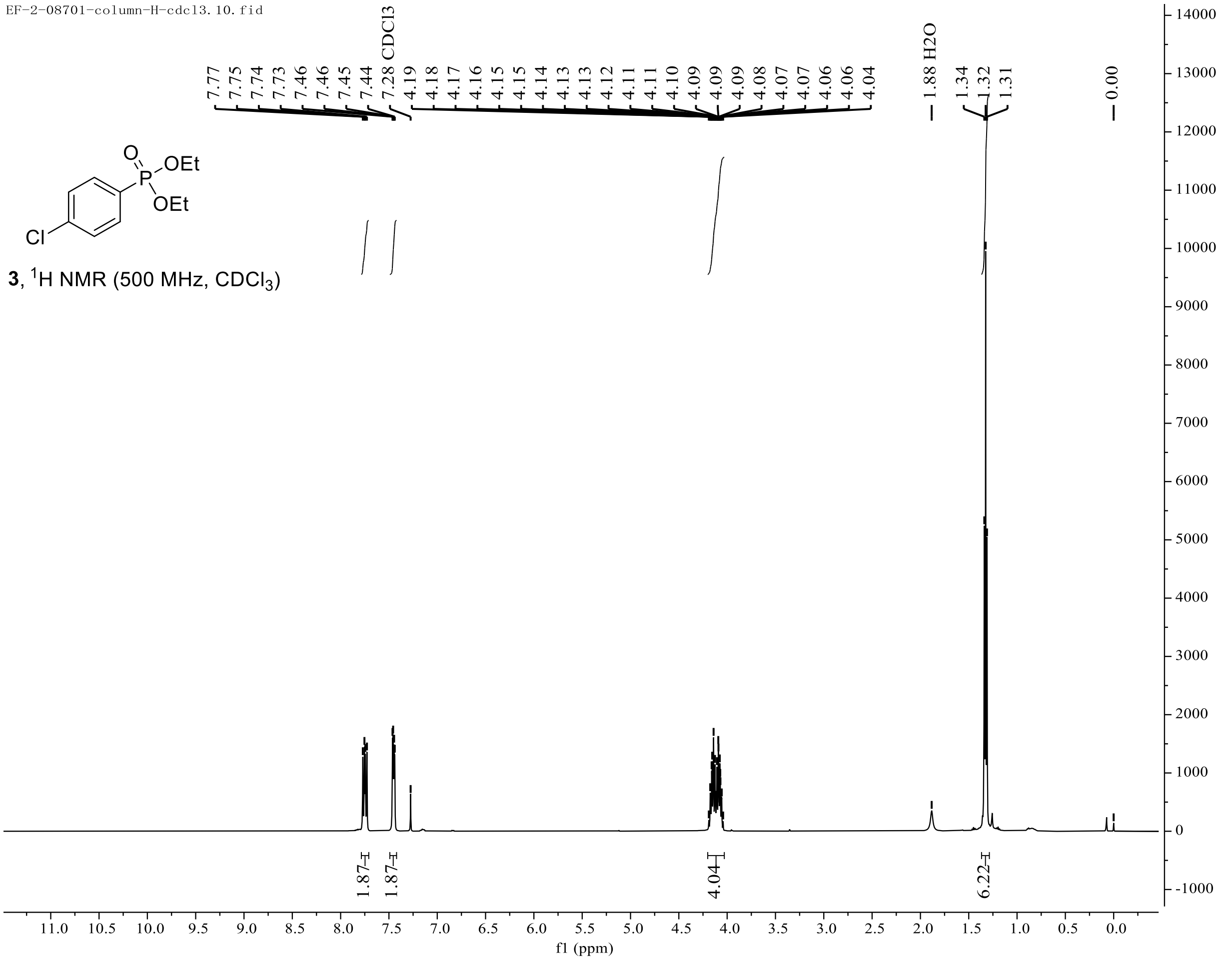

3, ^{1}H NMR (500 MHz, $CDCl_3$)

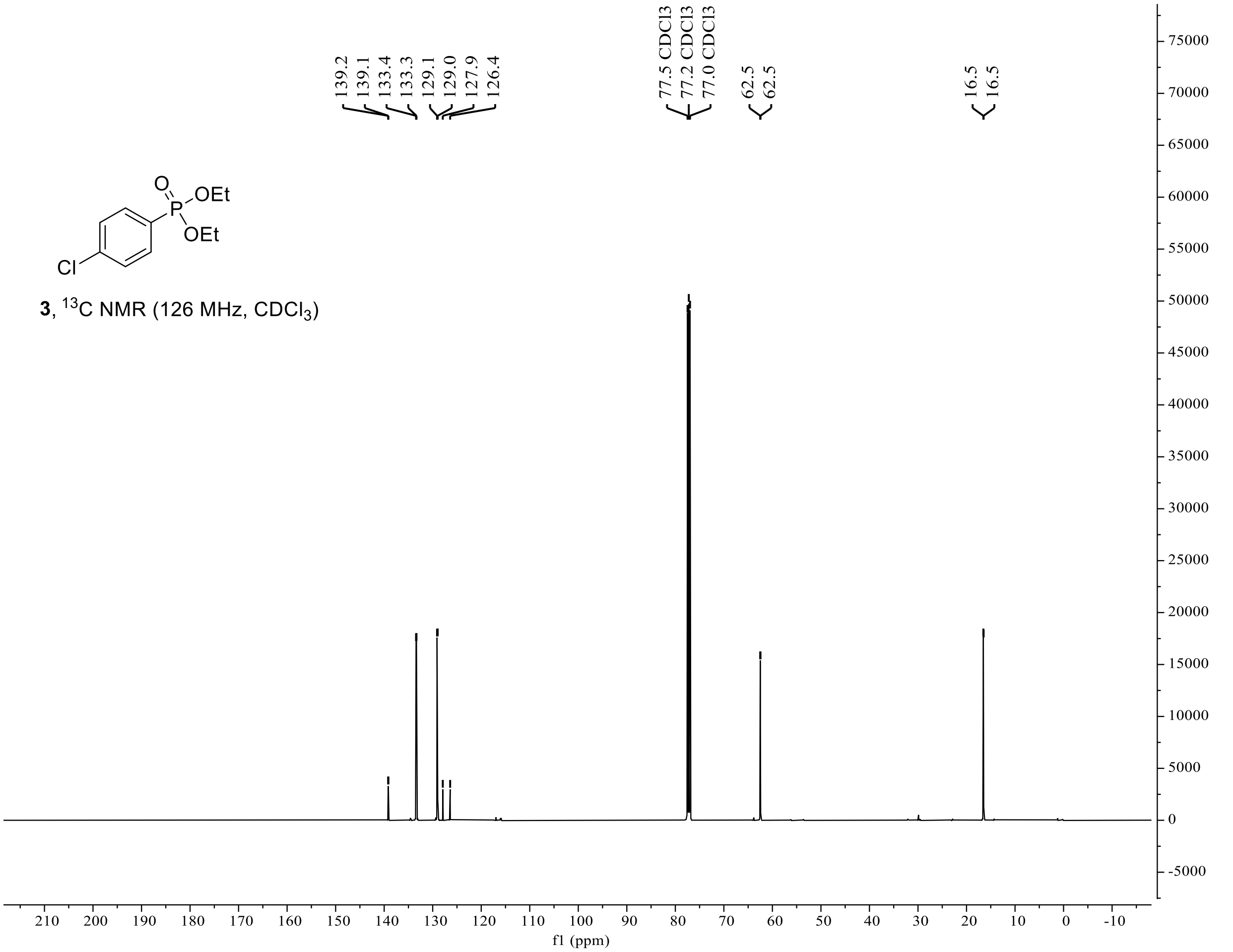

3, ^{13}C NMR (126 MHz, $CDCl_3$)

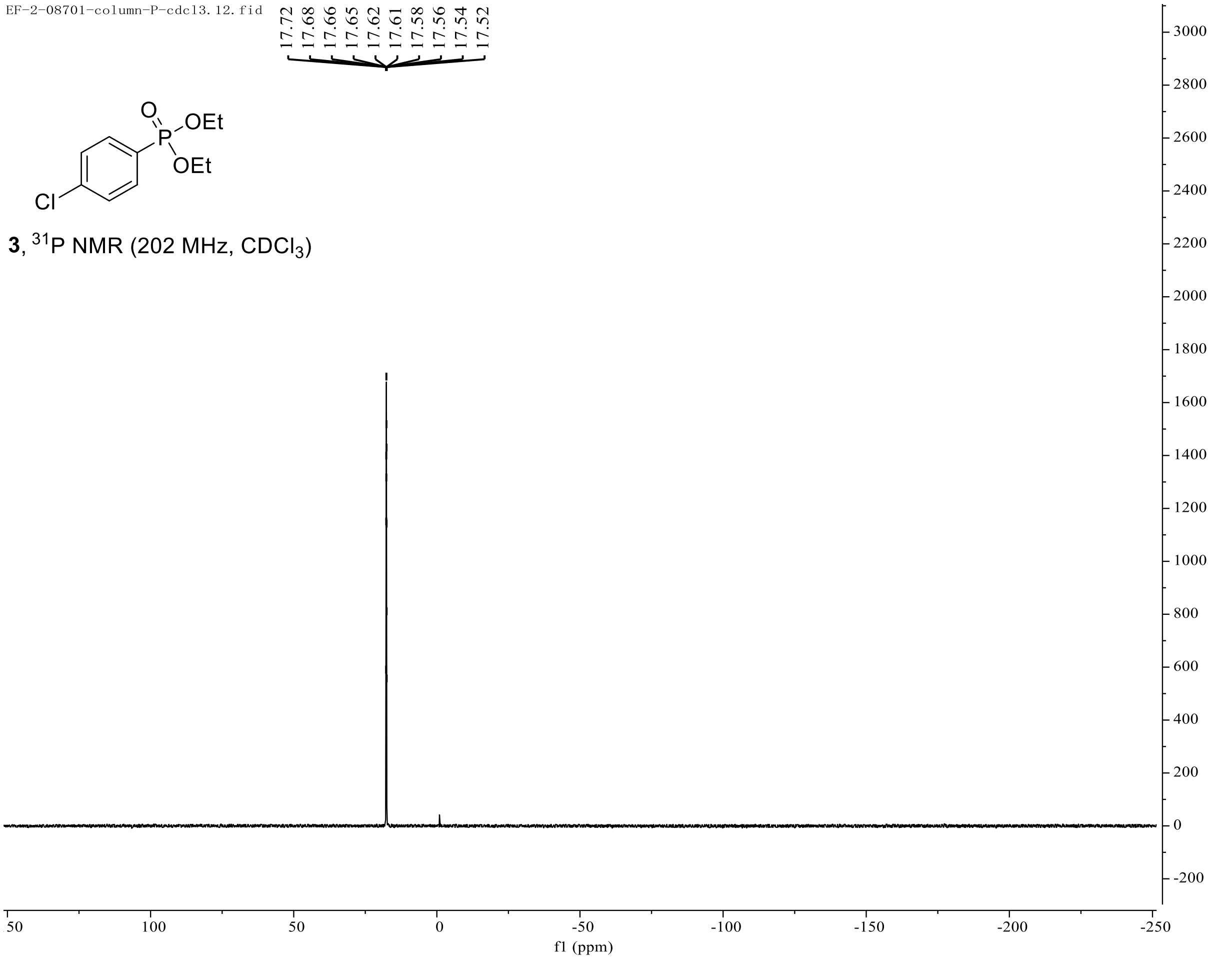

3, ^{31}P NMR (202 MHz, $CDCl_3$)

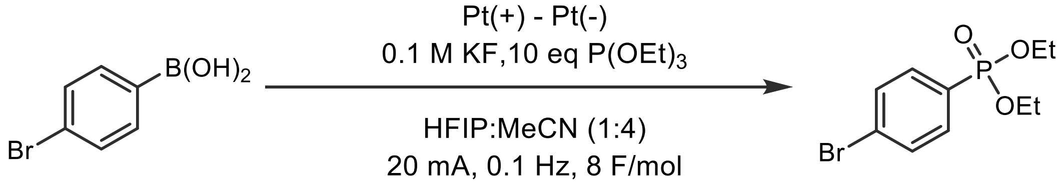
Br
B(OH)2
Pt(+) - Pt(-)
0.1 M KF,10 eq P(OEt)3
HFIP:MeCN (1:4)
20 mA, 0.1 Hz, 8 F/mol
O
P
OEt
OEt
Br

14% NMR yield

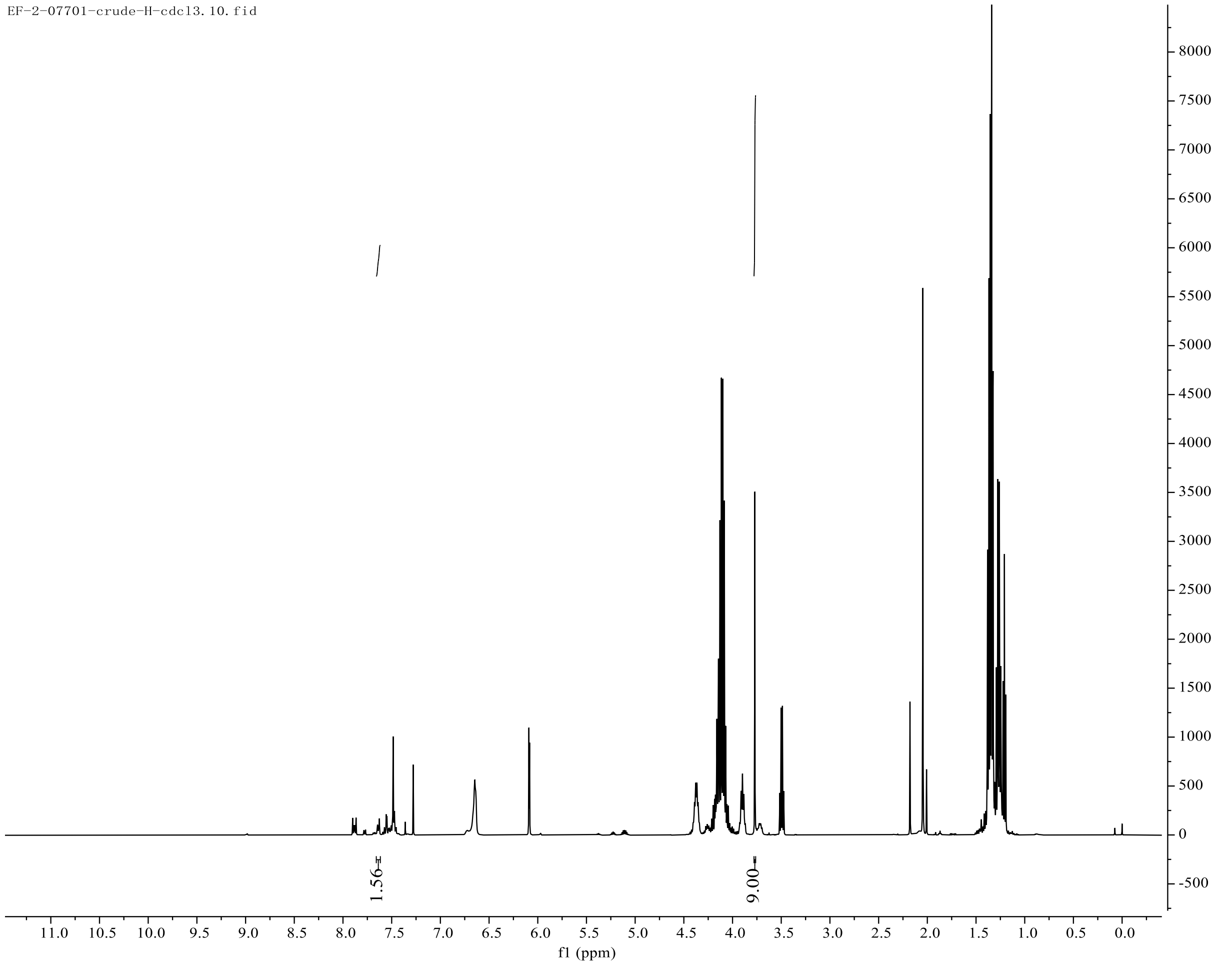
EF-2-07701-crude-H-cdcl3.10.fid
1.56
9.00
f1 (ppm)

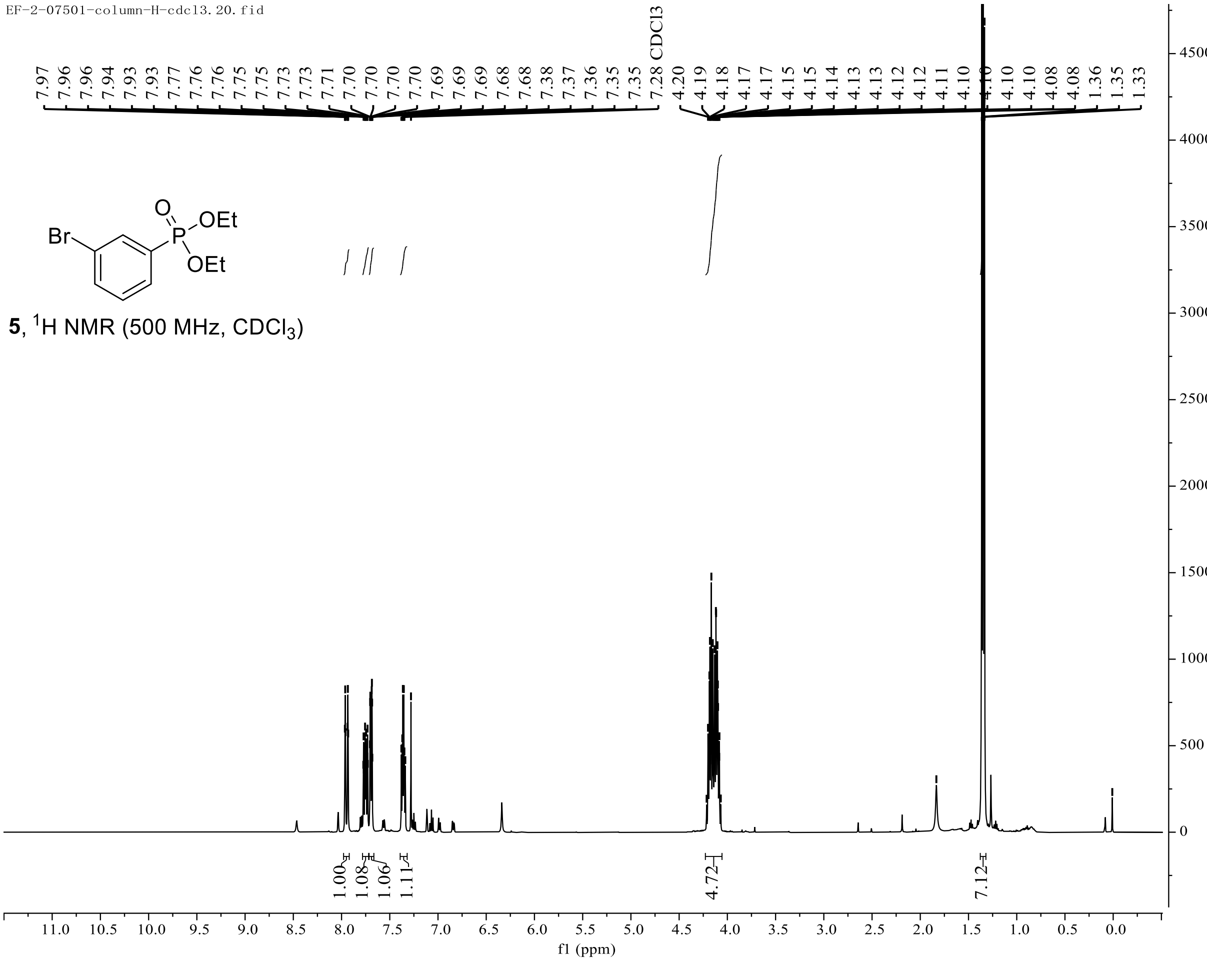

5, ^{1}H NMR (500 MHz, $CDCl_3$)

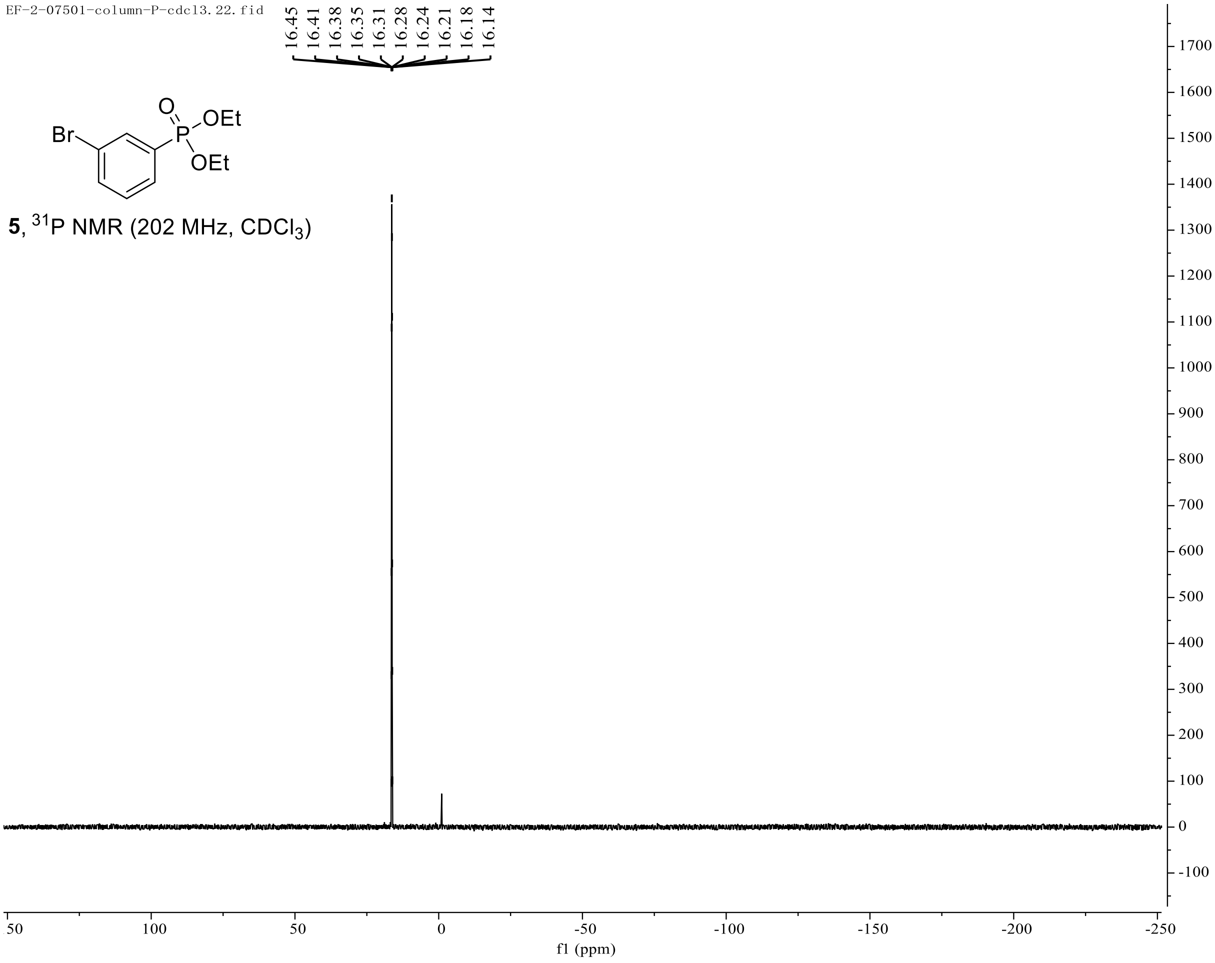

EF-2-07501-column-P-cdcl3.22.fid
16.45
16.41
16.38
16.35
16.31
16.28
16.24
16.21
16.18
16.14
Br
O
P
OEt
OEt
5, ^{31}P NMR (202 MHz, CDCl$_3$)
f1 (ppm)

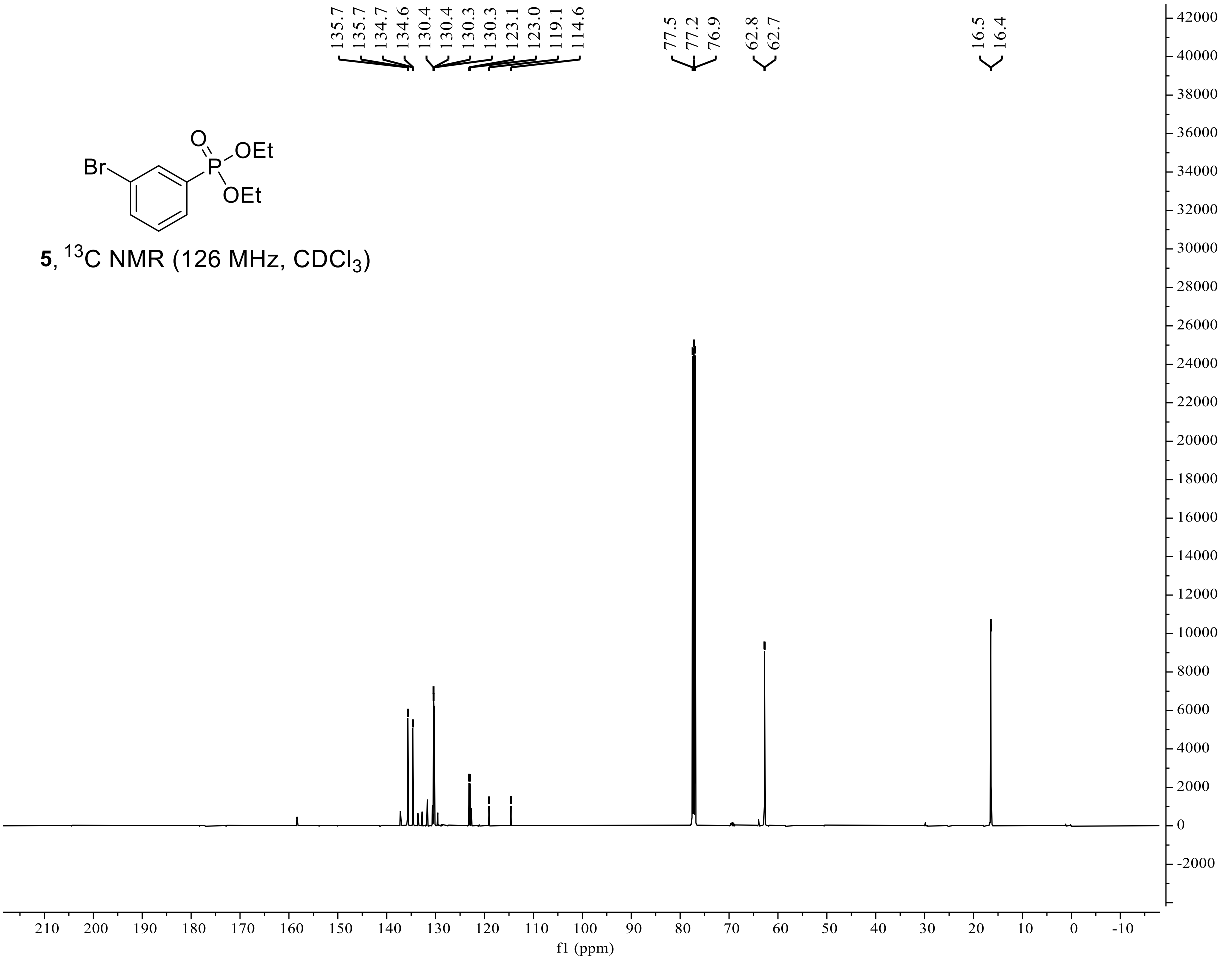

5, ^{13}C NMR (126 MHz, $CDCl_3$)

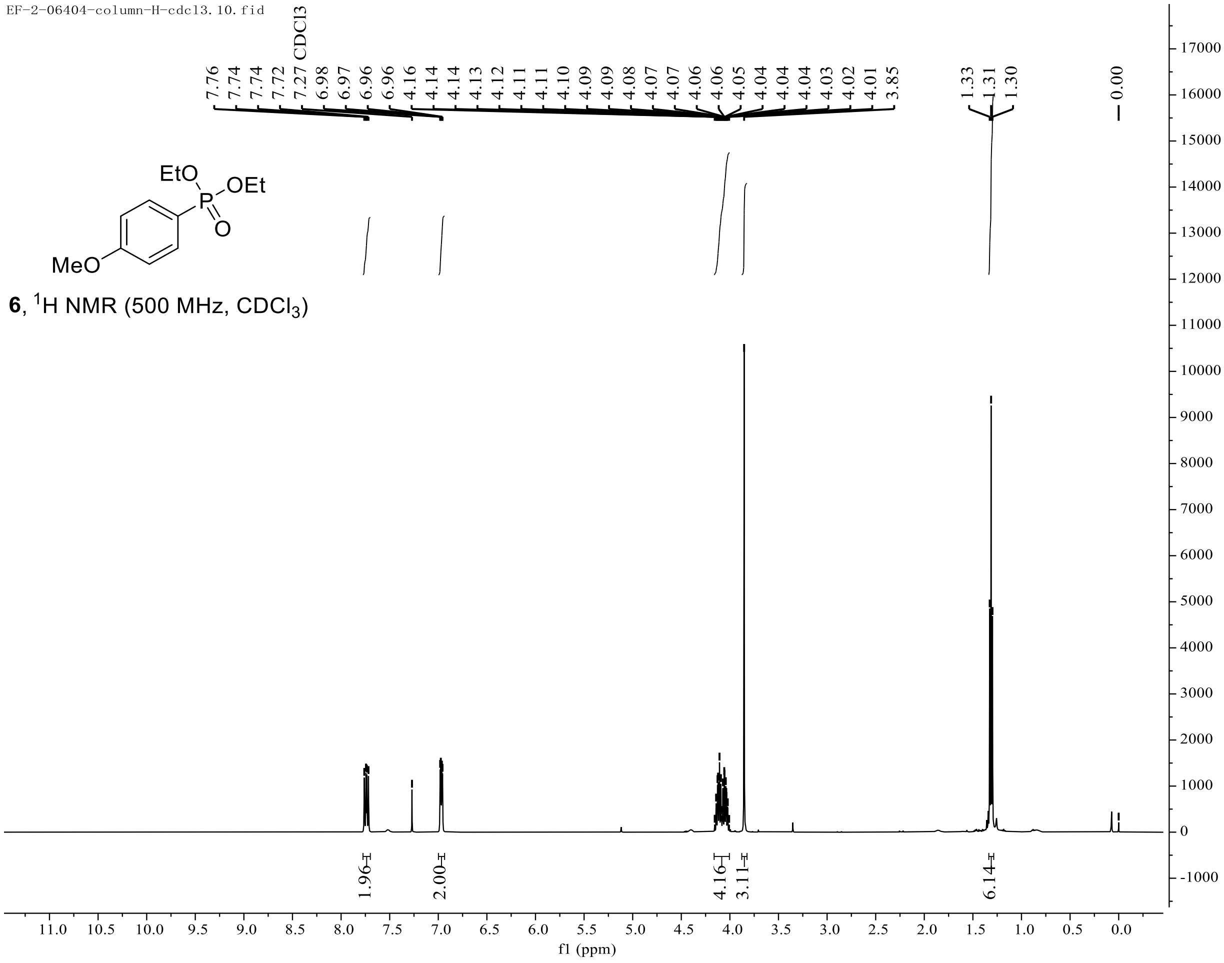

6, ^{1}H NMR (500 MHz, $CDCl_3$)

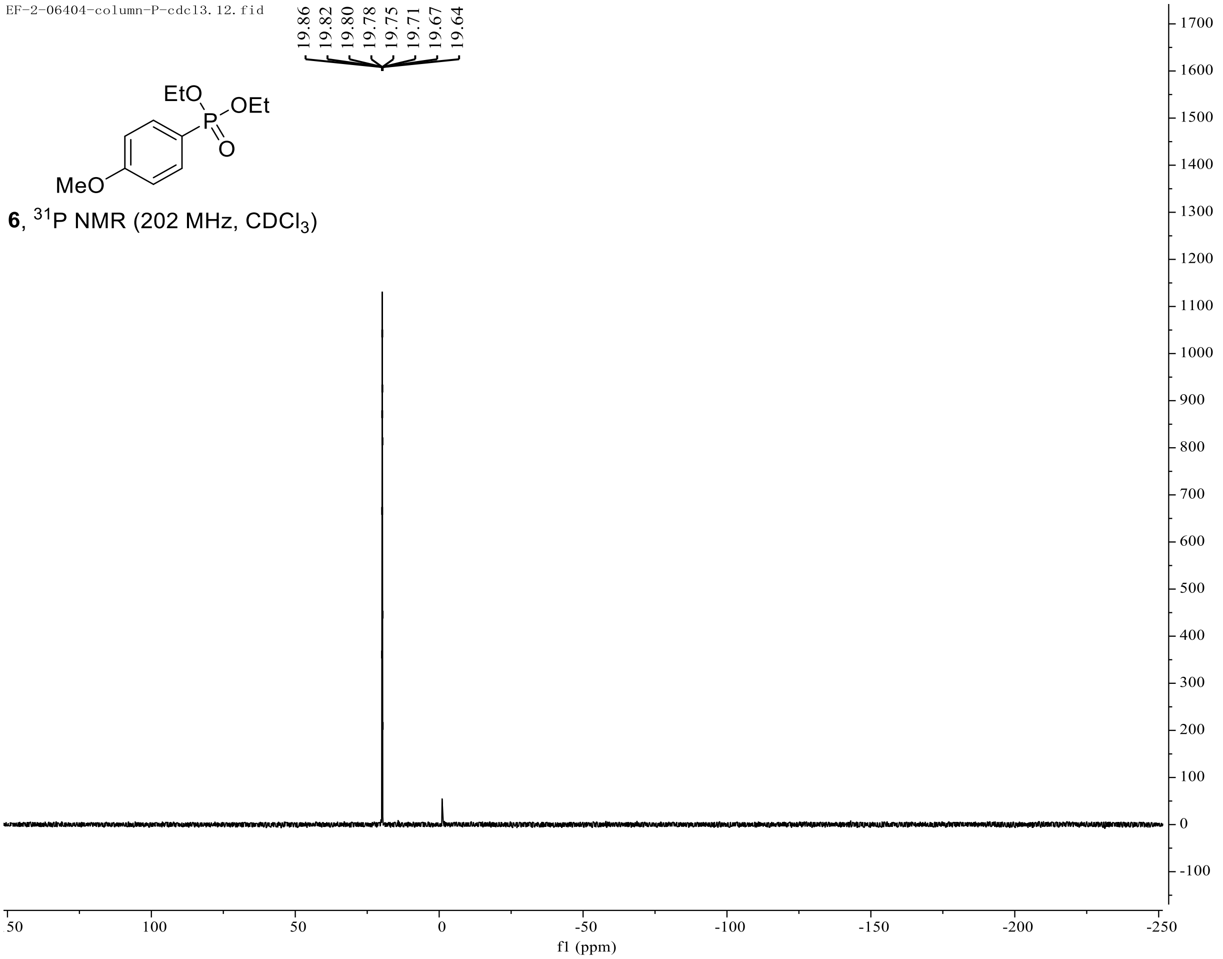

6, ^{31}P NMR (202 MHz, $CDCl_3$)

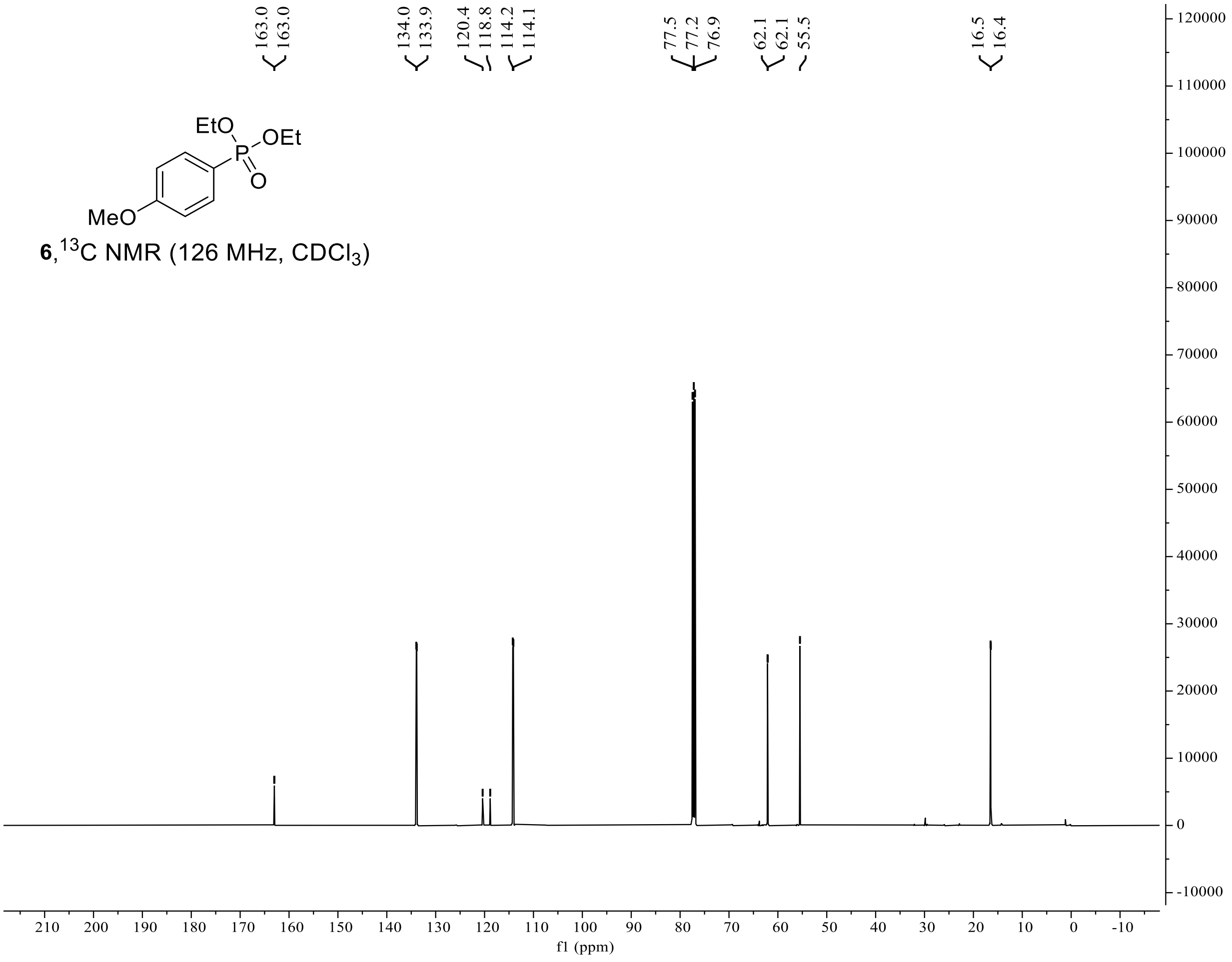

163.0
163.0
134.0
133.9
120.4
118.8
114.2
114.1
77.5
77.2
76.9
62.1
62.1
55.5
16.5
16.4
EtO
OEt
P
O
MeO
6, 13C NMR (126 MHz, CDCl3)
f1 (ppm)

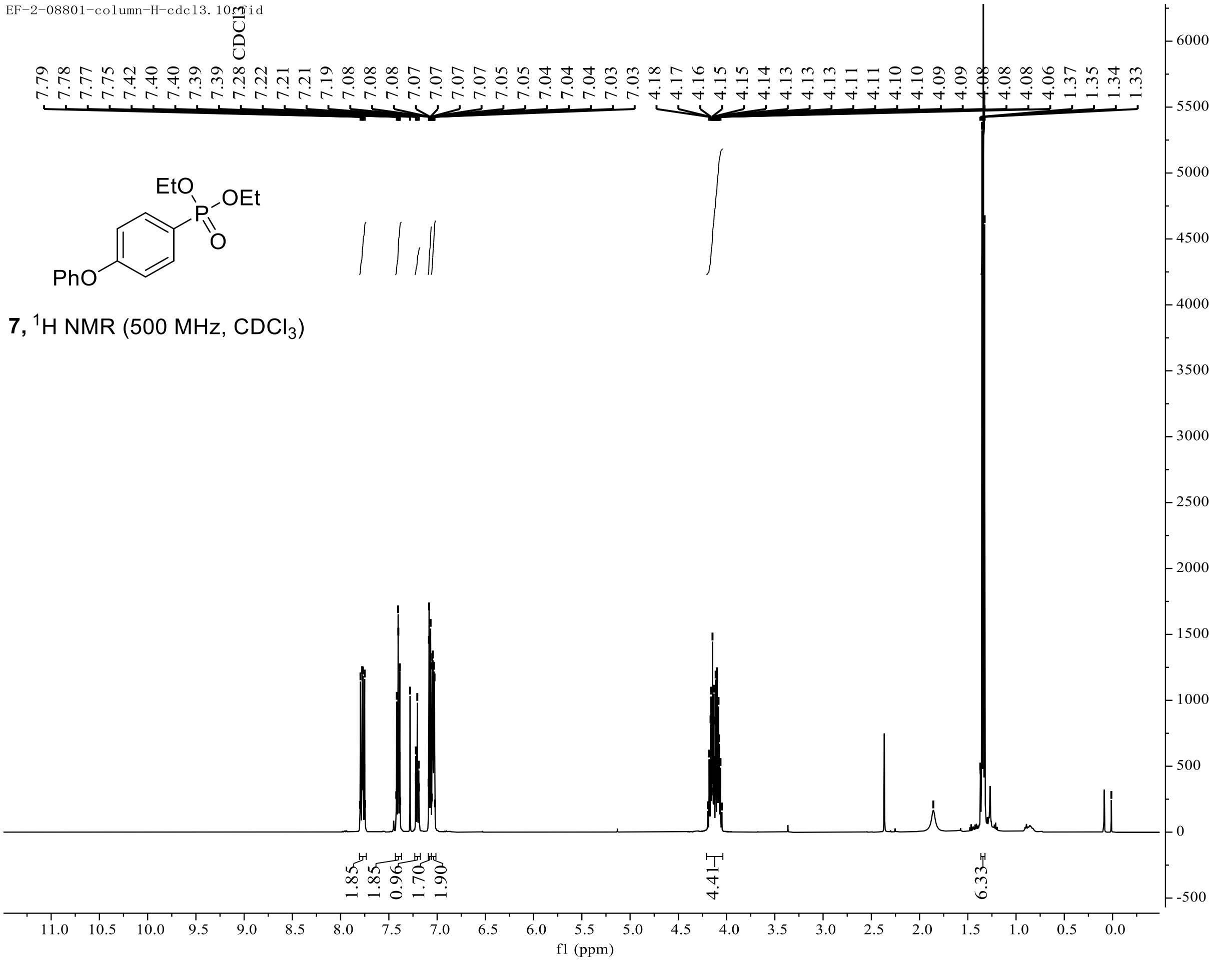
EF-2-08801-column-H-cdcl3.10.fid
EtO
P
OEt
O
PhO
7, ¹H NMR (500 MHz, CDCl3)
f1 (ppm)

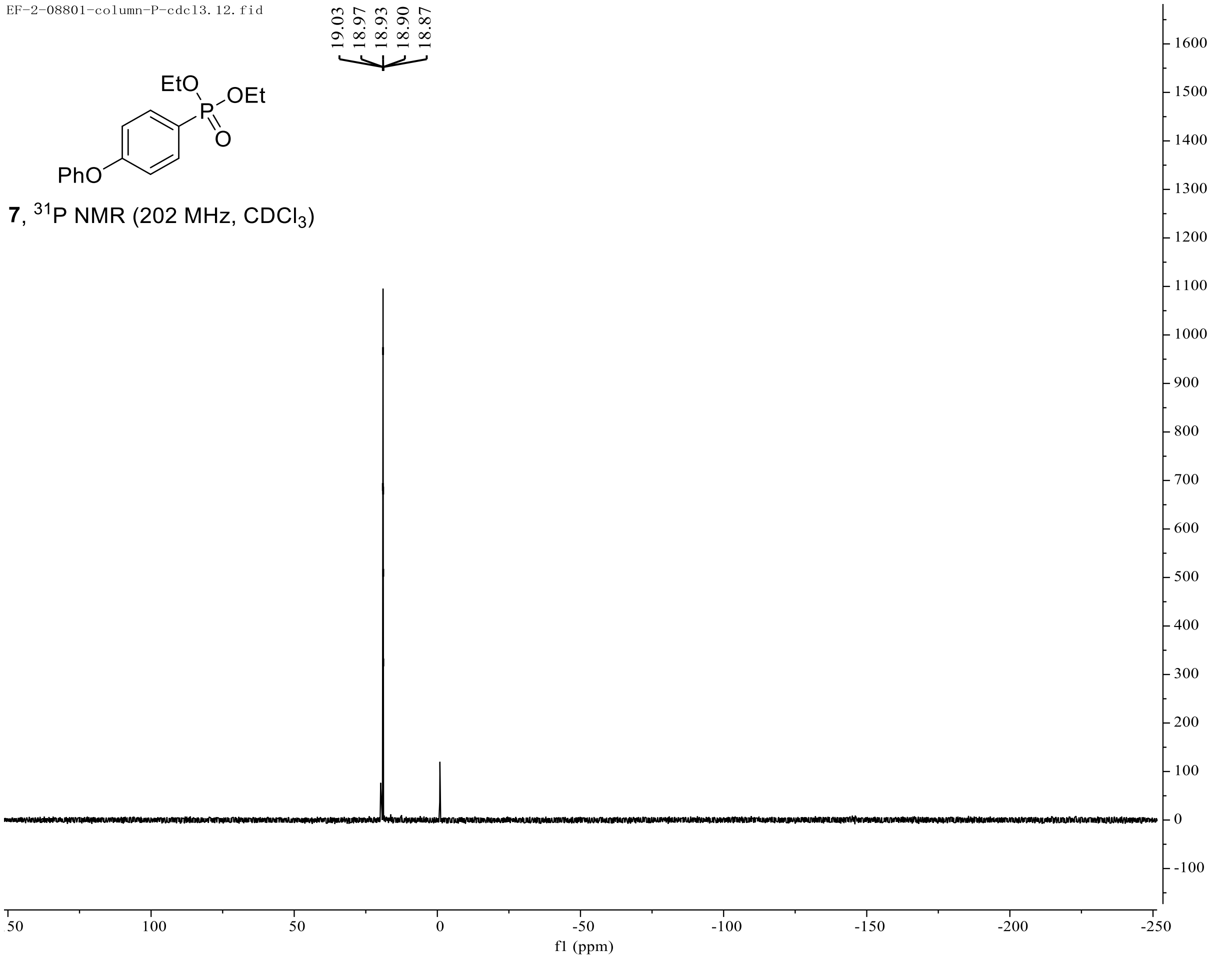

7, ^{31}P NMR (202 MHz, $CDCl_3$)

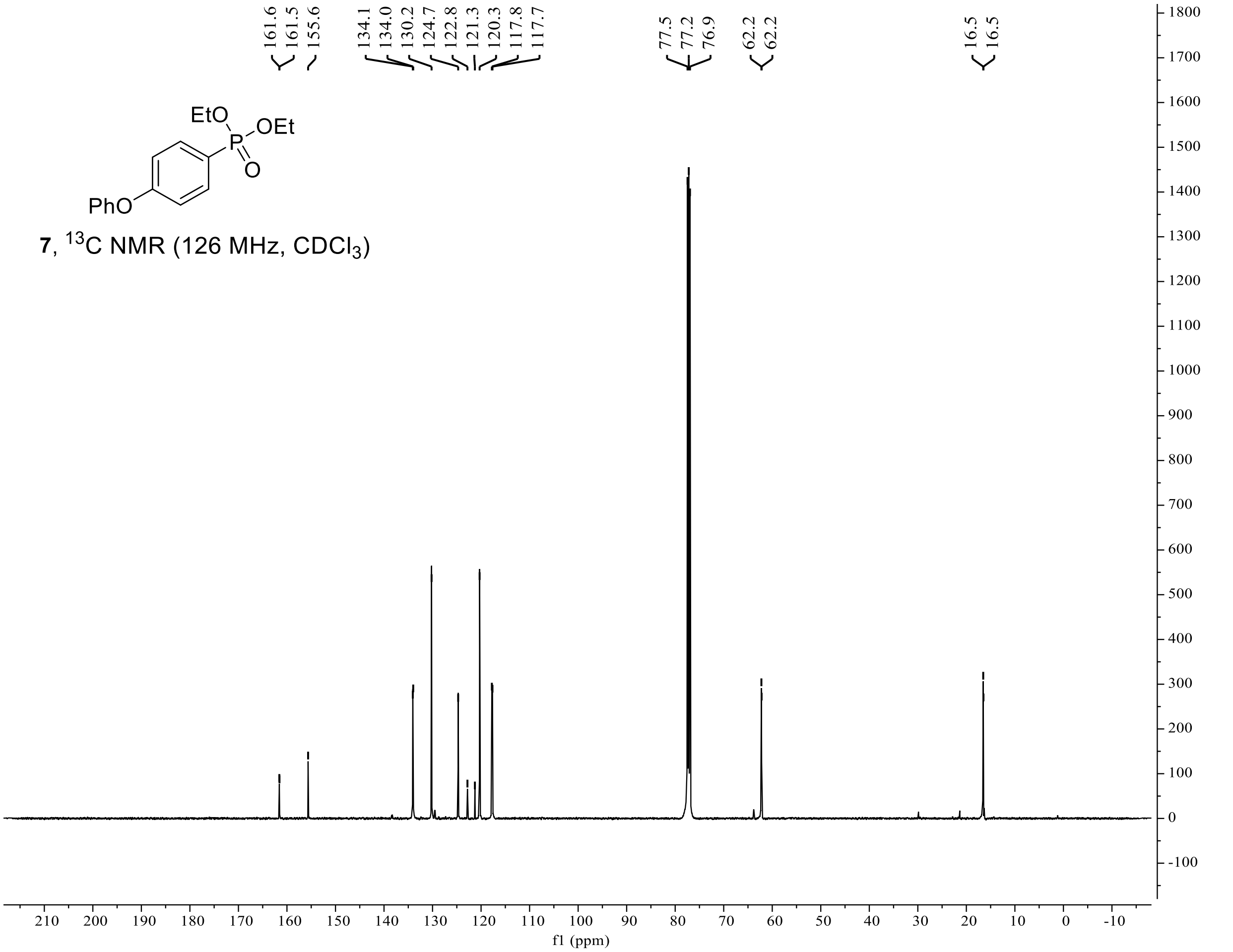
161.6
161.5
155.6
134.1
134.0
130.2
124.7
122.8
121.3
120.3
117.8
117.7
77.5
77.2
76.9
62.2
62.2
16.5
16.5
EtO
OEt
P
O
PhO
7, 13C NMR (126 MHz, CDCl3)
f1 (ppm)

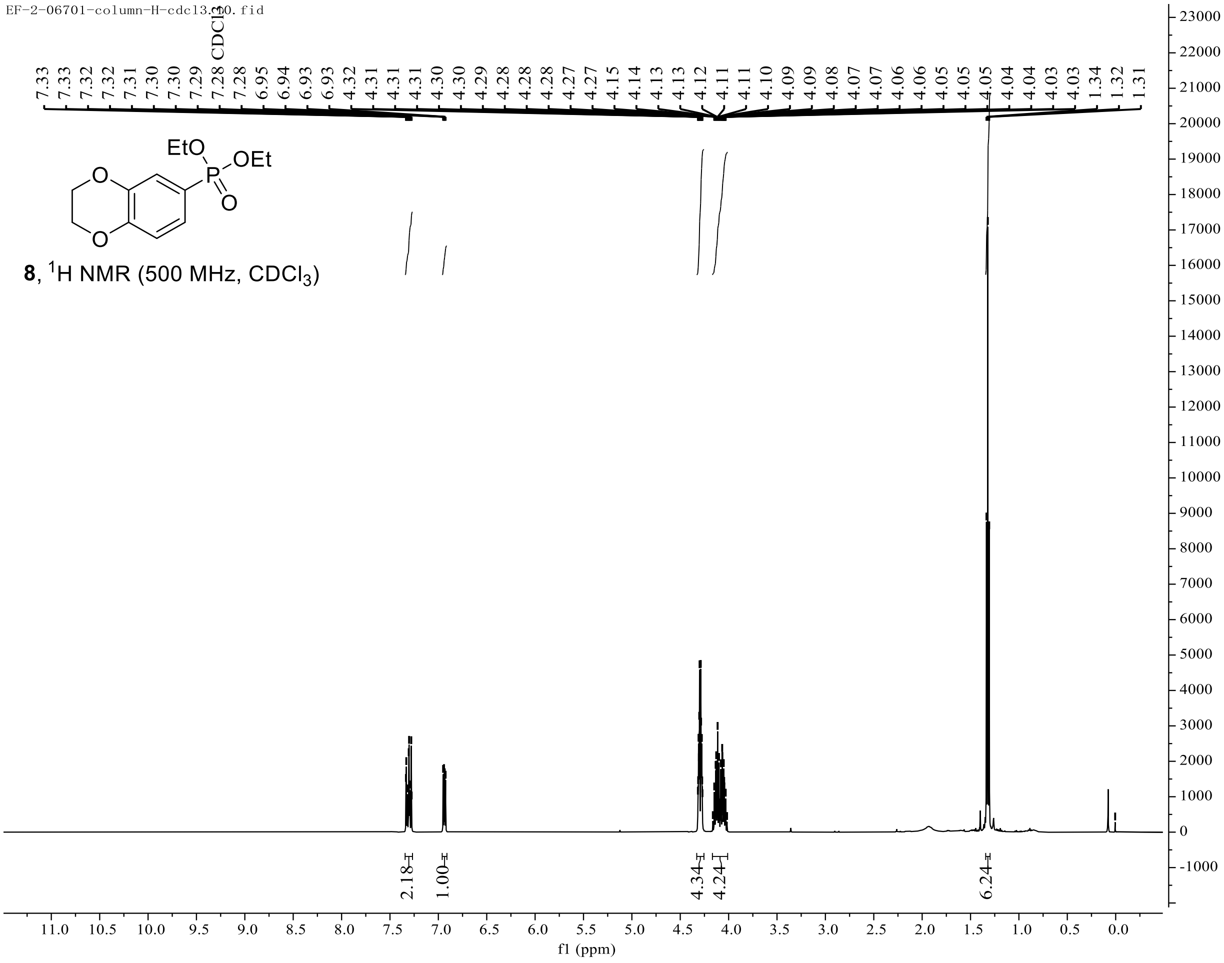

8, ^{1}H NMR (500 MHz, $CDCl_3$)

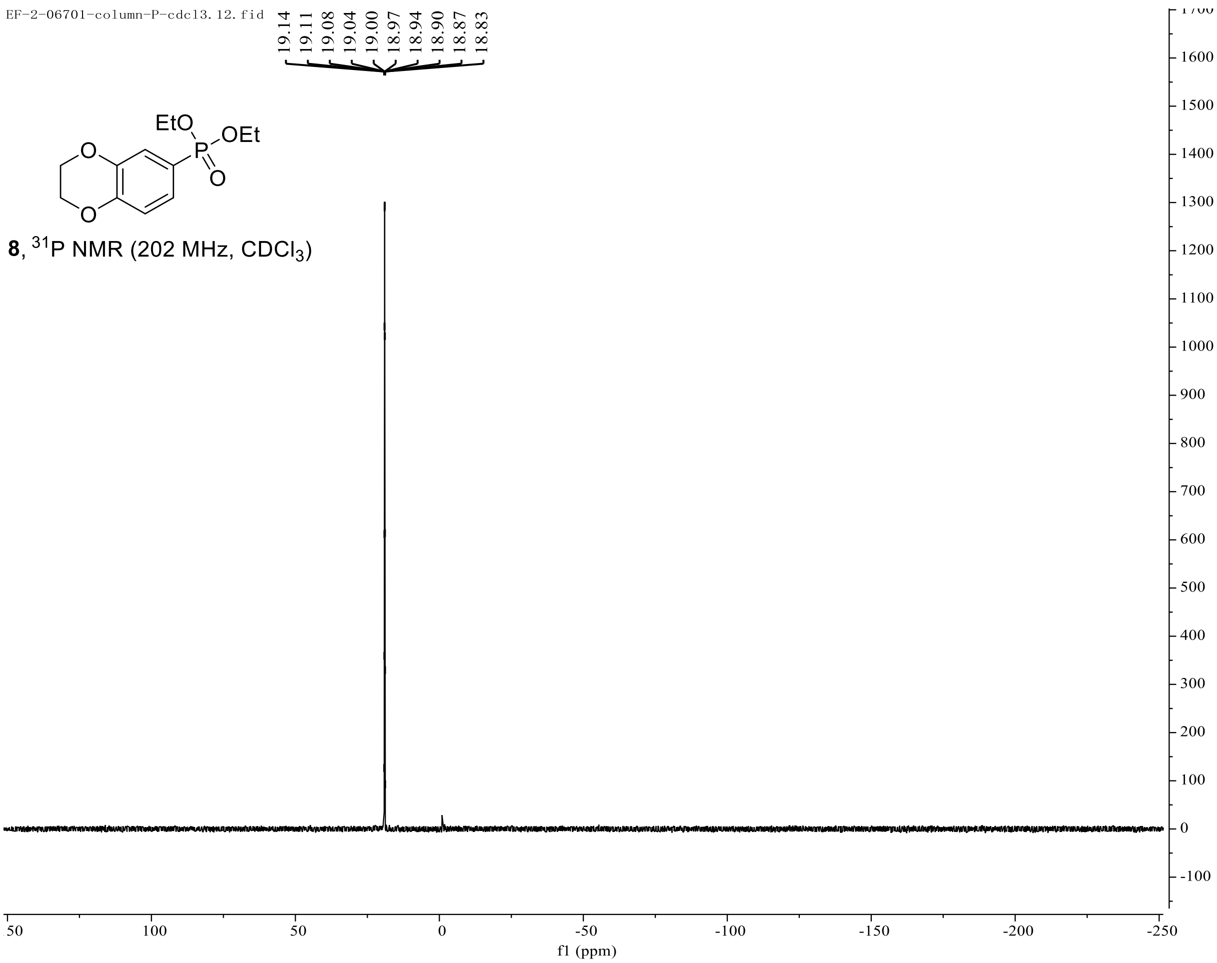
EF-2-06701-column-P-cdcl3.12.fid
19.14
19.11
19.08
19.04
19.00
18.97
18.94
18.90
18.87
18.83
EtO
OEt
P
O
O
O
8, ^{31}P NMR (202 MHz, $CDCl_3$)
f1 (ppm)

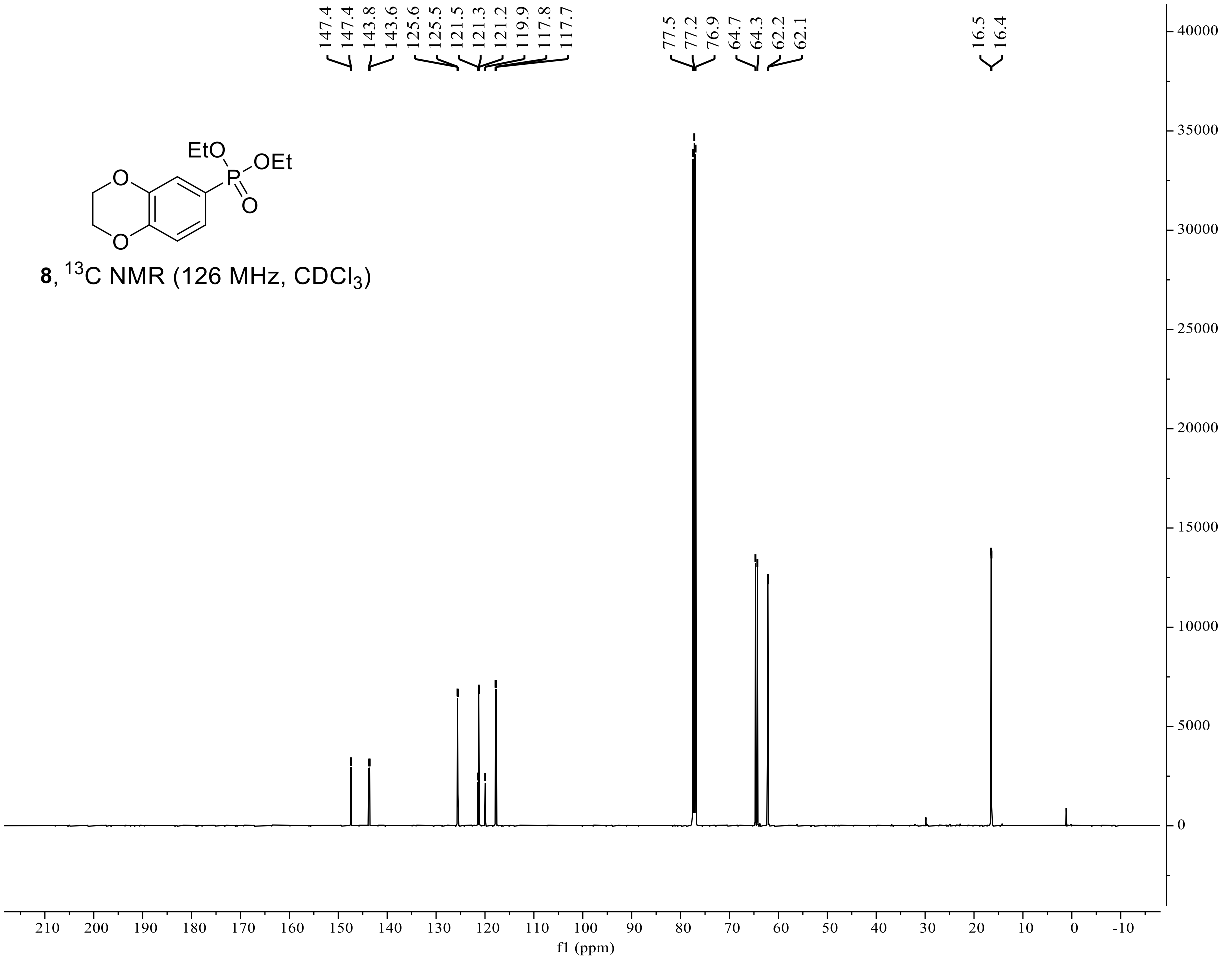

147.4
147.4
143.8
143.6
125.6
125.5
121.5
121.3
121.2
119.9
117.8
117.7
77.5
77.2
76.9
64.7
64.3
62.2
62.1
16.5
16.4
EtO
OEt
P
O
O
O
8, 13C NMR (126 MHz, CDCl3)
f1 (ppm)

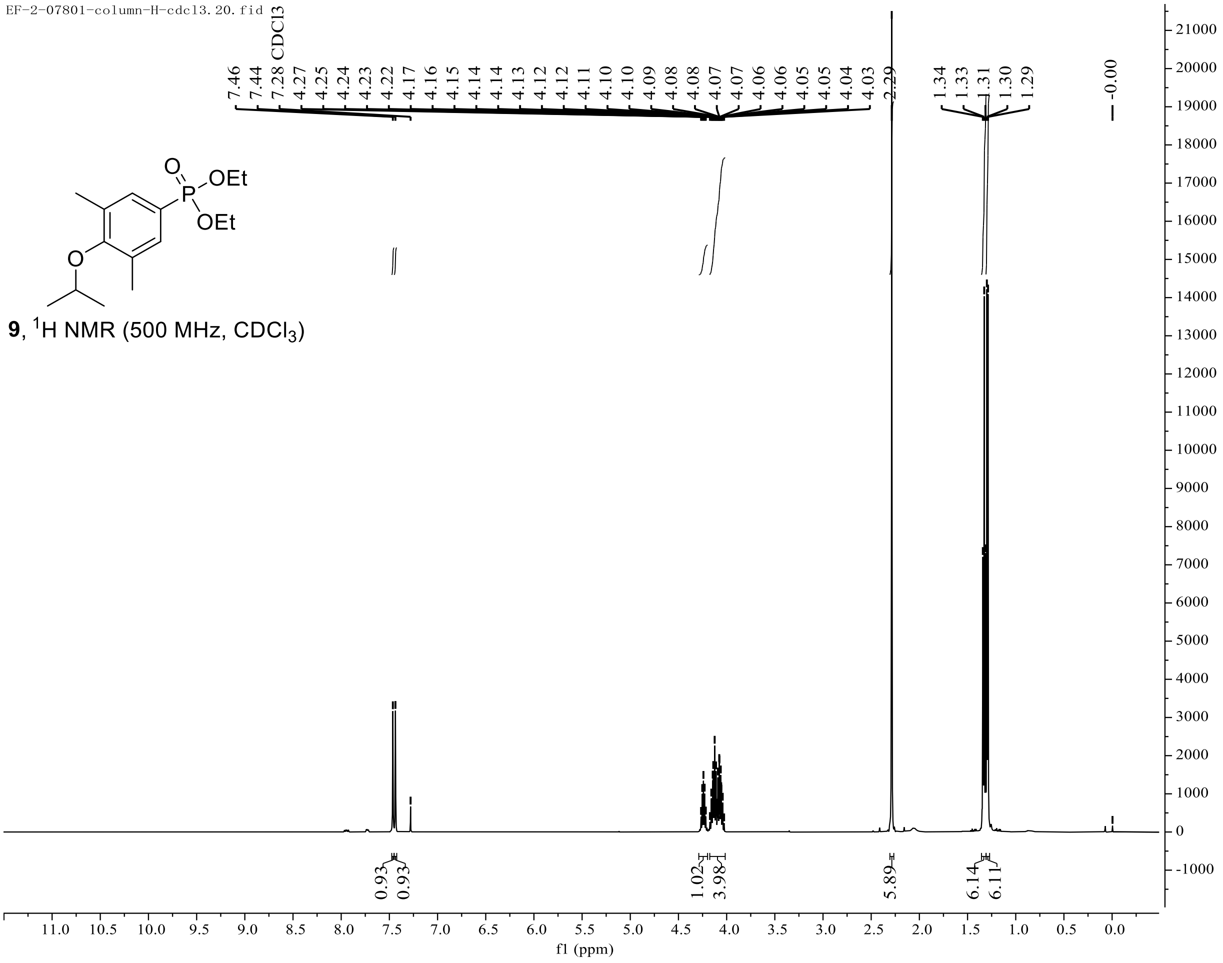

9, ^{1}H NMR (500 MHz, $CDCl_3$)

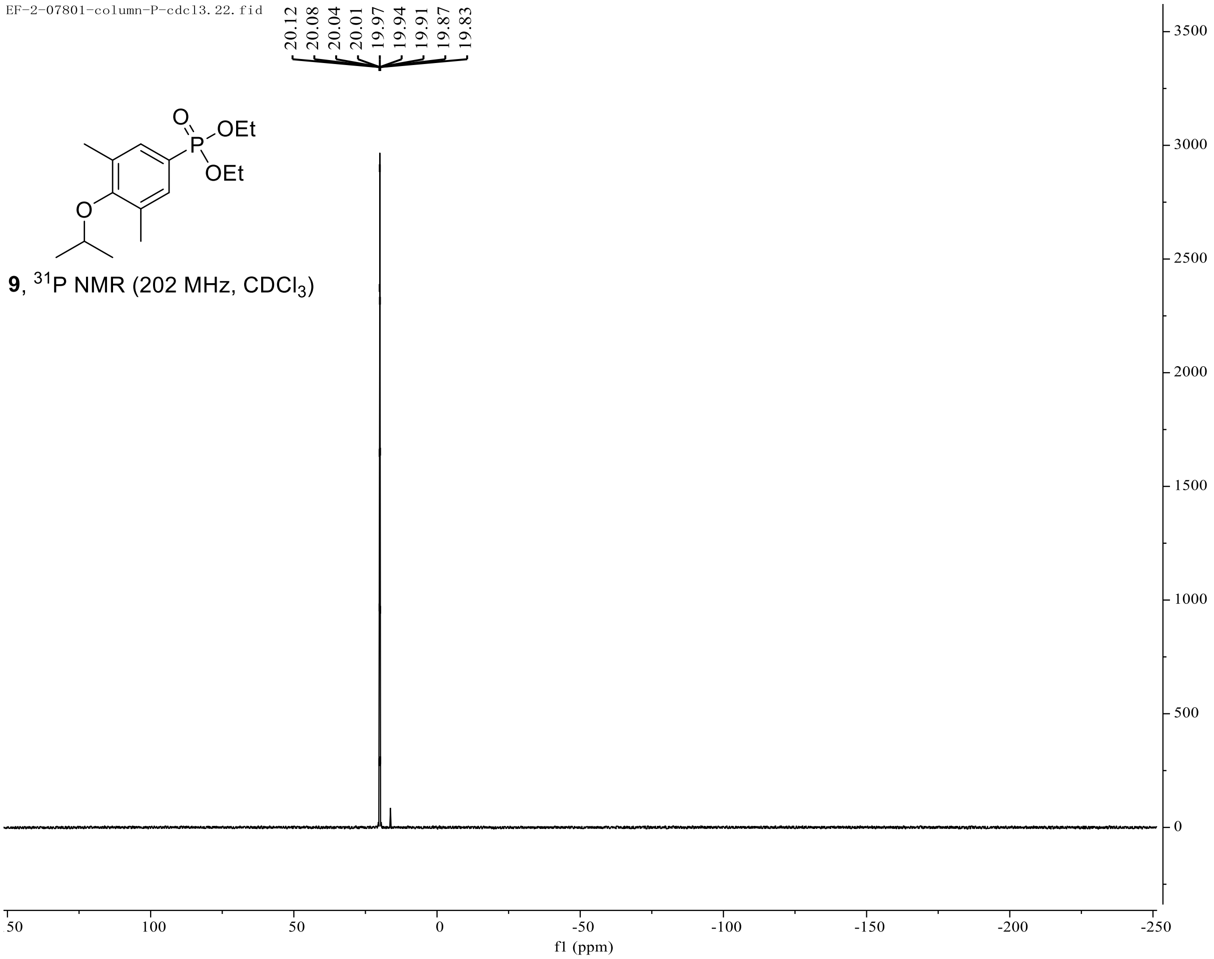

9, ^{31}P NMR (202 MHz, $CDCl_3$)

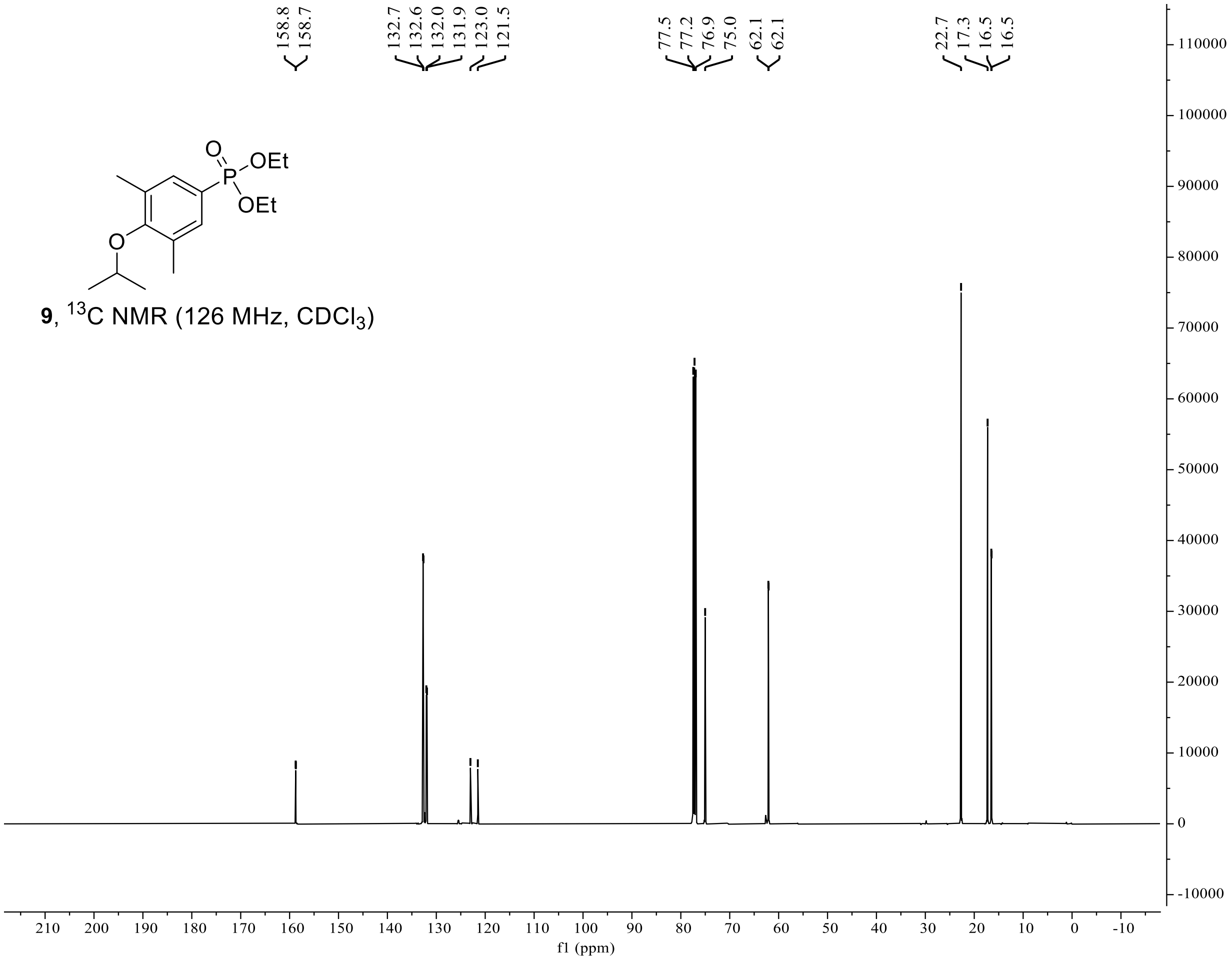

9, ^{13}C NMR (126 MHz, $CDCl_3$)

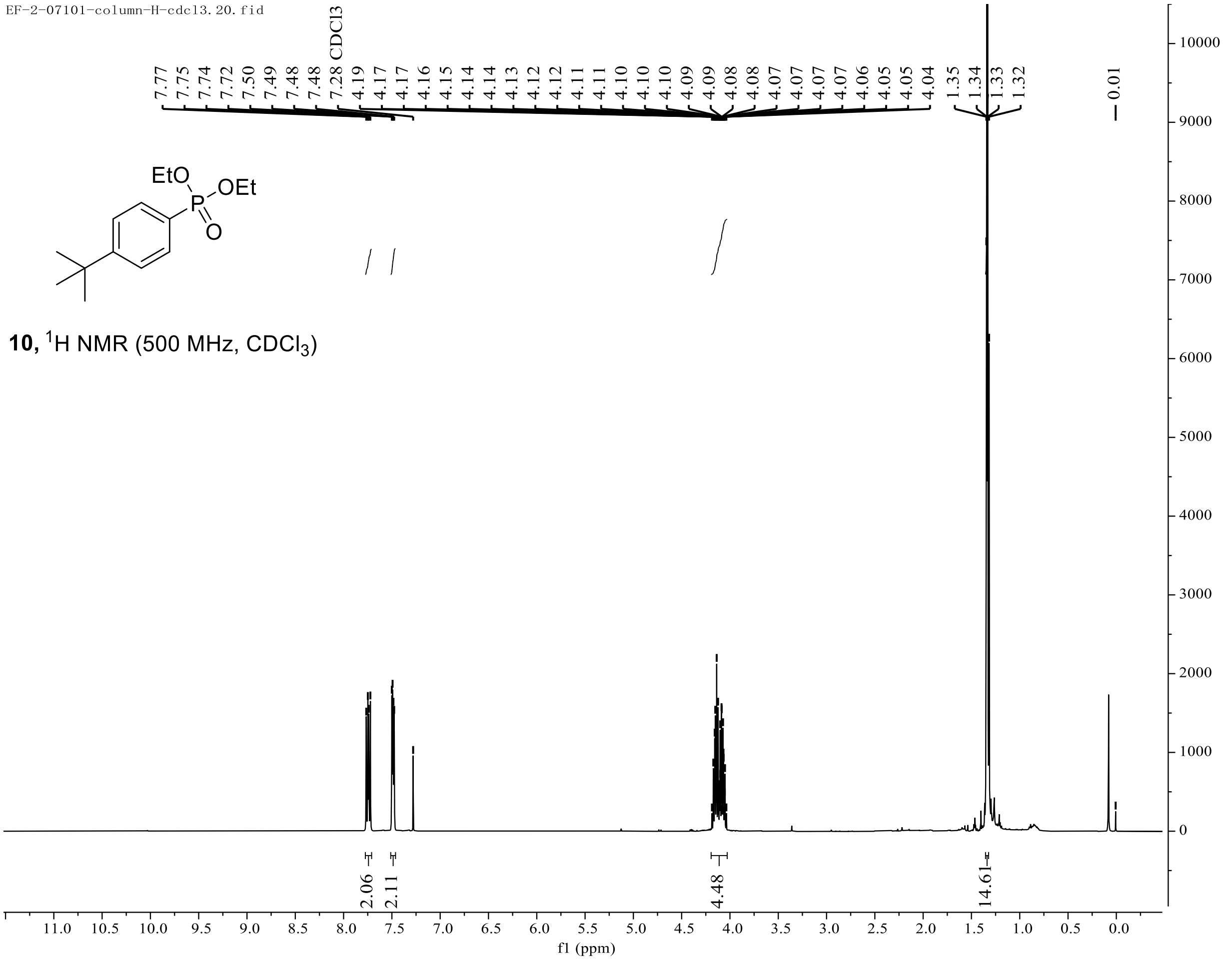
EF-2-07101-column-H-cdcl3.20.fid
7.77 7.75 7.74 7.72 7.50 7.49 7.48 7.48 7.28 CDCl3 4.19 4.17 4.17 4.16 4.15 4.14 4.14 4.13 4.12 4.12 4.11 4.11 4.10 4.10 4.10 4.09 4.09 4.08 4.08 4.07 4.07 4.07 4.07 4.06 4.05 4.05 4.04 1.35 1.34 1.33 1.32
0.01
EtO
OEt
P
O
10, ^{1}H NMR (500 MHz, $CDCl_3$)
2.06
2.11
4.48
14.61
f1 (ppm)

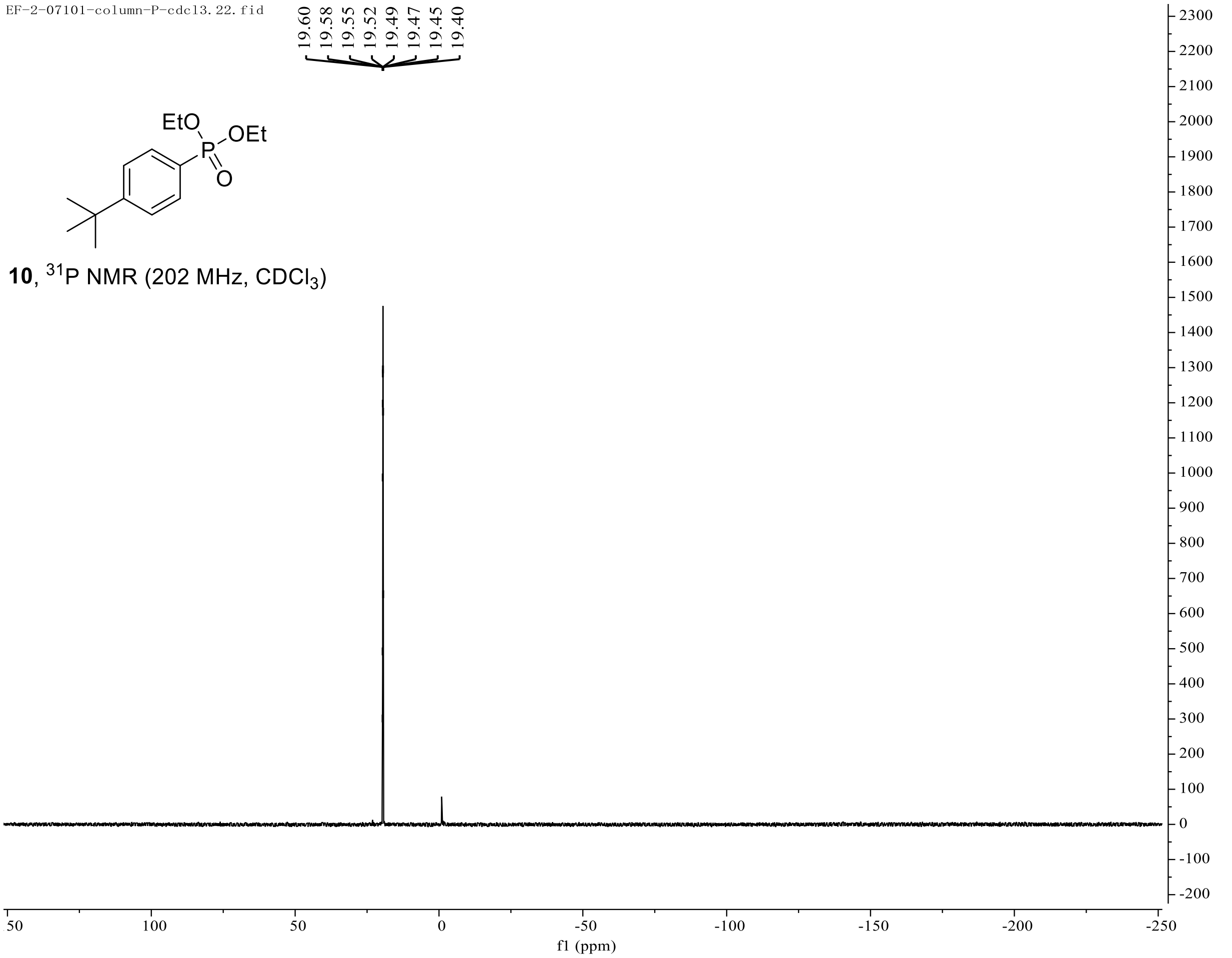
EF-2-07101-column-P-cdcl3.22.fid
19.60
19.58
19.55
19.52
19.49
19.47
19.45
19.40
EtO
OEt
P
O
10, ³¹P NMR (202 MHz, CDCl3)
f1 (ppm)

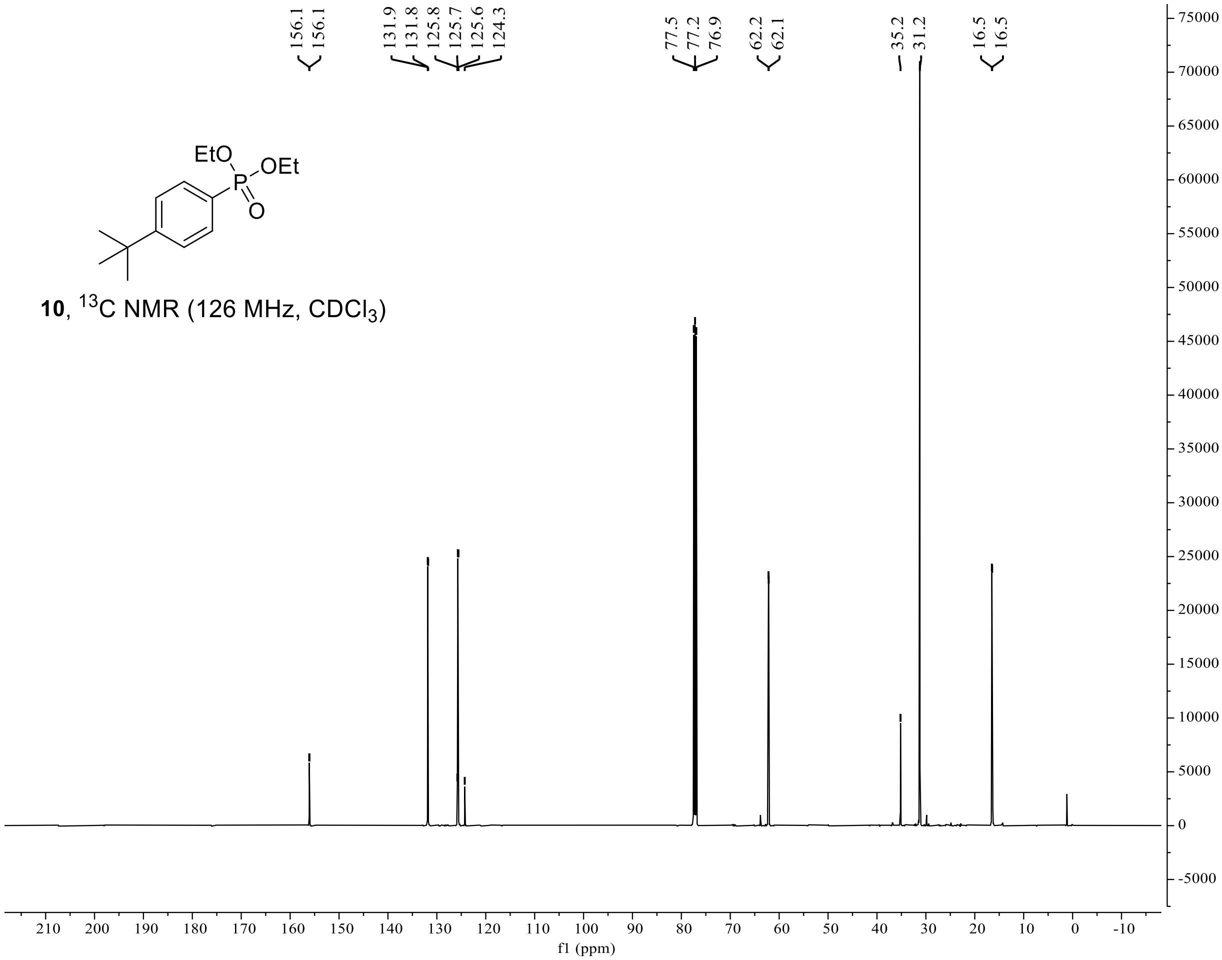

10, ^{13}C NMR (126 MHz, $CDCl_3$)

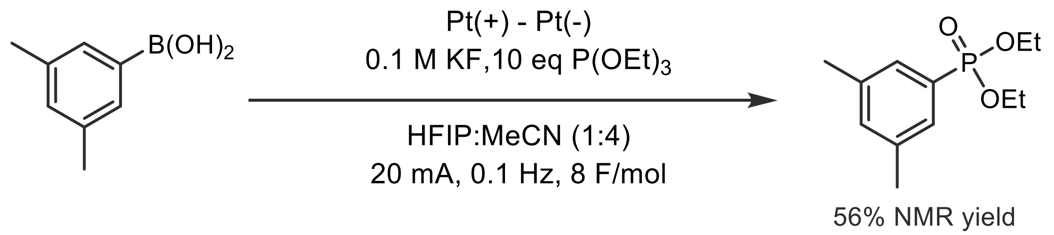
B(OH)2
Pt(+) - Pt(-)
0.1 M KF,10 eq P(OEt)3
HFIP:MeCN (1:4)
20 mA, 0.1 Hz, 8 F/mol
OEt
OEt
56% NMR yield

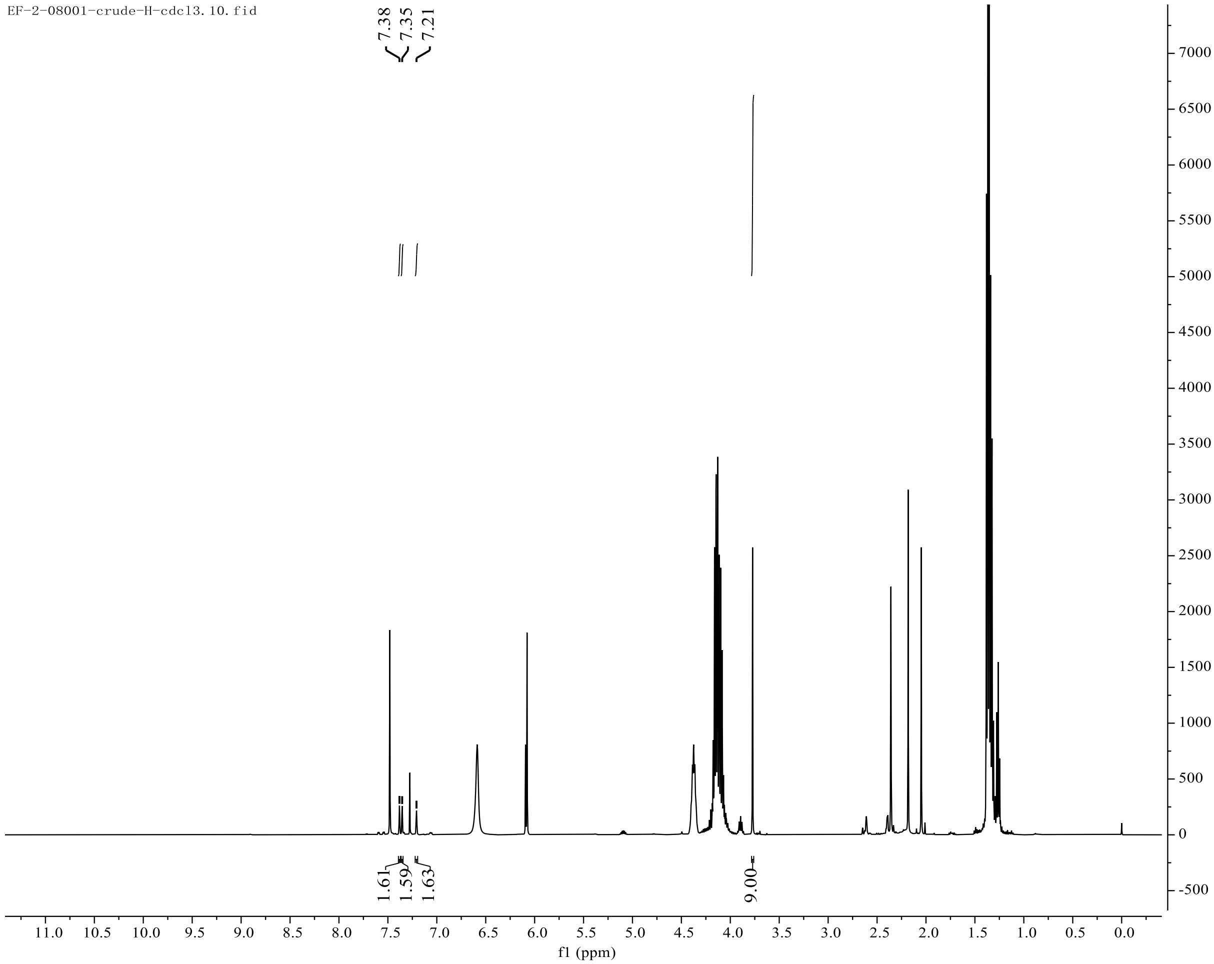
EF-2-08001-crude-H-cdcl3.10.fid
7.38
7.35
7.21
1.61
1.59
1.63
9.00
f1 (ppm)

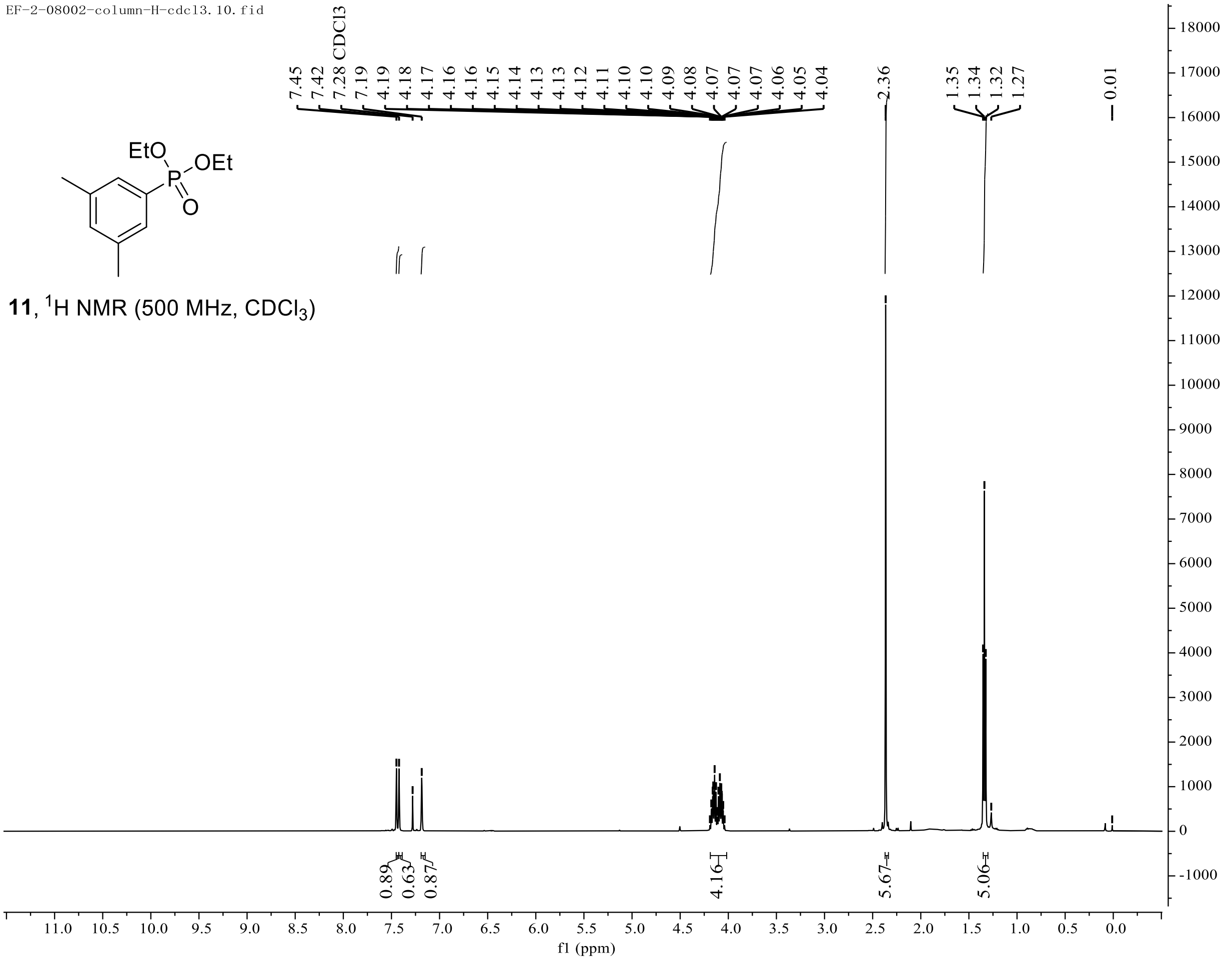
EF-2-08002-column-H-cdcl3.10.fid
EtO
OEt
P
O
11, 1H NMR (500 MHz, CDCl3)
7.45
7.42
7.28 CDCl3
7.19
4.19
4.18
4.17
4.16
4.16
4.15
4.14
4.13
4.13
4.12
4.11
4.10
4.10
4.09
4.08
4.07
4.07
4.07
4.06
4.05
4.04
2.36
1.35
1.34
1.32
1.27
0.01
0.89
0.63
0.87
4.16
5.67
5.06
f1 (ppm)

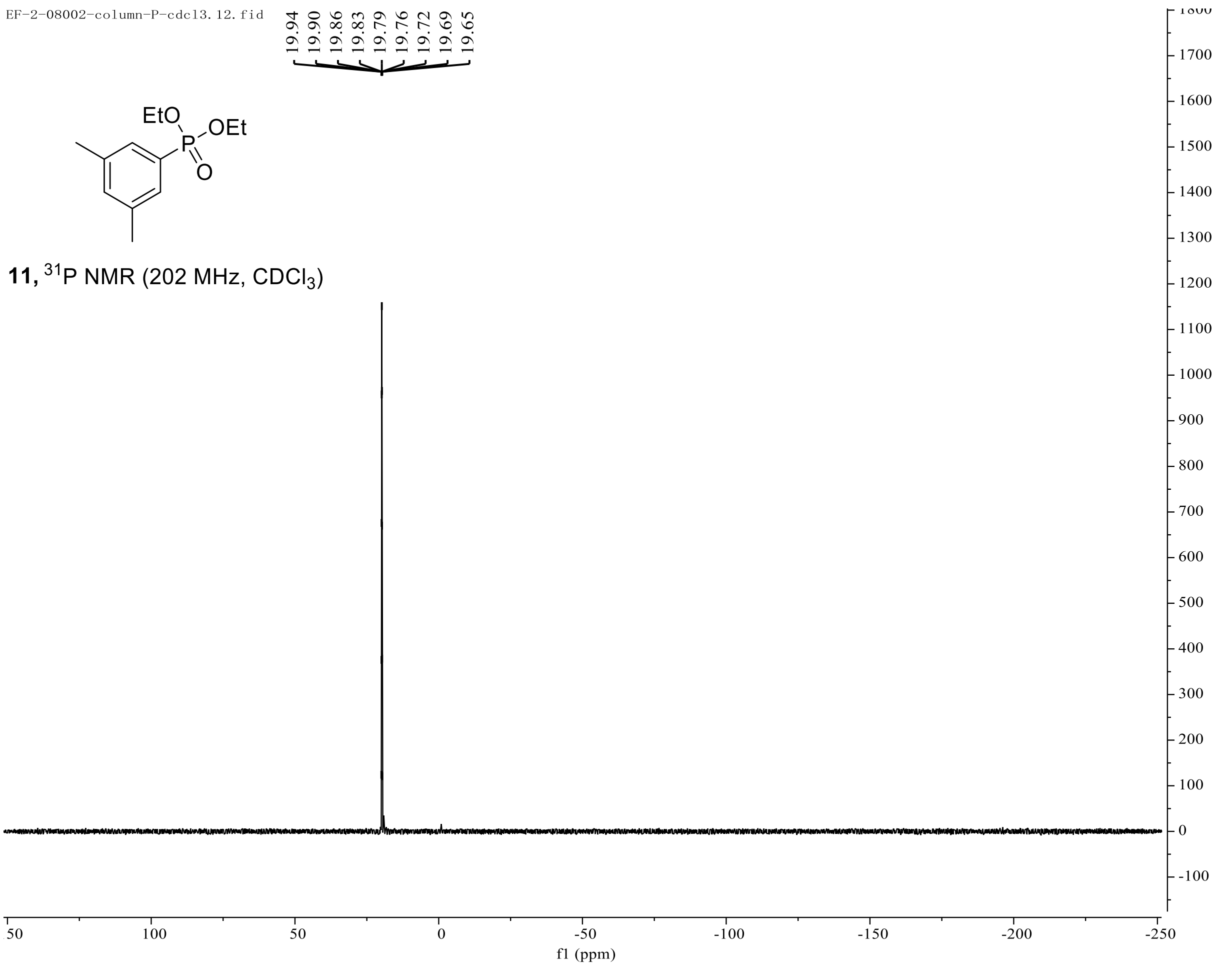

11, ^{31}P NMR (202 MHz, $CDCl_3$)

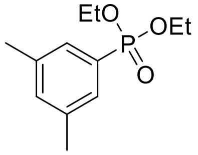

11, ^{13}C NMR (126 MHz, $CDCl_3$)

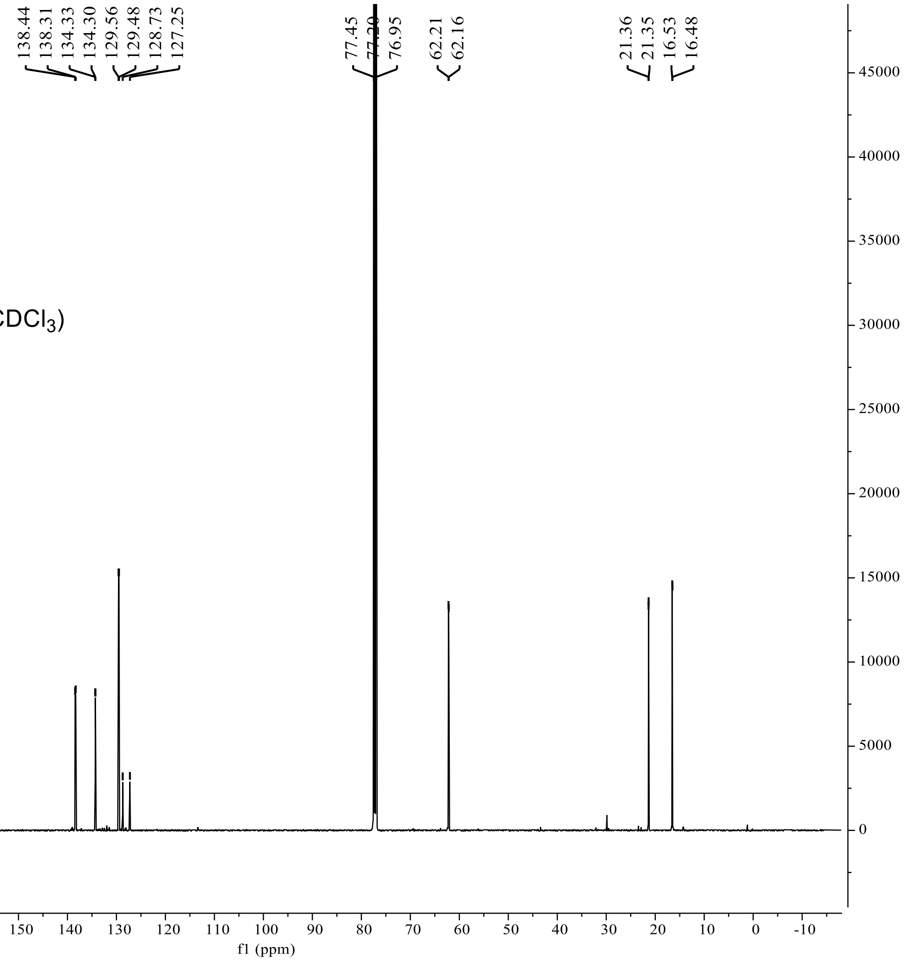

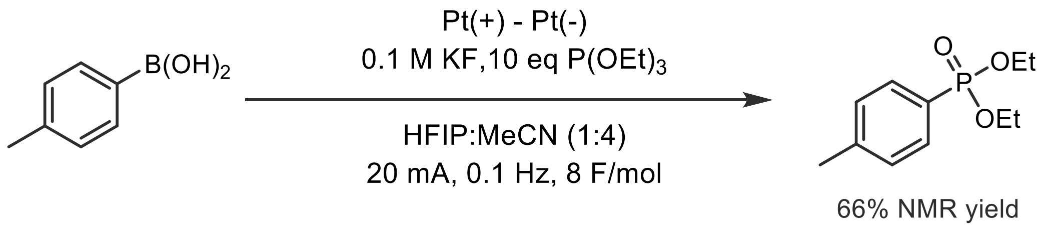
B(OH)$_2$
Pt(+) - Pt(-)
0.1 M KF,10 eq P(OEt)$_3$
HFIP:MeCN (1:4)
20 mA, 0.1 Hz, 8 F/mol
OEt
OEt
66% NMR yield

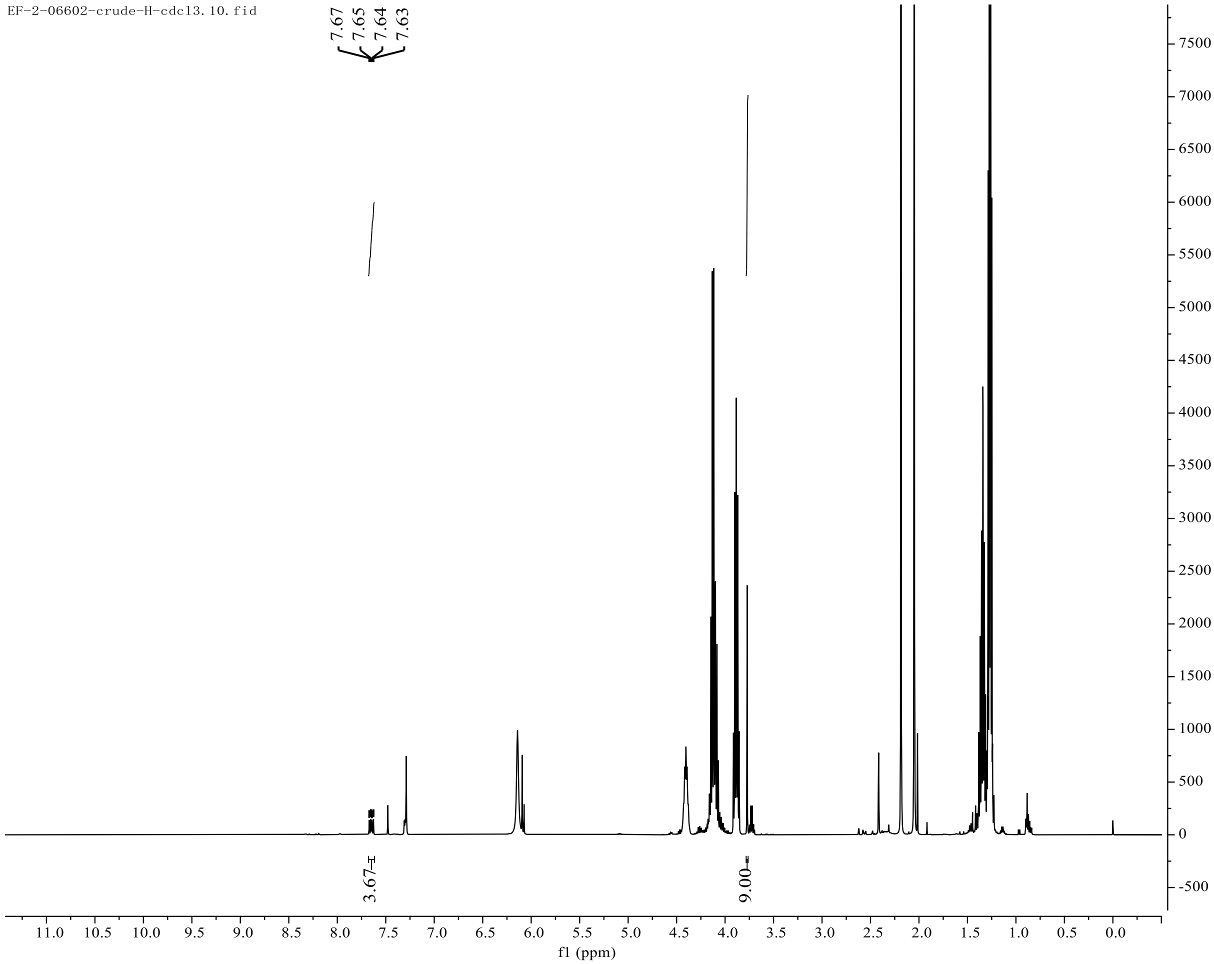
EF-2-06602-crude-H-cdcl3.10.fid
7.67
7.65
7.64
7.63
3.67
9.00
f1 (ppm)

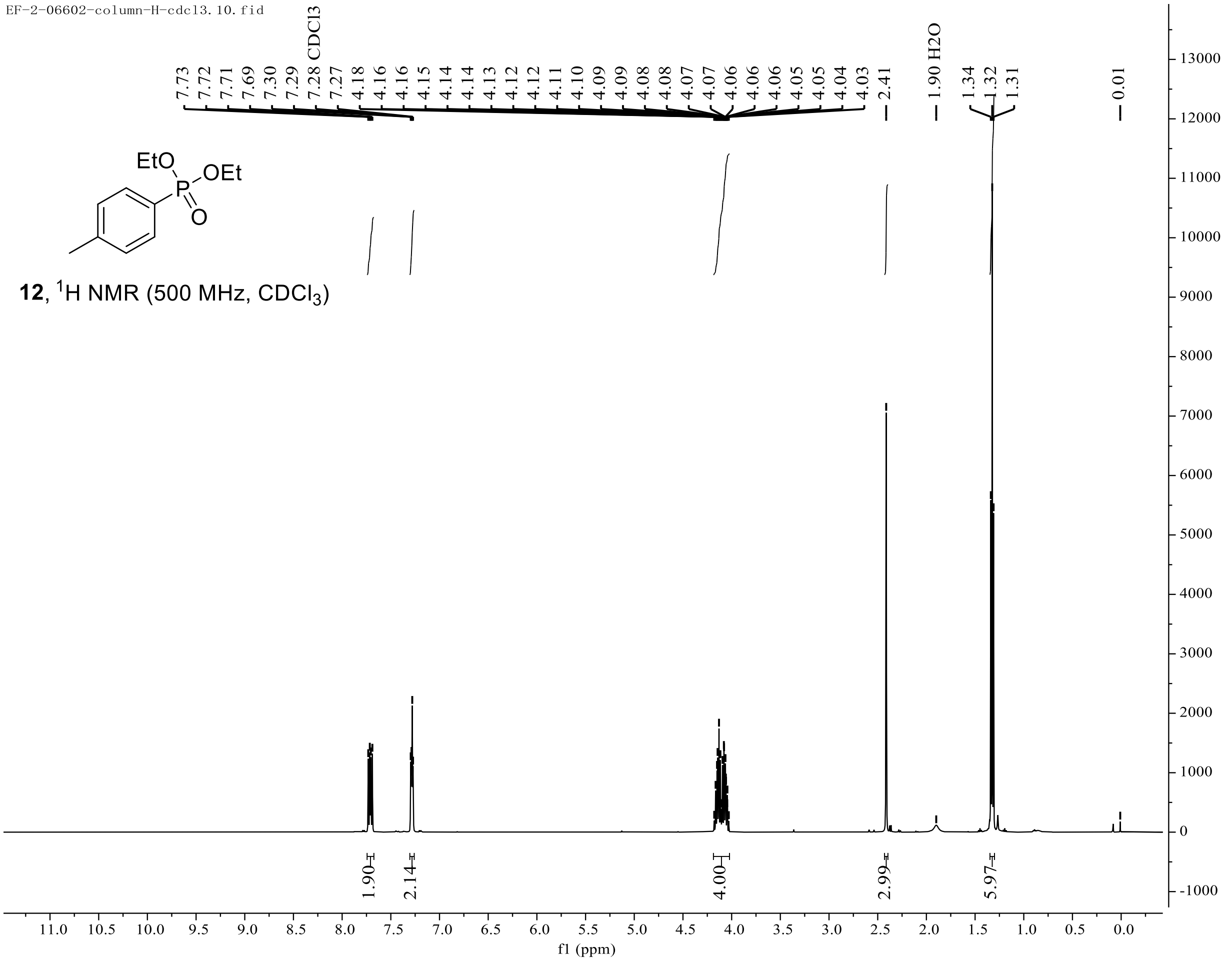
EF-2-06602-column-H-cdcl3.10.fid
7.73
7.72
7.71
7.69
7.30
7.29
7.28 CDCl3
7.27
4.18
4.16
4.16
4.15
4.14
4.14
4.13
4.12
4.12
4.11
4.10
4.09
4.09
4.08
4.08
4.07
4.07
4.06
4.06
4.06
4.05
4.05
4.04
4.03
2.41
1.90 H2O
1.34
1.32
1.31
0.01
EtO
OEt
P
O
12, 1H NMR (500 MHz, CDCl3)
1.90
2.14
4.00
2.99
5.97
f1 (ppm)

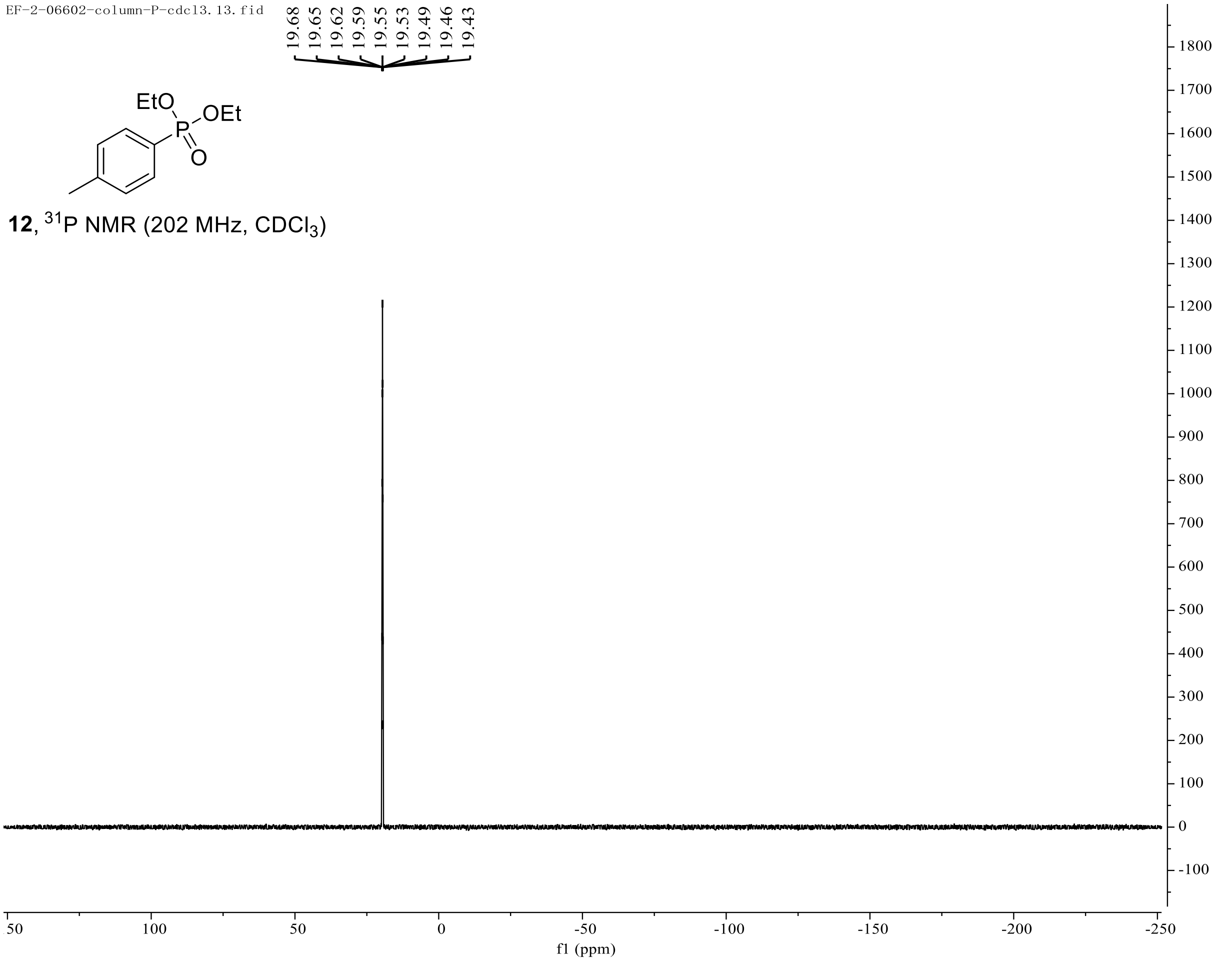

12, ^{31}P NMR (202 MHz, $CDCl_3$)

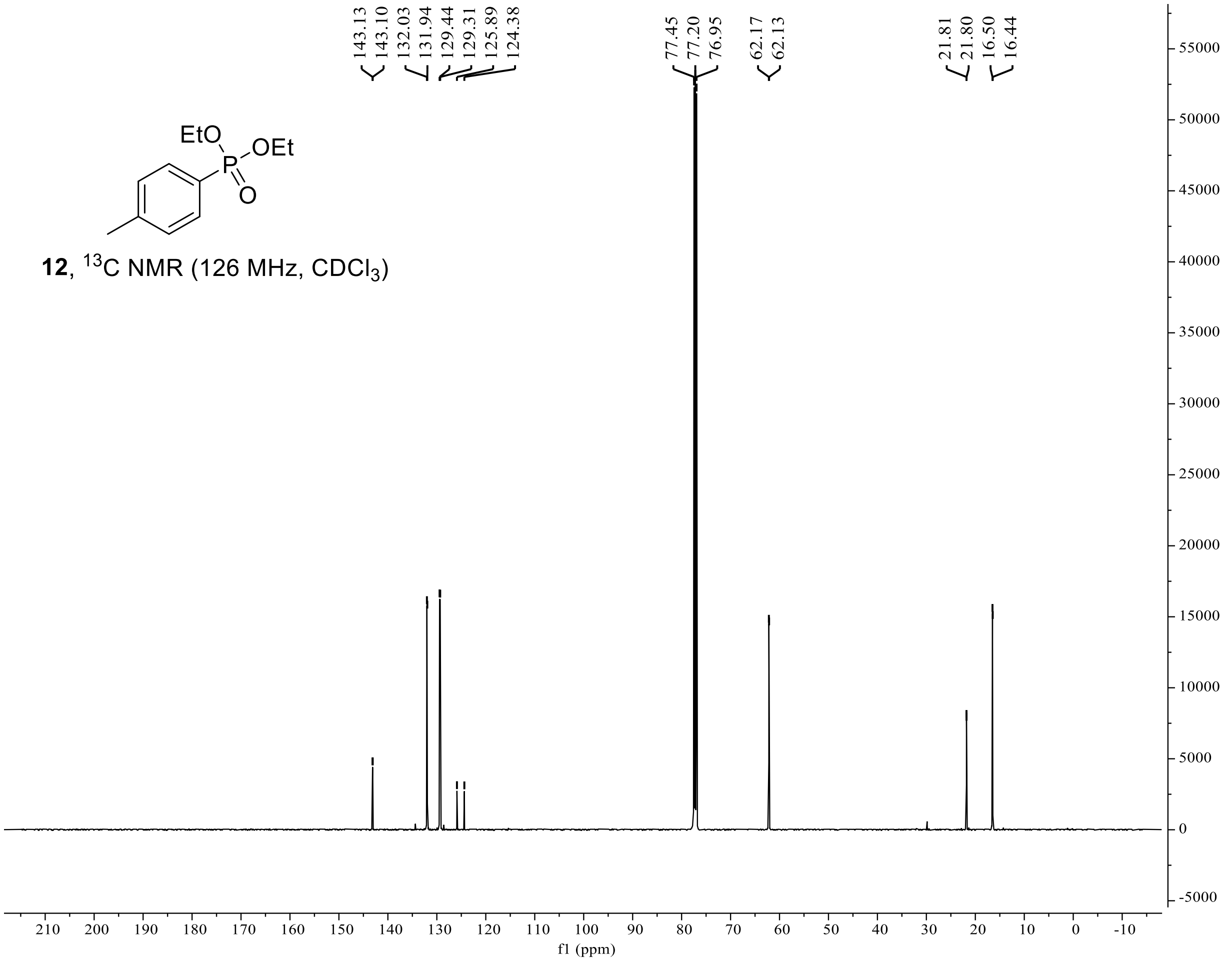

143.13
143.10
132.03
131.94
129.44
129.31
125.89
124.38
77.45
77.20
76.95
62.17
62.13
21.81
21.80
16.50
16.44
EtO
OEt
P
O
12, ^{13}C NMR (126 MHz, CDCl$_3$)
f1 (ppm)

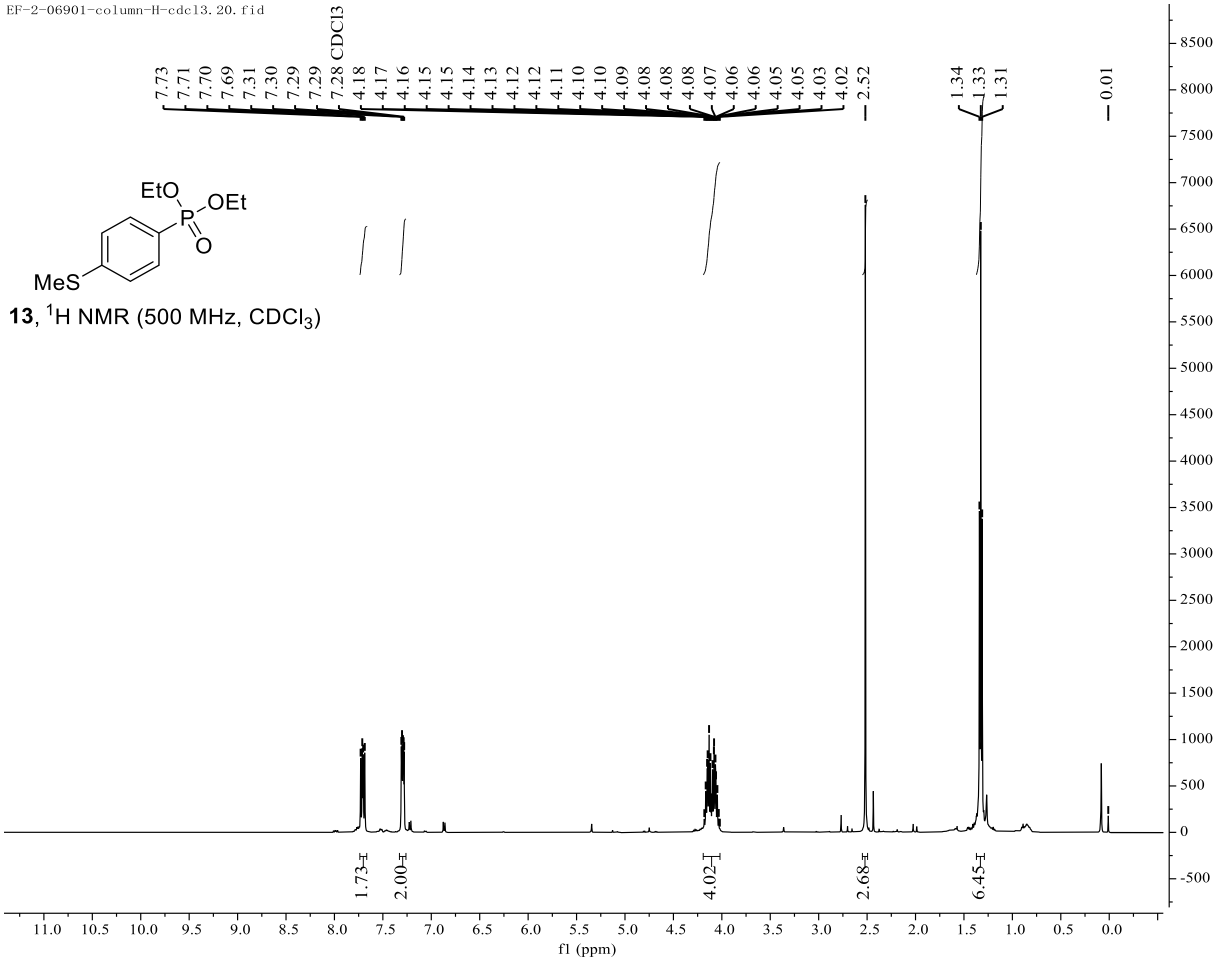

13, ^{1}H NMR (500 MHz, $CDCl_3$)

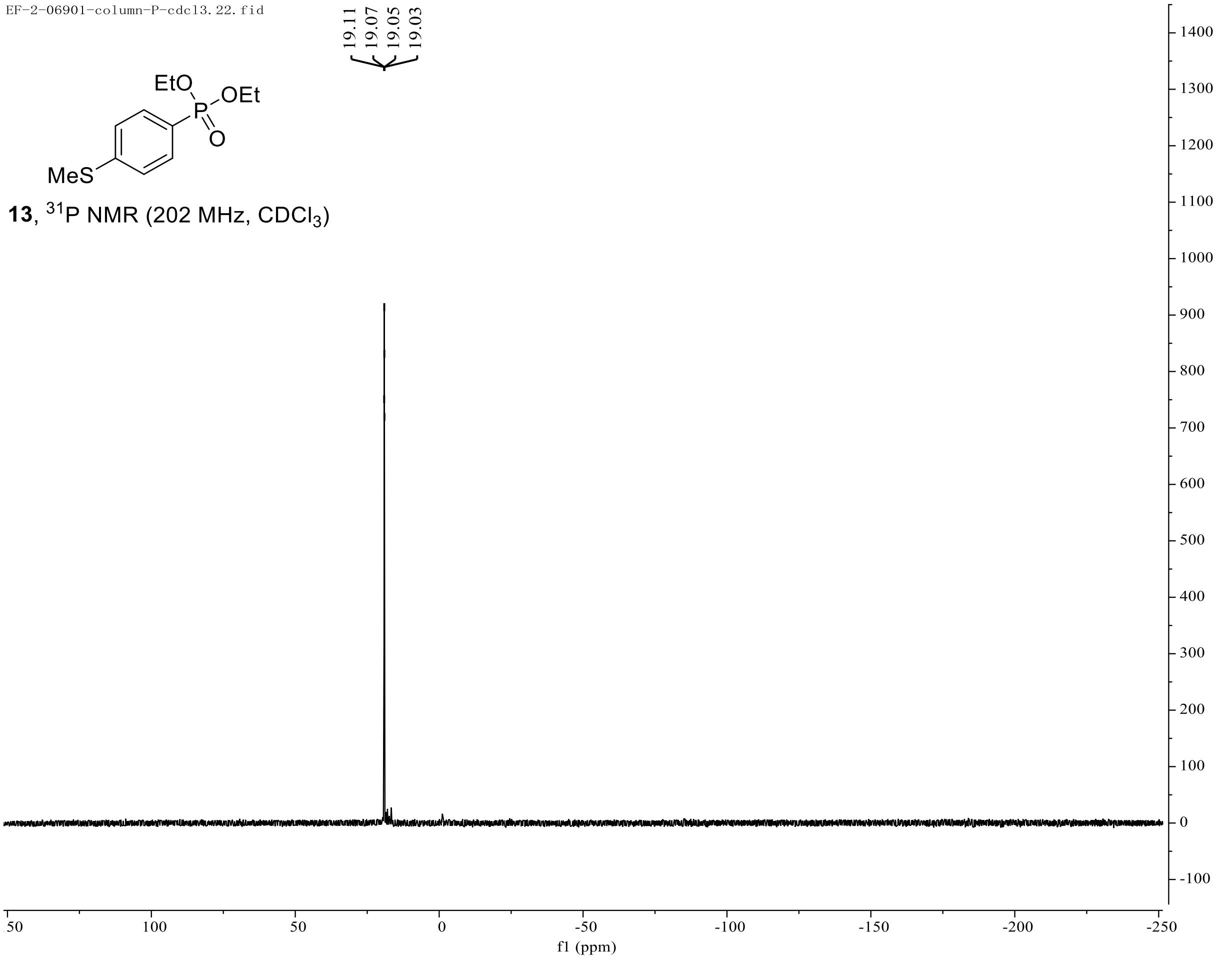
EF-2-06901-column-P-cdcl3.22.fid
19.11
19.07
19.05
19.03
EtO
OEt
P
O
MeS
13, ³¹P NMR (202 MHz, CDCl₃)
f1 (ppm)

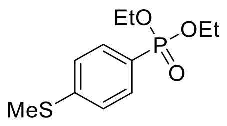

13, ^{13}C NMR (126 MHz, $CDCl_3$)

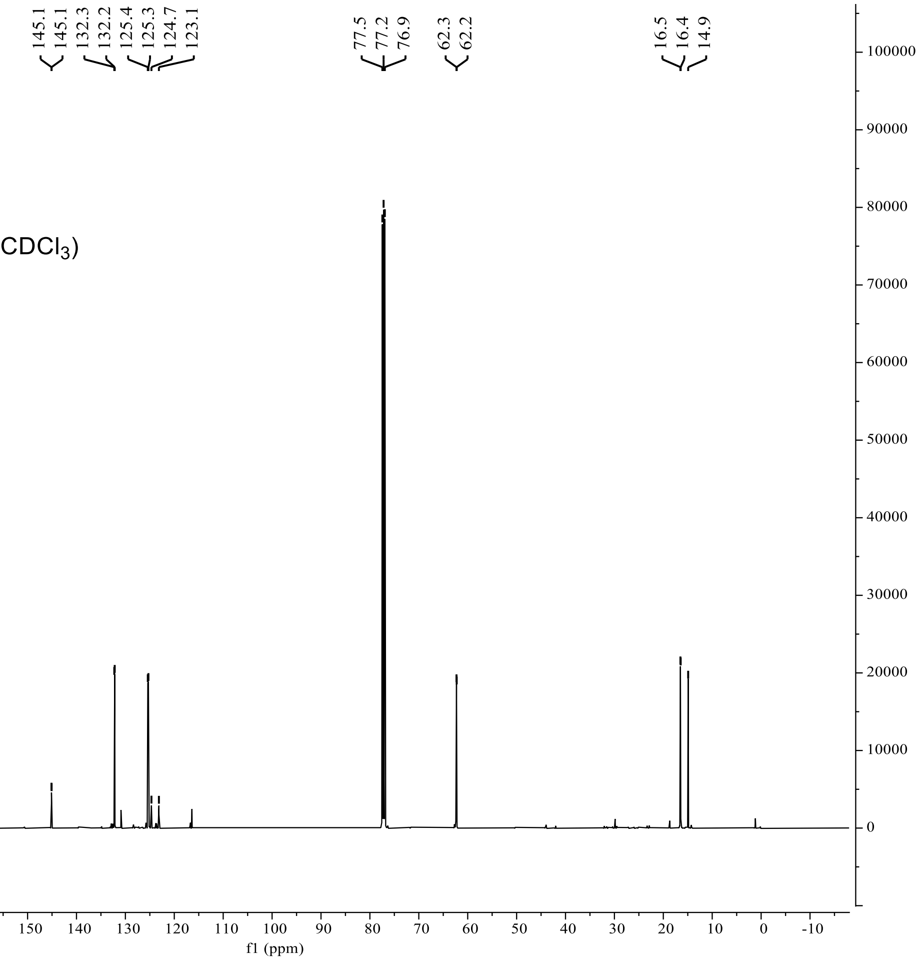

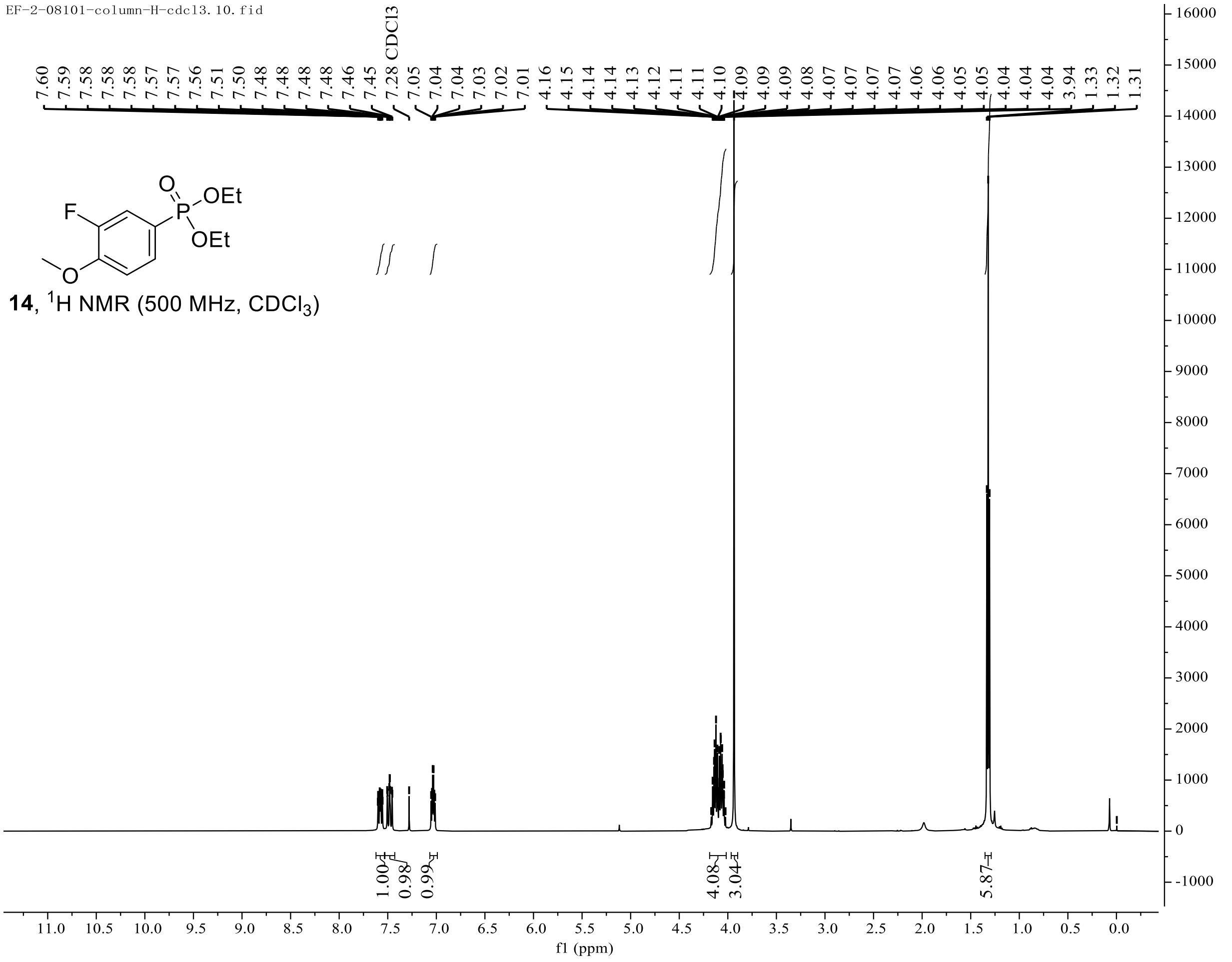

14, ^{1}H NMR (500 MHz, $CDCl_3$)

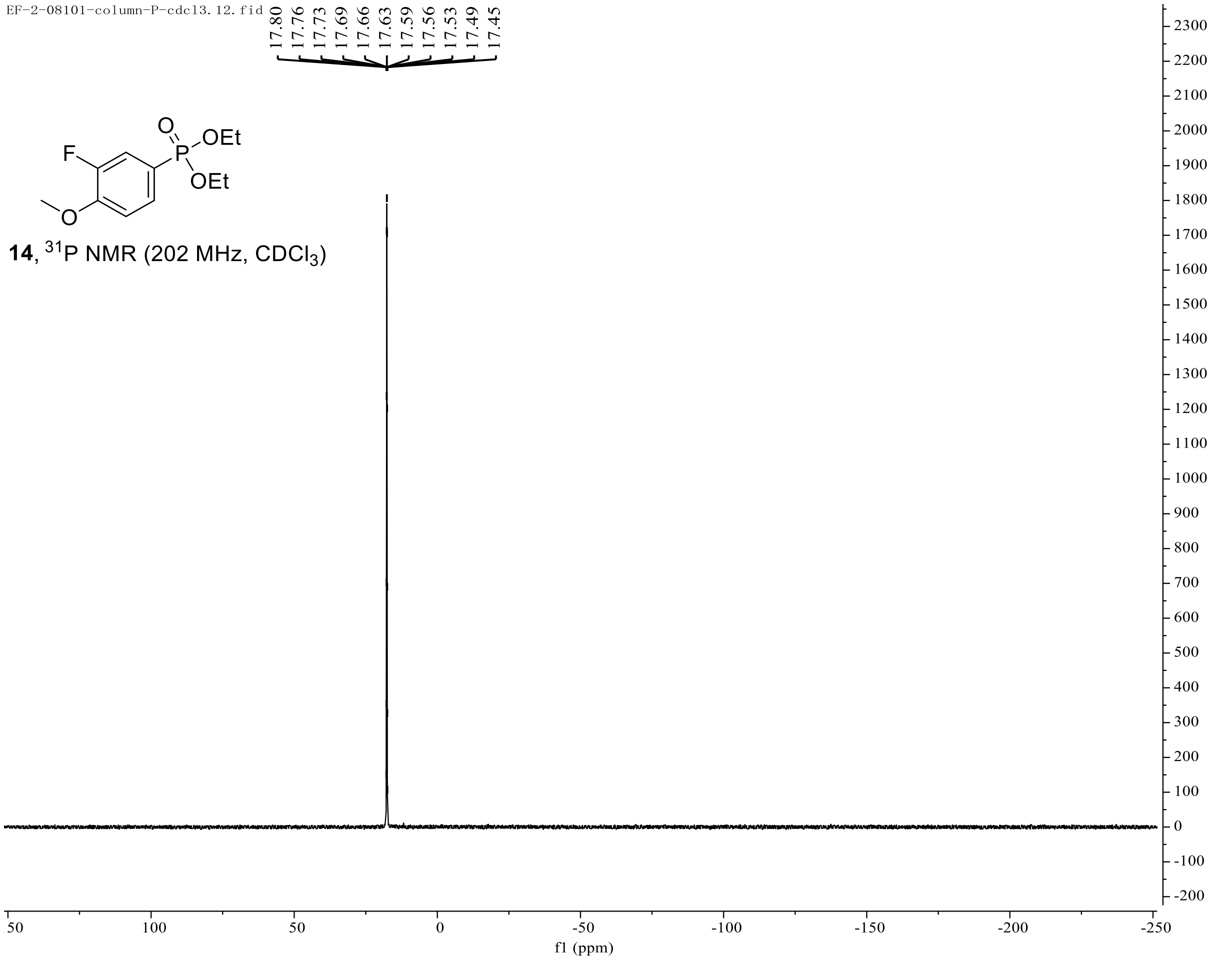

EF-2-08101-column-P-cdcl3.12.fid
17.80
17.76
17.73
17.69
17.66
17.63
17.59
17.56
17.53
17.49
17.45
F
O
P
OEt
OEt
O
14, ^{31}P NMR (202 MHz, $CDCl_3$)
f1 (ppm)

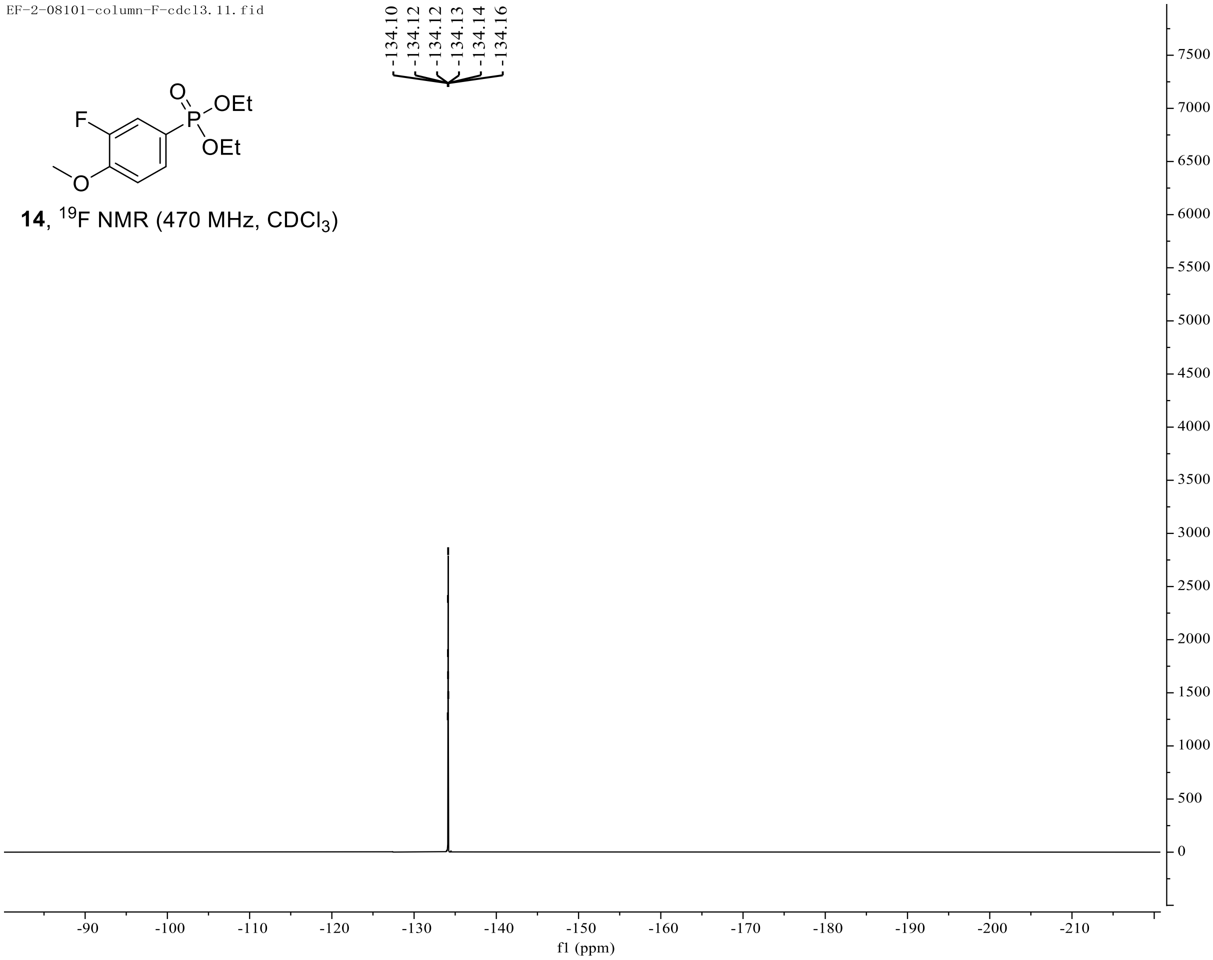

14, ^{19}F NMR (470 MHz, $CDCl_3$)

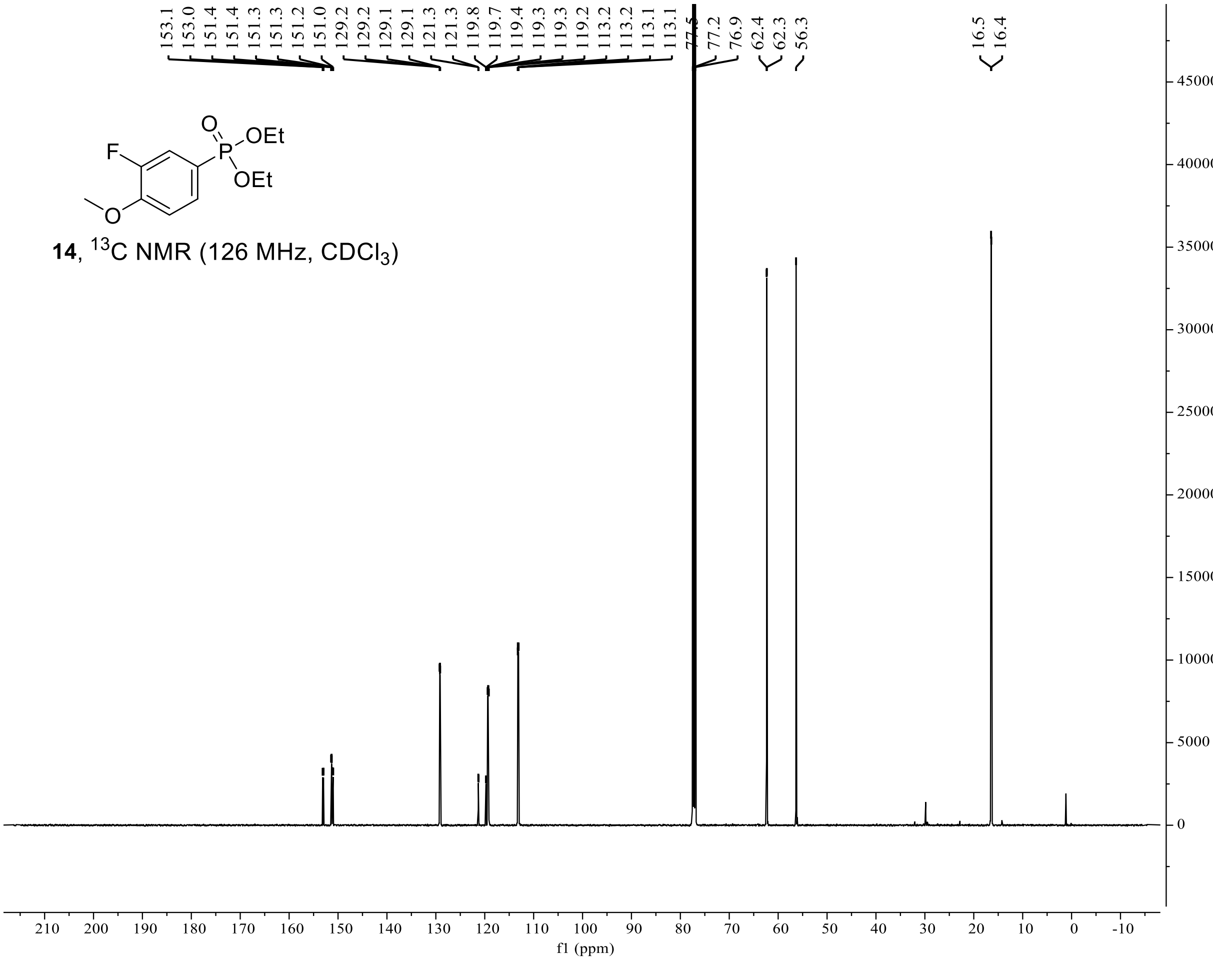

153.1
153.0
151.4
151.4
151.3
151.3
151.2
151.0
129.2
129.2
129.1
129.1
121.3
121.3
119.8
119.7
119.4
119.3
119.3
119.2
113.2
113.2
113.1
113.1
77.5
77.2
76.9
62.4
62.3
56.3
16.5
16.4
F
O
OEt
OEt
P
O
14, ^{13}C NMR (126 MHz, $CDCl_3$)
45000
40000
35000
30000
25000
20000
15000
10000
5000
0
210 200 190 180 170 160 150 140 130 120 110 100 90 80 70 60 50 40 30 20 10 0 -10
f1 (ppm)

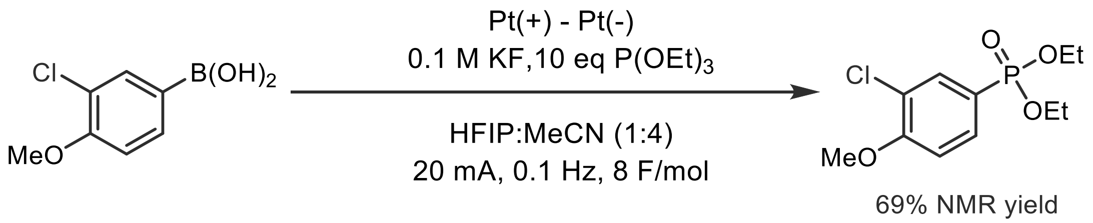
Pt(+) - Pt(-)
0.1 M KF,10 eq P(OEt)$_3$
HFIP:MeCN (1:4)
20 mA, 0.1 Hz, 8 F/mol
69% NMR yield

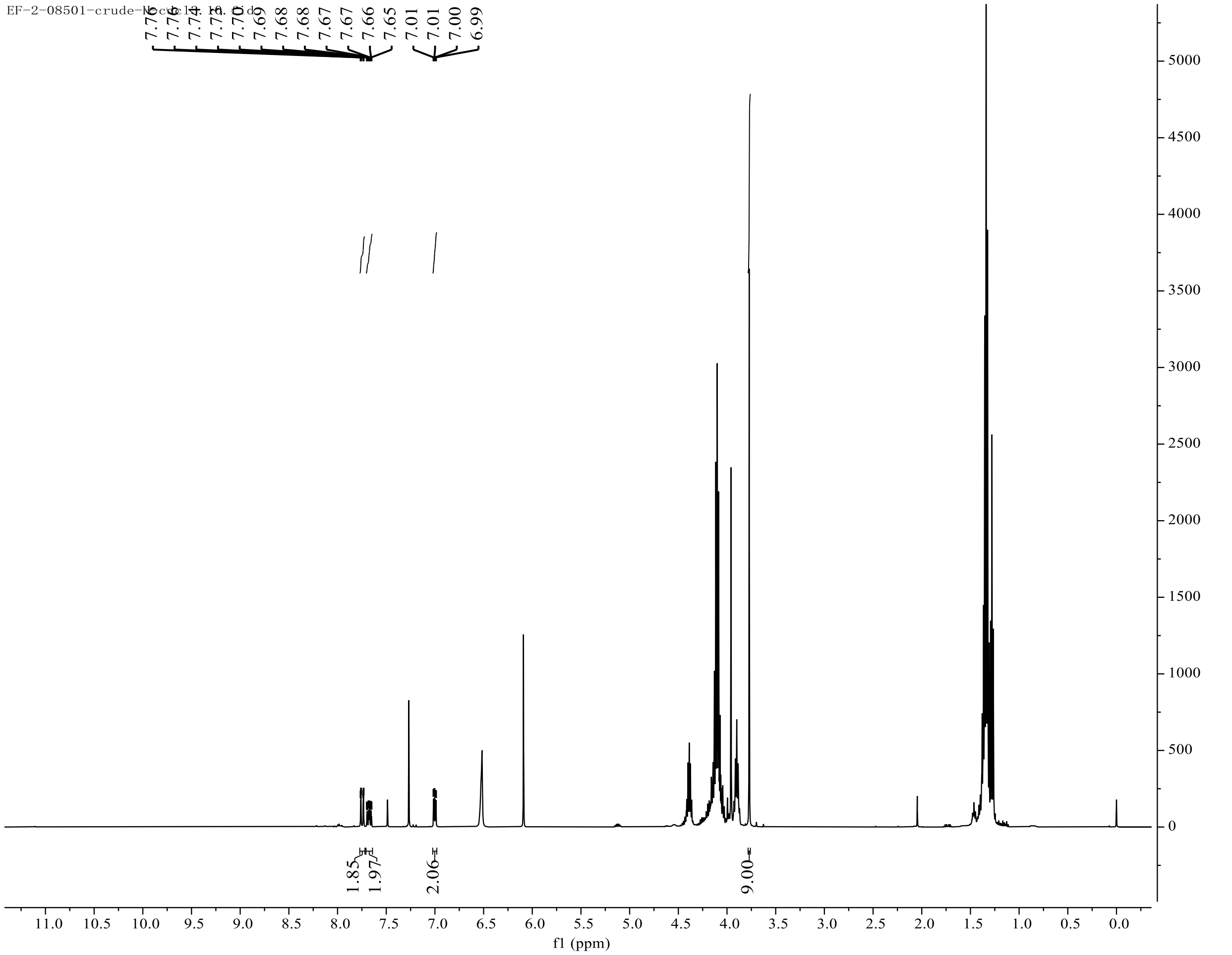
EF-2-08501-crude
f1 (ppm)

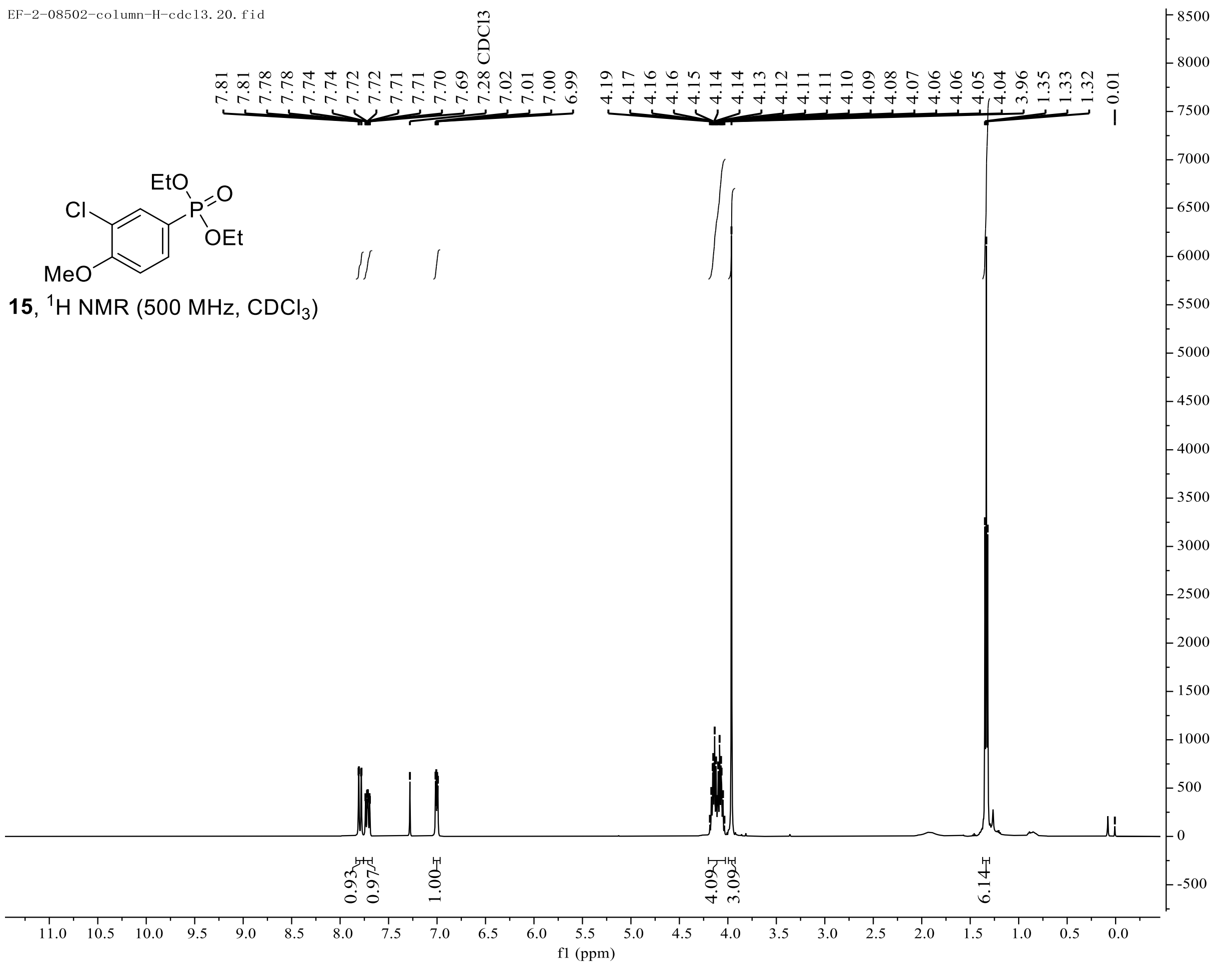

EF-2-08502-column-H-cdcl3.20.fid
EtO
O
P
Cl
OEt
MeO
15, ^{1}H NMR (500 MHz, $CDCl_3$)
7.81 7.81 7.78 7.78 7.74 7.74 7.72 7.72 7.71 7.71 7.70 7.69 7.28 CDCl3 7.02 7.01 7.00 6.99
4.19 4.17 4.16 4.15 4.14 4.14 4.13 4.12 4.11 4.11 4.10 4.09 4.08 4.07 4.06 4.06 4.05 4.04 3.96 1.35 1.33 1.32
0.01
0.93 0.97 1.00 4.09 3.09 6.14
f1 (ppm)

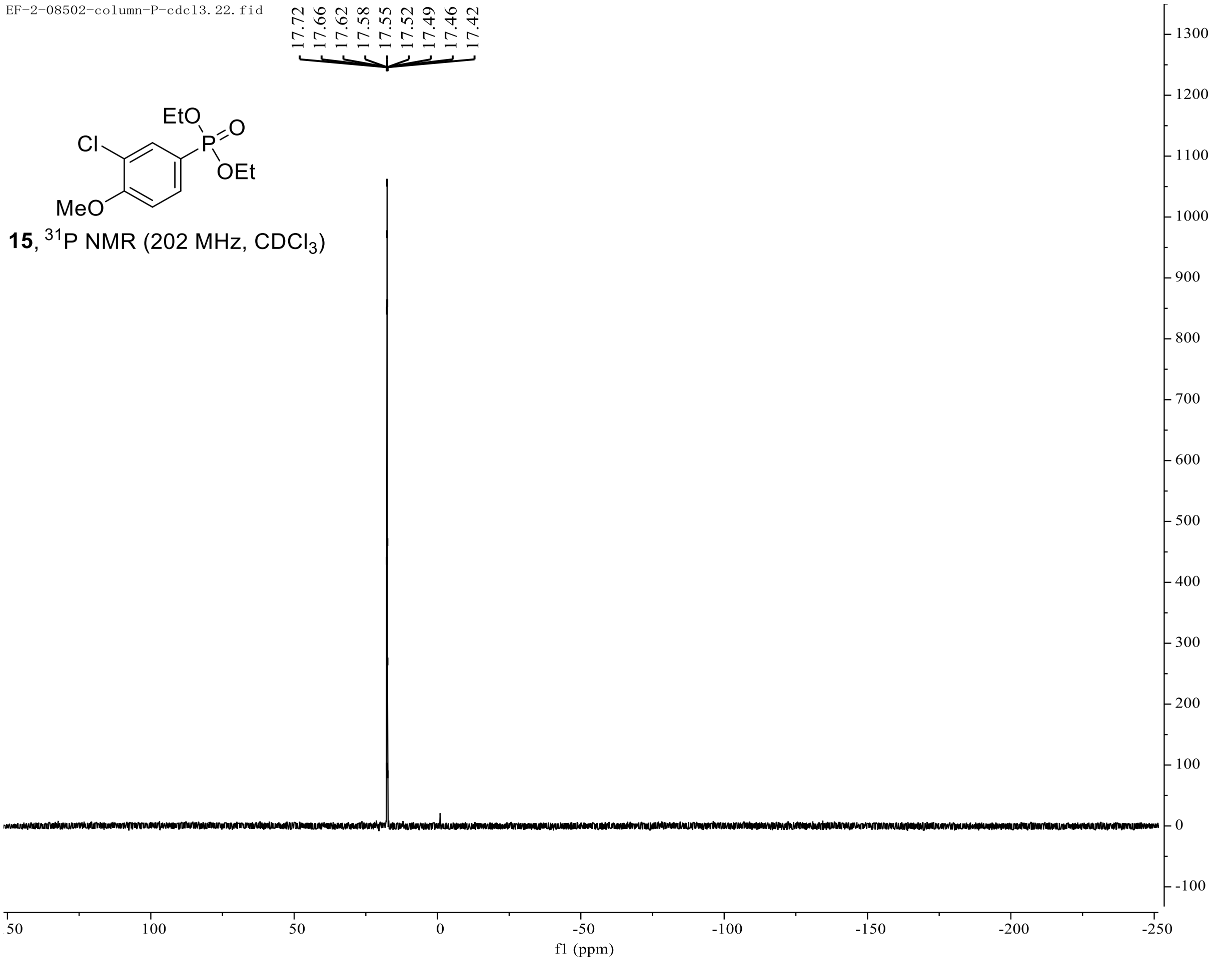

EF-2-08502-column-P-cdcl3.22.fid
17.72
17.66
17.62
17.58
17.55
17.52
17.49
17.46
17.42
EtO
P
O
Cl
OEt
MeO
15, 31P NMR (202 MHz, CDCl3)
1300
1200
1100
1000
900
800
700
600
500
400
300
200
100
0
-100
50
100
50
0
-50
-100
-150
-200
-250
f1 (ppm)

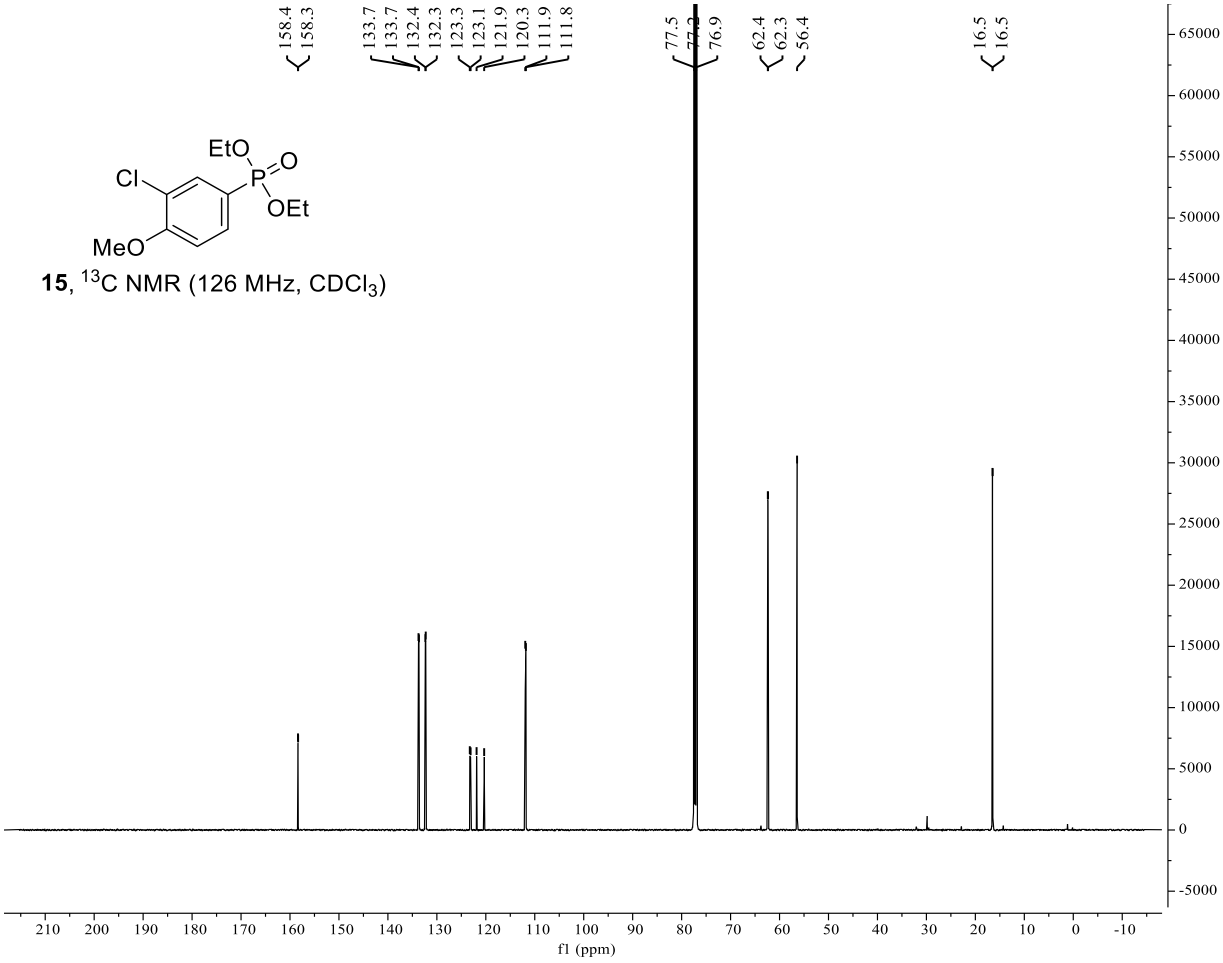

15, ^{13}C NMR (126 MHz, $CDCl_3$)

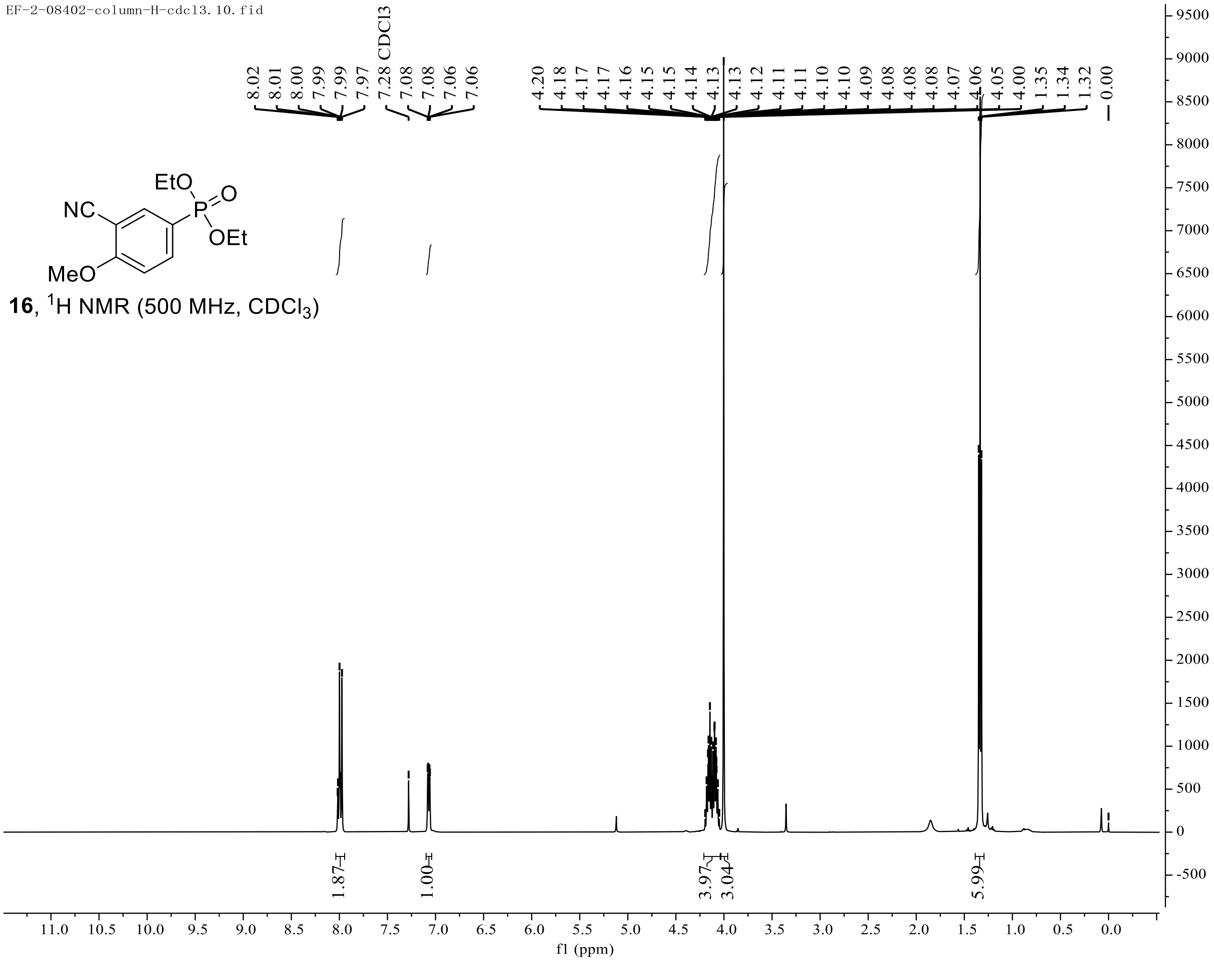

16, ^{1}H NMR (500 MHz, $CDCl_3$)

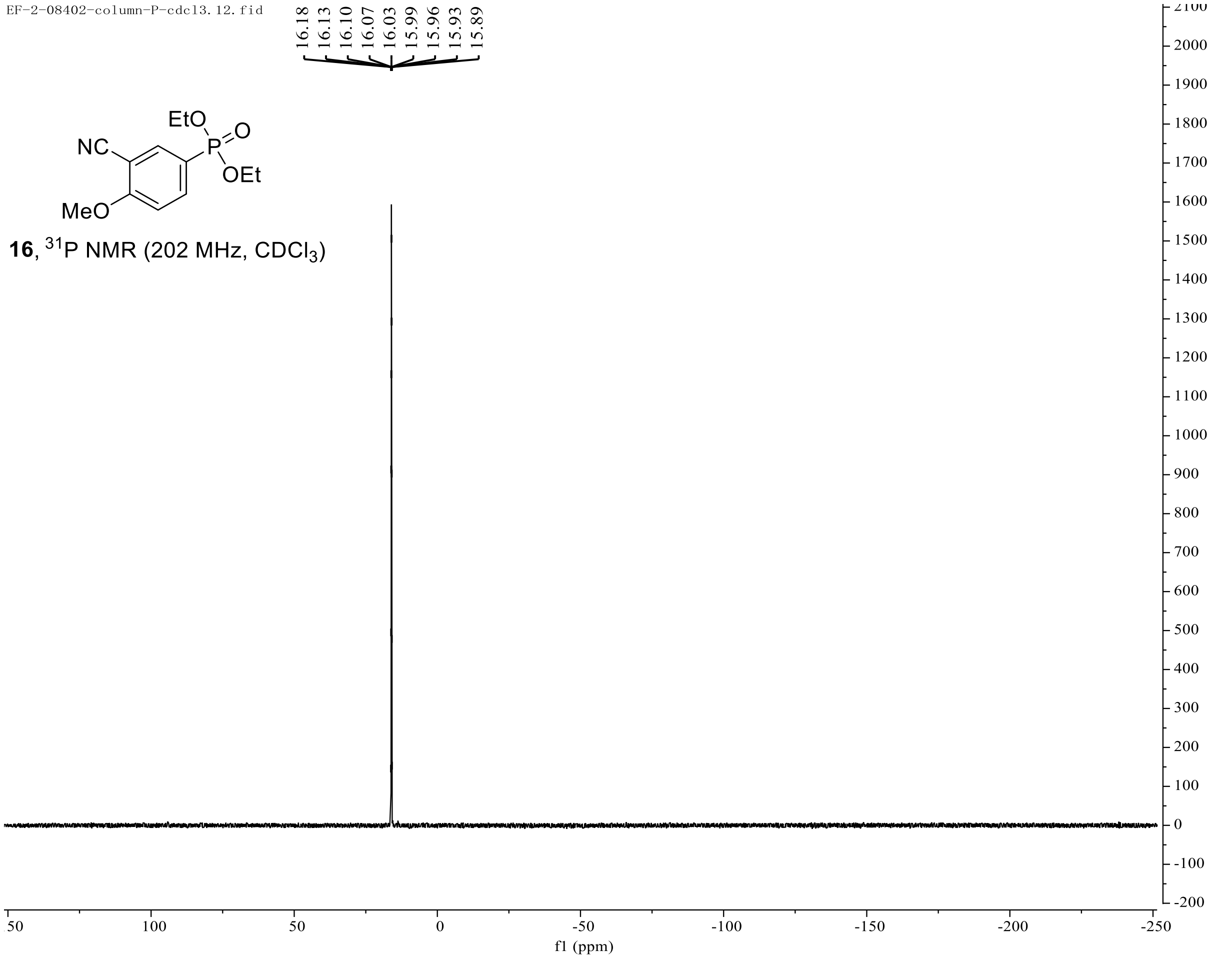

EF-2-08402-column-P-cdcl3.12.fid
16.18
16.13
16.10
16.07
16.03
15.99
15.96
15.93
15.89
EtO
P
O
OEt
NC
MeO
16, ^{31}P NMR (202 MHz, $CDCl_3$)
f1 (ppm)

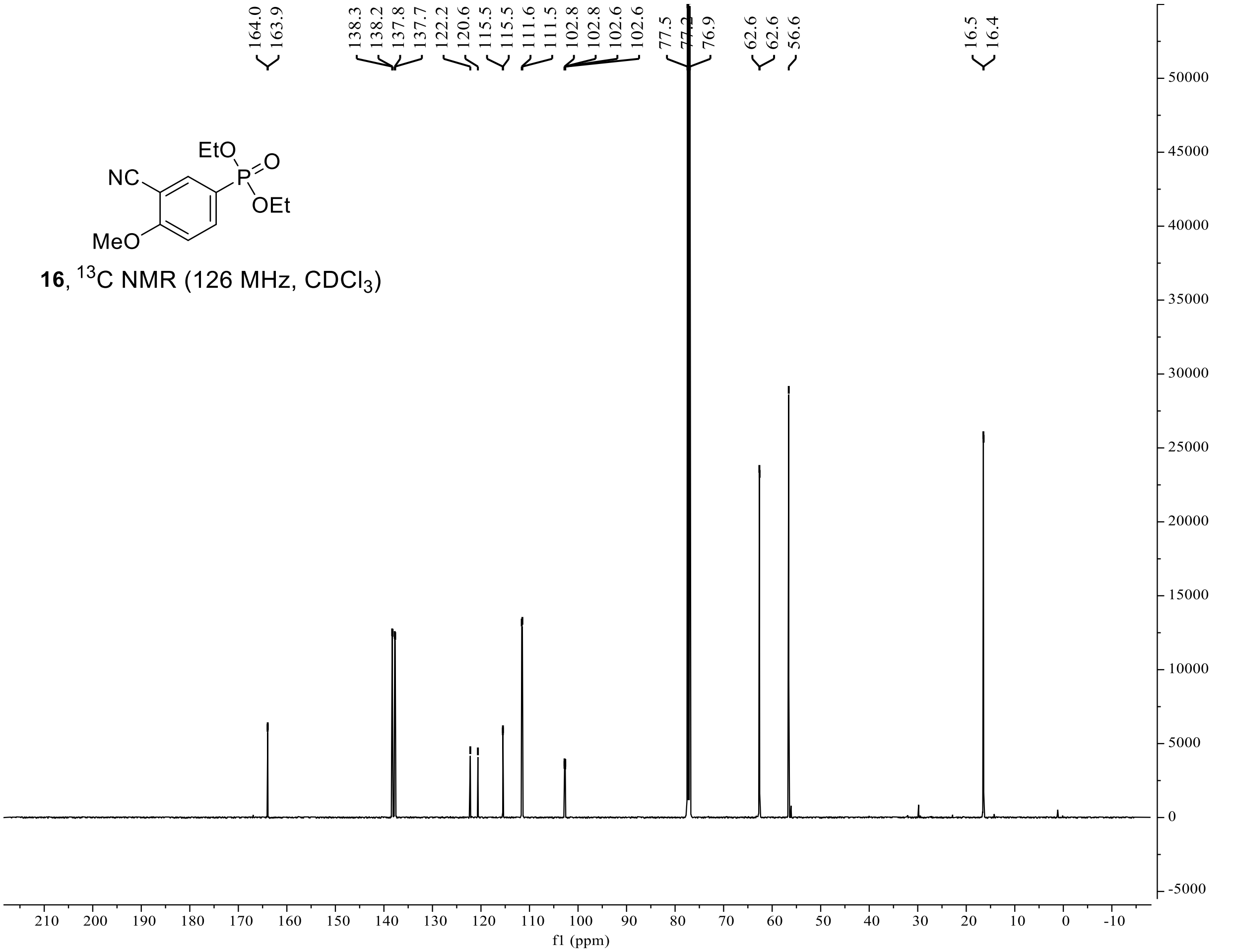

16, ^{13}C NMR (126 MHz, $CDCl_3$)

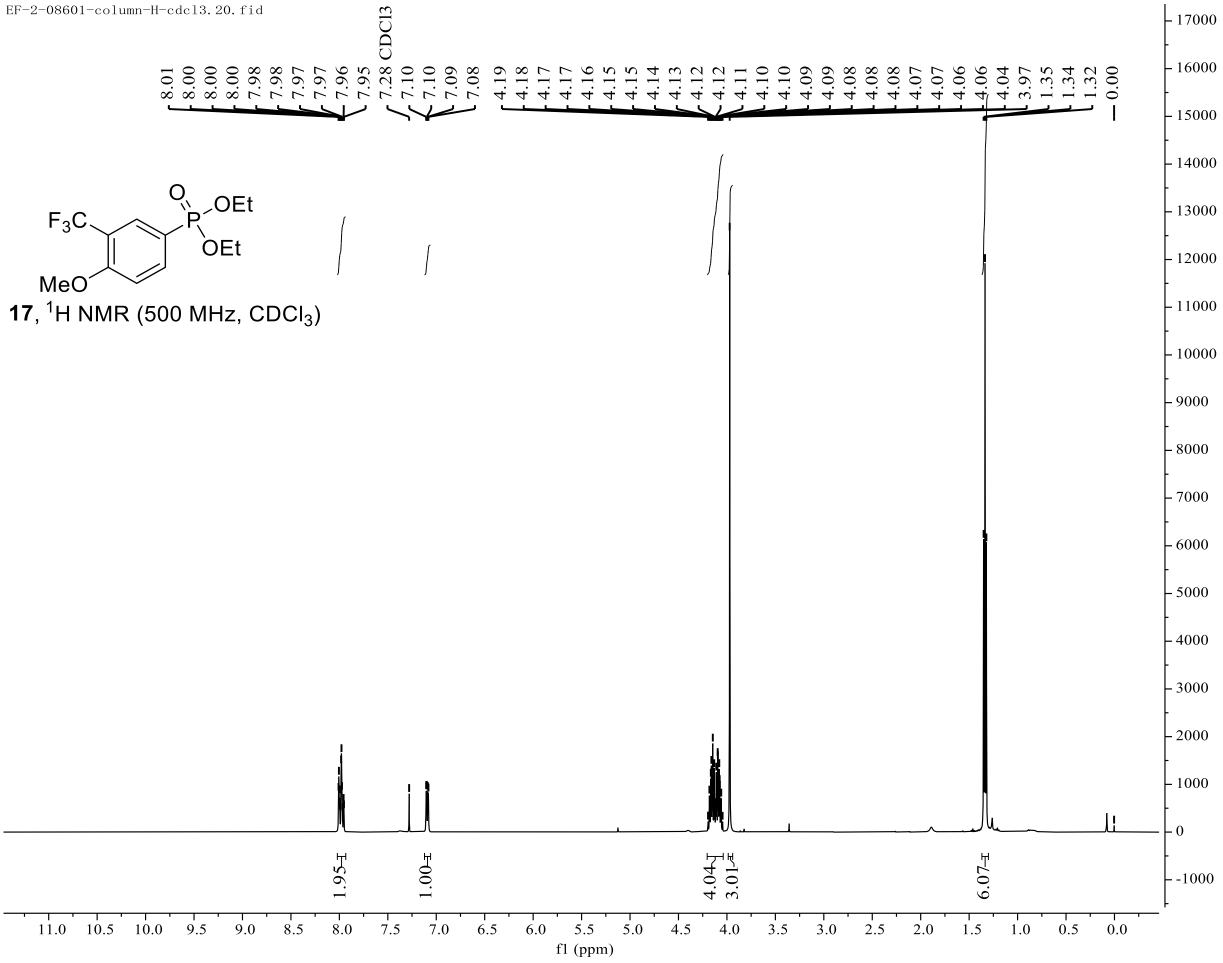

17, ^{1}H NMR (500 MHz, $CDCl_3$)

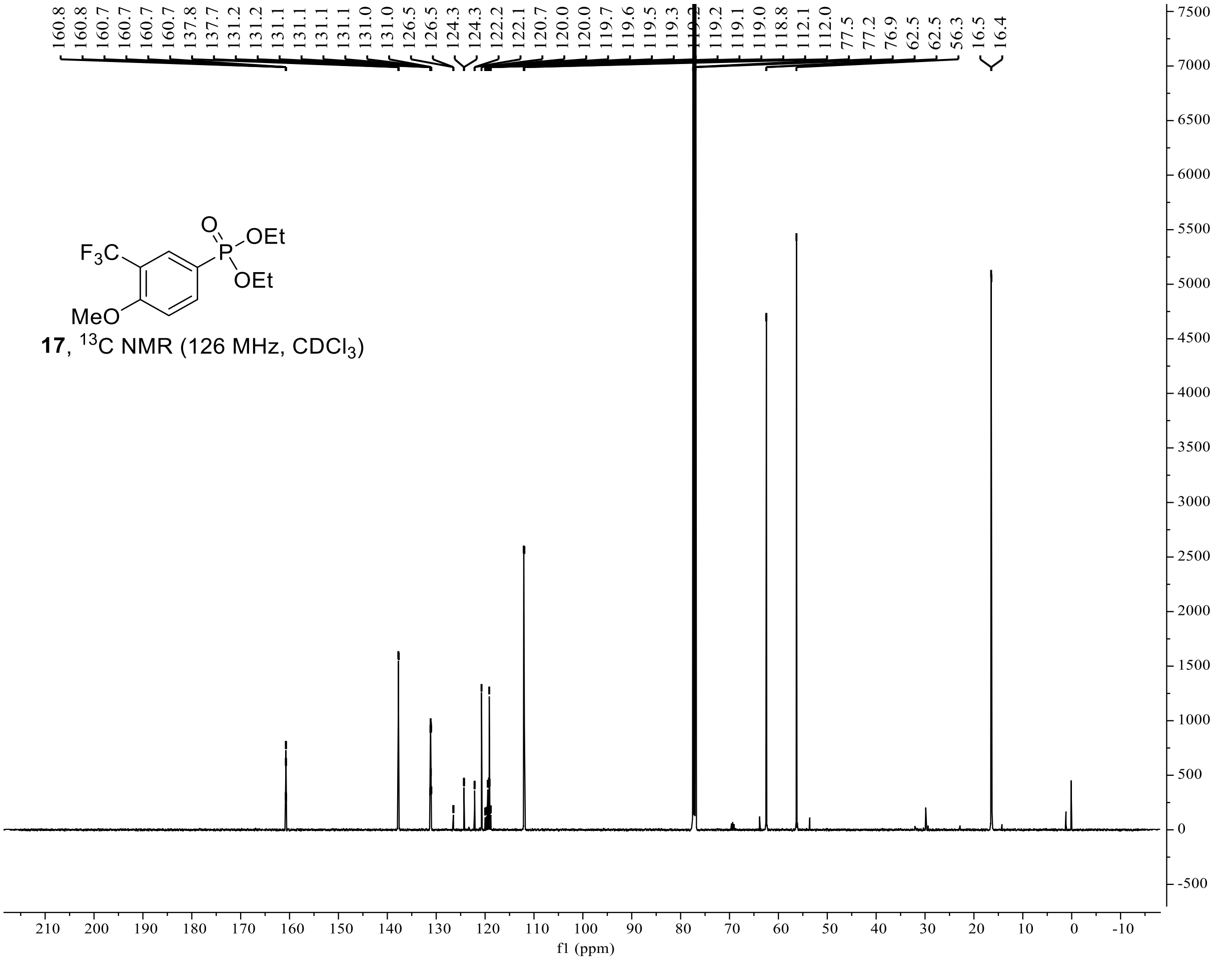

17, ^{13}C NMR (126 MHz, $CDCl_3$)

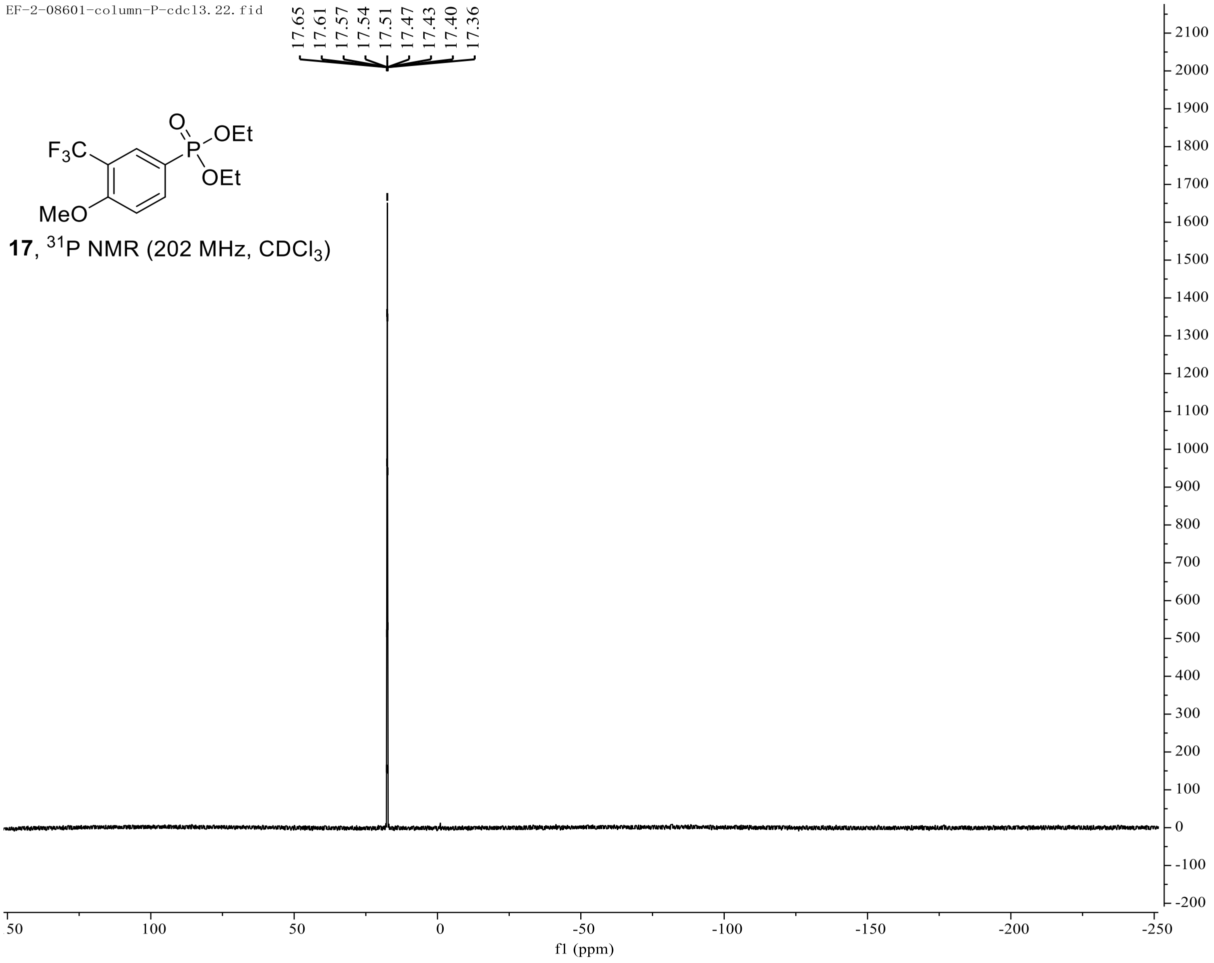
EF-2-08601-column-P-cdcl3.22.fid
17.65
17.61
17.57
17.54
17.51
17.47
17.43
17.40
17.36
OEt
OEt
F3C
MeO
17, 31P NMR (202 MHz, CDCl3)
f1 (ppm)

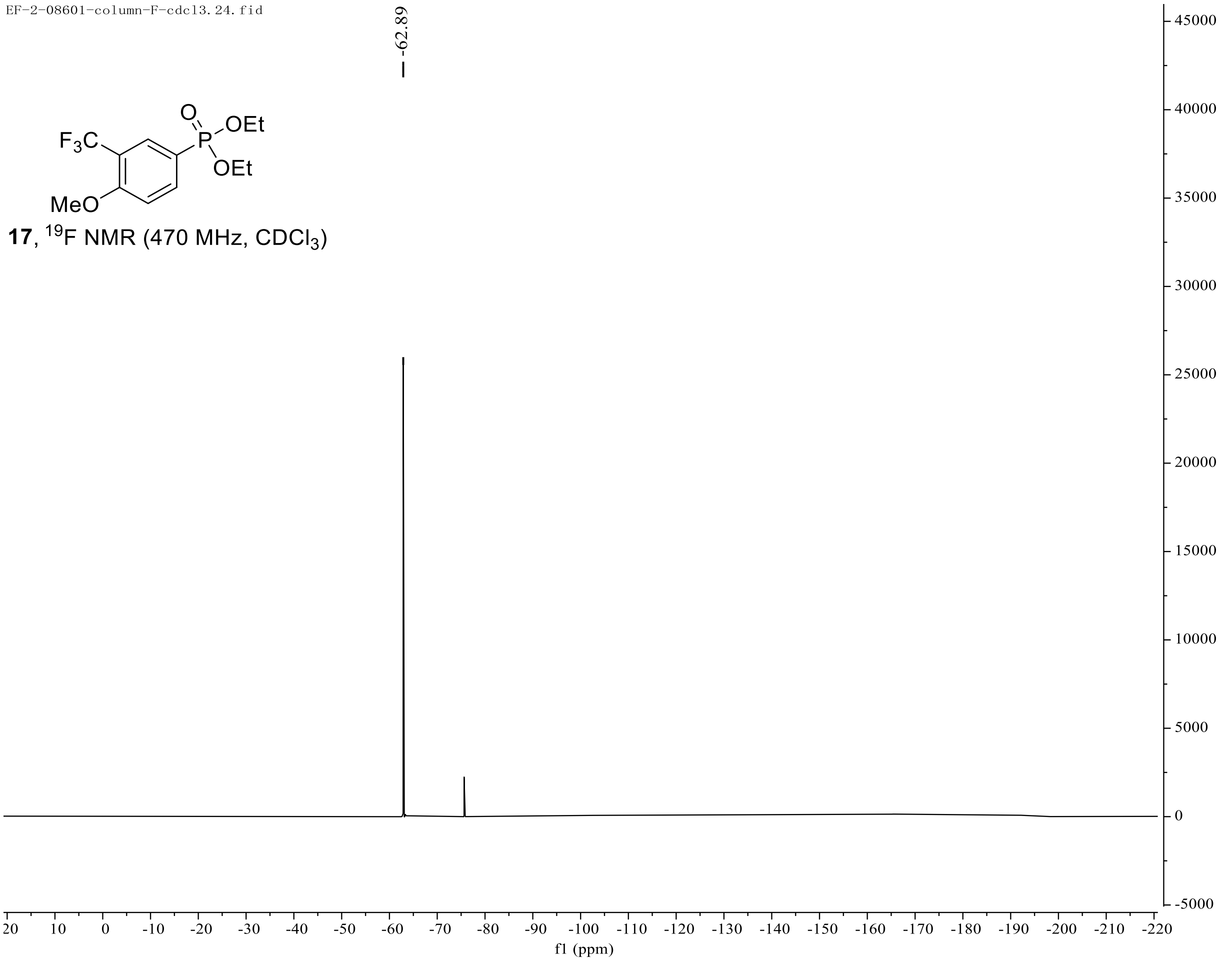
EF-2-08601-column-F-cdcl3.24.fid
-62.89
F3C
O
P
OEt
OEt
MeO
17, 19F NMR (470 MHz, CDCl3)
f1 (ppm)

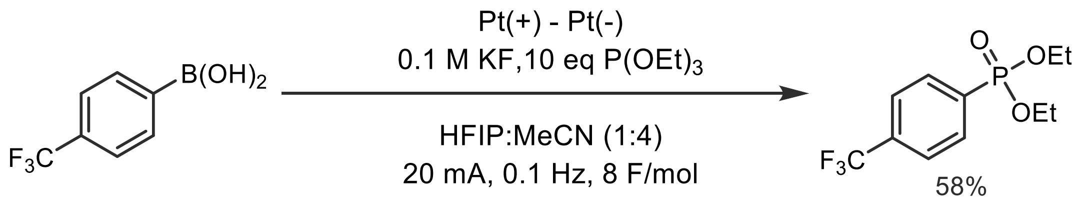
Pt(+) - Pt(-)
0.1 M KF,10 eq P(OEt)3
HFIP:MeCN (1:4)
20 mA, 0.1 Hz, 8 F/mol
B(OH)2
F3C
OEt
58%

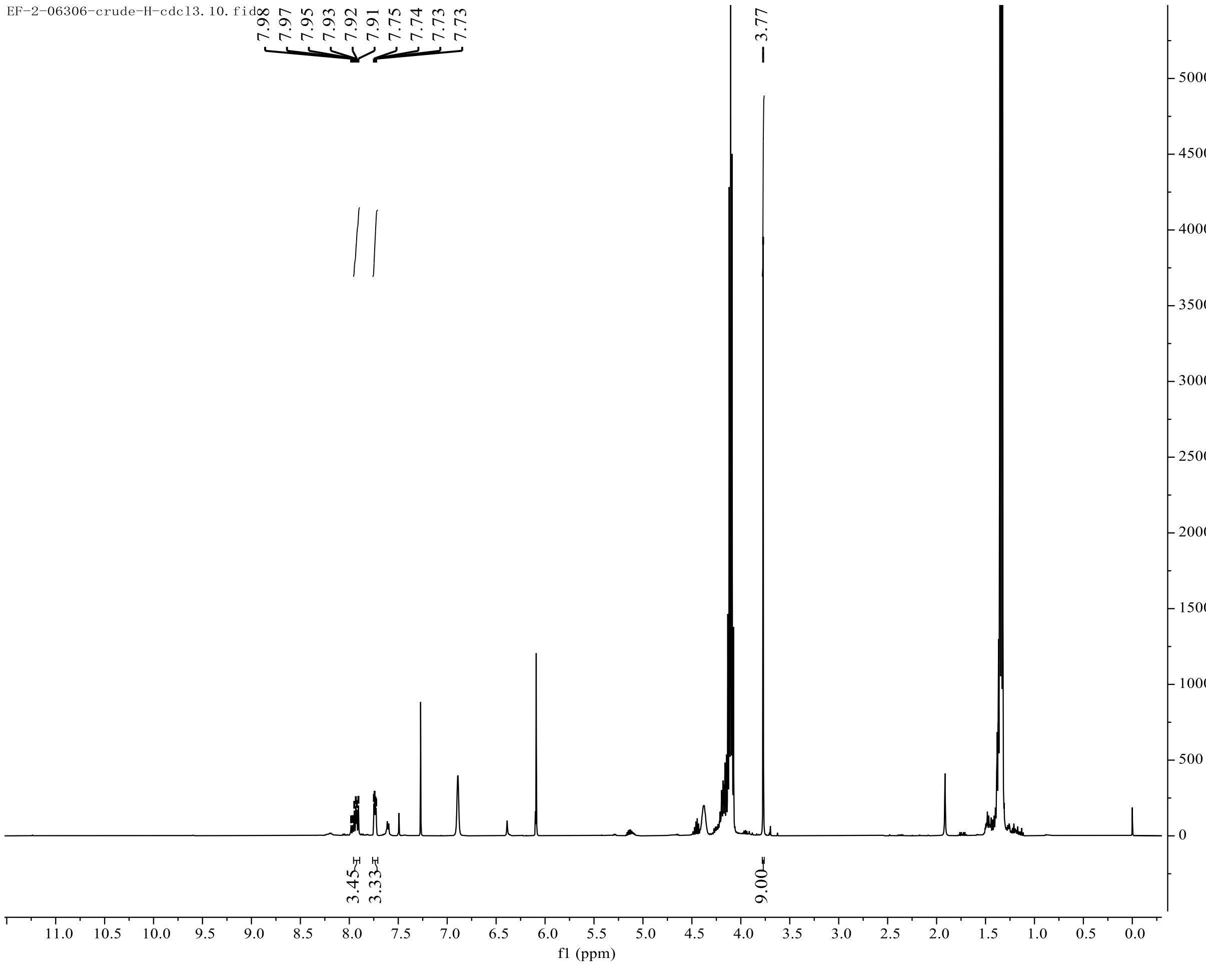
EF-2-06306-crude-H-cdcl3.10.fid
7.98
7.97
7.95
7.93
7.92
7.91
7.75
7.74
7.73
7.73
3.77
3.45
3.33
9.00
f1 (ppm)

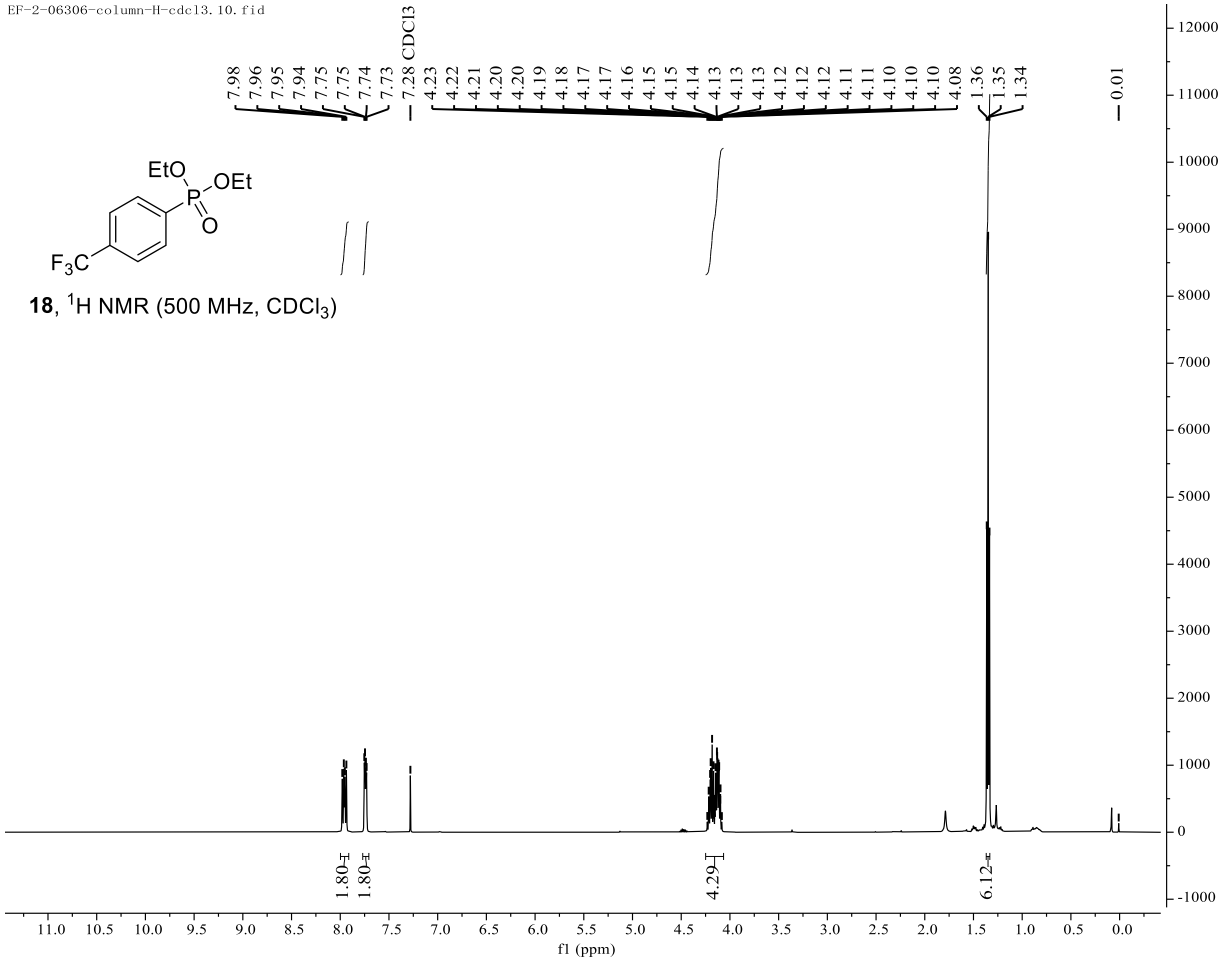

18, ^{1}H NMR (500 MHz, $CDCl_3$)

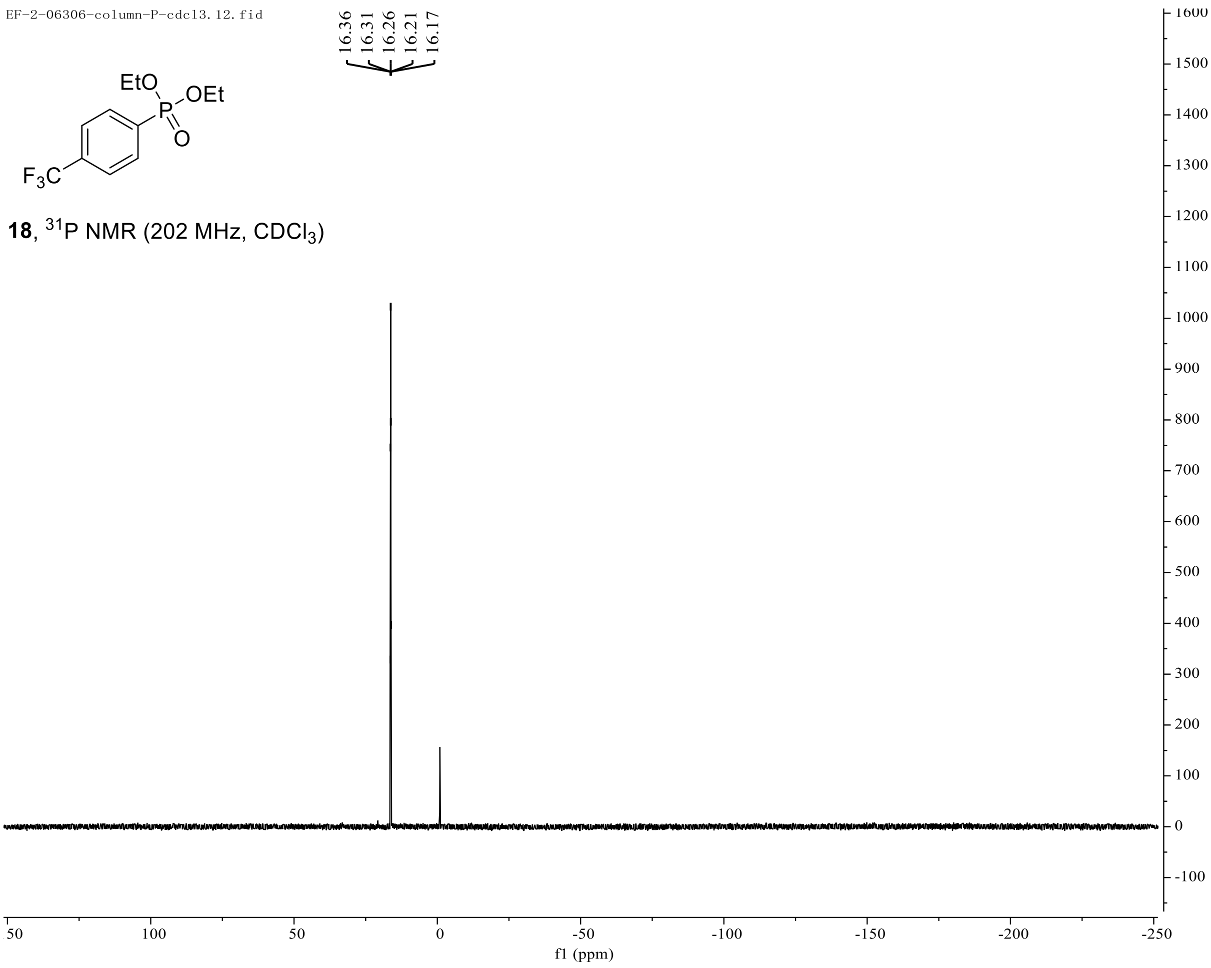

18, ^{31}P NMR (202 MHz, $CDCl_3$)

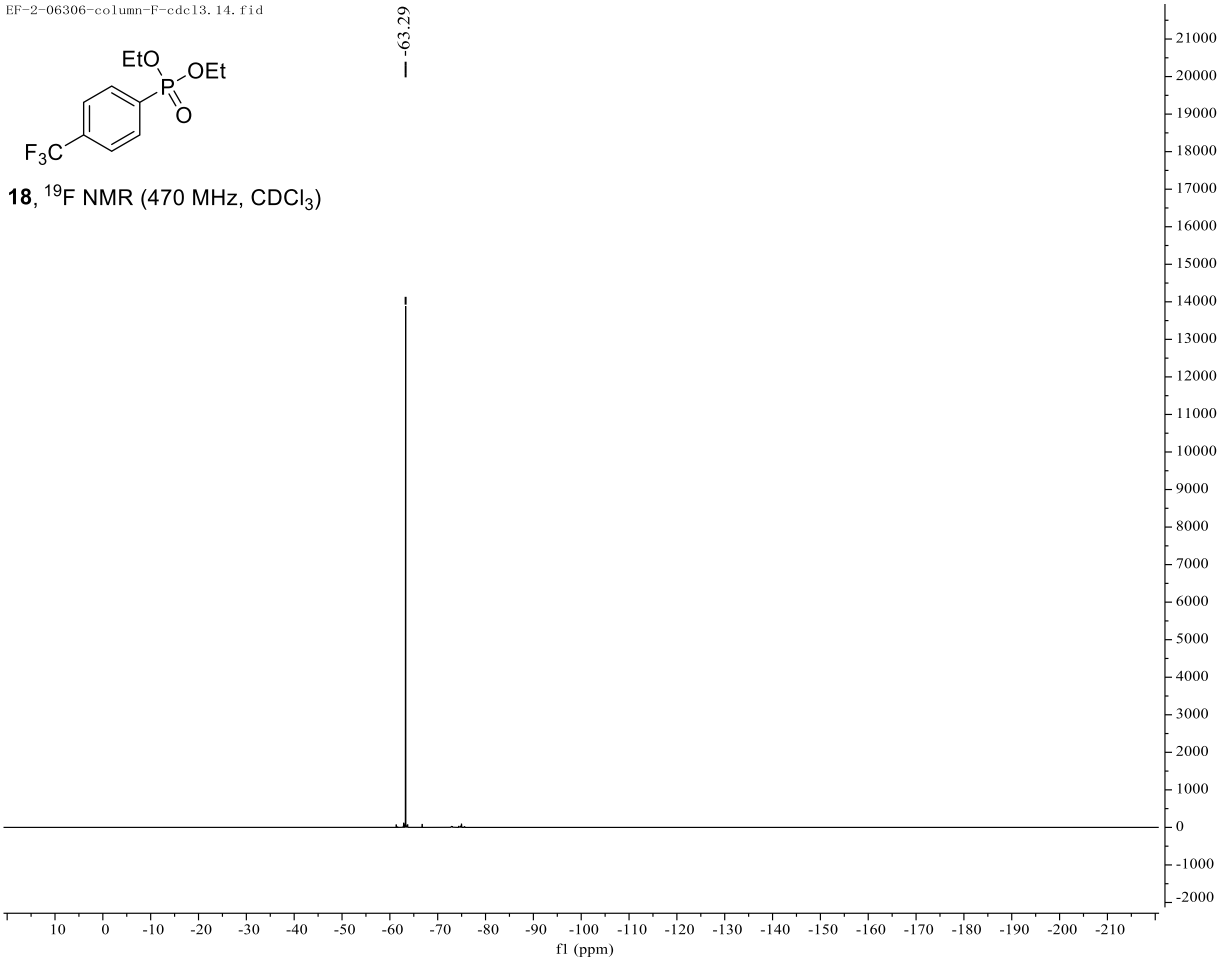
EF-2-06306-column-F-cdcl3.14.fid
-63.29
EtO
OEt
P
O
F3C
18, 19F NMR (470 MHz, CDCl3)
f1 (ppm)

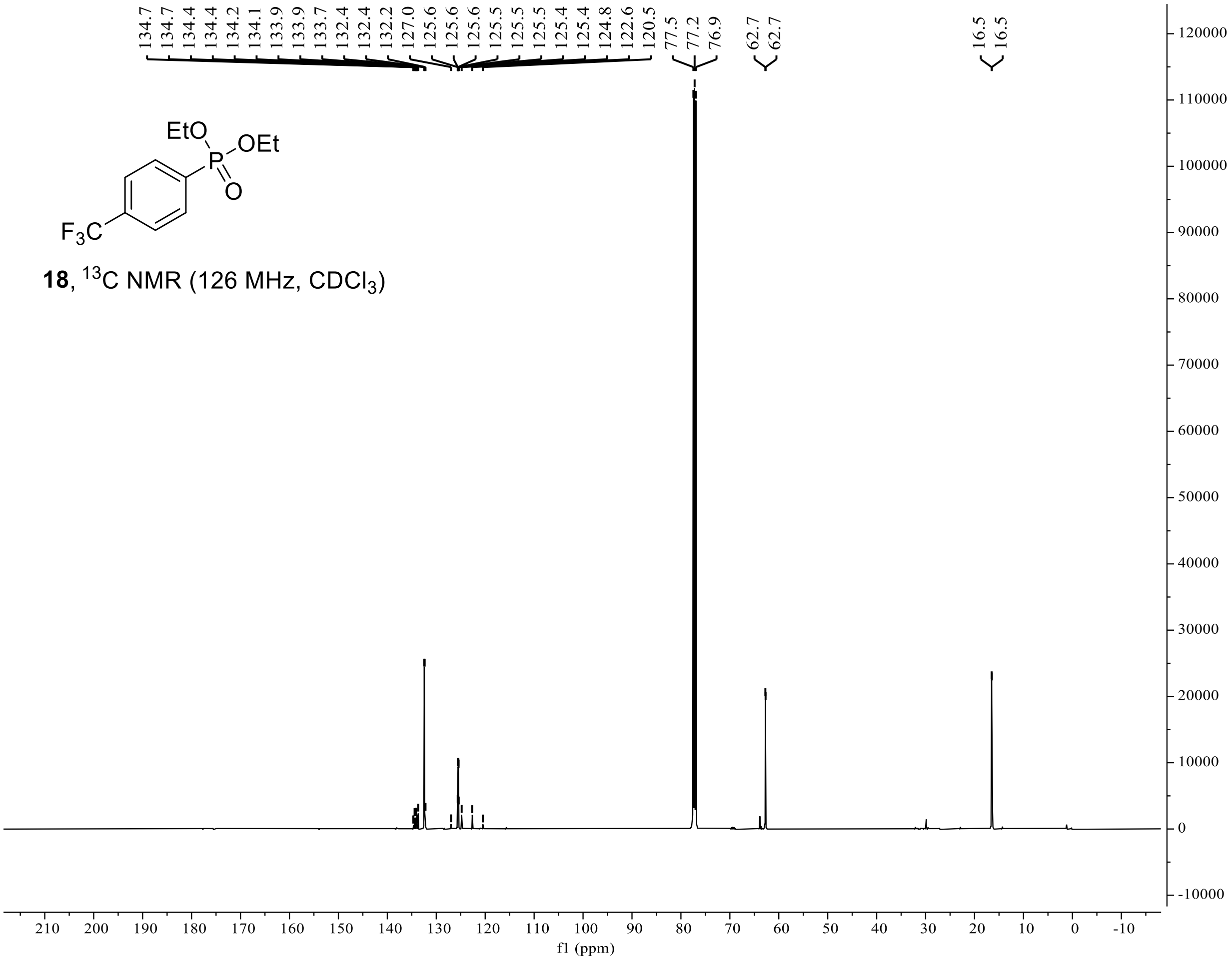
134.7
134.7
134.4
134.4
134.2
134.1
133.9
133.9
133.7
132.4
132.4
132.2
127.0
125.6
125.6
125.6
125.5
125.5
125.5
125.4
125.4
124.8
122.6
120.5
77.5
77.2
76.9
62.7
62.7
16.5
16.5
EtO
P
OEt
O
F3C
18, 13C NMR (126 MHz, CDCl3)
f1 (ppm)

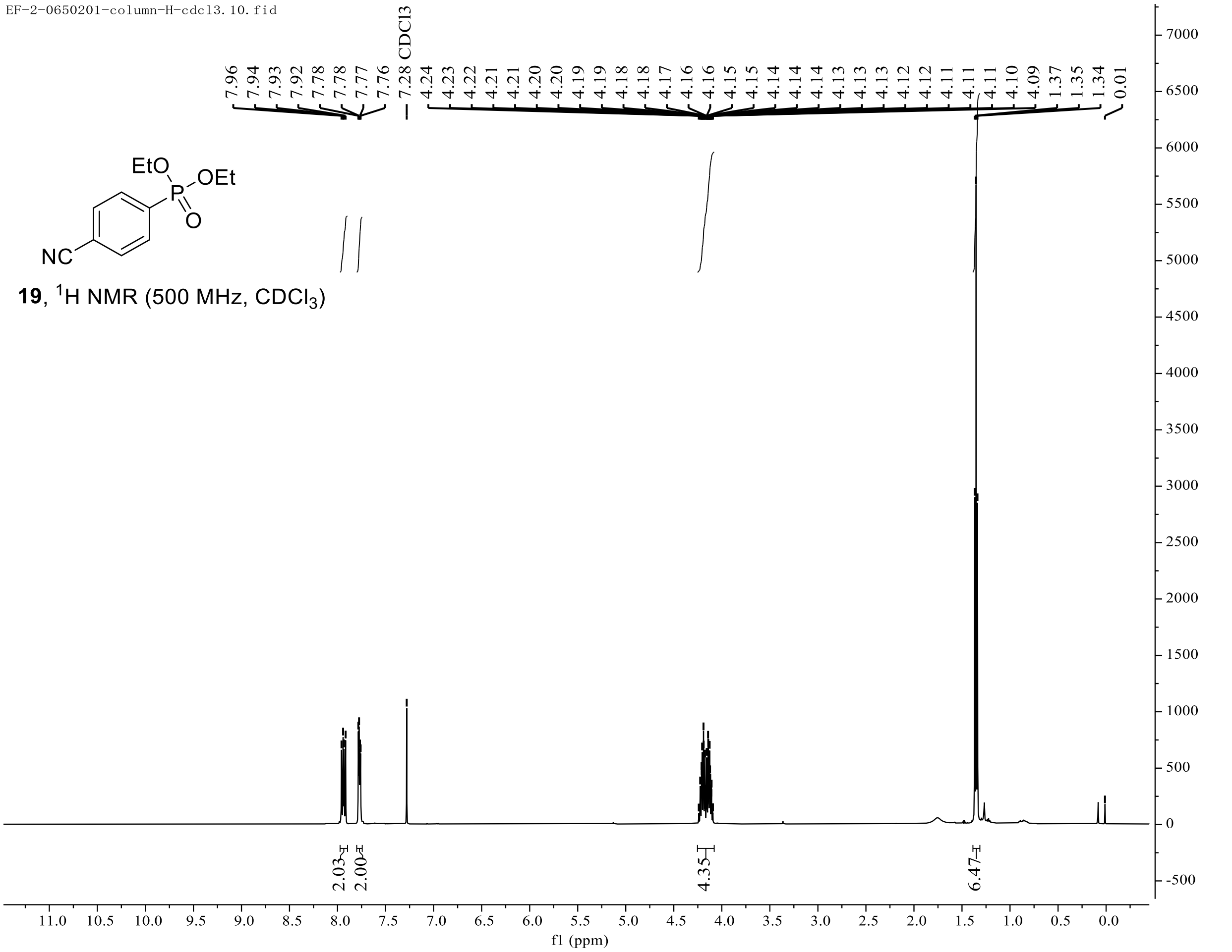

19, ^{1}H NMR (500 MHz, $CDCl_3$)

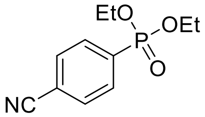

19, ^{31}P NMR (202 MHz, $CDCl_3$)

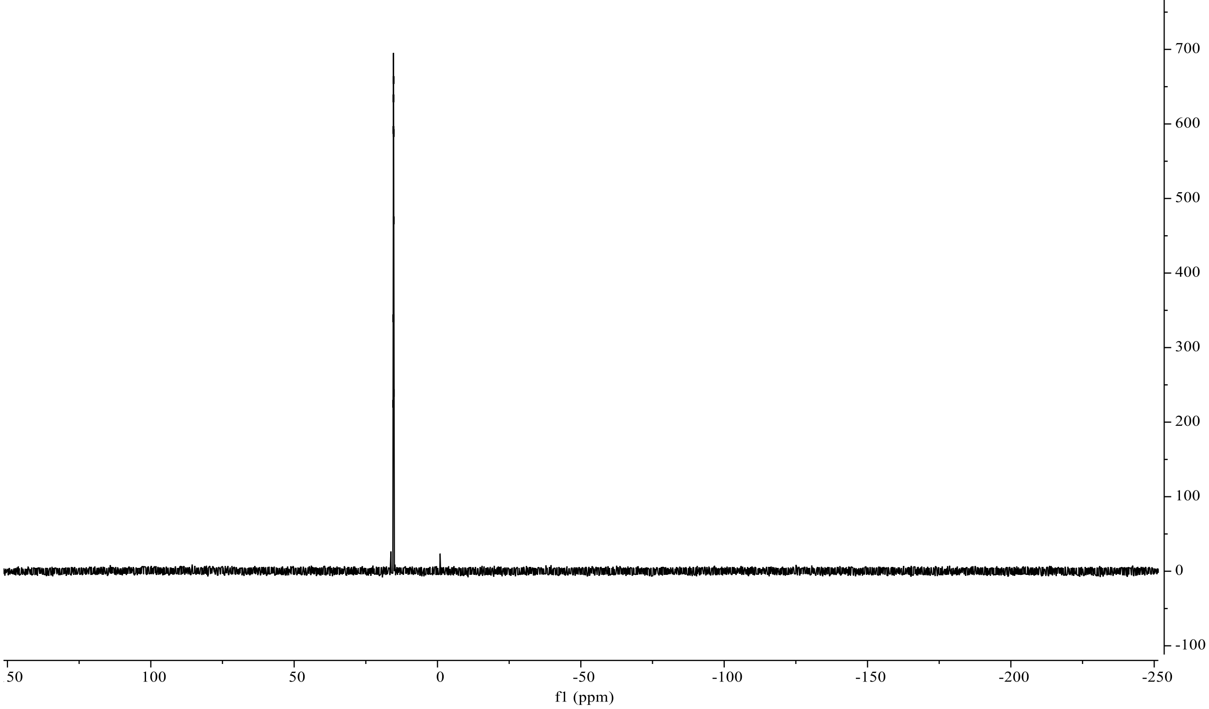

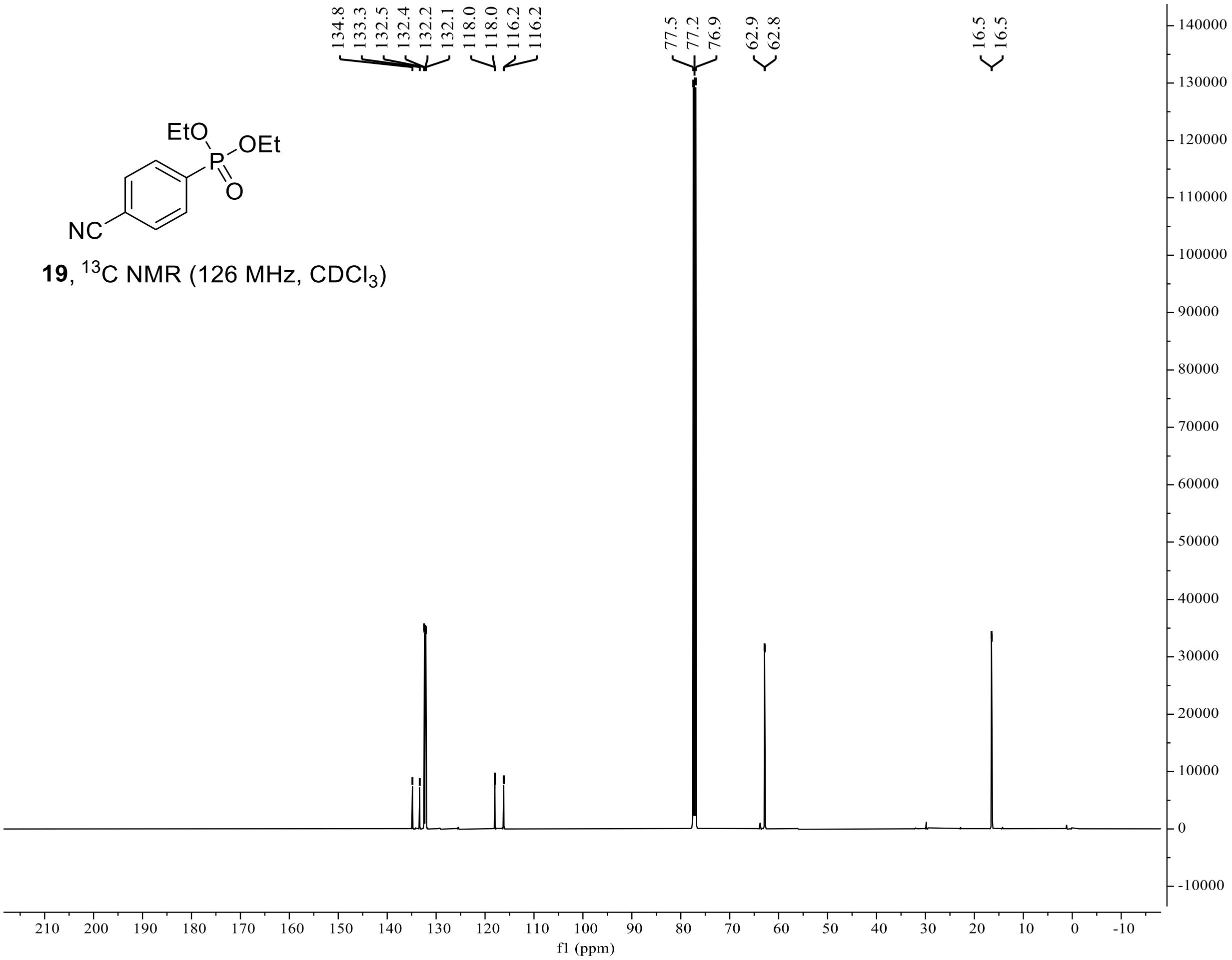

134.8
133.3
132.5
132.4
132.2
132.1
118.0
118.0
116.2
116.2
77.5
77.2
76.9
62.9
62.8
16.5
16.5
EtO
OEt
P
O
NC
19, ^{13}C NMR (126 MHz, $CDCl_3$)
140000
130000
120000
110000
100000
90000
80000
70000
60000
50000
40000
30000
20000
10000
0
-10000
210 200 190 180 170 160 150 140 130 120 110 100 90 80 70 60 50 40 30 20 10 0 -10
f1 (ppm)

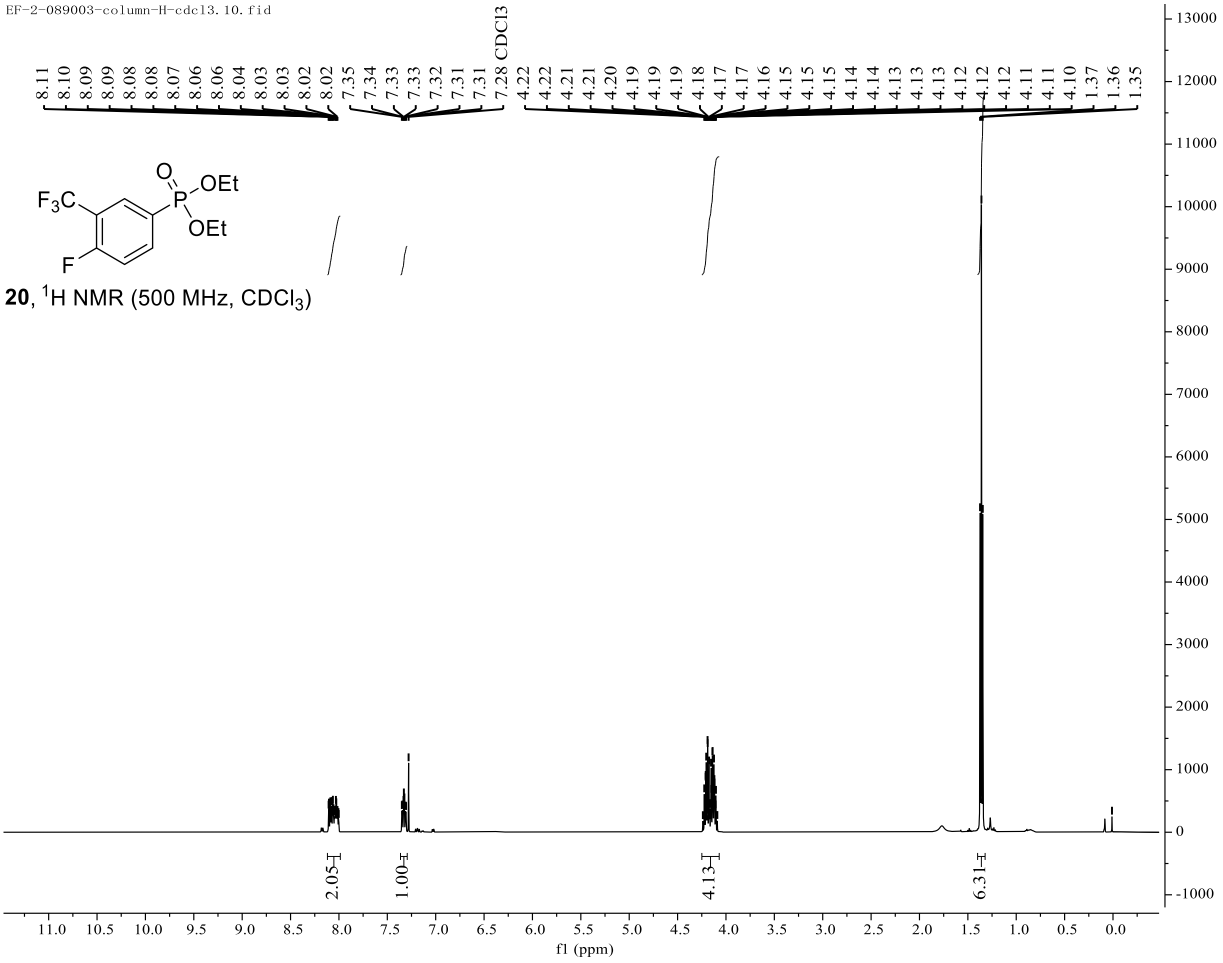
EF-2-089003-column-H-cdcl3.10.fid
F3C
O
P
OEt
OEt
F
20, 1H NMR (500 MHz, CDCl3)
f1 (ppm)

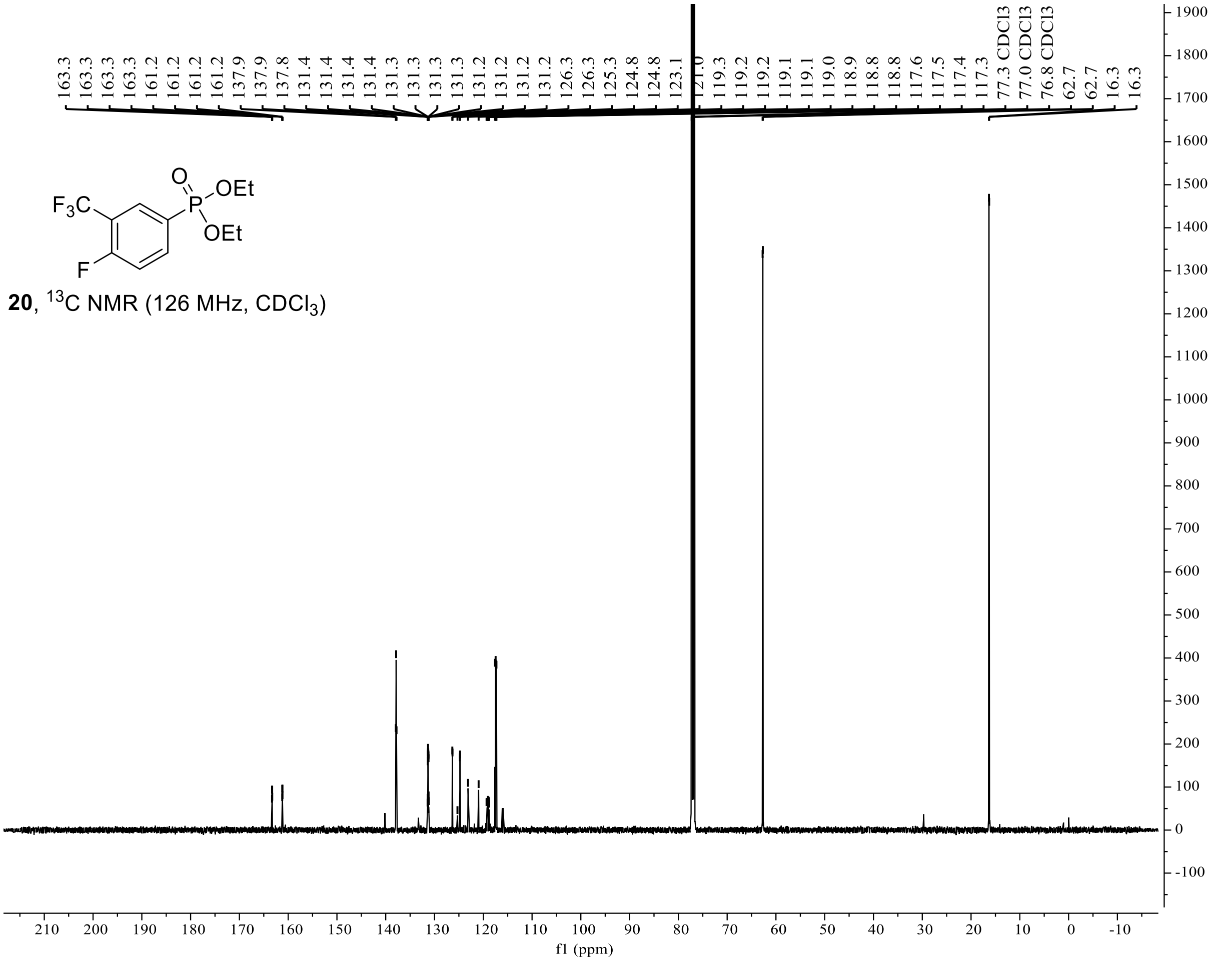

20, ^{13}C NMR (126 MHz, $CDCl_3$)

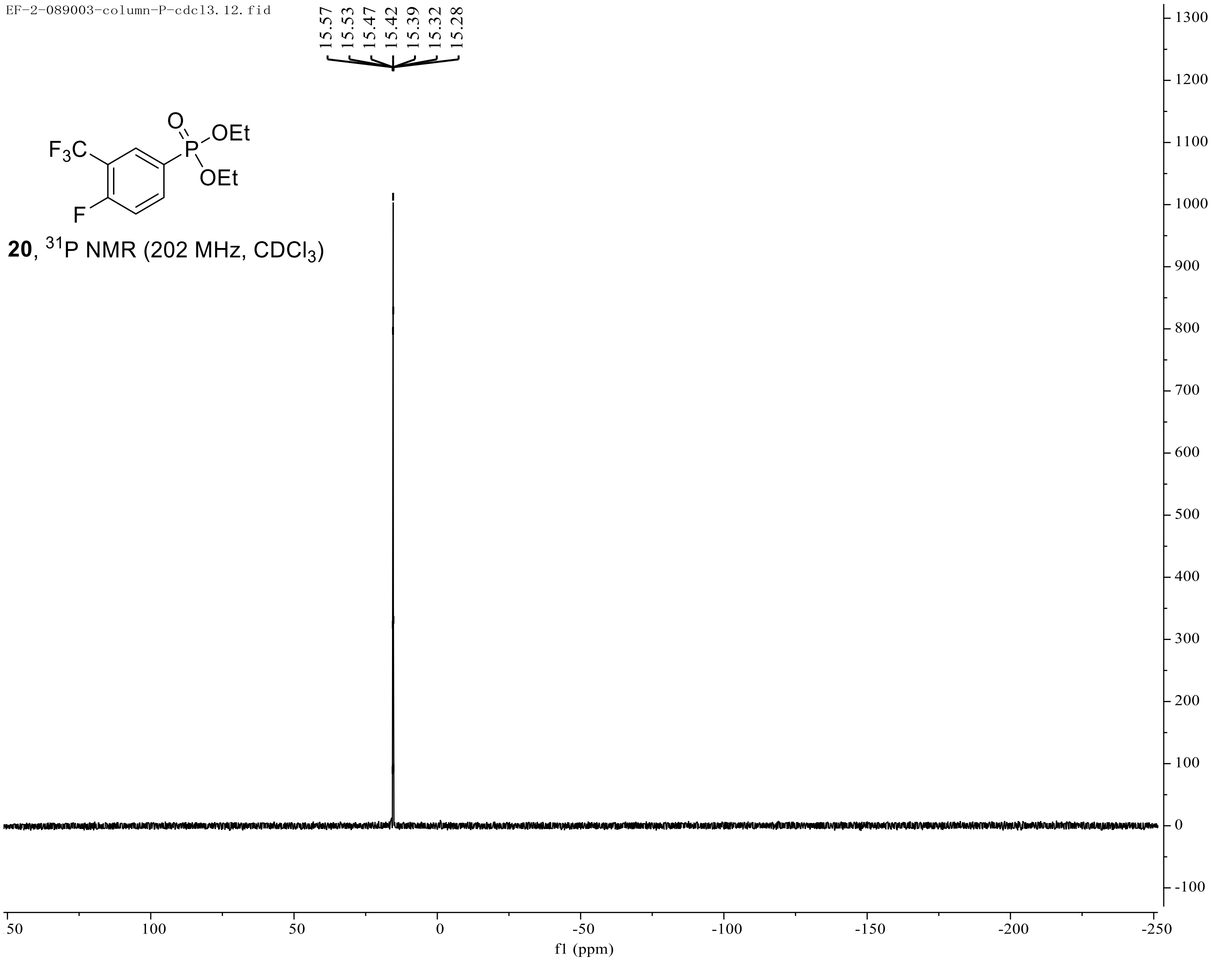

20, ^{31}P NMR (202 MHz, $CDCl_3$)

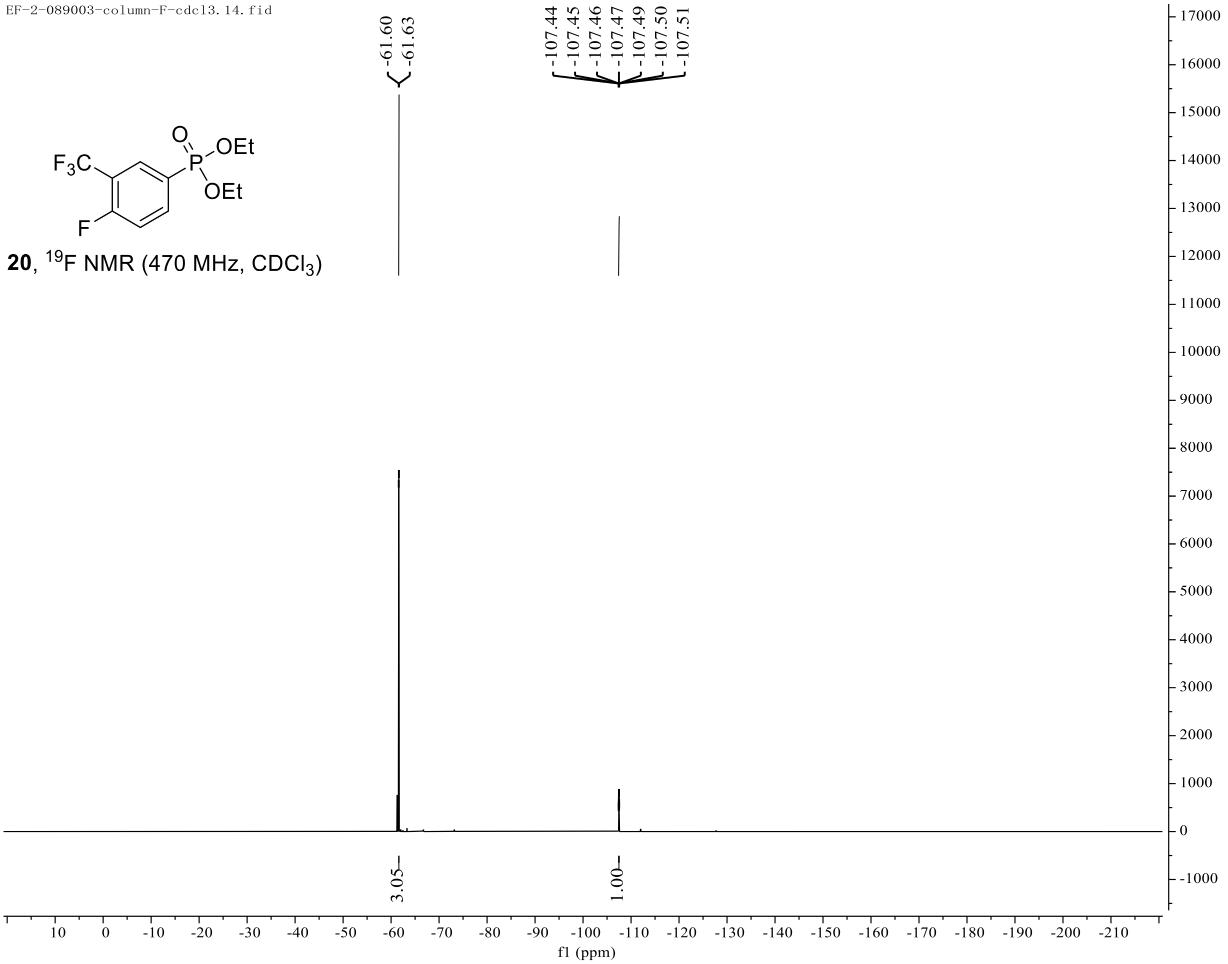
EF-2-089003-column-F-cdcl3.14.fid
-61.60
-61.63
-107.44
-107.45
-107.46
-107.47
-107.49
-107.50
-107.51
O
P
OEt
OEt
F3C
F
20, 19F NMR (470 MHz, CDCl3)
3.05
1.00
f1 (ppm)

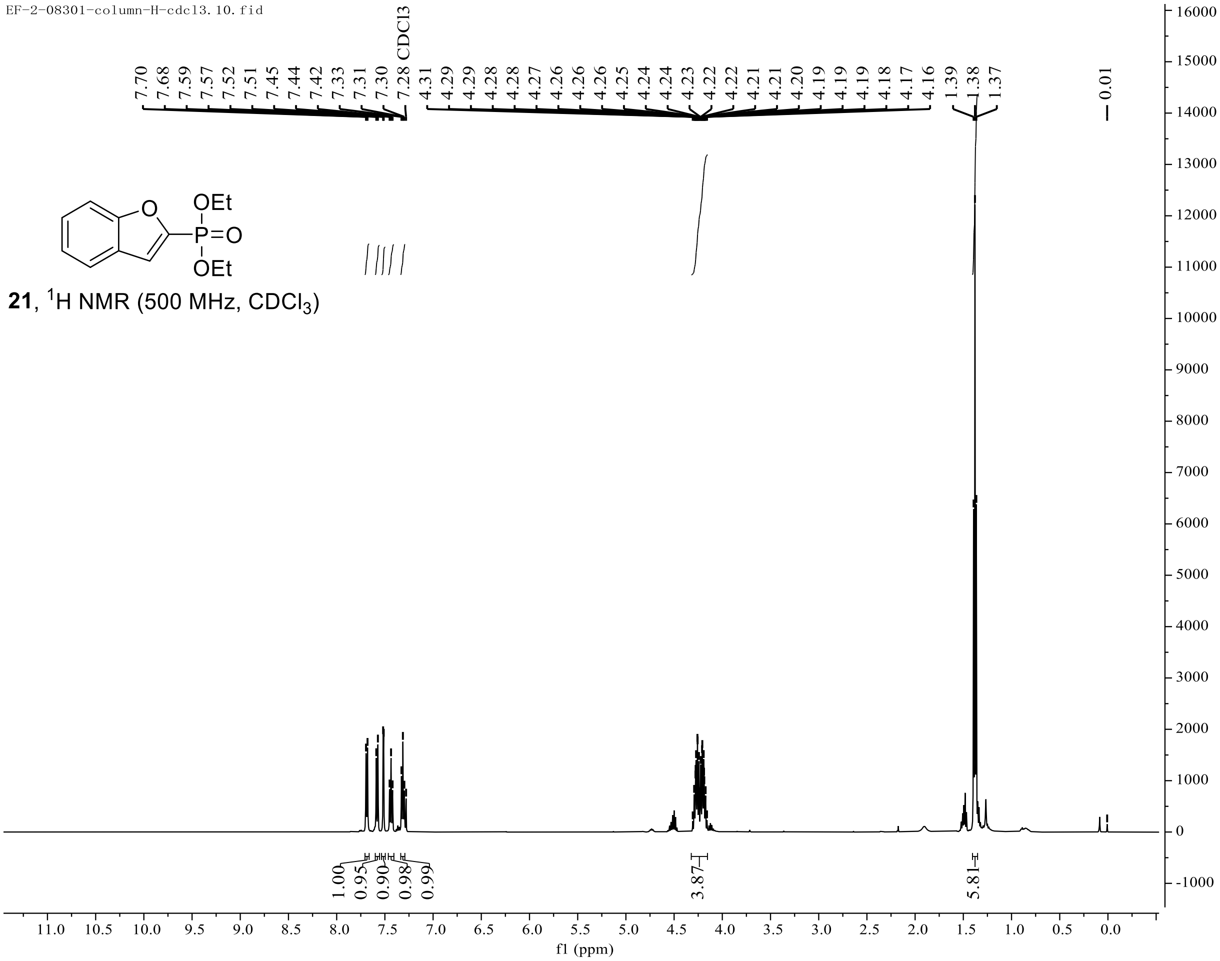

21, ^{1}H NMR (500 MHz, $CDCl_3$)

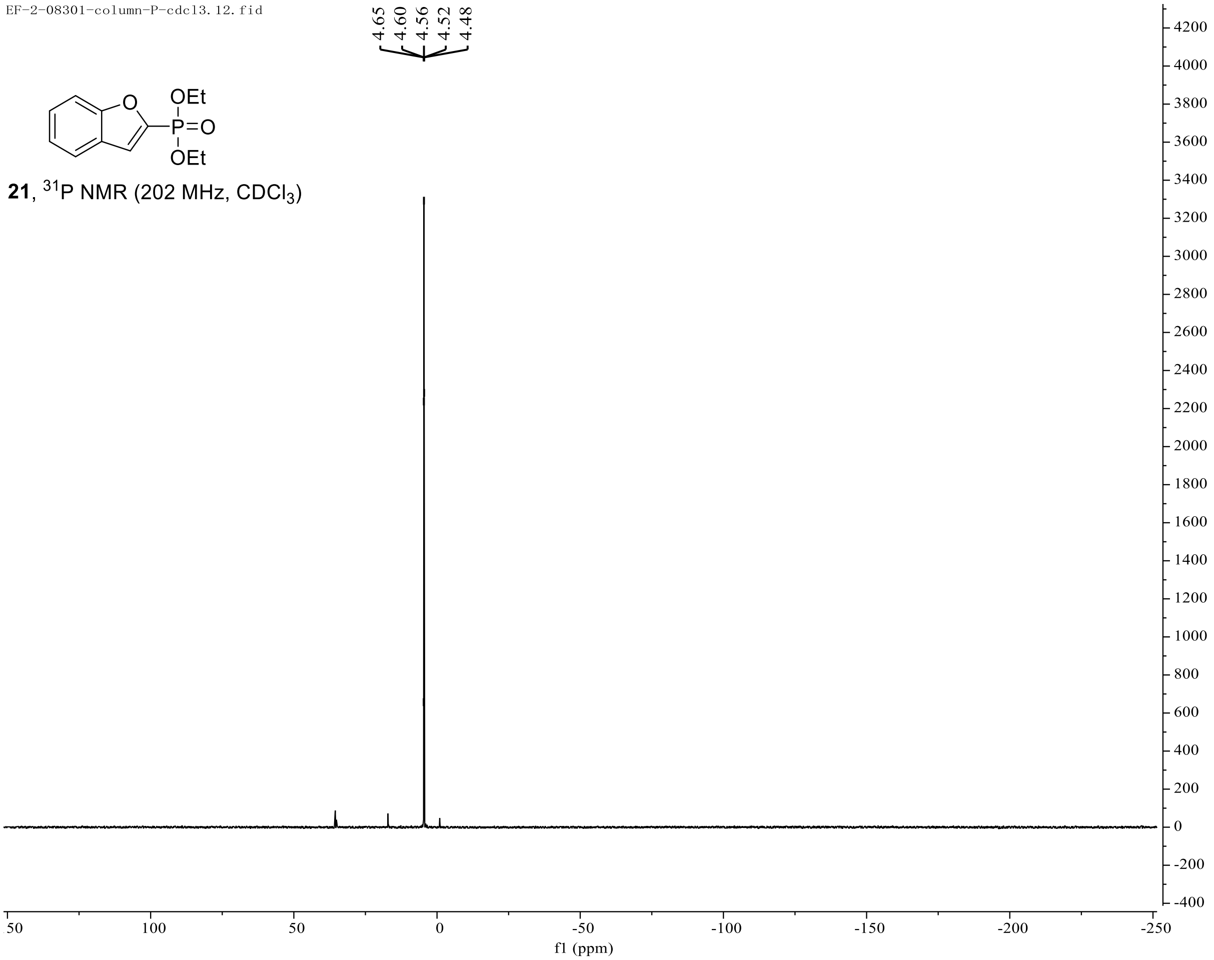

21, ^{31}P NMR (202 MHz, $CDCl_3$)

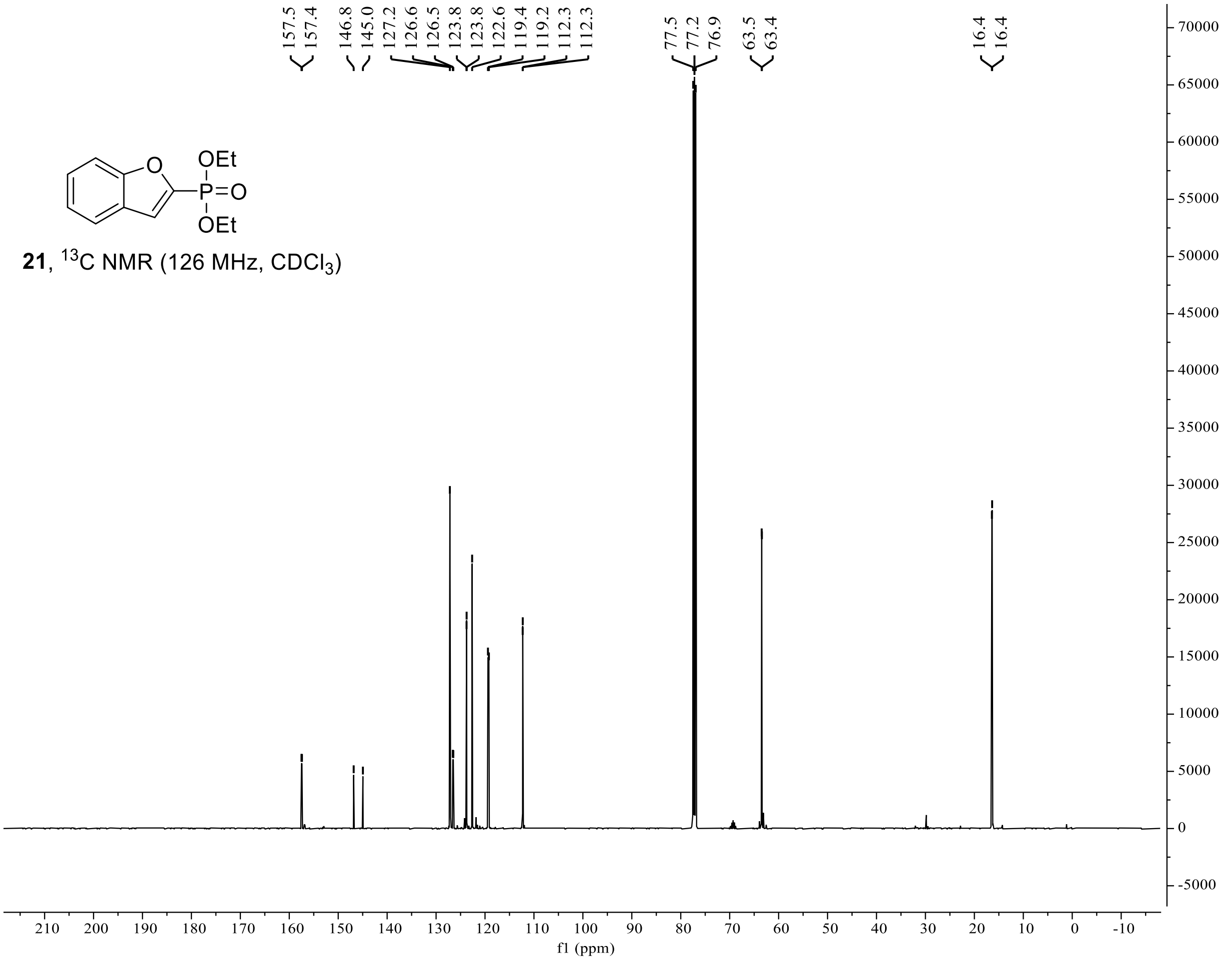

21, ^{13}C NMR (126 MHz, $CDCl_3$)

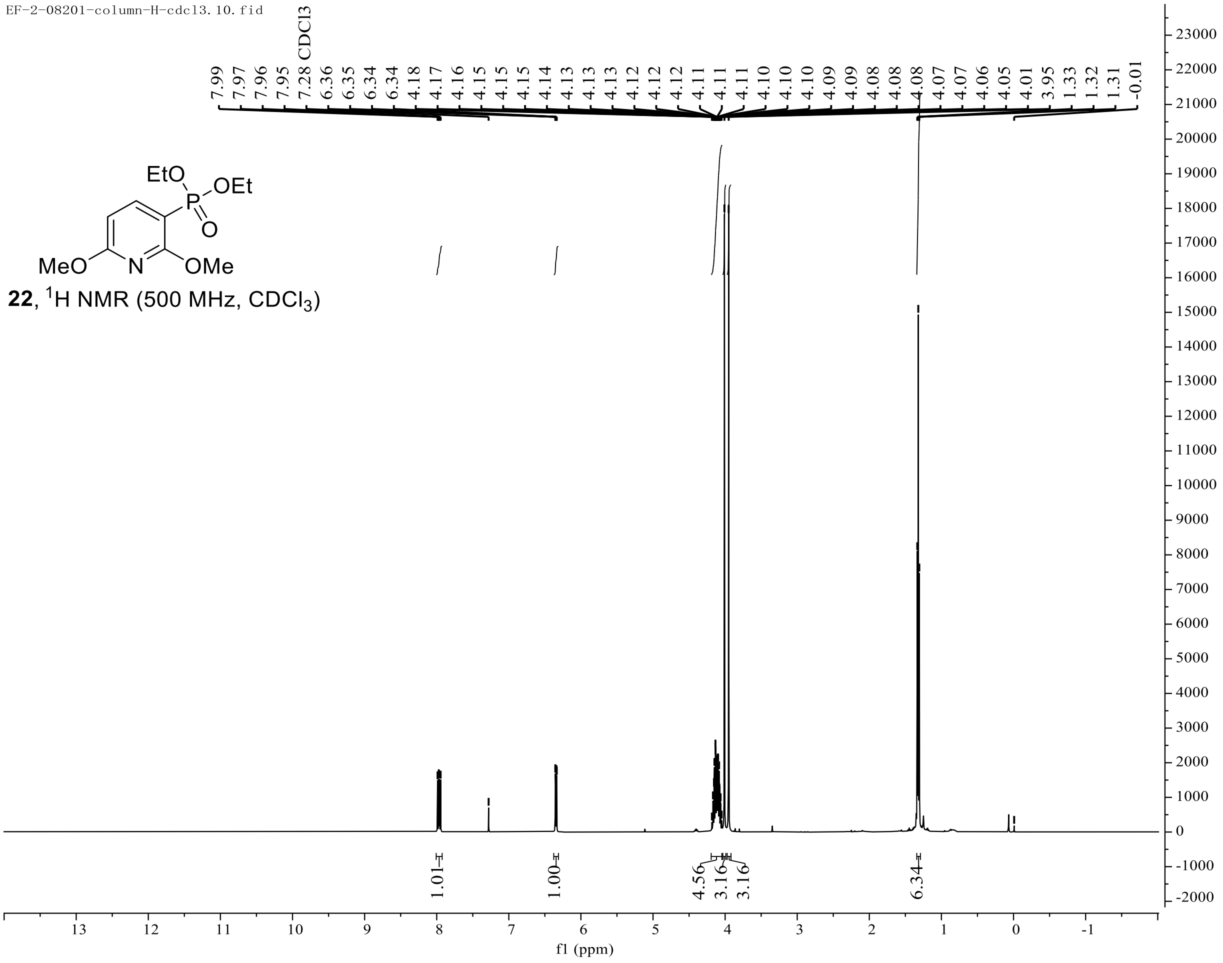
EF-2-08201-column-H-cdcl3.10.fid
EtO
OEt
P
O
MeO
N
OMe
22, ¹H NMR (500 MHz, CDCl₃)
f1 (ppm)

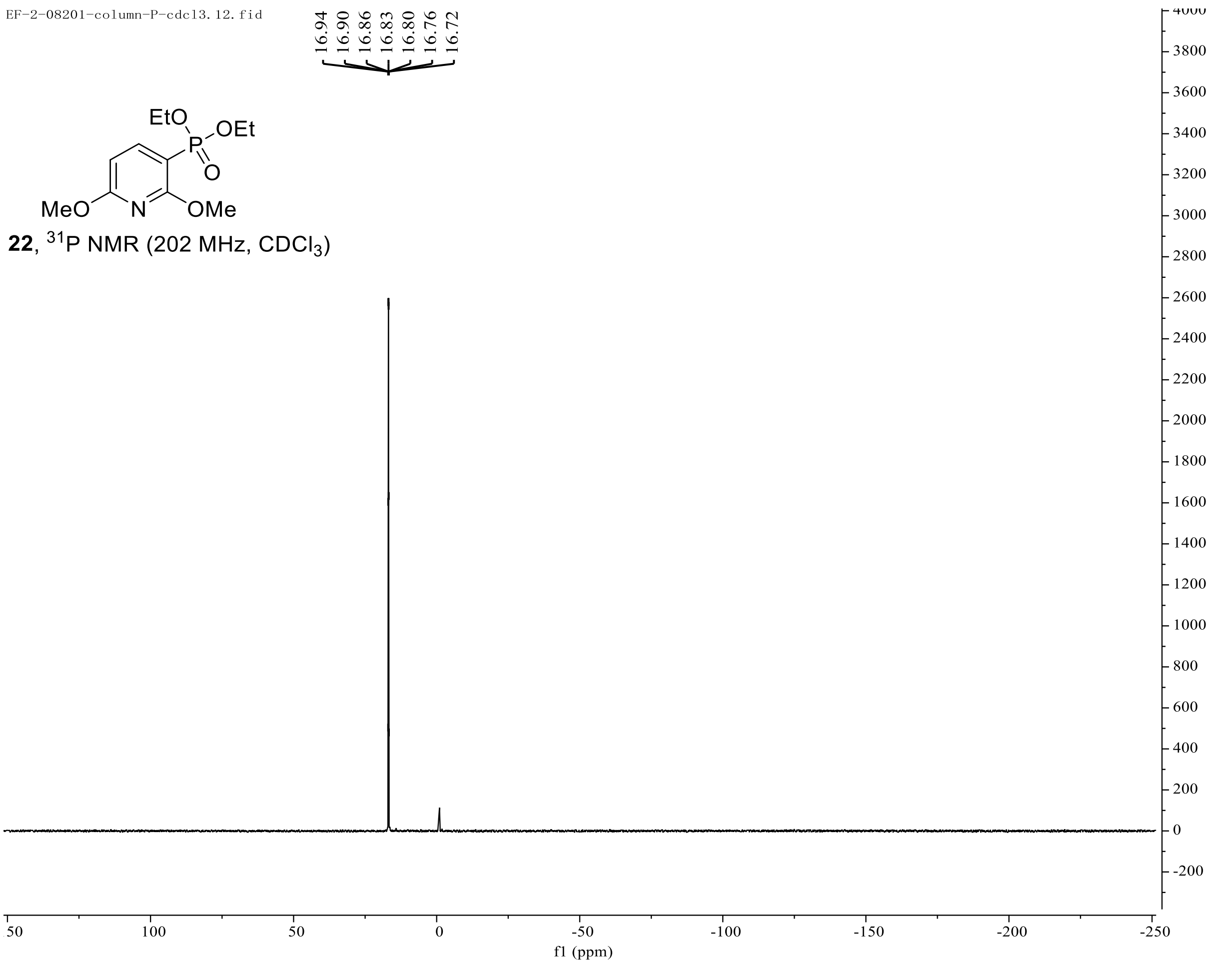

22, ^{31}P NMR (202 MHz, $CDCl_3$)

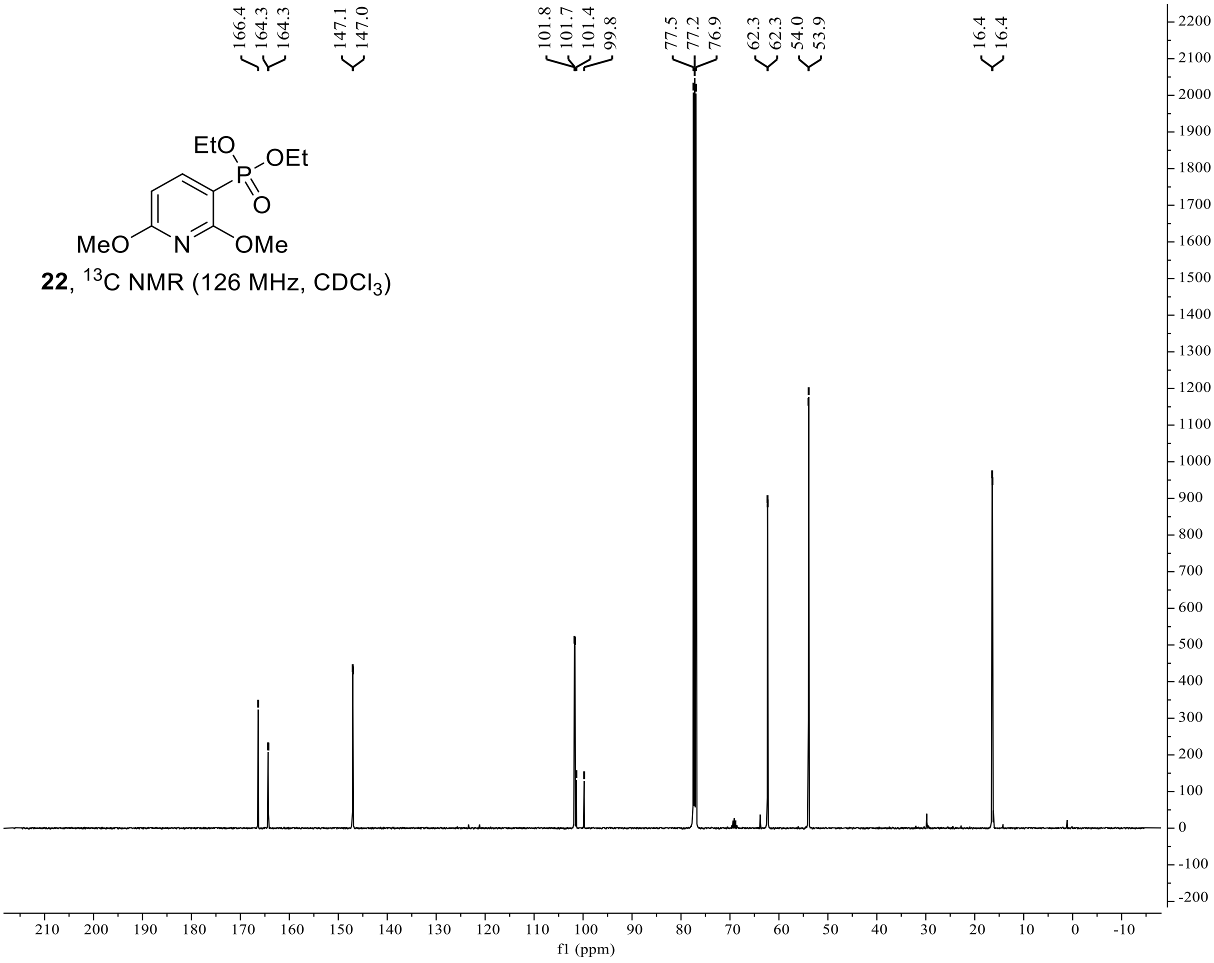

22, ^{13}C NMR (126 MHz, $CDCl_3$)

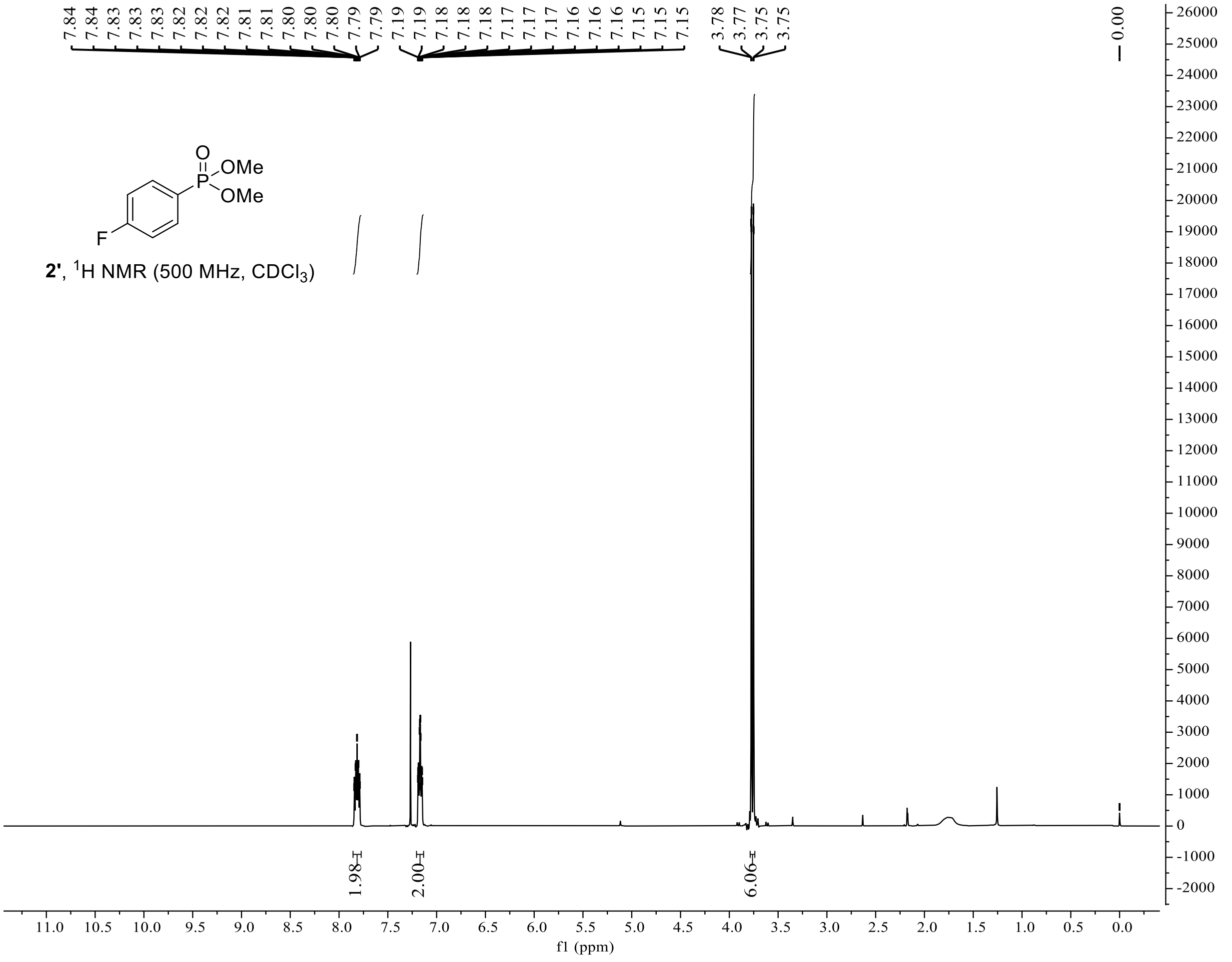

7.84
7.84
7.83
7.83
7.83
7.82
7.82
7.82
7.81
7.80
7.80
7.80
7.79
7.79
7.19
7.19
7.18
7.18
7.18
7.17
7.17
7.17
7.16
7.16
7.16
7.15
7.15
7.15
3.78
3.77
3.75
3.75
0.00
OMe
OMe
F
2', ¹H NMR (500 MHz, CDCl3)
1.98
2.00
6.06
f1 (ppm)

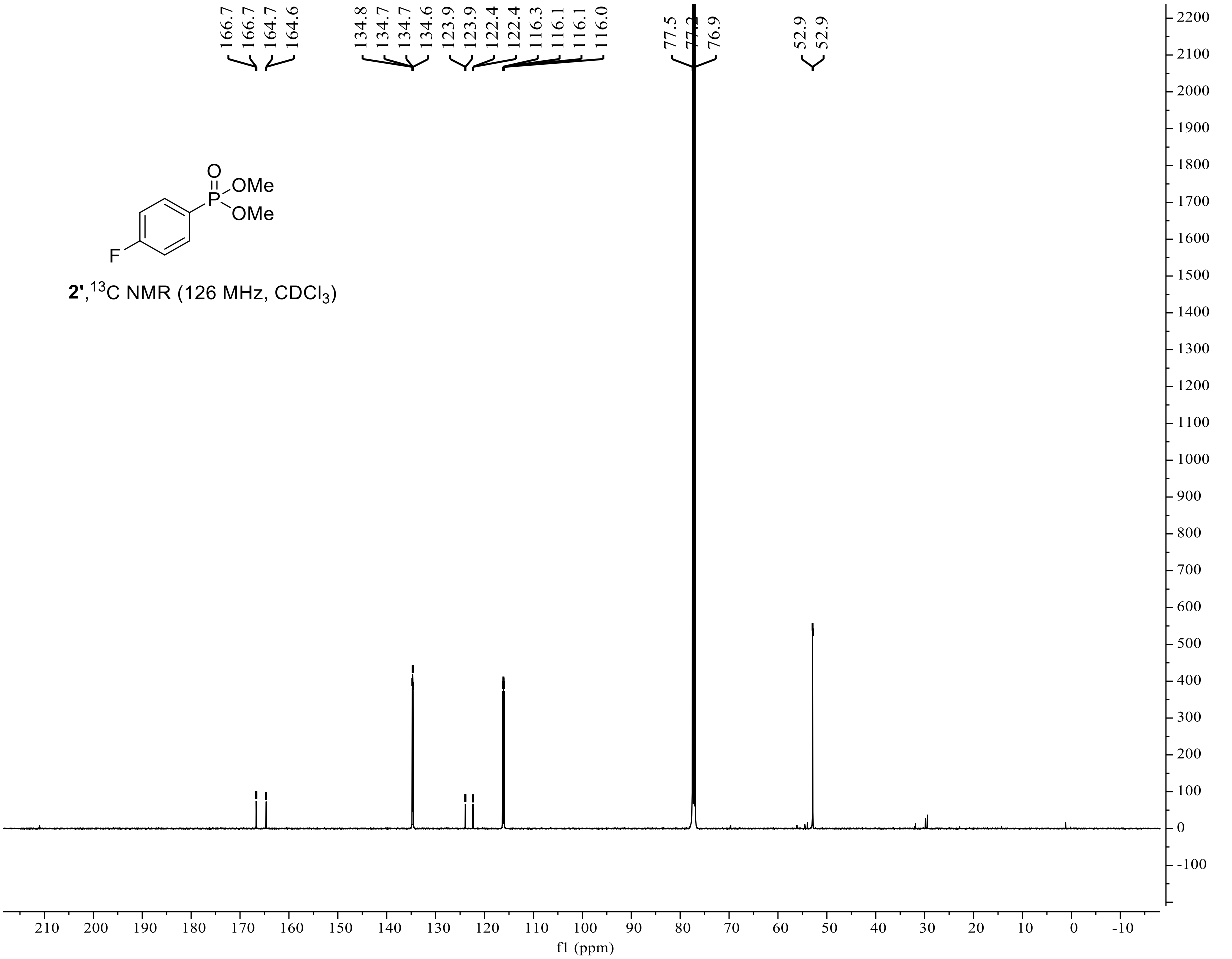
166.7
166.7
164.7
164.6
134.8
134.7
134.7
134.6
123.9
123.9
122.4
122.4
116.3
116.1
116.1
116.0
77.5
77.2
76.9
52.9
52.9
OMe
OMe
F
2',13C NMR (126 MHz, CDCl3)
f1 (ppm)

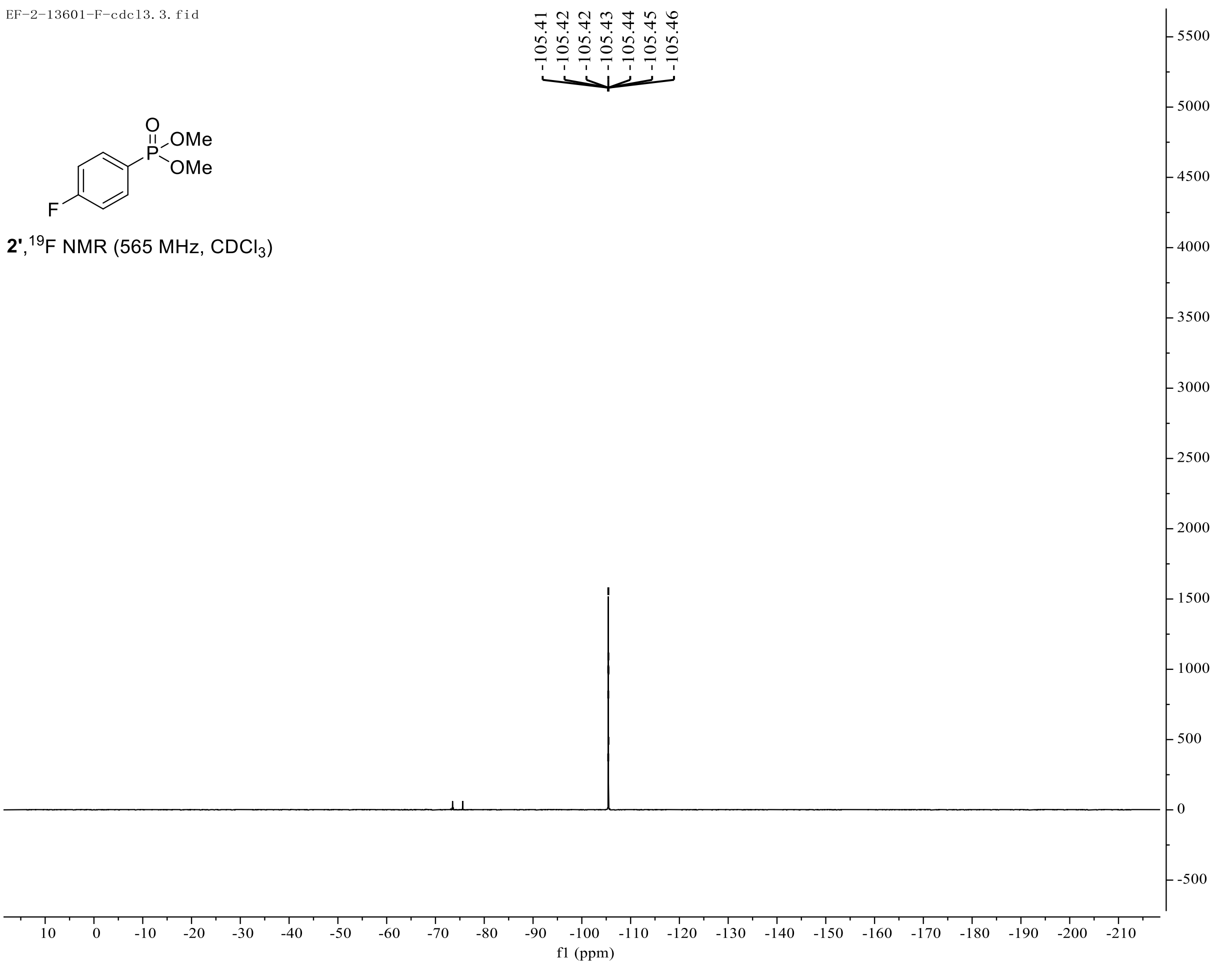

EF-2-13601-F-cdcl3.3.fid
-105.41
-105.42
-105.42
-105.43
-105.44
-105.45
-105.46
OMe
OMe
F
2',¹⁹F NMR (565 MHz, CDCl3)
f1 (ppm)

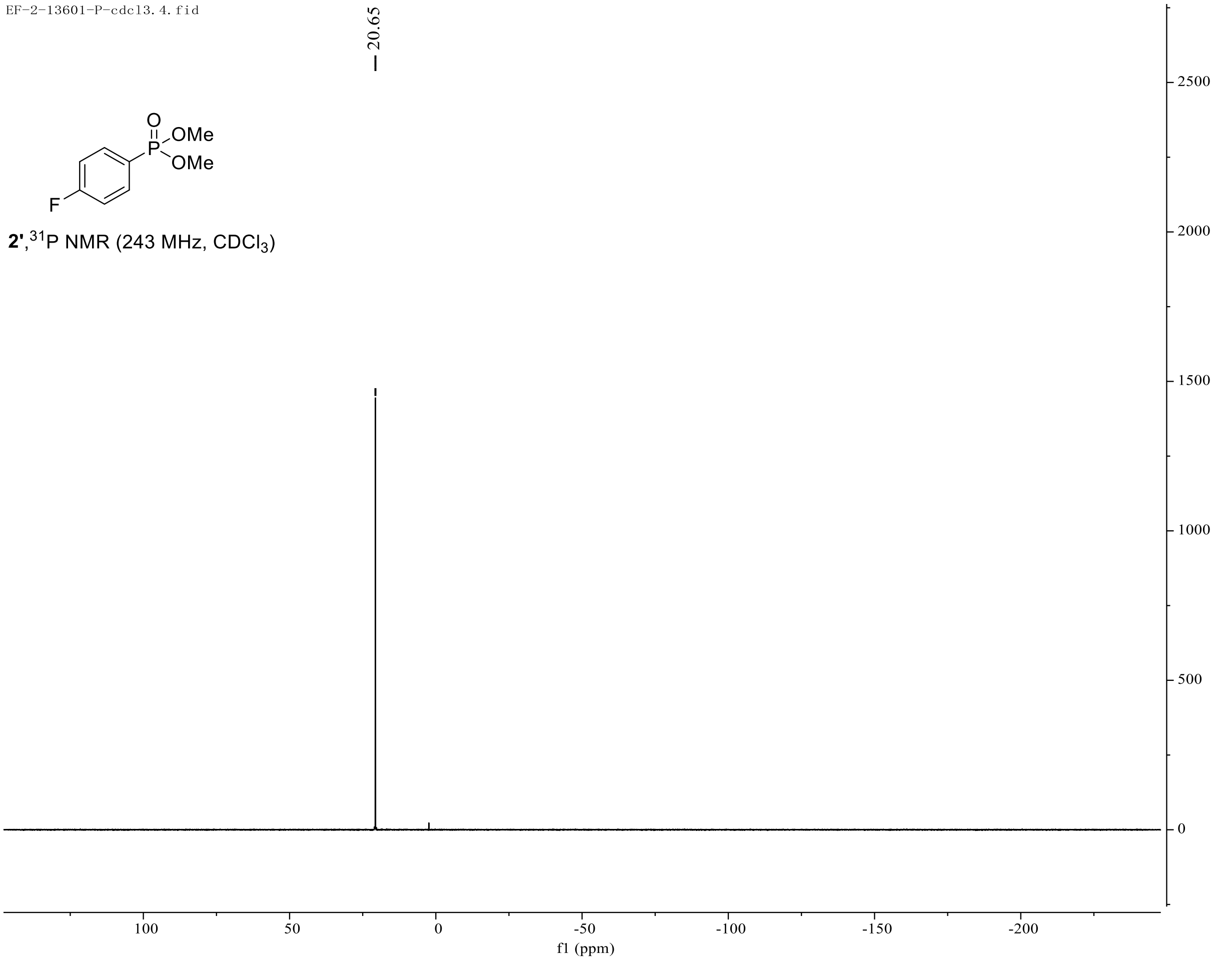

2', ^{31}P NMR (243 MHz, $CDCl_3$)

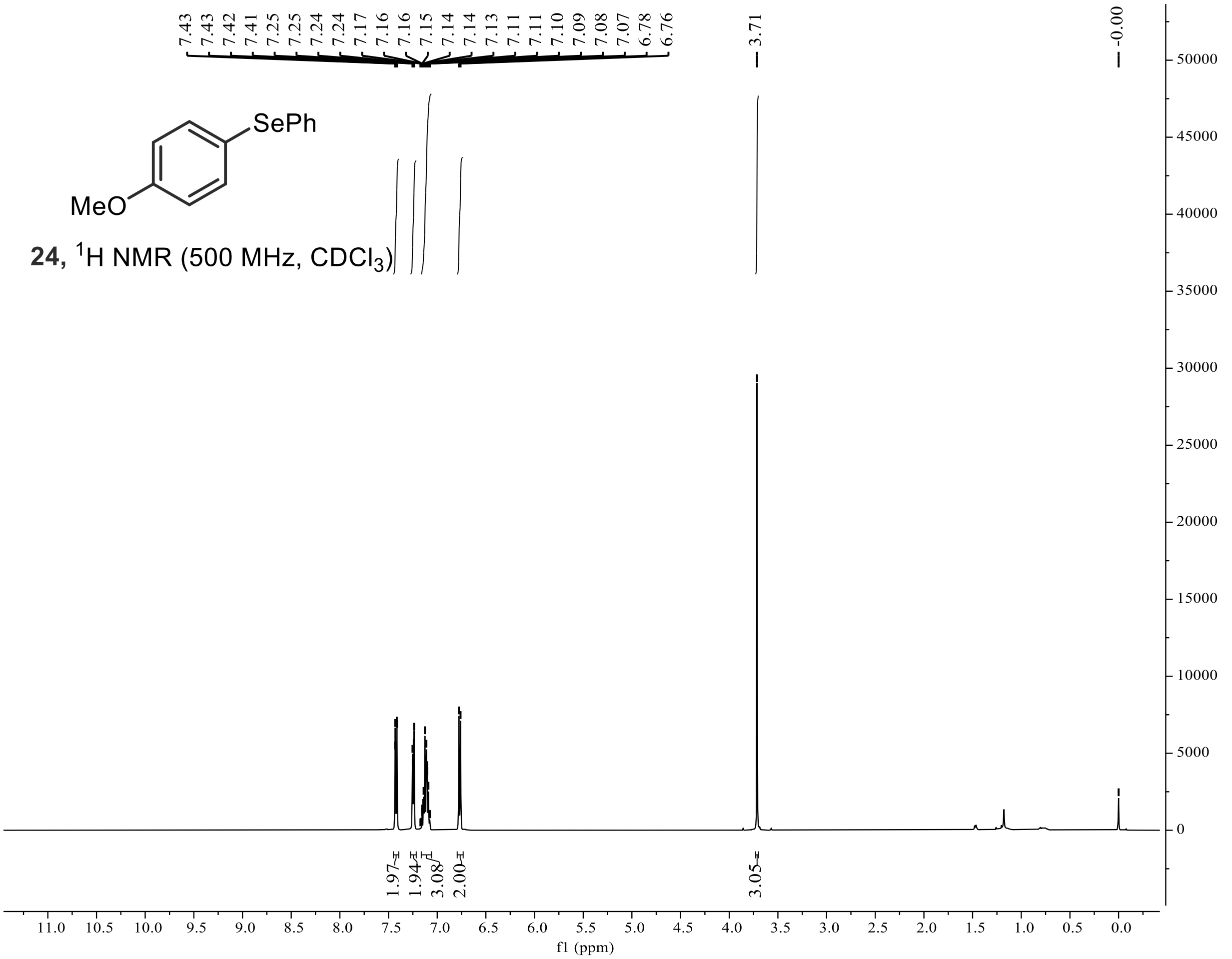

SePh
MeO
24, ¹H NMR (500 MHz, CDCl3)
7.43
7.43
7.42
7.41
7.25
7.25
7.24
7.24
7.17
7.16
7.16
7.15
7.14
7.14
7.13
7.11
7.11
7.10
7.09
7.08
7.07
6.78
6.76
3.71
-0.00
1.97
1.94
3.08
2.00
3.05
f1 (ppm)

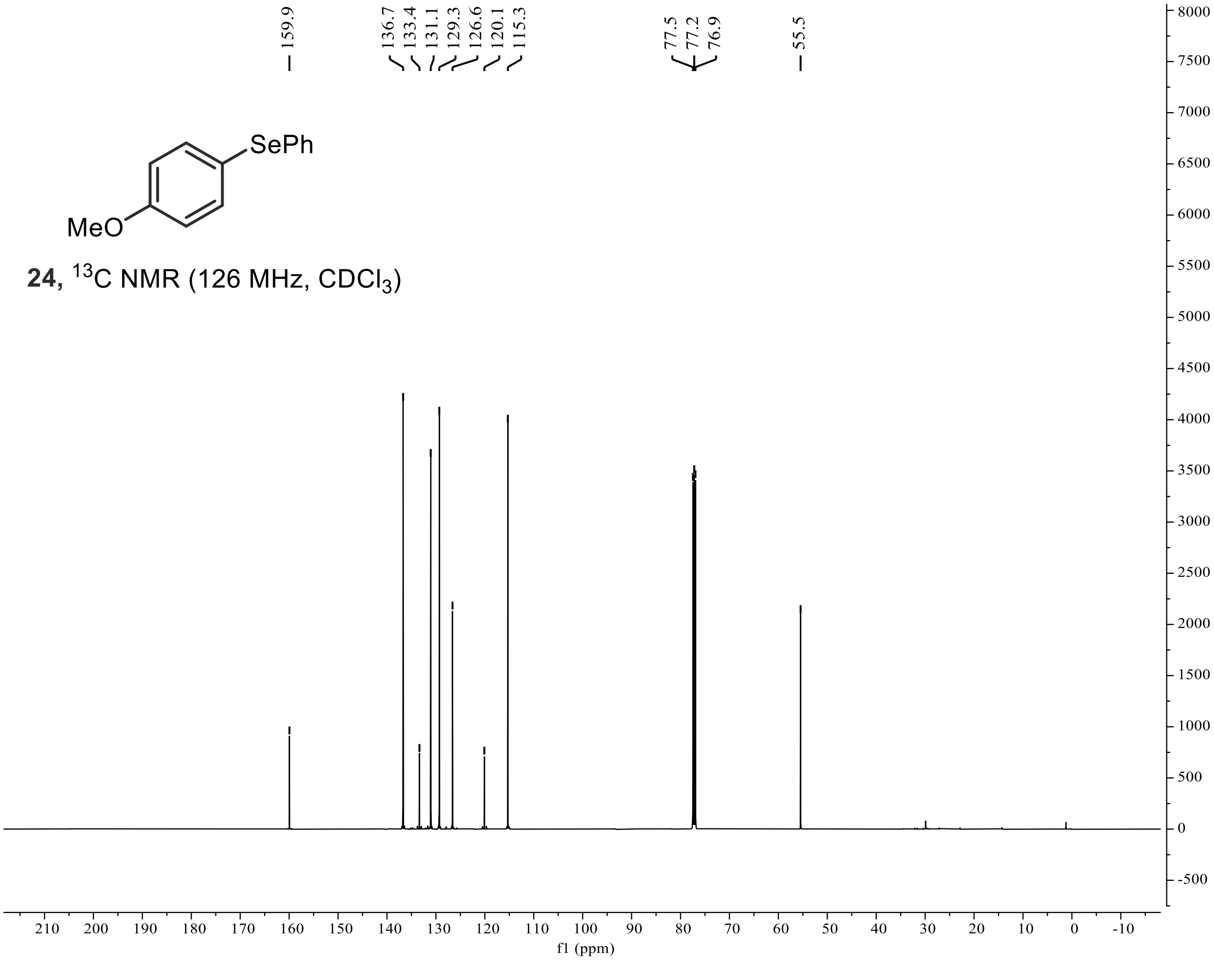

24, ^{13}C NMR (126 MHz, $CDCl_3$)

10. Reference

[1] T. Miao, L. Wang, *Advanced Synthesis & Catalysis* **2014**, *356*, 967-971.

[2] Y. He, H. Wu, F. D. Toste, *Chemical Science* **2015**, *6*, 1194-1198.

[3] Y.-X. Chen, S. Zhang, Y.-J. Xue, L.-P. Mo, Z.-H. Zhang, *Applied Organometallic Chemistry* **2022**, *36*, e6480.

[4] C. Liu, M. Szostak, *Angewandte Chemie International Edition* **2017**, *56*, 12718-12722.

[5] M. Boudjelel, J. Zhong, L. Ballerini, I. Vanswearingen, R. Al-Dhufari, C. A. Malapit, *Angew Chem Int Ed Engl* **2024**, *63*, e202406203.

[6] W. Xu, G. Hu, P. Xu, Y. Gao, Y. Yin, Y. Zhao, *Advanced Synthesis & Catalysis* **2014**, *356*, 2948-2954.

[7] F. Siméon, P.-A. Jaffrès, D. Villemin, *Tetrahedron* **1998**, *54*, 10111-10118.

[8] A. Gallego-Gamo, D. Reyes-Mesa, A. Guinart-Guillem, R. Pleixats, C. Gimbert-Suriñach, A. Vallribera, A. Granados, *RSC Advances* **2023**, *13*, 23359-23364.

[9] R. Zhuang, J. Xu, Z. Cai, G. Tang, M. Fang, Y. Zhao, *Organic Letters* **2011**, *13*, 2110-2113.

[10] R. S. Shaikh, I. Ghosh, B. König, *Chemistry – A European Journal* **2017**, *23*, 12120-12124.

[11] H. Zeng, Q. Dou, C.-J. Li, *Organic Letters* **2019**, *21*, 1301-1305.
